# Improving knowledge and changing behavior towards guideline based decisions in diabetes care: a controlled intervention study of a team-based learning approach for continuous professional development of physicians

**DOI:** 10.1186/1756-0500-6-14

**Published:** 2013-01-15

**Authors:** Lisa Kühne-Eversmann, Martin R Fischer

**Affiliations:** 1 Medizinische Klinik und Poliklinik IV, Klinikum der Universität München, Ziemssenstr. 1, Munich 80336, Germany

**Keywords:** Team-based learning, Continuing professional development, Continuing medical education, Postgraduate education, Guideline adherence, Objective behavior change

## Abstract

**Background:**

Continuing Professional Development (CPD) courses should ideally improve a physician’s knowledge and change their professional behavior in daily practice towards a best clinical practice reference model and guideline adherence. Interactive methods such as team-based learning and case-based learning, as compared to lectures, can impart sustainable knowledge and lead to high satisfaction among participants. We designed an interactive case-based CPD-seminar on diabetes care using a team-based learning approach to evaluate whether it leads to an improvement of short-term knowledge and changing of behavior towards guideline based decisions and how this learning approach is perceived by participants.

**Methods:**

Questionnaires and an electronic voting system were used to evaluate motivation, acceptance and knowledge of voluntary participants. Furthermore, we analyzed data on index diagnostic tests and referrals of patients with diabetes of participating physicians over a period of six months before and after the course in comparison with a matched control group in a quasi-experimental design.

**Results:**

Participants (n=103) rated the interactivity and team-based discussions as the main reasons for enhanced learning. They also expected that the course would change their professional behavior. Participants scored a mean of 43.9% right answers before and 62.6% after the course (p<0.001). The referral to diabetes specialists increased by 30.8% (p<0.001). Referral for fundoscopy also increased (8.5%, n.s.) while it dropped in the control group. Furthermore, the participating physicians tested their patients more often for microalbuminuria (7.1%, n.s.).

**Conclusions:**

Our team-based learning CPD-approach was highly accepted and resulted in an increase of short-term knowledge. It significantly increased the referral to diabetes specialists in daily practice whereas all other key professional behavior indicators did change but not significantly.

## Background

For several years, physicians practicing in Germany and other countries have been required to participate regularly in clinical training and earn CPD credit points, after their specialization. This lifelong training in medicine is called *continuing medical education* (CME) or *continuing professional development* (CPD). Formal CPD courses for physicians are wide-spread. Several authors examined their effectiveness concerning objective behavior change and enhanced patient outcomes in review articles [1-9]. In the most recent Cochrane Database review Forsetlund et al. [2] concluded that educational meetings can improve professional practice and patient outcomes. The effect is relatively small and similar to other formats of CPD activities such as audit and feedback and educational outreach visits. The effectiveness of interventions increases if mixed interactive and didactic formats are used, outcomes that are perceived as serious by the physicians are focused on and attendance at educational meetings is high. Other authors [3-6] came to a similar conclusion in their reviews of the effectiveness of CPD courses.

There is good evidence to suggest that interactive methods such as team-based learning and case-based learning, as compared to lectures, can impart sustainable knowledge and performance change and lead to high satisfaction among participants [9-11]. We selected the team-based learning approach because it allows for flexible small group formation in large group settings and has been proven to activate the pre-knowledge of participants and to lead to high-quality learning groups. It was first described by Michaelsen and colleagues [12]. They defined team-based learning as an instructional strategy that is based on procedures of developing high behavior learning teams that can dramatically enhance the quality of learning. In team-based learning activities each group formulates its own responses to the problems posed. Then an expert leads a comparison of the different responses by the groups and offers feedback on the quality of their responses. Case-based learning is an educational method that is based on a practical or theoretical problem which the learners can solve on their own. This strategy allows for self-structured learning in small groups in a realistic environment. In medicine the problem usually consists of a patient case [13].

Unfortunately, few CPD courses in Germany are interactive or case-based. Currently most certified CPD courses follow the traditional lecture format with subsequent discussion, producing no significant change in physician behavior [1,8,14]. Therefore there is a strong need for high-quality CPD courses.

We designed an interactive, team-based CPD concept and launched it in a series of seminars on endocrinology and diabetes care. After a pilot phase, the design of the CPD course was applied to a series of CPD activities on topics of internal and general medicine. Several courses with numerous participants from Munich and the surrounding area took place and were evaluated [15]. The course on diabetes care is evaluated in the present study. Our aim was to design a CPD course with augmented activation of the participating physicians. To achieve this we used pre- and post-course knowledge tests and case discussions in small groups as a key feature of the course. We used a team-based learning approach with carefully prepared patient cases as problems for case discussions to allow for activation of pre-knowledge and self-directed learning. The lecture format was only used in a short introduction of the topic by an expert and an evidence-based summary in the end of the course. We evaluated the acceptance of the new course and its learning outcomes as improved short-term knowledge and change in practical behavior towards guideline adherent decisions with respect to key learning objectives of participating physicians. The key learning objectives of the course we based on the guidelines of the German Diabetes Association [16] on management of diabetes care regarding regular diagnostic tests and referrals to specialists (ophthalmologist, diabetes specialist). A few facts have to be explained about the health care system in Germany concerning the referrals of patients to a specialist. Patients with diabetes can independently see a specialist without referral from their general practitioner. But this is unlikely because patients have to pay a special fee of €10 for each visit per quarter unless they have a referral from their GP and furthermore because of the mostly low compliance of patients with diabetes in Germany [17]. This is in addition to the fact that the physicians could have made a referral to specialists but the patients chose not to go.

## Methods

### Design of the CPD course

The CPD course is characterized by the following features: voluntary, interactive, evidence-based, and case-based related to medical practice, innovative in didactic terms and independent of pharmaceutical influences. The learning and teaching methods included team-based and case-based learning, a formal lecture by the expert and plenary discussions. The content of the CPD course on diabetes was the management of patients with type 2 diabetes in daily practice.

**Figure 1:**
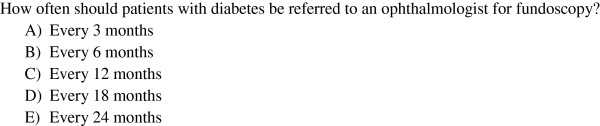
**(Pre- and post-course knowledge test).** How often should patients with diabetes be referred to an ophthalmologist for fundoscopy? **A**) Every 3 months, **B**) Every 6 months, **C**) Every 12 months, **D**) Every 18 months, **E**) Every 24 months.

The interactive seminars comprise five hours of teaching on two topical units with three to four key learning goals for guideline adherent behavior in each topic. They begin and end with a knowledge test with multiple-choice (MC) questions in the single best answer format using an electronic audience response system (see Figure 1). This technology has been shown to improve interaction in large groups and enhance learner attention, involvement and initiate discussions. Teachers found it useful for obtaining immediate results and thereby receiving feedback on their teaching [18,19]. After a short introduction of the topic by an expert (ca. 10–15 minutes), participants are divided into small groups of four to six participants. The group work uses team-based learning [12] with prepared paper cases on realistic patient problems in diabetes care and a discussion of the group’s joint solutions in a plenary session with the expert. In our CPD course, an additional evidence-based summary is presented by the expert at the end of the course (ca. 20–30 minutes).

### Instruments

Two questionnaires with 46 items were used to evaluate the characteristics, motivation, expectations, self-reported pre-course knowledge level, and validation of the CPD course participants immediately before and after the course using a Likert scale from 1 to 6 (1=*strongly disagree* to 6=*strongly agree*) and open questions. The questionnaires were developed using items of validated questionnaires of former studies of our research group regarding motivations, expectations und characteristics of participants. Then the questionnaires were validated in the pilot study.

To evaluate the pre- and post-course knowledge of the participants, ten MC-questions in single best answer format were asked at the beginning and the end of the course using an electronic audience response system. The MC-questions were written using evidence-based principles of item writing and were identical in the pre- and post-course tests.

### Participants

The total number of participants in September 2006 was 103. Course and study participation was voluntary. The participants were informed about the course and the interactive course design prior to the course in an invitation letter. Prior to the course each participant created a unique code to allow for anonymized data analysis. The age of the participants ranged from 27 to 68 years, mean: 48.4 years (*SD*=9.1). 56% of physicians were women, 44% men. The participants were mainly general practitioners and internists (60.3% vs. 22.7%) from Munich (Germany) and the surrounding area (75.2% vs. 24.8%). The mean time since their specialization was 13.0 years (*SD*= 9.0); 12.6% were residents.

### Evaluation of behavior towards guideline standards

To evaluate if an objective behavior change occurred we formulated key indicators for guideline adherent decisions of the evidence-based course content and analyzed data on diagnostics and referral practice of patients with diabetes of the participants a half year before and after the course and compared it with a matched control group. In Germany, the doctor’s billing is processed every quarter of a year and some diagnostic tests should be done once every three or six months. That is why we used a half year before and after the course for the evaluation of performance. The control group consisted of an aggregated match of general practitioners and internists in Munich and suburbs (87 of 1023, approximately 10%) with similar characteristics (e.g. number of overall treated patients). Physicians were excluded if they did not work in the whole period (January to July 2006 and 2007) or if they could be identified by certain characteristics (size of practice, specialization). We used this random sample to compare two groups of similar size. The participating physicians as well as the control group had constant numbers of patients with diabetes and showed consistent behavior regarding basic laboratory diagnostics. But it is important to note that the number of treated patients with diabetes was much higher in the intervention group. The data was provided and anonymized by the Bavarian Association of Statutory Health Insurance Physicians (“Kassenärztliche Vereinigung Bayern”) and analyzed by the first author of this article. We focused the comparative part of our study on the evaluation of professional behaviour because there are numerous studies which have shown that an interactive intervention results in a knowledge gain compared to no intervention.

## Results

### Acceptance of CPD course format

The pre-course questionnaire showed that an important reason to participate was the interactivity of the CPD course design, case discussions and the relevance of the topics for their clinical work. The participants had high expectations of a gain in knowledge and practical guidelines for decision making. They rated their own knowledge about diabetes care prior to the course as average.

The post-course questionnaire showed that they considered the contents of the course to be appropriate for their pre-knowledge and strongly agreed that the cases enhanced thinking and learning. Furthermore they highly expected that the learned topics would change their professional behavior. Additionally they appreciated the course in general and considered their expectations for the course to be fulfilled. The participants considered the guidelines and theoretical information provided sufficient (see Tables 1 and 2).

**Table 1:** Pre-course questionnaire

	**N**	**Min**	**Max**	**Mean**	**SD**
**Reasons to participate were…**					
…topical focuses of the CME course.	152	2	6	**5.29**	.93
…the interactivity and problem-based format.	148	1	6	**4.57**	1.41
…the selection of the experts.	146	1	6	**3.65**	1.60
…the time-frame of the course.	142	1	6	**4.04**	1.57
**Of this CME course I expect…**					.
…a gain in theoretical knowledge.	154	1	6	**5.39**	.95
…practical guidelines for decision making	153	1	6	**5.36**	.99
…the presentation of newest research findings.	153	1	6	**4.92**	1.20
…social contact with colleagues.	147	1	6	**3.35**	1.48
…to get to know a new design of CME course.	144	1	6	**3.28**	1.59
**I am interested in the topics of the CME course.**	153	1	6	**5.52**	.87
**I enjoy dealing with topics on endocrinology/diabetes.**	152	1	6	**5.36**	1.03
**The topics of the CME course are highly relevant for my clinical work.**	152	1	6	**5.28**	1.01
**I would like to know more about the topics of the CME course than I know now.**	150	1	6	**5.01**	1.20
**Learning more about endocrinology/diabetes is a general challenge for me.**	146	1	6	**4.75**	1.32
**I rate my knowledge about the theoretical background of endocrinology/diabetes prior to the course as low.**	145	1	6	**2.59**	1.13
**I rate my knowledge about the management of patients with endocrine diseases or diabetes mellitus prior to the course as low.**	143	1	6	**2.48**	1.23

N= Number of participants who completed the questionnaire, Min= lowest score, Max= highest score, Mean= mean score, SD= standard deviation.

Likert-Scale: 1=strongly disagree, 6=strongly agree.

**Table 2:** Post-course questionnaire, Likert-Scale: 1=strongly disagree, 6=strongly agree

	**N**	**Min**	**Max**	**Mean**	**SD**
The contents were appropriate to my pre-knowledge.	95	1	6	**4.96**	1.23
The course was diversified.	95	2	6	**5.21**	1.01
The cases enhanced thinking and learning.	95	3	6	**5.46**	.75
I learned a lot in this course	95	3	6	**5.16**	.84
What I learned will change my professional behavior and decisions	93	1	6	**4.83**	.02
The course fulfilled my expectations.	94	2	6	**5.21**	.91
I expected more practical guidelines for decision making	94	1	6	**2.60**	1.46
I expected more theoretical background information	94	1	6	**2.42**	1.47
I enjoyed the course very much	95	2	6	**5.37**	.96

N= Number of participants who completed the questionnaire, Min= lowest score, Max= highest score, Mean= mean score, SD= standard deviation.

### Knowledge gain

Our CPD course led to a significant short-term gain in the participants’ knowledge with a previously identified pre-knowledge level. In the pre-course knowledge test they achieved a mean of 43.9% right answers and in the post-course test a mean of 62.6% right answers. That means an overall improvement of knowledge about presented and discussed topics of 42.9% (SD=16.9, p<0.001). As stated in the method section we did not test the knowledge gain of the control group.

### Changes of professional behaviour

We could show that the percentage of patients referred to a diabetes specialist once a year (as recommended in the guidelines of the German Diabetes Association, 16) was significantly increased (30%) in the intervention group while it decreased in the control group. Furthermore, the referral of patients to an ophthalmologist for fundoscopy had increased by 8.5%, which was not statistically significant. In contrast, the referrals of the control group decreased. The frequency of tests for microalbuminuria also increased, but to similar degrees both in the intervention and the control group (7.1% and 10%) and with no statistical significance (Table 3). Remarkably, physicians of the intervention group had done the test twice as often as the control group even before the intervention (10% versus 5%).

**Table 3:** Objective performance change of the physicians in the intervention and control group regarding the key learning goals (*first and second quartal of 2006 and 2007)

**Key professional performance indicators**	** Intervention group**			** Control group**		
	2006*	2007*		2006*	2007*	
**Diagnosis of diabetes** (mean number of patients with diabetes per physician)	145.5	147.0	**+1% p=0.661**	87.4	87.8	**+0.5% p=0.735**
**Referral to diabetes specialist** (percentage of patients with diabetes)	7.7%	10.2%	**+30.8%p<0.001**	13.4%	13.0%	**−3% p=0.546**
**Referral to ophthalmologist for fundoscopy** (percentage of patients with diabetes)	11.8%	12.8%	**+8.5% p=0.172**	16.7%	16.2%	**−3% p=0.578**
**Tests for microalbuminuria** (percentage of patients with diabetes)	9.8%	10.5%	**+7.1% p=0.563**	5%	5.5%	**+10% p=0.5**

There was a high standard deviation of the number of patients treated, the provision of medical services and the referrals by the participating physicians. This means that their expert knowledge and behavior was possibly heterogeneous.

## Discussion

### Acceptance of CPD course format

Our findings about participating physicians’ expectations and acceptance are consistent with a study of Gerlach et al. [20] that found that physicians expect knowledge transfer with practical guidelines, opportunity for intensive discussion, imparting of the latest information, and case-based design from a high-quality CPD course. The authors conclude that there is an explicit need for CPD courses that enhance the exchange of knowledge through the activation of physicians, cover relevant clinical topics, and facilitate knowledge and competence gain at the same time. Other studies have shown that physicians still prefer traditional didactic lectures for CPD although numerous studies have shown their ineffectiveness [21,22].

### Knowledge gain

Compared to our study Qureshi et al. [23] who evaluated the effectiveness of an interactive, case-based intervention in changing the compliance with antihy-pertensive medication, showed a knowledge gain of 30.9%. Premi et al. [24] demonstrated a knowledge gain of 12.1% of the participating general practitioners through a problem-based CPD course with small group work. The control group, physicians who wanted to participate but did not get a ticket, had a knowledge gain of 2.8%. The comparison of these groups was highly significant. Unlike these studies Chan et al. [25] and Heale et al. [26] could not show an effective gain of participants’ knowledge. Both authors did randomized studies in problem-based CPD courses for general practitioners. Chan et al. compared a web-based intervention with a problem-based e-mail-course, Heale et al. a problem-based intervention in small groups with a formal CPD course (lecture followed by a discussion in the plenum). In summary, the findings of the study presented here concerning the knowledge of the participants are consistent with the conclusion of recent studies of interactive CPD courses especially those using team- and problem-based learning strategies. Compared to formal CPD interventions such as lectures which failed to change the knowledge significantly, there seem to be clear benefits for interactive interventions.

### Changes of professional behaviour

We were able to show that primary health care of patients with diabetes in our sample does not meet the guidelines in a number of instances. For example just a small percentage of patients was referred to a diabetes specialist and an ophthalmologist for fundoscopy once a year as recommended [16]. Also, the provision of medical services like tests for microalbuminuria was low and was not performed by all participating physicians.

In the most recent Cochrane Database review of the effects of continuing educational meetings and workshops Forsetlund et al. [2] identified a total of 81 trials and included 30 studies with 36 comparisons of interventions in univariate meta-regression analyses. The percentage change relative to control in compliance with desired practice was 6% (interquartile range 1.8 to 15.9) when a CME course was compared to no intervention. For continuous outcomes in professional performance the percentage change was 10% and for patient outcomes 3%. The performance changes found in our study were of a similar degree. The authors concluded that educational meetings can improve professional practice and patient outcomes but the effects tended to be small and appear to be higher when mixed interactive and didactic components are used. Furthermore the effects are lower for highly complex behaviors and less serious outcomes. This last finding of Forsetlund et al. could be an explanation for the mostly insignificant changes of the performance change targeted in our study because we focused on the general care for patients with diabetes which has serious outcomes but just in the long term.

### Limitations of the study

An important limitation of our study was that the control group included in the behaviour assessment did not participate in the knowledge assessment for practicality reasons. There are many studies which have shown that an interactive intervention results in a knowledge gain compared to no intervention. Therefore we focused the comparative part of our study on the evaluation of professional behaviour. Furthermore, the sample size for assessing real life behaviour data was relatively small and there surely was a selection bias. Another limitation of our study design is that a possible reason why the doctors who attended the CPD session were more adherent to the guidelines is that they are likely more interested in diabetes care than the control group. Nevertheless, our data showed that the guideline adherence was poor in both group but is was improved in the intervention group. Furthermore, we did not apply methods of direct observation of professional behaviour like audits. Finally, we are aware of the fact, that there are numerous confounding variables on top of our CPD intervention that influence the professional behaviour of the physicians in our sample. We tried to control for these confounders by matching controls but we are aware that this is not as powerful as a true randomization of a larger group of physicians.

## Conclusions

The team-based learning approach offers a promising format for designing effective CPD-courses. It was highly accepted by participants, resulted in an increase of short-term knowledge and our results are at least suggestive of behavioral changes towards guideline based decisions. In summary, the findings of this study concerning the acceptance and knowledge gain of par-ticipants are consistent with the data from other recent studies on interactive CPD courses especially those using team- and problem-based learning strategies. The professional performance of the participating physicians in our study has been improved in daily practice for two key indicators for guideline adherent decisions (referral to fundoscopy, test for microalbuminuria), with one additional statistically significant change (referral to diabetes specialists). Even statistically not significant changes in guideline adherent decisions could have an important impact on patient outcomes. In summary, our data showed that the primary health care of patients with diabetes is not fully compliant with guidelines and that we need further incentives to evaluate the process of changing professional behavior towards more guideline adherent decisions. Furthermore, the transfer of knowledge acquisition in CPD courses into daily medical practice and patient and health care outcomes needs more attention and support. In our perception a promising strategy is to use the theories about transfer of knowledge proved to be effective in pedagogical and psychological research and implement and evaluate them in medical education. Barnett and Ceci [27] provide a very helpful framework for far transfer and principles to teach for successful transfer.

### Availability of supporting data

We added our supporting data as Additional file 1. It contains the doctoral thesis of the corresponding author written in German: “Fallbasierte interaktive Fortbildung für Allgemeinmediziner und Internisten: Akzeptanz, Lernerfolg und Verhaltensänderung durch ein modifiziertes Team-based Learning-Konzept”.

## Competing interests

The authors declare that they have no competing interests.

## Authors’ contributions

LKE served as the principal investigator in this work and was responsible for the study conception and design, the collection, analysis and interpretation of the data, and the drafting of the manuscript. MF substantially contributed to the study design, and the acquisition, analysis and interpretation of the data. All authors contributed to the critical revision of the manuscript and approved the final version for publication. The authors are funded by the university hospital in Munich.

## Supplementary Material

Additional file 1 Doctoral thesis of corresponding author (German): "Fallbasierte interaktive Fortbildung für Allgemeinmediziner und Internisten: Akzeptanz, Lernerfolg und Verhaltensänderung durch ein modifiziertes Team-based Learning-Konzept". Click here for file

## References

[B1] Davis D O'Brien MA Freemantle N Wolf FM Mazmanian P Taylor-Vaisey A Impact of formal continuing medical education: do conferences, workshops, rounds, and other traditional continuing education activities change physician behavior or health care outcomes? JAMA 1999 282 9 867 874 10.1001/jama.282.9.867 10478694

[B2] Forsetlund L Bjørndal A Rashidian A Jamtvedt G O'Brien MA Wolf F Davis D Odgaard-Jensen J Oxman AD Continuing education meetings and workshops: effects on professional practice and health care outcomes Cochrane Database Syst Rev 2009 15 2 CD003030 19370580 10.1002/14651858.CD003030.pub2 PMC7138253

[B3] Davis D Galbraith R Continuing Medical Education Effect on Practice Performance: Effectiveness of Continuing Medical Education: American College of Chest Physicians Evidence-Based Educational Guidelines Chest 2009 135 42S 48S 10.1378/chest.08-2517 19265075

[B4] Davis D Does CME work? An analysis of the effect of educational activities on physician performance or health care outcomes Int J Psychiatry Med 1998 28 1 21 39 10.2190/UA3R-JX9W-MHR5-RC81 9617647

[B5] Marinopoulos SS Dorman T Ratanawongsa N Wilson LM Ashar BH Magaziner JL Miller RG Thomas PA Prokopowicz GP Qayyum R Bass EB Effectiveness of continuing medical education Evid Rep Technol Assess (Full Rep) 2007 149 1 69 17764217 PMC4781050

[B6] O’Neil KM Addrizzo-Harris DJ Continuing Medical Education Effect on Physician Knowledge Application and Psychomotor Skills: Effectiveness of Continuing Medical Education: American College of Chest Physicians Evidence-Based Educational Guidelines Chest 2009 135 37S 41S 10.1378/chest.08-2516 19265074

[B7] Sohn W Ismail AI Tellez M Efficacy of educational interventions targeting primary care providers’ practice behaviours: An overview of published systematic reviews J Public Health Dent 2004 64 3 164 172 10.1111/j.1752-7325.2004.tb02747.x 15341140

[B8] Oxman AD Thomson MA Davis DA Haynes RB No magic bullets: a systematic review of 102 trials of interventions to improve professional practice Can Med Assoc J 1995 153 10 1423 1431 7585368 PMC1487455

[B9] Smits PB Verbeek JH de Buisonjé CD Problem based learning in continuing medical education: A review of controlled evaluation studies BMJ 2002 324 153 156 10.1136/bmj.324.7330.153 11799034 PMC64518

[B10] Kiessling A Henriksson P Efficacy of case method learning in general practice for secondary prevention in patients with coronary artery disease: Randomised controlled study BMJ 2002 325 877 880 10.1136/bmj.325.7369.877 12386042 PMC129638

[B11] Kiessling A Lewitt M Henriksson P Case-based training of evidence-based clinical practice in primary care and decreased mortality in patients with coronary heart disease Ann Fam Med 2011 9 211 218 10.1370/afm.1248 21555748 PMC3090429

[B12] Michaelsen LK Team-Based Learning: A Transformative Use of Small Groups 2002 Westport Conn and London: Praeger

[B13] Williams B Case-based learning – a review of the literature: is there scope for this educational paradigm in prehospital education? Emerg Med J 2005 22 577 581 10.1136/emj.2004.022707 16046764 PMC1726887

[B14] Bloom BS Effects of continuing medical education on improving physician clinical care and patient health: a review of systematic reviews Int J Technol Assess Health Care 2005 21 3 380 385 16110718 10.1017/s026646230505049x

[B15] Kühne-Eversmann L Eversmann T Fischer MR Team- and case-based learning to activate participants and enhance knowledge: an evaluation of seminars in Germany J Contin Educ Health Prof 2008 28 3 165 171 10.1002/chp.175 18712804

[B16] http://www.deutsche-diabetes-gesellschaft.de

[B17] Möbes J Compliance: Neue Positionen am Beispiel des Diabetes mellitus Z Allg Med 2003 79 238 243

[B18] Collins J Audience response systems: technology to engage learners J Am Coll Radiol 2008 5 9 993 1000 10.1016/j.jacr.2008.04.008 18755440

[B19] Jensen JV Ostergaard D Faxholt AK Good experiences with an audience response system used in medical education Dan Med Bull 2011 58 11 A4333 22047931

[B20] Gerlach FM Beyer M Ärztliche Fortbildung aus der Sicht niedergelassener Ärztinnen und Ärzte—representative Ergebnisse aus Bremen und Sachsen-Anhalt [Continuing medical education from the view of ambulatory care physicians--representative outcomes and needs in Bremen and Saxony-Anhalt Z Arztliche Fortbildung und Qualität 1999 93 581 589 10596039

[B21] Stancic N Mullen PD Prokhorov AV Frankowski RF McAlister AL Continuing medical education: What delivery format do physicians prefer? J Contin Educ Health Prof 2003 23 162 167 10.1002/chp.1340230307 14528787

[B22] Kelly MH Murray TS General practitioners’ view on continuing medical education Br J Gen Pract 1994 44 469 471 7748636 PMC1239022

[B23] Qureshi NN Hatcher J Chaturvedi N Jafar TH Hypertension Research Group Effect of general practitioner education on adherence to antihypertensive drugs: cluster randomised controlled trial BMJ 2007 335 7628 1030 10.1136/bmj.39360.617986.AE 17991935 PMC2078673

[B24] Premi J Shannon S Hartwick K Lamb S Wakefield J Williams J Practice-based small-group CME Acad Med 1994 69 10 800 802 10.1097/00001888-199410000-00002 7916790

[B25] Chan DH Leclair K Kaczorowski J Problem-based small-group learning via the Internet among community family physicians: a randomized controlled trial MD Comput 1999 16 3 54 58 10439603

[B26] Heale J Davis D Norman G Woodward C Neufeld V Dodd P A randomized controlled trial assessing the impact of problem-based versus didactic teaching methods in CME Res Med Educ 1988 27 72 77 3218878

[B27] Barnett SM Ceci SJ When and where do we apply what we learn? A taxonomy for far transfer Psychol Bull 2002 128 4 612 637 12081085 10.1037/0033-2909.128.4.612

