# Supplementary material for: Improving knowledge and changing behavior towards guideline based decisions in diabetes care: a controlled intervention study of a team-based learning approach for continuous professional development of physicians
Source: BMC Res Notes. 2013 Jan 15;6:14. doi: 10.1186/1756-0500-6-14 (PMC3574038; doi:10.1186/1756-0500-6-14)
Supplement: Additional file 1 — Doctoral thesis of corresponding author (German): "Fallbasierte interaktive Fortbildung für Allgemeinmediziner und Internisten: Akzeptanz, Lernerfolg und Verhaltensänderung durch ein modifiziertes Team-based Learning-Konzept". [file 1756-0500-6-14-S1.pdf]

Aus der Medizinische Klinik Innenstadt
der Ludwig-Maximilians-Universität München

Direktor: Prof. Dr. med. M. Reincke

in Zusammenarbeit mit dem
Schwerpunkt Medizindidaktik

Leiter: Prof. Dr. med. M. Fischer

Fallbasierte interaktive Fortbildung für Allgemeinmediziner und Internisten: Akzeptanz, Lernerfolg und Verhaltensänderung durch ein modifiziertes Team-based Learning-Konzept

Dissertation
zum Erwerb des Doktorgrades der Medizin
an der Medizinischen Fakultät der
Ludwig-Maximilians-Universität, München

vorgelegt von
Lisa Kühne-Eversmann
aus München
2010

Mit Genehmigung der Medizinischen Fakultät
der Ludwig-Maximilians-Universität München

1. Berichterstatter:	Prof. Dr. med. Martin Reincke
Mitberichterstatter:	Priv. Doz. Dr. Martin Weigl<br>Prof. Dr. Dennis Nowak
Mitbetreuung durch die promovierten Mitarbeiter:	Prof. Dr. med. Martin Fischer
Dekan:	Prof. Dr. med. Dr. h.c. M. Reiser, FACR, FRCR

Tag der mündlichen Prüfung:21. Januar 2010

Danksagung

Diese Arbeit hätte ohne die Unterstützung vieler Personen nicht realisiert werden können. Deshalb danke ich allen Beteiligten sehr herzlich für ihre wichtigen Ratschläge und konstruktive Kritik, auch denjenigen, die ich hier nicht namentlich nennen kann.

Ich danke insbesondere meinem Betreuer und Doktorvater Prof. Dr. Martin Fischer, durch den ich das hochinteressante und viel versprechende Forschungsgebiet der Medizindidaktik kennen lernen konnte, das jetzt auch ein Schwerpunkt meiner beruflichen Weiterbildung ist. Durch sein Engagement, seine konstruktive Kritik und persönliche sowie fachliche Unterstützung konnte ich schwierige Phasen der Doktorarbeit überwinden.

Meiner Arbeitsgruppe, dem Schwerpunkt Medizindidaktik der Medizinischen Klinik Innenstadt, schulde ich besonderen Dank für die Hilfe und Unterstützung bei der Entwicklung, Auswertung und stets fruchtbaren Diskussion über die Ergebnisse und Schlussfolgerungen meiner Arbeit. Dem Direktor der Klinik Prof. Dr. Martin Reincke danke ich herzlich für das Interesse und die Unterstützung meiner Forschungsarbeit.

Ich möchte allen Studienteilnehmern, Dozenten, Moderatoren und Organisatoren der Fortbildungsveranstaltungen für ihre Mitarbeit, Evaluationen und Verbesserungsvorschläge danken. Den Mitarbeitern der Kassenärztlichen Vereinigung Bayern, insbesondere Anne-Kathrin Pfeifer, Gabriele Ulrich und Martin Tauscher, für die Möglichkeit, die anonymisierten Daten der Fortbildungsteilnehmer auszuwerten. Ersterer gebührt mein Dank besonders für die Hilfsbereitschaft bei der statistischen Auswertung und der kritischen Diskussion der Analyse.

Schließlich möchte ich meiner Familie danken. Meinem Mann Florian vor allem für die moralische und auch technische Unterstützung, meiner Mutter für die sprachlichen und inhaltlichen Anregungen und meinem Vater besonders für die fachliche Hilfe.

Teile dieser Arbeit wurden veröffentlicht:

Kühne-Eversmann L, Eversmann T, Fischer MR. Team- and case-based learning to activate participants and enhance knowledge: an evaluation of seminars in Germany. J Contin Educ Health Prof. 2008 Summer; 28(3):165-71.

Eversmann L, Eversmann Th, Saller B, Fischer MR (2006). Activation of CME-course participants through group learning and online cases: a modified team-based approach. Abstractband der AMEE-Jahrestagung, Genua, 2006.

Kühne-Eversmann L, Eversmann T und Fischer MR (2007). Ein innovatives Blended Learning-Konzept in der ärztlichen Fortbildung: Teilnehmeraktivierung und Wissenszuwachs in der Präsenzphase und ein Integrationskonzept für Onlinefälle. In G. Kundt, J. Bernauer, M. Fischer, M. Haag, R. Klar, J. Leven, H. Matthies, F. Puppe (Hrsg.), eLearning in der Medizin und Zahnmedizin. Aachen: Shaker

Kühne-Eversmann L, Pfeifer AK, Eversmann T, Fischer MR. Ärztliche Fortbildung: Wissenszuwachs und objektive Verhaltensänderung durch das Team-based Learning-Konzept des Hausärztlichen Curriculums München (HCM). GMA Jahreskonferenz 2008, Tagungsband.

Kühne-Eversmann L, Pfeifer AK, Eversmann T, Seissler J, Fischer MR. Interaktives Fortbildungskonzept im Rahmen des Hausärztlichen Curriculums München (HCM) führt zu Wissenszuwachs und Verhaltensänderung bei der Betreuung von Patienten mit Diabetes mellitus. Abstractband der Jahrestagung der Deutschen Diabetesgesellschaft, Leipzig, 2009.

CME for physicians on diabetes care: Improving knowledge and changing behaviour through a team-based learning approach. Lisa Kühne-Eversmann, Anne-Kathrin Pfeifer, Thomas Eversmann. Martin R . Fischer. Vortrag auf AMEE-Jahrestagung 2009 (29.8.-3.9.2009).

Inhaltsverzeichnis:

I. Einleitung 7
1. Ärztliche Fortbildung 9
1.1. Hintergrund und Grundlagen 9
1.2. Fortbildungsformate und ihre Effektivität 15
1.2.1. Präsenzveranstaltungen: Vorträge, Workshops, Seminare 17
1.2.2. Selbststudium: Fachzeitschriften, E-Learning 19
1.2.3. Klinische Fortbildung: Audit and Feedback 26
1.3. Lehr- und Lernstrategien für effektive Fortbildungen 32
1.3.1. Team-based Learning 32
1.3.2. Problem-based learning 36
1.3.3. Evidenzbasierte Medizin 41
2. Ziele und Fragestellung 45

II. Methoden 48
1. Pilotstudie 48
2. Hauptstudie: Teilnehmercharakteristika 53
3. Studiendesign 54
4. Beschreibung des Fortbildungskonzeptes 55
4.1. Präsenzveranstaltung 55
4.2. Aufbau des Syllabus 58
4.3. Daten zum Diagnose-, Leistungs- und Überweisungsverhalten 60
5. Instrumente: 64
5.1. Fragebögen: 64
5.2. Vor- und Nachwissenstests: 65
5.3. Lernfälle: 66
5.4. Datenauswertung zum Diagnose-, Leistungs- und Überweisungsverhalten: 66

III. Ergebnisse 67
1. Präsenzveranstaltungen 67
1.1. Ergebnisse der Eingangsevaluation 67
1.1.1. Fortbildungsverhalten der Teilnehmer: 67
1.1.2. Motivation, Erwartungen und Selbsteinschätzung der Teilnehmer: 69
1.1.3. Computerkenntnisse und -nutzung: 71
1.2. Ergebnisse der Abschlussevaluation 74
1.2.1. Akzeptanz der Teilnehmer: 74
1.2.2. Interesse an Onlinefällen: 75
1.3. Vor- und Nachwissenstests: 76
2. Daten zum Diagnose-, Leistungs- und Überweisungsverhalten 77
2.1. Teilnehmer der Fortbildung Sekundäre Hypertonie 77
2.2. Teilnehmer der Fortbildung Schilddrüsenerkrankungen 78
2.2.1. Diagnose Schilddrüsenerkrankung und Unterdiagnosen 78
2.2.2. Durchführung einer Schilddrüsensonographie bei Patienten mit Schilddrüsenerkrankungen: 79
2.2.3. Überweisung von Patienten mit Schilddrüsenerkrankungen zur Schilddrüsensonographie: 81
2.2.4. Überweisung von Patienten mit Schilddrüsenerkrankungen zur Schilddrüsenszintigraphie (GOP 17320): 83
2.2.5. Überweisung von Patienten mit Schilddrüsenerkrankungen zur Farbduplexsonographie (GOP 33075): 84

2.3. Teilnehmer der Fortbildung Diabetes mellitus im Vergleich mit einer Kontrollgruppe 85
2.3.1. Diagnose Diabetes mellitus und Unterdiagnosen 85
2.3.2. Leistungsverhalten der Ärzte: 88
2.3.3. Abrechnungsfrequenz der Laborpauschale manifester Diabetes 89
2.3.4. Frequenz von Mikroalbuminurie-Tests 90
2.3.5. Therapie des Diabetischen Fußsyndroms 93
2.3.6. Überweisungsfrequenz zur Funduskopie zum Facharzt für Augenheilkunde ... 93
2.3.7. Überweisungsfrequenz zum Diabetologen 97
2.4. Zusammenfassung des Vergleichs des Verhaltens der Teilnehmer der Fortbildung Diabetes mit der Kontrollgruppe 99

IV. Diskussion und Ausblick 105
1. Diskussion der Ergebnisse 105
2. Stärken und Limitationen der vorliegenden Studie 120
3. Ausblick 131
4. Zusammenfassung: 136

V. Literatur: 138

VI. Anhang 150
Anhang 1 – Eingangsfragebogen 150
Anhang 2 – Abschlussfragebogen 153
Anhang 4 - Instruktionen für Referenten und Moderatoren 155
Anhang 5 - Themen bereits besuchter Fortbildungs-veranstaltungen bzw. geplanter Fortbildungen 156
Anhang 6 - Erstellung der Onlinefälle: 157

VII. Lebenslauf 158

I. Einleitung

Ärztliche Fort- und Weiterbildung – es besteht erheblicher Forschungsbedarf:

Seit einigen Jahren sind alle Ärzte in Deutschland nach Abschluss der Facharztausbildung verpflichtet, sich regelmäßig fortzubilden (BAEK 2006). Dieses lebenslange Lernen wird in der Medizin auch als Continuing Medical Education (ACCME) oder Continuing Professional Development (CPD) bezeichnet. Durch die Einführung der Fortbildungspflicht für Ärzte besteht ein hoher Bedarf an hochwertigen und flexiblen Angeboten.

Es gibt zahlreiche Hinweise in der Literatur, dass interaktive Methoden im Vergleich zum reinen Frontalvortrag neues Wissen besser und dauerhafter vermitteln können (Oxman, Thomson et al. 1995; Davis, O'Brien et al. 1999; Thomson O'Brien, Freemantle et al. 2001; Sohn, Ismail et al. 2004). Leider gibt es immer noch einen Mangel an interaktiven, fallorientierten Fortbildungsformaten. Die meisten der von den Landesärztekammern zertifizierten CME-Angebote sind derzeit Frontalvorträge mit anschließender Diskussion, die in Studien keinen signifikanten Wissenszuwachs bei den Teilnehmern gezeigt haben (Bloom 2005).

Aufgrund der wachsenden Zahl an wissenschaftlichen Erkenntnissen in den letzten Jahrzehnten, sind Ärzte dazu gezwungen, sich laufend zu informieren und regelmäßig wissenschaftliche Publikationen zu lesen, um auf dem aktuellen Wissensstand zu bleiben. Die Halbwertszeit medizinischen Wissens beträgt nach Schätzungen derzeit fünf bis zehn Jahre (Hall and Platell 1997; Masic, Novo et al. 2007), das bedeutet, dass in drei bis vier Jahren etwa 50% des aktuellen Wissens falsch oder überholt sein werden (Masic, Novo et al. 2007) oder dass das medizinische Wissen sich alle sechs bis acht Jahre verdoppelt (Mantovani, Castelnuovo et al. 2003).

Schätzungen gehen davon aus, dass täglich durchschnittlich 1,4 Fachfragen pro Patient während eines stationären Aufenthaltes auftreten, die sich dem behandelnden Arzt stellen. In einer Hausarztpraxis ist es dagegen nur eine Frage bei jedem 15. Patienten (Anderson, Casebeer et al. 1999). Eine andere Untersuchung bei niedergelassenen Allgemeininternisten zeigte, dass täglich 12 Fragen zum Management des Patienten

auftauchen, bei denen nur in 33% eine Antwort gesucht wird (Peterson, Galvin et al. 1999). In einer weiteren Studie ergab sich in mehr als der Hälfte der Patientenbesuche eine Frage zur Versorgung; nur in 30% der Fälle verfolgten die Ärzte diese weiter und konnten so 24% der Fragen beantworten (Gorman and Helfand 1995). Eine der größten Herausforderungen für den Arzt ist es dabei, die richtige Antwort möglichst zuverlässig und schnell zu finden. Dabei vertrauen viele Ärzte auf die Meinung von Kollegen (Osheroff, Forsythe et al. 1991; Dee and Blazek 1993), lesen Fachzeitschriften oder Lehrbücher oder nützen CME-Angebote oder das Internet (Brennan 1995; Glowniak 1995; Bennett, Casebeer et al. 2004; 2006).

Das Internet vereinfacht und beschleunigt die Informationssuche: über Suchmaschinen lassen sich innerhalb kürzester Zeit Studien zu einem bestimmten Thema über Suchmaschinen finden, die aktuellen Leitlinien der Fachgesellschaften herunterladen oder Arzneimittelnebenwirkungen und –dosierungen nachschlagen. Mit der zunehmenden Verbreitung des Internets bieten sich auch interaktive Fort- und Weiterbildungsveranstaltungen im Online-Format als didaktische Strategie an (Harden 2005). Die Vorteile einer solchen Veranstaltung liegen auf der Hand: bequem und flexibel, weitgehend unabhängig von Zeit und Raum und mit einer Vielzahl von Kommunikationsmöglichkeiten. Für praktizierende Ärzte außerhalb von Ballungsgebieten sind Online-Veranstaltungen oft die einzige Gelegenheit, sich ohne größeren zeitlichen und finanziellen Aufwand weiterzubilden (Cobb 2004; Sargeant, Curran et al. 2004; Curran and Fleet 2005). Lange wurde die Effektivität von Online-Fort- und Weiterbildung in Frage gestellt. Inzwischen konnte jedoch in einigen Studien gezeigt werden, dass gut gestaltete Online-Veranstaltungen zu einer Veränderung des Verhaltens sowie zu einer Zunahme des Wissens führen. In dieser Hinsicht sind sie Präsenzveranstaltungen vergleichbar oder sogar überlegen (Wutoh, Boren et al. 2004; Fordis, King et al. 2005).

Allerdings besteht noch ein erheblicher Forschungsbedarf zur kurz- und langfristigen Ergebnisqualität der medizinischen Fort- und Weiterbildung. Systematische Übersichtsartikel zur Effektivität von Fortbildungsangeboten haben zwar gezeigt, dass es in 70% zu einer Veränderung des Verhaltens und der Entscheidungsfindung und in 50% zu einer Verbesserung der Patientenversorgung der teilnehmenden Ärzte kommt

(Davis 1998). Die Autoren der bisherigen Reviews kritisieren jedoch meist die methodische Qualität der vorhandenen Studien (Davis, Thomson et al. 1992; Davis 1998; Thomson O'Brien, Freemantle et al. 2001). Viele Studien zur Verhaltensänderung bei Ärzten beruhen beispielsweise nur auf einer subjektiven Selbsteinschätzung.

Aufgrund des Mangels an hochwertigen Fortbildungsangeboten wurde ein neuartiges, interaktives und fallorientiertes Fortbildungskonzept entwickelt und evaluiert, das im Folgenden näher beschrieben wird.

1. Ärztliche Fortbildung

1.1. Hintergrund und Grundlagen

Um effektive Fortbildungsstrategien entwickeln zu können, ist es notwendig, die anerkannten Prinzipien der Erwachsenenbildung und die speziellen Lernstrategien und Bedürfnisse von Ärzten zu beachten. Auf diese Grundlagen soll im folgenden Abschnitt kurz eingegangen werden.

Lehren und Lernen von Erwachsenen

In der Literatur ist man sich weitgehend einig, dass Erwachsene teilweise andere Lernstrategien haben als Kinder oder Jugendliche. Also müssen einerseits die Lehrstrategien entsprechend angepasst werden, um ein effektives Lernen zu ermöglichen. Andererseits gelten viele Aspekte effektiven Lernverhaltens für alle Altersgruppen. Jannette Collins (2004) schreibt dazu in ihrer Übersichtsarbeit zu den Lehrstrategien für lebenslanges Lernen: „Erwachsene sind selbstbestimmter in ihrem Lernverhalten und haben ein größeres Bedürfnis zu wissen, warum sie etwas lernen sollen. Sie haben feste Gewohnheiten, starke Bevorzugungen und eventuell auch Vorurteile, die teilweise die Lernumgebung stören können. Außerdem wollen sie wählen können, was sie lernen.“ Malcolm Knowles (1970; 1985), der als einer der Väter der Lerntheorie bei Erwachsenen gilt, prägte den Begriff der „Andragogy“ im Gegensatz zur „Pedagogy“. Für ihn sind die Methoden der Pädagogik eher lehrerbezogen, die Methoden der „Andragogy“ eher lernerorientiert. Allerdings kam er in späteren Studien zu dem Schluss, dass es sich hierbei nicht um gegensätzliche

Lernmethoden handelt, sondern eher um Entwicklungsstufen, die nacheinander durchlaufen werden.

Collins (2004) formuliert in ihrem Artikel zehn Grundsätze des Lernverhaltens von Erwachsenen (siehe Abb. 1, Principles of Adult Learning), die Organisatoren und Leiter von Fortbildungsveranstaltungen für Erwachsene berücksichtigen sollten.

1. Adults have accumulated a foundation of life experiences and knowledge.
2. Adults are autonomous and self-directed.
3. Adults are goal-oriented.
4. Adults are relevancy-oriented and practical.
5. Adults (all learners) need to be respected.
6. Adults are motivated to learn by both intrinsic and extrinsic motivation.
7. Adults learn best when they are active participants in the learning process.
8. Not all adults learn the same way.
9. Adults learn more effectively when given timely and appropriate feedback and reinforcement of learning.
10. Adults learn better in an environment that is informal and personal.

Abb. 1: Principles of Adult Learning nach Collins (2003; 2004)

Einer der wichtigsten Grundsätze in der Erwachsenenbildung ist außerdem konstruktives Feedback, um erfolgreiches Lernen und die Meisterung neuen Wissens zu erreichen.

Slotnick (1999) untersuchte selbstbestimmte Lernaktivitäten von Ärzten im Hinblick auf die bisherigen wissenschaftlichen Erkenntnisse, wie Ärzte lernen. Die von den befragten Ärzten beschriebenen Lernerfahrungen deckten sich mit den früheren wissenschaftlichen Ergebnissen, dass zwei Arten von Problemen (allgemeine und spezielle) das Lernbedürfnis aktivieren. Jeder Problemtyp ist mit einer besonderen Lernform der Ärzte assoziiert. Diese Formen sind einerseits semi-strukturierte Lernaktivitäten (d. h. vorhandene Literatur- und Beratungsquellen), die genutzt werden, um spezifische Probleme zu lösen, und andererseits formale Lernaktivitäten (d. h. geplante Fortbildungen, Seminare oder Treffen von Fachgesellschaften), die bei

der Lösung allgemeiner Probleme im Vordergrund stehen. Diese Lernaktivitäten folgen laut Slotnick bestimmten Regeln. Zuerst wird das Problem aufgespürt (Scanning), dann entschieden, ob das Lernziel weiterverfolgt wird (Evaluation) und bei einer positiven Entscheidung neues Wissen und Fertigkeiten erlernt (Learning skills and knowledge). Schließlich wird dieses Wissen angewendet und mit weiterer Erfahrung vertieft (Gaining experience) (siehe Tabelle 1: Die 4-Stadien-Theorie von selbstbestimmten Lernaktivitäten von Ärzten). Diese Schritte sind in mehreren Studien Slotnick (1996), (Slotnick); (Moore and Klingborg 2003) untersucht und beschrieben worden. Die Lernstrategie, die zur Lösung eines konkreten Problems führt, resultiert in einer schrittweisen Zunahme des Wissens und der Fertigkeiten der Ärzte, während das Lernverhalten bei allgemeinen Problemen größere Veränderungen im Verhalten der Ärzte nach sich zieht. Dieses Lernverhalten von Ärzten resultiert notwendigerweise aus der Tatsache, dass im ärztlichen Alltag ständig spezielle und allgemeine Probleme auftauchen, die entweder einer kurz- oder auch langfristigen Lösung bedürfen.

**Stadien**	**Name**	**Definition**
**0**	Scanning	Ärzte sind sich bewusst, dass in der Praxis immer wieder Fragen und Probleme auftreten, und sind deshalb aufmerksam. Wenn ein Problem identifiziert wird, wird zur Evaluations-Stufe weitergegangen.
**1**	Evaluation	Ärzte entscheiden über das weitere Vorgehen zur Lösung des aufgetretenen Problems. Es wird entweder verworfen oder als wichtig erachtet. Dann werden zunächst schnell erreichbare Informationsquellen genutzt, beispielsweise Kollegen oder Nachschlagewerke o. ä.. Bei allgemeinen Problemen wird außerdem überlegt, mit welchen Mitteln (Fortbildungsmaßnahmen etc.) das Problem gelöst werden kann.
**2**	Learning skills and knowledge	Die Ärzte lesen (Fachzeitschriften, Lehrbücher), suchen Rat bei erfahrenen Kollegen, besuchen Fortbildungsveranstaltungen oder nutzen andere Fortbildungsangebote.
**3**	Gaining experience	Die Ärzte wenden die Lösung auf das Problem an oder erproben das erlernte Wissen und Fertigkeiten im klinischen Alltag und bilden sich wenn nötig bei allgemeinen Problemen weiter fort.

Tab. 1: Die 4-Stadien-Theorie der selbstbestimmten Lernaktivitäten von Ärzten

Fortbildungspflicht

Ein Großteil der Ärzte hat sich deshalb vor Einführung der Fortbildungspflicht bereits regelmäßig freiwillig fortgebildet. In der Berufsordnung steht zur Fortbildung (BAEK 2006): „Ärztinnen und Ärzte, die ihren Beruf ausüben, sind verpflichtet, sich in dem Umfange beruflich weiterzubilden, wie es zur Erhaltung und Entwicklung der zu seiner Berufsausübung erforderlichen Fachkenntnisse notwendig ist." Als 1995 die ersten Vorwürfe aus der Politik über mangelnde Fortbildung der Ärzte auf einer Gesundheitsministerkonferenz erhoben wurden, reagierten die Ärztekammern mit der Einführung des „Freiwilligen Fortbildungszertifikats der Ärztekammern". Dieses wurde von den Ärzten in Deutschland jedoch nur sehr zurückhaltend erworben (Herzig 2003).
Im Zuge der Gesundheitsreform 2004 wurde die Fortbildungspflicht für Ärzte erstmals bundesgesetzlich geregelt. Ärzte müssen im Rahmen ihrer Fortbildungspflicht innerhalb eines Fünf-Jahres-Zyklus bestimmte zertifizierte Fortbildungen durchlaufen. Jeder Vertrags- und Facharzt muss 250 Fortbildungspunkte (CME-Punkte) nach diesem Zeitraum nachweisen. Gemäß § 95d SGB V (GKV-Modernisierungsgesetz, (Gesundheit 2003)) verpflichtet der Gesetzgeber die Vertragsärzte, sich regelmäßig fachlich fortzubilden und die Nachweise alle fünf Jahre der Kassenärztlichen Vereinigung (KV) vorzulegen. Vertragsärzte, die zum 30. Juni 2004 zugelassen sind, müssen die Fortbildungsnachweise erstmalig bis zum 30. Juni 2009 erbringen. Für an einem Krankenhaus beschäftigte Fachärzte gilt ebenfalls eine Fünf-Jahres-Frist, sie müssen die Fortbildungsnachweise erstmalig zum 1. Januar 2011 erbringen. Ärzte, die sich nur unzureichend oder gar nicht fortbilden, müssen mit Vergütungsabschlägen oder sogar mit dem Entzug der Zulassung rechnen. Die bisher ausschließlich berufsrechtlich geregelte Fortbildungspflicht wird also zu einem Bestandteil des Zulassungsrechts. Fehlen die Nachweise, muss die KV den Vergütungsanspruch des Arztes zunächst um zehn Prozent, ab dem fünften Quartal um 25% kürzen. Fehlende Nachweise kann der Arzt innerhalb von zwei Jahren erbringen. Wird diese Frist überschritten, soll die KV unverzüglich die Entziehung der Zulassung beantragen.

Ein Teil der Gesamtpunkte (150 Punkte) muss in der eigenen Fachrichtung erworben werden. Außerdem werden 30 bzw. 50 Punkte ohne Nachweis für Selbststudium von

den Landesärztekammern anerkannt. Eine Beschränkung, mit welcher Art von Fortbildungsformaten (Online-Formaten, medizinischen Fachzeitschriften, Präsenzveranstaltungen) die Fortbildungspunkte erworben werden müssen, gibt es bei der Mehrzahl der Landesärztekammern nicht.

Zentraler Koordinator ist der Deutsche Senat für ärztliche Fortbildung, der im Auftrag der Landesärztekammern die Qualitätszertifizierung für Fortbildungsveranstaltungen verantwortet. Zertifiziert werden nur Veranstaltungen, deren Inhalte bestimmten Anforderungen genügen, die die Bundes- und Landesärztekammern festlegen. So müssen ärztliche Fortbildungen frei von wirtschaftlichen Interessen sein; produktbezogene, von der pharmazeutischen Industrie durchgeführte Veranstaltungen werden nicht als Fortbildung anerkannt. Laut Umfrage der Ärzte-Zeitung (2005) hat das Interesse der Ärzte an zertifizierten Fortbildungsveranstaltungen bereits kurz nach der Einführung der Fortbildungspflicht im ersten Quartal 2004 deutlich zugenommen.

„Wichtigstes Ziel der Fortbildung ist die Verbesserung des ärztlichen Handelns. Somit ist Fortbildung ein bedeutendes Instrument der Qualitätssicherung in der Medizin“, heißt es in den Empfehlungen zur ärztlichen Fortbildung der Bundesärztekammer (BAEK 2007). Fortbildung könne nur dann erfolgreich sein, wenn sie einerseits ein subjektiv empfundenes Bedürfnis befriedigt, andererseits aber auch auf objektive Wissens- und Handlungsdefizite eingeht. Dabei erfolge die Erarbeitung der fachlichen Kenntnisse und Fähigkeiten am besten durch systematische Fortbildung in folgenden Schritten:

1. Erkennen des Fortbildungsbedarfs
2. Festsetzung eines Zielpunktes für die Verbesserung
3. Auswahl der Lernmethode
4. Erarbeitung des Lernziels
5. Selbstkontrolle des Lernerfolges
6. Nutzung des Wissenszuwachses in der praktischen Arbeit
7. kontinuierliche Überprüfung des Wissens.

Die Bundesärztekammer bezieht sich dabei ebenfalls auf das selbstbestimmte Lernen der Ärzte und die bisherigen Erkenntnisse zum Lernen von Ärzten.

Qualität der Fortbildung

Als wichtiges Instrument zur Qualitätssicherung in der Medizin ist eine regelmäßige Überprüfung der Effektivität der angewandten Fortbildungsmethoden unerlässlich. Möglich ist dies laut Bundesärztekammer nur, wenn die Qualität von Fortbildungsmaßnahmen nach einheitlichen Kriterien bewertet wird. Dazu haben Curran et al (2005) das von Kirkpatrick (1994) entwickelte Modell für summative Evaluation modifiziert (siehe Tabelle 2).

	Level	Erklärung
**1**	**Zufriedenheit des Lernenden (Reaktion)**	Dieses Evaluationslevel liefert Daten zu den Erwartungen und Erfahrungen der Teilnehmer im Bezug auf Aufbau, Inhalt, Vermittlung der Fortbildungsthemen und Beurteilung des Experten.
**2**	**Lernergebnisse (Lernen)**	Auf diesem Level werden in irgendeiner Form das Wissen, die Fertigkeiten oder die Verhaltensweisen der Lernenden beurteilt; meist durch Tests vor und nach der Intervention.
**3**	**Verhaltensverbesserung (Verhalten)**	Dieses Level liefert Informationen dazu, inwieweit das Lernen das Verhalten und die Leistung eines Teilnehmers in der täglichen Arbeit beeinflusst und ob der Lernende die neu erlernten Fähigkeiten und Fertigkeiten in seinem Arbeitsalltag anwendet.
**4**	**Patienten-/Gesundheits-versorgung**	Dieses Evaluationslevel misst die Auswirkungen auf das Gesundheitssystem und die Gesundheit der Patienten. Diese Daten sind schwer zu erheben, da es eine Vielzahl von unkontrollierbaren Variablen gibt, auf die der Lernende nach der Intervention in der täglichen Arbeit stößt.

Tab. 2: Modifizierte Version des Kirkpatrick-Modells für summative Evaluation (Curran and Fleet 2005)

Bei der Beurteilung der Qualität spielt auch die Evaluation der Fortbildungsveranstaltung durch die Teilnehmer eine Rolle. Die Ergebnisse müssen auf Verlangen der Ärztekammer vorgelegt werden. Die Bundesärztekammer (BAEK 2007) regt außerdem an, dass auch den Lernenden die Möglichkeit gegeben werden soll, den Fortbildungserfolg, das bedeutet den Zuwachs an Wissen und Fähigkeiten, in Form einer Selbstkontrolle überprüfen und dokumentieren zu können. Um die Evaluation und Qualitätskontrolle von Fortbildungsmaßnahmen mit der Zeit selbstverständlich werden zu lassen, vergibt die Bundesärztekammer für Veranstaltungen, die Lernkontrollen beinhalten, zusätzliche Fortbildungspunkte.

1.2. Fortbildungsformate und ihre Effektivität

Geeignete Methoden zur Fortbildung sind einerseits Präsenzveranstaltungen, andererseits mediengestütztes Eigenstudium (Fachliteratur, audiovisuelle Lehr- und Lernmittel) und klinische Fortbildung (Vorlesungen, Visiten, Hospitationen, Fallvorstellungen). Als Fortbildungsmedium zum Eigenstudium gewinnen zunehmend computergestützte Lernprogramme an Bedeutung.

Das derzeit häufigste Fortbildungsformat bleiben aber immer noch Fortbildungsveranstaltungen in Form von Seminaren und Vorträgen. Laut den Empfehlungen der Bundesärztekammer (BAEK 2007) sind die besten Erfolge der Wissensvermittlung dann zu erwarten, wenn die Veranstaltungen folgenden Kriterien entsprechen:

Sie sollten

- praxisrelevant sein, d.h. gezielt die täglichen Arbeitsprobleme des Lernenden behandeln („problemorientiertes Lernen")
- die persönlichen Bedürfnisse des Lernenden berücksichtigen
- interaktiv sein („Lernen in Gruppen")
- die Erfahrungen des Lernenden in die Interaktion mit einbeziehen („fallbezogenes Lernen")
- Rückkopplung zwischen Lehrendem und Lernendem ermöglichen („Verfügbarkeit von Experten zur individuellen Beratung")
- Den Lernenden dazu befähigen, eigene Entscheidungen zu fällen („Kritische Anwendung von Expertenwissen auf die eigenen Probleme"),
- Zu weiterem Studium führen („Stärkung von Problembewusstsein und Umgang mit Informationsquellen")

Allerdings geht die Bundesärztekammer davon aus, dass diesen Ansprüchen eher Veranstaltungen in kleinen Gruppen als solche vor großem Auditorium gerecht werden. Im Gegensatz dazu geht Michaelsen (2002) davon aus, dass Lernen in Gruppen (Team-based Learning) auch in großen Gruppen funktioniert (vgl. Abschnitt Team-based Learning).

Im Folgenden sollen die verschiedenen Fortbildungsformate beschrieben und deren Effektivität bezüglich Wissenserwerb, Verhaltensänderung und Patientenversorgung

der teilnehmenden Ärzte dargestellt werden. In Tabelle 3 sind die Methoden der Dissemination definiert und ihre Wirksamkeit nach bisherigen Studien dargestellt.

**Medium**	**Definition**	**Wirksamkeit**
**Gedruckte Materialien**	Gedruckte oder elektronische Praxis-Empfehlungen, als persönliche Anschreiben oder Massensendungen	Beitrag zur Versorgung bestenfalls geringfügig (Freemantle, Harvey et al. 1997), allerdings wichtig als Teil einer komplexen Strategie
**Örtliche Multiplikatoren (local opinion leaders)**	Durch systematische Befragung von Ärzten werden einflussreiche Individuen herausgefunden, diese wiederum treten nach Schulung an ihre Kollegen vor Ort heran	Studienlage nicht eindeutig (Thomson O'Brien, Oxman et al. 2000), aber einige Positivbeispiele publiziert
**Interaktive Seminare und Kleingruppen**	Kurze Konferenzen (maximal ein Tag)	Ohne Umsetzungshilfen und Ähnliches kaum wirksam (Davis, Thomson et al. 1995; Oxman, Thomson et al. 1995)
**Frontalvortrag**		Weitgehend wirkungslos (Davis, O'Brien et al. 1999)
**Praxisbesuche (academic detailing, outreach visits)**	Entspricht dem Besuch eines Pharmareferenten	Effekte vor allem im Bereich der rationalen Medikamentenverschreibung dokumentiert
**Audit und Feedback**	Systematische Erfassung der Versorgungsqualität in einer definierten Einheit (Praxis, Klinik, Region usw.), meist fokussiert auf ein spezifisches Thema (bestimmte Krankheit oder Prozesse z. B. Verschreibung); Rückmeldung der Ergebnisse an Betroffene (Feedback).	Wirksam, wo entsprechendes Wissen und Handlungsbereitschaft vorhanden (Shea, DuMouchel et al. 1996; Thomson O'Brien, Oxman et al. 2000)
**Erinnerungshilfen (reminder)**	Idealerweise mit Software zur Praxisdokumentation gekoppelt	Computergestützte Erinnerungshilfen können die Berücksichtigung von Früherkennungsmaßnahmen fördern (Shea, DuMouchel et al. 1996)
**Massenmedien**	An Laien gerichtetes Fernsehen, Radio, Printmedien	Vermutlich wirksam zur Steuerung der Inanspruchnahme von medizinischen Leistungen (Reduzierung überholter wie auch Verbreitung neuer Leistungen) (Grilli, Freemantle et al. 2000)

Tab. 3: Methoden der Dissemination (Donner-Banzhoff 2001)

1.2.1. Präsenzveranstaltungen: Vorträge, Workshops, Seminare

Diese Fortbildungsform umfasst variable Formate: von passiven Lehrveranstaltungen für eine große Teilnehmerzahl bis zu sehr interaktiven Interventionen in Form von Workshops, Kleingruppen und individuellen Trainingseinheiten. Beispiele für diese Formate sind Visiten, Lehrveranstaltungen, Konferenzen, Auffrischungskurse, Programme, Seminare, Vorlesungen, Workshops und Symposien. Eine Mehrheit der Ärzte nimmt regelmäßig an derartigen Fortbildungsveranstaltungen teil. Nach Schätzungen bringen Ärzte dafür durchschnittlich eine bis drei Wochen im Jahr auf, damit steht diese Form der Fortbildung an zweiter Stelle nach dem Selbststudium von Lehrbüchern oder Fachzeitschriften (Rothenberg, Wolk et al. 1982; Goulet, Gagnon et al. 1998; Lecoq-D'Andre and Balblanc 1999; Frank, Baldwin et al. 2000; Nylenna and Aasland 2000). Da inzwischen in vielen Ländern eine Fortbildungspflicht für Ärzte eingeführt wurde, haben sich wirtschaftliche Interessen und eine eigene Fortbildungsindustrie entwickelt. Allerdings wird seit langem infrage gestellt, ob passive Fortbildungsmaßnahmen wie Vorlesungen wirksam das Verhalten der Ärzte und die Patientenversorgung verbessern können (Davis, Thomson et al. 1995). Einige Studien haben einen mangelnden Effekt auf die Compliance von Ärzten mit Praxisleitlinien (Grimshaw and Russell 1993; Davis and Taylor-Vaisey 1997) und große Unterschiede zwischen der realen und idealen Ausführung gezeigt (Lau, Antman et al. 1992; Bero, Grilli et al. 1998). Allerdings ziehen viele Ärzte diese Fortbildungsformate vor (Kelly and Murray 1994; Stancic, Mullen et al. 2003).

Aufgrund der weiten Verbreitung dieses Formats untersuchten Forscher in mehreren großen Metaanalysen die bisherigen randomisierten und qualitätvollen Studien zur Effektivität formaler Fortbildungsmaßnahmen, eine Verhaltensänderung der Ärzte oder eine verbesserte Patientenversorgung herbeizuführen (Davis, O'Brien et al. 1999; Thomson O'Brien, Freemantle et al. 2001). Im aktuellsten Review von O’Brien wurden 32 Studien identifiziert mit 36 Vergleichen von Interventionen. Sie schlossen keine Studien ein, die lediglich das Wissen der Teilnehmer evaluierten. Interventionen wurden als passiv definiert, wenn sie überwiegend aus einer Vorlesung oder Präsentationen mit geringen Diskussionsanteilen bestanden. Als interaktive Interventionen wurden Workshops oder Seminare bezeichnet, die eine Form von

Interaktion der Teilnehmer in verschiedenen Gruppengrößen beinhalteten. Diese Interaktion konnte aus Rollenspiel, Falldiskussionen oder der Möglichkeit, klinische Fertigkeiten zu praktizieren, bestehen.

Die Qualität der Studien wurde insgesamt als mittel bis hoch eingeschätzt. Thomson O'Brien et al (2001) kritisierten jedoch, dass die verwendeten Methoden von den Autoren generell schlecht beschrieben worden waren. In 31 Studien wurden objektive Daten zum Verhalten der Ärzte erhoben, in acht Studien Daten zur Patientenversorgung. In den sieben Vergleichen von passiven Fortbildungsveranstaltungen zeigte sich, bis auf ein Teilkriterium in einer einzelnen Studie, kein wesentlicher Effekt auf das Verhalten und die Patientenversorgung der Ärzte. Im Gegensatz dazu fanden die Wissenschaftler bei sechs von zehn Vergleichen von interaktiven Fortbildungsmaßnahmen einen mittelmäßigen bis mittelhohen, statistisch signifikanten Effekt und in vier einen geringen Effekt, von denen einer ebenfalls signifikant war. Interventionen, die passive und aktive Fortbildungsformen kombinierten, zeigten in 12 Vergleichen einen mittelmäßigen bis mittelhohen Effekt (elf davon signifikant) und einen geringen in sechs Vergleichen (einen davon signifikant).

Trotz aller Einschränkungen der Interpretation der bisherigen Studienlage (kleine Studiengröße und –probanden, freiwillige Teilnahme etc.) kommen die Autoren zu dem Schluss, dass ein Effekt von passiven Fortbildungsveranstaltungen auf das Verhalten und die Patientenversorgung unwahrscheinlich ist, interaktive Workshops diesen jedoch bewirken können. Thomson O'Brien et al (2001) betonen aus den Erkenntnissen der Reviews, dass die zukünftige Forschung sich auf die Charakteristika der interaktiven Fortbildungsmaßnahmen konzentrieren und nicht weiter passive Fortbildungsmaßnahmen evaluieren sollte, da deren Ineffektivität bereits gezeigt wurde. Es sei wichtig herauszufinden, welche Qualitätsmerkmale interaktiver Maßnahmen deren Effektivität beeinflussen und steigern. Bislang fehlten beispielsweise wissenschaftliche Daten, welche Gruppengröße sich besonders eignet oder wie lange eine Intervention dauern sollte.

1.2.2. Selbststudium: Fachzeitschriften, E-Learning

Die Fortbildungsangebote zum Selbststudium reichen von Onlineangeboten bis zu regelmäßigen CME-Einheiten in medizinischen Fachzeitschriften. Als Erfolgskontrolle der Teilnahme an den Fortbildungsangeboten hat sich das Multiple-Choice-(MC)-Fragenformat etabliert. Immer mehr Ärzte nützen die Fortbildungsangebote der medizinischen Fachzeitschriften und die zahlreichen Onlineangebote. Der Vorteil dieser Fortbildungseinheiten ist insbesondere, dass sie flexibel und zu jeder Zeit und an jedem Ort bearbeitet werden können. Außerdem werden 30 bzw. 50 Punkte für Selbststudium ohne Nachweis von den deutschen Landesärztekammern anerkannt. Dabei wird vorausgesetzt, dass Ärzte sich selbstständig fortbilden, indem sie Fachliteratur zu Rate ziehen oder im Internet recherchieren, wenn sie auf konkrete klinische Fragestellungen stoßen.

Im folgenden Abschnitt wird auf die Charakteristika dieser Fortbildungsformate und den bisherigen Kenntnisstand in der wissenschaftlichen Literatur eingegangen.

Fortbildungsangebote von Fachzeitschriften und gedruckte Fortbildungsmaterialien:

Sowohl im deutschsprachigen Raum als auch international bieten zahlreiche medizinische Fachzeitschriften regelmäßig eigene CME-Fortbildungen mit Multiple Choice-Tests an. Andere gedruckte Fortbildungsmaterialien bestehen aus Monographien oder Leitlinien, die auf verschiedene Arten verbreitet und vertrieben werden (beispielsweise von Fachgesellschaften). Die Fortbildungseinheiten der Fachzeitschriften bestehen meist aus einem oder mehreren Artikeln zu einem speziellen Thema, an die sich Multiple Choice-Fragen (MC-Fragen) zur Wissenskontrolle anschließen. An diesen CME-Angeboten können die Abonnenten der Zeitschriften kostenlos teilnehmen, teilweise sind spezielle Angebote für alle Interessierten kostenfrei verfügbar. Der Teilnehmer kann in der Printausgabe der Zeitschrift oder im Internet nach erfolgreichem Login die Themenartikel lesen und anschließend online die MC-Fragen zur Erfolgskontrolle beantworten. Außerdem bieten die meisten Fachzeitschriften die Möglichkeit an, die Fragen auf einem Vordruck zu beantworten und anschließend per Fax oder per Post zu schicken. Einige

Zeitschriften sind inzwischen dazu übergegangen, die Erfolgskontrolle nur noch online anzubieten. Sie haben eigene Fortbildungsportale mit einem individuellen CME-Konto der Benutzer. Jeder angemeldete Benutzer kann so jederzeit seine bisher gesammelten Punkte, die bereits bearbeiteten und die insgesamt angebotenen Fortbildungen einsehen und seine Fortbildungszertifikate sammeln und ausdrucken. Ein bereits abgeschlossenes Fortbildungsangebot kann zwar im Nachhinein noch aufgerufen, jedoch nicht nochmals bearbeitet werden. In Deutschland sind zehn MC-Fragen als Erfolgskontrolle vorgeschrieben und werden einheitlich im „Single-Best-Answer"-Format gestellt, bei dem immer nur eine aus fünf Antwortmöglichkeiten zutrifft. Wenn ein Teilnehmer mindestens sieben der zehn Fragen richtig beantwortet, erhält er zwei bis drei CME-Punkte und ein Zertifikat über die erfolgreiche Teilnahme an der Fortbildung.

Theoretisch könnten durch regelmäßige Teilnahme an den CME-Beiträgen von nur einer Zeitschrift (Beispiel Deutsches Ärzteblatt) bis zu 82% der insgesamt vorgeschriebenen Fortbildungspunkte erworben werden. Dass ein großer Anteil von Ärztinnen und Ärzten diese Möglichkeit wahrnimmt, bestätigen die hohen Teilnehmerzahlen an den Fortbildungen (durchschnittliche Teilnehmerzahlen pro CME-Fortbildung: Deutsches Ärzteblatt ~22.000, Der Internist ~ 2.000). Nach Angaben des Accreditation Council for Continuing Medical Education (ACCME 2006) nahmen 2006 in den USA rund 630.000 Ärzte an Fortbildungsangeboten von Fachzeitschriften teil, 1998 waren es nur rund 210.000 (Annual report 1998 und 2006, www.accme.org).

Mit der zunehmenden Teilnahme der Ärzte an CME-Angeboten von medizinischen Fachzeitschriften stellt sich die Frage nach deren Qualität und Effektivität. In einem Übersichtsartikel der Cochrane-Gesellschaft zu diesem Thema zeigten Freemantle et al (1997), dass die passive Dissemination von Information im Vergleich zu keiner Interaktion sehr geringe Effekte aufweist. Andere Studien fanden jedoch heraus, dass die passive Verbreitung von nationalen Leitlinien einen positiven Effekt auf die klinische Tätigkeit hat, wenn der Kontext für eine Veränderung zuträglich war (Mann, Beziat et al. 2001; Black and Hutchings 2002). Aus der Tatsache, dass gedruckte Materialien eine gewohnte, leicht zu nutzende, kostengünstige Fortbildungsform sind,

schloss Freemantle et al (1997), dass sie möglicherweise eine effektive Maßnahme zur Verhaltensänderung darstellen. Wichtige Faktoren sind dabei die Informationsquelle, der Inhalt und die Verbreitungsart. Die Informationen sollten einer glaubwürdigen Quelle entstammen, klar und verständlich geschrieben und auf Fachwissen begründet sein. Außerdem kann der Inhalt auf individuelle Bedürfnisse zugeschnitten werden. Dazu haben mehrere Studien positive Effekte gezeigt (Kreuter and Strecher 1996; Bull, Holt et al. 2001; Kreuter 2001), allerdings gibt es wenige Studien, die diese verschiedenen Inhaltsstile direkt verglichen haben.

Bisher gibt es wenige wissenschaftliche Erkenntnisse dazu, wie gedruckte Fortbildungsmaterialen die klinische Tätigkeit beeinflussen können (Farmer 2003). Einige Studien lassen die Annahme zu, dass Leitlinien umso eher beachtet werden, je mehr sie mit bestehenden Normen und Werten vereinbar sind, je leichter sie zu befolgen sind, je mehr sie evidenzbasiert sind und je weniger sie die Aneignung neuen Wissens und neuer Fähigkeiten erfordern (Grilli and Lomas 1994; Grol, Dalhuijsen et al. 1998; Burgers, Grol et al. 2003). Allerdings berichteten Foy et al (2002), dass Richtlinien, die nicht mit bestehenden klinischen Werten vereinbar sind, mit einer größeren Verhaltensänderung assoziiert waren. Die Forscher fanden dies allerdings bei einer Intervention mit Audit and Feedback heraus, nicht mit gedruckten Fortbildungsmaterialen. Farmer et al (2003) schließen daraus, dass die Verhaltensänderung umso geringer ausfällt, je besser die Leitlinien mit den bestehenden Normen vereinbar sind. Dafür machen sie einen Ceiling-Effekt[1] verantwortlich. Die graphische Darstellung von gedruckten Fortbildungsbeiträgen scheint nach einer Studie von Avorn und Soumerai (1983) keinen Einfluss auf das Verschreibungsverhalten zu haben.

Zahlreichen Studien zufolge schätzen die Ärzte die Fortbildungsangebote in Zeitschriften sehr (Cole and Glass 2004; Tredwin, Eder et al. 2005; Scolapio, Deluise et al. 2007) und geben an, dass diese Angebote ihr Wissen steigern und ihr Verhalten

---

[1] Ceiling-Effekt: Auch Deckeneffekt oder Dacheffekt genannt. Hierunter versteht man ein Phänomen in der Testpsychologie, z. B. ein Leistungstest der an einer gewissen oberen Grenze die Ergebnisse der Testpersonen nicht mehr zuverlässig messen kann. Wenn Personen mit einer höheren Testleistung diesen Test begehen, dann ist er „zu leicht", die Personen können dann alle oder fast alle Aufgabe des Tests lösen. Nun würde dieser Test den Testpersonen einen Testwert zuordnen, der deren tatsächliches Leistungsvermögen nicht mehr adäquat widerspiegelt.

in der Praxis ändern. Cole et al (1998) bezeichnen aus diesen Gründen die Fortbildung durch medizinische Fachzeitschriften als wertvolle und relativ kostengünstige Möglichkeit, Fortbildungspunkte zu erwerben.

E-Learning:

Mit der Einführung und Verbreitung des Internets ist auch seine Bedeutung als Informationsquelle für Ärzte stark gestiegen. Dazu trägt die wachsende Zahl an wissenschaftlichen Veröffentlichungen in den letzten Jahrzehnten bei, die Ärzte dazu zwingt, sich laufend über neue Erkenntnisse zu informieren und regelmäßig wissenschaftliche Publikationen zu lesen, um auf dem aktuellen Wissensstand zu bleiben. Laut Studien nützten im Jahr 2003 etwa 88% der kanadischen Ärzte and 78% der amerikanischen Ärzte das Internet, mehr als zwei Drittel davon als Informationsquelle für praktische klinische Problemstellungen (Bennett, Casebeer et al. 2004; Martin 2004). Weniger Ärzte nützten dagegen Internet-basierte CME-Angebote: 23% in Kanada (2001) und 46% in Amerika (2003). Nach Wutoh et al (2004) nützten 2001 nur 2,7% der Ärzte das Internet für CME-Aktivitäten, zum Zeitpunkt der Veröffentlichung ihrer Studie 2004 dagegen schon 31%. CME war sogar der zweithäufigste Grund für die Internetnutzung und jüngere Ärztinnen die größte Nutzergruppe. Zu letzterem Ergebnis kommen auch Harris et al (2003). Sie berichten, dass junge Frauen Männer bei der Nutzung von Online-CME-Kursen überholt haben. Im Gegensatz dazu konnten Bernhardt et al (2003) keine Korrelation zwischen Alter, Geschlecht, ethnischer Zugehörigkeit und den Einschätzungen und Erwartungen von Medizinern zu Internet-basierten Fortbildungsangeboten feststellen.

Der häufigste Beweggrund zur Informationssuche im Internet ist eine spezielle Fragestellung bei einem Patienten (Curran and Fleet 2005). Casebeer et al (2002) fanden heraus, dass die Ärzte meist aufgrund des Bedürfnisses handelten, ihre Patienten so gut wie möglich zu versorgen. Die Möglichkeit, in kürzester Zeit eine große Informationsmenge zu filtern, macht die Suche im Internet zu einer einzigartigen Methode selbstbestimmten Lernens (Self-directed Learning). Das Internet eröffnet nach Curran et al (2005) eine neue Dimension zur vereinfachten

Nutzung von CME-Aktivitäten. Es ermöglicht den Ärzten, ihre Lernziele eigenständig und unabhängig von Zeit und Ort zu verfolgen.

In den letzten Jahren ist die Zahl der Online-CME-Angebote kontinuierlich gestiegen. Einer Studie von Sklar et al (2001) zufolge nahm die Anzahl von CME-Anbietern im Internet von 96 Internetseiten im Jahr 2000 innerhalb eines Jahres auf 200 und bis Anfang 2002 auf 17.000 Angebote zu. Nach Angaben der ACCME nahmen 1998 etwa 36.000 Ärzte an internetbasierten Fortbildungen teil, 2006 waren es bereits circa 2.180.000 (Annual report 1998, 2006, www.accme.org). Die Formate der CME-Aktivitäten variieren sehr. Fortbildungspunkte können sowohl mit Lesen, Hören oder Anschauen von Beiträgen erworben werden oder auch mit der Teilnahme an interaktiven Wissenstests. Mit der steigenden Nutzung und Verbreitung der Online-Angebote kam auch die Forderung von Experten nach Qualitätsstandards für die ärztliche Fortbildung im Internet auf. Manning und Debakey (2001) schlagen vor, dass web-basierte CME-Angebote selbstbestimmtes Lernen unterstützen sollten, indem sie es den Teilnehmern ermöglichen, den Inhalt, die Lernmethode und die Lernressourcen selbst auszuwählen. Sie glauben, dass Internet-basierte CME größere Möglichkeiten für qualitätvolle Fortbildung bieten kann, wenn sie diese Grundvoraussetzungen erfüllt.

Lange Zeit wurde die Effektivität von Online-CME-Angeboten bezweifelt. Inzwischen gibt es aber zahlreiche wissenschaftliche Studien, die deren Effektivität auch im Vergleich zu traditionellen Fortbildungsaktivitäten untersucht haben (Wutoh, Boren et al. 2004). Fordis et al (2005) verglichen dazu beispielsweise einen interaktiven Workshop mit Kleingruppenarbeit mit einer internetbasierten Fortbildung, um herauszufinden, ob die letztere CME-Aktivität ähnliche Resultate im Bezug auf den Wissenserwerb und Verhaltensänderung bei den Ärzten zeigte. Beide Gruppen zeigten sowohl den gleichen signifikanten Wissenszuwachs als auch ein verbessertes leitliniengerechtes Verhalten beim Screening auf Hypercholesterinämie. Im Bezug auf die Pharmakotherapie von Hochrisikopatienten schnitten die Teilnehmer der webbasierten Intervention dagegen besser ab als die Vergleichsgruppe, deren Verhalten keine signifikante Veränderung zeigte. Die Autoren der Studie erklären dieses Ergebnis mit der Teilnahme an einer weiteren Lehrveranstaltung zu einem

späteren Zeitpunkt, kombiniert mit der Bearbeitung der Fortbildungsinhalte in mehreren Sitzungen.

Inzwischen gibt es zahlreiche Metaanalysen zur Effektivität von CME-Aktivitäten im Internet. Curran et al (2005) untersuchten die Ergebnisse von webbasierten CME-Evaluations-Studien in der Peer-review-Literatur. Dazu kategorisierten sie die Studien nach dem Kirkpatrick- Modell ((1994)¸ siehe Tab. 2). Insgesamt untersuchten Curran et al 86 Studien, von denen die Mehrzahl (55) deskriptiv waren. Diese Studien beinhalteten meist keine systematische Evaluation der Effektivität der Maßnahmen. In 31 Studien wurde eine Evaluationsstufe benutzt, die nach Kirkpatrick kategorisiert werden konnte. 80,6% der Studien führten eine Analyse auf dem Level 1, 51,6% auf dem Level 2 und 6,5% auf dem Level 3 durch. Keine Studie berichtete über eine Evaluation der Patienten- oder Gesundheitsversorgung (Level 4). Die Ergebnisse der Studien sind in Tabelle 4 dargestellt.

	**Level**	
**1**	**Zufriedenheit des Lernenden (Reaktion)**	Generell waren die Ärzte mit webbasierten Fortbildungen zufrieden. Als Vorteile wurden genannt: einfache, ortsunabhängige, leicht zugängliche und zeitnahe Verfügbarkeit; Informationen auf dem neuesten Stand, selbstbestimmte Nutzung des Inhalts, geringer Aufwand, Multimedia-Format, interaktive klinische Fälle.<br>Nachteile oder abschreckende Faktoren waren: mangelnde Computerkenntnisse, Gefühl der Informationsüberflutung, fehlende soziale Kontakte, etc.
**2**	**Lernergebnisse (Lernen)**	Die Mehrheit der Studien zeigte, dass webbasierte CME-Angebote zu Wissenserwerb und einer veränderten Einstellung sowie zu einem geringeren Teil auch zu verbesserten Fähigkeiten bei den Ärzten führten. Die meisten Untersuchungen stützen sich auf Vor- und Nachtests, wenige schlossen eine Kontrollgruppe ein.
**3**	**Verhaltens-verbesserung (Verhalten)**	Die zwei einzigen Studien dazu zeigten, dass die Teilnahme an webbasierten Fortbildungen zu signifikanten Verhaltensänderungen führte. Die Ergebnisse beider Untersuchungen basierten aber auf Selbsteinschätzungen und –auskünften der Ärzte.
**4**	**Patienten-/ Gesundheits-versorgung**	Keine Studien vorhanden.

Tab. 4: Ergebnisse von Curran et al (2005)

Auch Wutoh et al (2004) führten ein Literaturreview zu diesem Thema durch. Sie untersuchten in ihrer Studie, ob (1) internetbasierte Fortbildungen effektiv sind, (2) effektiver als traditionelle CME-Maßnahmen sind und (3) was effektive webbasierte Fortbildungsformate auszeichnet, die einen positiven Effekt auf das Verhalten der Ärzte und die Patientenversorgung haben. Sie schlossen nur randomisierte Kontrollstudien, Metaanalysen, retrospektive Studien, Studien mit freiwilliger Teilnahme ein, wenn sie sich mit ärztlicher Aus- und Fortbildung beschäftigten. Sie identifizierten insgesamt 86 Studien, von denen sie 16 einschließen konnten. 15 davon evaluierten das Wissen der Teilnehmer nach objektiven Bewertungsmaßstäben vor und nach der Intervention, zehn Studien zusätzlich nach subjektiven Kriterien. Nur eine Studie erhob jedoch das Wissen und Verhalten im Langzeitverlauf nach vier bis sechs Monaten, zeigte aber keine anhaltende Wirkung der Intervention. In drei Studien wurden die Ärzte zu einer subjektiven Selbsteinschätzung gebeten, ob sie ihr Verhalten nach der webbasierten Fortbildung ändern würden. Marshall et al (2001) berichteten, dass 64,7% der Teilnehmer einer Online-Falldiskussion angaben, dass sie nach der webbasierten Fortbildung ihr Verhalten geänderten hätten, im Gegensatz zu 30,8% in der Vergleichsgruppe. Als Fortbildungsformate nutzten drei Studien E-mails oder Newsgroups zur Diskussion, die restlichen 13 Studien webbasierte Strategien. Diese umfassten allerdings weitestgehend nur Maßnahmen zum Literaturstudium, Newsgroups für Diskussionen, CD-Roms oder individuelle Wissenstests. Von sechs Studien, die eine webbasierte Methode mit gedruckten Materialien verglichen, konnte die Hälfte zeigen, dass die Teilnehmer der Internet-Fortbildung besser im Nachwissenstest abschnitten als die Vergleichsgruppe, die nur gedruckte Materialien zum Selbststudium bekam. Eine Studie fand heraus, dass die Teilnehmer der Online-Fortbildung mehr Zeit aufwendeten, eine andere Untersuchung fand keinen Unterschied im Bezug auf den zeitlichen Rahmen. Die Online-Fortbildungen wurden generell besser von den Teilnehmern bewertet als die bloße Verwendung gedruckter Fortbildungsmaterialien. Viele Teilnehmer bevorzugten Onlineformate, die Multimedia-Unterstützung anboten. Sechs Studien verglichen außerdem webbasierte mit traditionellen Fortbildungsformaten wie Vorträgen. In allen Untersuchungen kam heraus, dass beide Formate gleich effektiv im Bezug auf das Ergebnis der Abschlussprüfungen waren. Eine Studie wies einen generellen Vorteil eines

webbasierten Lernprogramms gegenüber einem formalen Vortrag nach (Goldberg and McKhann 2000).

Wutoh et al (2004) ziehen den Schluss, dass internetbasierte CME-Programme genauso effektiv im Bezug auf die Wissensvermittlung sind wie ausgewählte andere Fortbildungsprogramme. Außerdem schreiben die Autoren, dass immer noch zu wenig darüber bekannt sei, ob positive Veränderungen im Wissensbereich auch zu einer Veränderung im Verhalten führen. Sie fordern zusätzliche Studien, die untersuchen, wie lange neu erlerntes Wissen und Verhaltensweisen beibehalten werden. Dennoch zeigen diese und weitere Studien zum Thema den Wert und das Potenzial von internetbasierten Strategien zur Wissensvermittlung sowohl im Studentenunterricht als auch in der Fort- und Weiterbildung.

1.2.3. Klinische Fortbildung: Audit and Feedback

Kommunale Strategien wie Praxisbesuche („academic detailing“, „audit and Feedback“, „educational outreach visits“), örtliche Multiplikatoren („opinion leaders“), anwenderbezogene Methoden wie Erinnerungshilfen („reminders“) und patientenverbundene Strategien („patient-mediated strategies“) und multiple Interventionen scheinen effektive Methoden der Fortbildung zu sein (siehe Tabelle 3: Methoden der Dissemination nach Donner-Banzhoff (2001)).

Kommunale Strategien: Academic Detailing, educational outreach visits und Audit and feedback.

Diese Disseminationsformen beruhen auf der systematischen Erfassung ärztlichen Versorgungsverhaltens (performance). Um das Verhalten in realen Versorgungssituationen zu evaluieren, kann z.B. mit Hilfe der Praxisdokumentation das Verhalten von Ärzten bei bestimmten Problemen analysiert und in Bezug auf einen zuvor definierten Standard bewertet werden (so genanntes Audit). Dies bildet dann den Ausgangspunkt für Verbesserungen. Ein Beispiel wäre die wissenschaftliche Untersuchung von Medikamentenverschreibungen in einem bestimmten Zeitraum anhand der Praxisdokumentation nach definierten Kriterien auf Angemessenheit.

Detailliertes Feedback an die evaluierten Ärzte ist ein wesentlicher Bestandteil solcher Interventionen.

Soumerai und Avorn (1990) beschrieben die folgenden acht wichtigen Prinzipien dieser Disseminationsformen, um die klinische Entscheidungsfindung von Ärzten zu verbessern:

1. Erhebung des Vorwissens und der Motivationen für die klinische Entscheidungsfindung
2. Fokussierung der Fortbildung auf spezifische Ärztegruppen
3. Definition von klaren Lernzielen und Verhaltensänderungen
4. Herstellung von Glaubwürdigkeit (Fortbildungsanbieter, Informationsquellen)
5. Stimulation zur aktiven Teilnahme an den Fortbildungsangeboten
6. Nutzen von klaren graphischen Lehrmaterialien
7. Hervorheben und Wiederholung der essentiellen Botschaften
8. Anbieten von positiver Verstärkung durch follow-up-Besuche

Jamtvedt et al (2006) untersuchten in einem Literaturreview der Cochrane-Gesellschaft alle bisherigen randomisierten Studien zu den Effekten von Audit and Feedback auf das Wissen und Verhalten der Ärzte sowie auf die Patientenversorgung. Im aktuellsten Review identifizierten die Forscher 118 Studien, die entweder Audit and Feedback alleine oder in Verbindung mit anderen Disseminationsmethoden mit keiner Intervention oder einer anderen Intervention verglichen hatten. Sie untersuchten nur Studien, die objektive Daten zur Verhaltensänderung oder Patientenversorgung beschrieben. Ein weiteres Ziel des Reviews war herauszufinden, welche Faktoren die Effektivität von Audit and Feedback beeinflussen. Jamtvedt et al kommen zu dem Schluss, das Audit and Feedback das Verhalten der Ärzte wirksam verbessern können. Allerdings scheinen die Effekte variabel und generell eher gering bis mäßig zu sein. Die relative Wirksamkeit scheint jedoch höher zu sein, wenn das Verhalten vor der Intervention nur in geringem Maße den Zielrichtlinien entsprach. Youl et al (2007) fanden heraus, dass gerade unerfahrene Ärzte am meisten von Audit and Feedback-Interventionen profitierten. Das gleiche Phänomen zeigte sich bei einer Studie von Palmer et al (1985) in Bezug auf die Motivation der Ärzte, ihr Verhalten zu ändern. Es zeigte sich interessanterweise, dass gerade Aufgaben, die mit einer niedrigen

Motivation der Ärzte verbunden waren eine stärker ausgeprägte positive Veränderung eintrat. Dies erklärten die Autoren einerseits damit, dass diese Aufgaben eher administrativer Natur waren. Andererseits scheint aber auch Feedback besonders dann sehr wichtig zu sein, wenn nur eine niedrige Motivation zur Veränderung vorhanden ist. Zu diesem Ergebnis kamen auch andere Studien (Sibley, Sackett et al. 1982; Foy, MacLennan et al. 2002), die herausfanden, dass sich die Qualität der Versorgung nur dann verbesserte, wenn die Themen von geringerem Interesse waren. Jamtvedt et al (2006) ziehen in Betracht, dass dieses Phänomen für die variablen Effekte von Audit and Feedback verantwortlich sein könnte, ohne es aber wissenschaftlich beweisen zu können. Insgesamt verglichen nur sieben der 118 Studien Audit and Feedback mit einer anderen Intervention. Zwei Vergleiche bezogen sich auf Erinnerungshilfen (reminder) als Vergleichsintervention und zeigten keinen Vorteil einer Intervention. Die Autoren ziehen den Schluss, dass ihre Ergebnisse nicht gezeigt haben, dass Interventionen mit Audit and Feedback generell die Performance der Ärzte verbessern können, unabhängig von der Art und Weise der Darbietung.

Hoskins et al (1997) werfen die Frage auf, ob die Effekte von Audit and Feedback nur auf der allgemeinen Motivation der Ärzte beruhen, die an diesen Interventionen und Studien teilnehmen. Audit könnte eher der Katalysator als der eigentliche Grund für die Veränderung sein. Diese Annahme stützen auch die Ergebnisse von Allen et al (2007), die die Ansichten von Allgemeinärzten in Bezug auf Audit and Feedback bzw. Academic Detailing untersuchten. Dabei fanden sie heraus, dass die Nutzer dieser Interventionen sie als sehr wertvolle Lernmethoden bewerteten und Informationen aus anderen Quellen kritischer betrachteten als Ärzte, die diese Fortbildungsmethode nicht nutzten. Für diese Ärzte bestand ein wichtiger Grund, diese Angebote nicht zu nutzen, darin, dass sie dafür Arbeitszeit aufwenden müssten. Allen et al schlossen deshalb, dass solche Ärzte durch derartige Interventionen gar nicht erreicht werden können.

Ein weiterer wichtiger Faktor scheint die Art des Feedbacks zu sein. Jamtvedt et al (2006) gehen davon aus, dass die Effekte von Audit und Feedback größer sein könnten, wenn die Ärzte aktiver in den Feedback-Prozess mit einbezogen würden und nicht nur passive Empfänger des Feedbacks sind. Die Ergebnisse mehrerer Studien (Holmboe, Scranton et al. 1998; Hadjianastassiou, Karadaglis et al. 2001;

Shakespeare, Mukherjee et al. 2005; Hysong, Best et al. 2006) weisen darauf hin, dass ein individuelles, vertrauliches Feedback größere Effekte hat als allgemeines Feedback beispielsweise für eine Abteilung oder ein Krankenhaus. Auf diese Weise wird es von den Ärzten selbst eher akzeptiert und als wertvolle Hilfe angesehen (Rowan, Hogg et al. 2006).

Jamtvedt et al (2006) betonen die Kosteneffizienz solcher Interventionen. Sie sind der Ansicht, dass sich auch kleine Effekte, die durch Fortbildungsmaßnahmen mit Audit and Feedback ausgelöst werden, lohnen, wenn der Aufwand im Vergleich dazu gering ausfällt.

Örtliche Multiplikatoren (Local opinion leaders):

Als örtliche Multiplikatoren werden Mediziner definiert, die von ihren Kollegen als pädagogisch einflussreich eingeschätzt werden (Hiss 1978). Es wird davon ausgegangen, dass örtliche Multiplikatoren das Verhalten ihrer Kollegen positiv beeinflussen können, indem sie neue Normen verbreiten und das geeignete Verhalten vormachen. Diese Annahmen beruhen einerseits auf der Theorie der Diffusion von Innovationen und auf dem Modell der sozialen Beeinflussung von Verhalten (Soumerai, McLaughlin et al. 1989; Lomas 1991). Mittman et al (1992) geht davon aus, dass die Einstellung von Gleichgestellten (peers) die Wahrnehmung und Annahme neuer Informationen und Verhaltensweisen bedeutend beeinflusst. Greer et al (1988) weist darauf hin, dass örtliche Multiplikatoren eine Art sanktionierende Funktion ausüben können. Außerdem könnten sie die Adaptierung von Richtlinien an die lokalen Umstände verbessern.

Allerdings ist bislang unklar, auf welche Weise die örtlichen Multiplikatoren ihre Kollegen beeinflussen. Möglichkeiten sind beispielsweise ihre Glaubwürdigkeit und ihr Status als Gemeinschaftsmitglied sowie ihre Kenntnis der lokalen Gegebenheiten wie Ressourcen und Hindernisse. Außerdem merken Thomson O'Brien et al (2000) an, dass diese Personen häufig an Fortbildungsmaßnahmen teilnehmen oder sie organisieren und deshalb die beeinflussenden Faktoren schwierig auseinander zu halten sind. Die Forscher untersuchten in einem Literaturreview die Effekte der örtlichen Multiplikatoren auf das Verhalten und die Patientenversorgung von Ärzten.

Sie identifizierten acht randomisierte Studien, die insgesamt 296 Mediziner einschlossen und objektive Parameter des Verhaltens oder der Gesundheitsversorgung erfassten. Sie kamen zu einem gemischten Ergebnis. Sechs Studien zeigten in wenigstens einem Kriterium eine Verhaltensverbesserung; in zwei Studien zeigte sich eine signifikante und klinisch bedeutsame Veränderung des Verhaltens. Von drei Studien, die Daten der Patientenversorgung erhoben, zeigte nur eine einen klinisch relevanten Effekt. O'Brien et al weisen daraufhin, dass bislang sehr wenig qualitätvolle wissenschaftliche Daten vorliegen und mehrere Faktoren die Beurteilung der bisherigen Datenlage erschweren. Einerseits ist es die Auswahl und Definition der örtlichen Multiplikatoren und deren genaue Rolle, andererseits auch die Intensität einer Intervention. Sie kommen zu dem Schluss, dass örtliche Multiplikatoren eine wichtige Rolle bei der Beeinflussung des Verhaltens spielen können. Bislang sei es jedoch aufgrund der geringen Datenlage nicht möglich, die Art und Weise einer solchen Intervention zu bestimmen. Sie fordern deshalb, in weiteren Studien zu untersuchen, in welchen Situationen örtliche Multiplikatoren zur Dissemination genützt werden sollten, wie sie agieren sollen und wer sich genau als Multiplikator eignet.

Erinnerungshilfen (reminders):

Erinnerungshilfen können aus gedruckten Informationsmaterialien, die in Patientenakten gelegt werden, oder aus computergestützten Programmen bestehen, die in die Software der Praxis oder der Klinik integriert sind. Sie können sowohl bei Ärzten als auch bei Patienten eingesetzt werden. Sie werden vor allem in der Betreuung chronisch kranker Patienten, bei der Durchführung von Früherkennungsmaßnahmen und als Warnhinweise bei der Verschreibung von Medikamenten verwendet. So kann beispielsweise an regelmäßige Nachsorgeuntersuchungen erinnert werden oder vor gefährlichen Arzneimittelwechselwirkungen oder Kontraindikationen bei der geplanten Verschreibung eines bestimmten Medikaments gewarnt werden. Auch eine bessere Implementierung von Leitlinien ist auf diesem Weg versucht worden, allerdings mit unterschiedlichen Ergebnissen. Echlin et al (2004) untersuchten beispielsweise in einer

Studie die Leitlinien-Kenntnisse von Assistenzärzten zur Prävention, Diagnostik und Therapie von arterieller Hypertonie und Hypercholesterinämie, nachdem diese Leitlinien jeder geeigneten Patientenakte beigefügt wurden. Die Kenntnisse vor und nach dieser dreimonatigen Intervention nahmen nicht signifikant zu. In einer anderen randomisierten Studie untersuchten Nilasena et al (1995), ob eine Intervention mit computerbasierten Erinnerungshilfen mit patientenbezogenen Informationen zur leitliniengerechten Diabetestherapie im Vergleich zu nicht patientenbezogenen Informationen eine Verhaltensänderung der Ärzte bewirkte. Sie fanden eine signifikante Verhaltensverbesserung in beiden Gruppen, jedoch keine signifikanten Unterschiede. Allerdings stellten Nilasena et al fest, dass Ärzte, die zusätzlich bei Patientenbesuchen ein Formular am Computer ausfüllten, eine wesentlich höhere Compliance in Bezug auf die Leitlinien zeigten als Ärzte, die dies unterließen. Die Autoren schließen daraus, dass computerbasierte Erinnerungshilfen das Verhalten wirksam verbessern können, dies jedoch eher durch eine erleichterte Dokumentation oder Anmeldung von Prozeduren als durch patientenspezifische Informationen erreicht werden kann.

Auch in zwei weiteren Studien zeigte sich ein positiver Effekt auf das Verhalten der Ärzte in Bezug auf Krebsvorsorgeuntersuchungen (Ornstein, Garr et al. 1991; Litzelman, Dittus et al. 1993). In den Studien von Ornstein et al (1991) und Burack et al (1998) wurden nicht nur Erinnerungshilfen für Ärzte sondern auch für Patienten untersucht. Sie kamen zu gegensätzlichen Ergebnissen. Ornstein et al konnten am Beispiel von Präventionsmaßnahmen zeigen, dass beide Strategien die Compliance der Ärzte erfolgreich steigerten; insbesondere aber die Kombination aus Patienten- und Arzt-Remindern war wirksam. In der Arbeit von Burack et al zeigte sich nur ein sehr geringer, nicht signifikanter Effekt auf das Verhalten in Bezug auf Vorsorgeuntersuchungen und auch nur für eine Gruppe an Patienten. Außerdem fanden die Forscher heraus, dass sogar ein unvorhergesehener, unerwünschter Effekt auftreten kann. Deshalb betonen sie, dass effektivere Strategien entwickelt werden müssen und die Wirkungen besonders bei präventivmedizinischen Maßnahmen genau kontrolliert werden müssen.

In einer randomisierten Multicenter-Studie untersuchten Demakis et al (2000), ob ein elektronisches Erinnerungssystem die Compliance in Bezug auf Standards der ambulanten Versorgung verbessert. Dabei stellten sie einen signifikanten Unterschied zwischen den Ärzten, die Erinnerungshilfen bekommen hatten, und der Kontrollgruppe fest, die keine derartige Intervention bekommen hatte. Allerdings verringerte sich der positive Effekt mit der Zeit wieder. Die Autoren forderten deshalb, im Rahmen von zukünftigen Forschungsprojekten Methoden zu entwickeln, die den Effekt von Erinnerungshilfen dauerhafter machen.

In einer Metaanalyse der Cochrane-Gesellschaft untersuchten Fahey et al (2006) die Effektivität verschiedener Fortbildungsmaßnahmen auf die Blutdruckeinstellung und Betreuung von Patienten mit arterieller Hypertonie. Sie konnten zeigen, dass Reminder die Anzahl der Patienten, die zu Nachsorgeuntersuchungen kamen, erhöhten, einen wesentlichen Effekt auf die Blutdruckeinstellung fanden die Autoren nicht. In einer Studie mit Erinnerungshilfen als Warnhinweise bei der Verschreibung von bestimmten Medikamenten fanden Judge et al (2006) heraus, dass die Ärzte relativ selten auf diese Hinweise reagierten, obwohl die Anzahl an Warnhinweisen auf einen hohen Bedarf schließen ließ. Sie forderten deshalb, diese Art der Intervention zur Verhaltensänderung zu bessern und ihren Effekt auf Verschreibungsfehler und Nebenwirkungen von Arzneimitteln genauer zu untersuchen.

1.3. Lehr- und Lernstrategien für effektive Fortbildungen

Im nächsten Abschnitt soll auf die theoretischen Grundlagen von drei wichtigen Konzepten aus der Lern- und Lehrforschung eingegangen werden, auf denen das hier beschriebene und evaluierte Fortbildungsformat beruht: Team-based Learning, Problem-based Learning und Evidence-based Medicine.

1.3.1. Team-based Learning

“Team-based learning, an instructional strategy that is based on procedures for developing high performance learning teams that can dramatically enhance the quality of student learning – in almost any course.”

Die Idee des Team-based Learning geht zurück auf Larry Michaelsens (2002) Arbeiten in den späten 70er Jahren des 20. Jahrhunderts. Als Mitglied der Fakultät der Universität von Oklahoma wurde Michaelsen mit einer Vielzahl neuer pädagogischer Probleme konfrontiert. So verdreifachte sich beispielsweise die Gruppengröße seiner Klassen von 40 auf 120 Studenten. Bereits in früheren Kursen hatte er Gruppenaktivitäten angewandt und dabei festgestellt, dass so Studenten nicht nur sehr effektiv neues Wissen vermittelt, sondern auch beigebracht werden konnte, wie sie das erlernte Wissen anwenden können. Auf der Grundlage seiner Erfahrungen ging er davon aus, dass dieselbe Art von Gruppenaktivitäten auch in größeren Klassen funktionieren könnte. Entgegen aller Empfehlungen seiner Kollegen, die Kurse in eine Reihe von Vorträgen umzuwandeln, entschied sich Michaelsen dafür, den Großteil der Kurszeit mit Gruppenarbeit zu füllen. Bald merkte er, dass seine Lehrstrategie funktionierte, sogar besser, als er es sich vorgestellt hatte. Die Studenten nahmen die Lernmethode gut an, sie bewerteten sogar die Klassengröße positiv. Michaelsen fand heraus, dass seine Lehrstrategie mit Kleingruppenarbeit das Potenzial der Teams stark steigerte. Die Gruppen entwickelten sich durch den Prozess des Team-based Learning zu leistungsfähigen Lernteams. Er veröffentlichte daraufhin einige Studien zum Thema der Entwicklung von und Umgang mit effektiven Lerngruppen (Michaelsen 1989; Watson 1993).

Für Kleingruppenarbeit werden in der englischsprachigen Literatur unterschiedliche Begriffe verwendet: learning groups, collaborative learning, cooperative learning, team-based learning. Nach Ansicht von Michaelsen (2002) gibt es drei Formen von Kleingruppenarbeit: gelegentlicher, eher zwangloser Gebrauch von Kleingruppenarbeit, kooperatives Lernen (strukturierter, individueller Gebrauch von Kleingruppenarbeit) und Team-based Learning. Letzteres sei ein besonderer Denkansatz, der sowohl dem Lehren als auch dem Lernen zu einer höheren pädagogischen Bedeutung verhilft. Michaelsen, Fink und Knight (2002) schreiben, dass Team-based Learning eine der effektivsten Lehrmethoden darstellt, wenn Lehrende das Problem haben, dass ihre Studenten nicht interessiert sind, sich nicht vorbereiten und das zu lernende Material nicht verstehen. Sie sind überzeugt davon, dass diese Lehrmethode sowohl die Erfahrungen der Lernenden als auch des Lehrers

verändern und verbessern kann. Wenn die Methode richtig angewandt würde, führt Team-based Learning zu vier Umwandlungen:

1. Es lässt aus Kleingruppen Teams werden.
2. Es lässt eine Technik zu einer Strategie werden.
3. Es verwandelt die Lernqualität.
4. Für viele Lehrer verändert oder bringt es die Freude am Lehren wieder zurück.

Millis und Cotell (1998) hingegen halten kooperatives Lernen für das übergeordnete Konzept mit Team-based Learning als einer von mehreren Varianten dieses Konzepts.

Zum gelegentlichen Gebrauch von Kleingruppenarbeit schreibt Michaelsen (2002): „Diese Methode wird oft von Lehrern verwendet, die ihre ersten Erfahrungen mit Kleingruppenarbeit sammeln. Beispielsweise indem ein Lehrer, nach einer kurzen Einführung in Form eines Vortrags die Klasse in Kleingruppen einige Minuten über ein spezielles Problem diskutieren lässt und nach dieser Zeit einige Studenten bittet, ihre Ergebnisse vorzustellen. Nach der Diskussion erläutert er dann weitere Aspekte oder fährt mit seinem Vortrag fort. Die Technik braucht kaum Vorbereitung, ist einfach zu gebrauchen und bringt mehr Abwechslung in die Unterrichtsstunden. Außerdem werden die Studenten zu aktivem Denken und sozialer Interaktion angeregt. Der Nachteil der Methode liegt in der Unmöglichkeit, damit eine höhere Form des Lernens zu erreichen. Sie bleibt nur ein kurzer Ausbruch aus dem üblichen Frontalunterricht."

Im Gegensatz zum gelegentlichen Gebrauch wird beim kooperativen Lernen häufig Kleingruppenarbeit benutzt, intensiv vorbereitet und strukturiert. In Gruppenarbeit werden nur bestimmte Themen erarbeitet. Phasen mit Kleingruppenarbeit werden in den ursprünglichen Lehrplan integriert, ohne das ganze Kurskonzept zu ändern.

Team-based Learning stellt nach Michaelsen einen noch intensiveren Gebrauch von Kleingruppenarbeit dar. Das Besondere an Team-based Learning ist, dass es keine Lehrtechnik, sondern eine Strategie ist, die es nötig macht, das gesamte Kurskonzept zu verändern. Ziel ist es, die Entwicklung von Hochleistungs-Lernteams zu unterstützen, das als solches eine höhere Leistung erzielen, als es für ein einzelnes Teammitglied möglich gewesen wäre (Michaelsen 1989). Als Gesamtstrategie statt als Einzelaktivität nützt die Methode des Team-based Learning viele verschiedene Aktivitätsformen, um aus den Kleingruppen wirkliche Teams zu machen. Ein

Themenblock ist dabei in drei Phasen eingeteilt: Vorbereitung, Anwendung und Bewertung.

In der Vorbereitungsphase bereiten sich die Studenten auf die Inhalte vor, indem sie Literatur lesen oder recherchieren. Dabei geht es nicht darum, tiefe Einsichten und völliges Verständnis zu erreichen, sondern einen allgemeinen Überblick über die wichtigsten Inhalte und Ideen zu erlangen. Daraufhin wird eine kurze Prüfung durchgeführt und die Ergebnisse besprochen. Dabei geht der Lehrer nur auf die Punkte ein, die die Studenten alleine nicht verstanden haben. Mit der Beschränkung auf kurze, prägnante Anmerkungen wird auch eine hohe Konzentration der Lernenden erreicht, da sie sich gerade intensiv mit dem Thema befasst haben und die Fragen noch gut in Erinnerung sind. In der Anwendungsphase lernen sie, mit den erlernten Inhalten Probleme zu lösen, Antworten auf Fragen zu finden, Erklärungen zu formulieren und Erwartungen abzugeben. Dazu bearbeiten die Lernteams eine Reihe von zunehmend schwierigeren Aufgaben. Jede Gruppe formuliert ihre eigenen Lösungen der Probleme, der Lehrer moderiert die Diskussion der verschiedenen Lösungen und gibt Feedback zur Qualität der Antworten. Die letzte Phase dient der Bewertung der Gruppenarbeit und des Einzelnen im Rahmen einer Prüfung.

Teams sind im Vergleich zu Kleingruppen charakterisiert durch ein hohes Maß von individuellem Einsatz zum Wohlergehen des Teams und an Vertrauen zwischen den Mitgliedern eines Teams. Dazu müssen folgende Bedingungen erfüllt sein: gemeinsame Zeit zur Interaktion, (intellektuelle) Ressourcen, eine anspruchsvolle Aufgabenstellung, deren Lösung zum gemeinsamen Ziel wird, und häufiges Feedback zur individuellen und zur Gruppenleistung. Auch große Klassen können auf diese Weise aktiviert werden. Team-based Learning führt zu einer weitaus höheren Aktivierung und tieferem Verständnis als jede zwanglose oder temporäre Gruppenarbeit. Die folgende Tabelle fasst die Empfehlungen dazu erschienener Forschungsliteratur nach Birmingham und McCord (2002) zur Entwicklung von Hochleistungs-Lernteams zusammen.

Wie groß sollen die Gruppen sein?	• Mindestens 5, am besten 6-7 Mitglieder<br>• So gemischt, wie die Klassenzusammensetzung es erlaubt
Wie sollen die Gruppen eingeteilt werden?	Vom Lehrer und in einer Weise, dass<br>• die Gruppen ähnlich zusammengesetzt sind in Bezug auf Unterschiede im Vorwissen und Fertigkeiten<br>• die Aufteilung von den Mitgliedern als angemessen wahrgenommen wird
Wie lange soll die Gruppeneinteilung stabil bleiben?	permanent
Welche Arten von Anweisungen für die Gruppen sind die effektivsten?	Anweisungen basierend auf gemeinsamen (nicht individuellen) Aufgaben, um<br>• inhaltsbezogene, auf der Grundlage des Gebens und Nehmens beruhende Diskussionen zu stimulieren<br>• eine Basis für Lernen unter Gleichrangigen zu schaffen<br>• die Gruppenentwicklung zu unterstützen<br>Zu schwierig für Einzelne, anspruchsvoll für eine Gruppe<br>Basierend auf für Studenten interessanten Gebieten
Welche Art von Bewertungsmaßstab soll angelegt werden?	Die meisten beinhalten die Bewertung<br>• der einzelnen Gruppenmitglieder<br>• der Gruppe als Team
Wie wichtig ist rechtzeitiges Feedback zur Leistung?	Essentiell für die Entwicklung von:<br>• hoher Teilnehmermotivation<br>• effektiver Gruppenfunktion

Tab. 5: Empfehlungen zur Entwicklung von Hochleistungs-Lernteams nach Birmingham und McCord (2002)

1.3.2. Problem-based learning

Problembasiertes Lernen (PBL), auch Problemorientiertes Lernen (POL) ist eine Lernmethode, bei der ein praktisches oder theoretisches Problem im Mittelpunkt steht, das die Lernenden weitgehend selbstständig lösen sollen. Diesen pädagogischen Ansatz kennzeichnet, dass reale und komplexe Problemstellungen aus dem beruflichen Alltag den Ausgangspunkt für selbstbestimmtes Lernen in der Gruppe bilden.

PBL wurde erstmals Mitte der 1960er Jahre an der McMaster University in Kanada eingesetzt und wird inzwischen weltweit an zahlreichen Universitäten als Lehrmethode verwendet. Norman und Schmidt (1992) schätzten 1992, dass rund 60 medizinischen Hochschulen weltweit PBL ganz oder teilweise eingeführt haben, und

weitere dabei sind, die Methode zu übernehmen. Die amerikanische Association of American Medical Colleges und die World Federation of Medical Education haben diese Entwicklung stark unterstützt (Walton and Matthews 1989). Mit der Einführung einer Experimentierklausel in der damaligen deutschen Approbationsordnung wurde PBL im Wintersemester 1999/2000 erstmals offiziell in der medizinischen Lehre an der Charité Berlin im Rahmen eines Reformstudienganges parallel zum Regelstudiengang angeboten. Seit der Einführung der neuen Approbationsordnung haben inzwischen weitere medizinische Fakultäten Studiengänge mit PBL-Elementen eingeführt.

Bei einer PBL-Lernsitzung wird die so genannte Methode des 7-Sprungs angewandt:

1. Klären unbekannter Begriffe
2. Themenfindung oder Problemdefinition
3. Brainstorming
4. Hypothesenbildung und Systematisierung
5. Lernzielformulierung
6. Lernzeit
7. Synthese

In der medizinischen Ausbildung ist das Problem häufig ein speziell konstruierter oder realer, klinischer Fall. Dieser wird mit einer Beschreibung eines Patienten begonnen, der ein oder mehrere typischen Krankheitszeichen oder Symptome zeigt (Norman and Schmidt 1992). In der Regel wird der Fall in einer Kleingruppe von etwa 10 Studierenden und einem ausgebildeten PBL-Tutor bearbeitet. Nach der Fallvorstellung werden gemeinsam Fragestellungen zu dem jeweiligen Fall formuliert. Diese können als Lernziele für das folgende Selbststudium oder die Arbeit in Kleingruppen genutzt werden. Insbesondere zu Beginn des Studiums ist dabei nicht das Ziel, am Ende eine konkrete Diagnose zu stellen, sondern grundlegende Zusammenhänge zu erarbeiten und zu verstehen. Ein PBL-Kurs ist meist thematisch statt fachbezogen aufgebaut und sollte deshalb am besten von einem Team aus Mitgliedern mit unterschiedlichem fachlichen Hintergrund organisiert werden. Die Fallbeschreibungen und die Möglichkeit zur Literaturrecherche (Internet, Bibliothek etc.) sind zwar die Kernelemente der PBL-Methode, können aber durch Lernressourcen wie audiovisuelle Medien und gelegentliche Vorlesungen ergänzt werden (Schmidt 1993). Norman und

Schmidt haben in einer Metaanalyse die wissenschaftliche Literatur zu den wichtigsten Theorien zu PBL zusammengefasst und kritisch betrachtet (Norman and Schmidt 1992). Drei Ansätze zum Effekt von PBL repräsentieren nach Ansicht der Autoren die Schritte im Prozess der klinischen Entscheidungsfindung, die sie in einer weiteren Studie beschrieben (Schmidt, Norman et al. 1990).

1. Der Erwerb von Faktenwissen in dem Kontext, in dem das Wissen später angewendet wird.

2. Das Beherrschen von allgemeinen Prinzipien und Konzepten auf eine Weise, dass diese auf folgende neuartige, aber ähnliche Probleme angewendet werden können.

3. Die Kenntnis von und Erinnerung an frühere Beispiele, um anhand von Ähnlichkeiten oder der Wiedererkennung von Mustern Probleme zu lösen.

Die wissenschaftlichen Grundlagen für die drei Ansätze beruhen auf sehr unterschiedlichen Forschungsbereichen der kognitiven Psychologie: Gedächtnis, Problemlösung, fallorientierte Argumentation, Begriffsbildung und Kategorisierung

Nach Barrows (1980; 1994) ist das primäre Ziel von PBL, die klinische Entscheidungsfindung und die Fähigkeit zur Problemlösung der Lernenden zu fördern. Außerdem soll PBL die Kluft zwischen vorklinischen und klinischen Fächern schmälern oder ganz beseitigen, indem sie anhand eines Falles sowohl die naturwissenschaftlichen Grundlagen als auch die klinisch-praktische Herangehensweise lernen. Lange Zeit wurde davon ausgegangen, dass eine generelle Fähigkeit zur Problemlösung mit PBL gelehrt und trainiert werden kann. Zahlreiche Studien in der Medizin und anderen Gebieten zeigten jedoch, dass die Übertragbarkeit auf andere Probleme gering ist. Dieses Phänomen wird als „content specificity" (dt. Inhaltsspezifität) bezeichnet. Norman und Schmidt (1992) ziehen aus den Folgerungen der Studien den Schluss, dass es keine allgemeinen Charakteristika des Problemlösens gibt. Sie schränken diesen Schluss zwar ein, betonen aber, dass bisher keine Beweise dafür vorliegen, dass sowohl herkömmliche als auch PBL-basierte Curricula zu einer verbesserten Fähigkeit zur Problemlösung der Studenten führen können, unabhängig vom Wissenserwerb.

Nach Bransford (1972) erleichtert eine Aktivierung des Vorwissens den folgenden Lernprozess von neuem Wissen. Diese Maxime der kognitiven Psychologie

unterstützen mehrere Studien in verschiedenen Fachgebieten (De Groot 1946; Spilich 1972); andere haben allerdings gezeigt, dass dieser Lernprozess nicht stattfindet, wenn kein relevantes Vorwissen aktiviert wird (Franks 1982; Schmidt, Norman et al. 1990). Norman und Schmidt (1992) zufolge ist die Diskussion eines Problems in Kleingruppen eine der Methoden, die relevantes Vorwissen zu Tage fördern kann. Mehrere Studien haben bereits den Effekt von PBL auf den Wissenserwerb und das Kurz- und Langzeitbehalten untersucht. Martenson et al (1985) haben beispielsweise herausgefunden, dass Studenten eines PBL-Kurses zwar keine Unterschiede im Kurzzeitbehalten von Wissen im Vergleich zu Studenten einer herkömmlichen Unterrichtsmethode zeigen, jedoch ein signifikant höheres Langzeitbehalten des Wissens (bis zu 60% höher) auch nach mehreren Jahren. Zu ähnlichen Ergebnissen kamen Eisenstaedt et al (1990) und Tans et al (1986); sie fanden sogar heraus, dass die Studenten der PBL-Methode im Kurzzeittest schlechter abschlossen.

Eine ausführliche Erläuterung des Wissens während der Lernphase kann dessen spätere Abrufbarkeit steigern. Sie kann in verschiedenen Formen stattfinden: im Rahmen einer Diskussion, beim Aufzeichnungen machen, beim Beantworten von Fragen und bei der Nutzung von Wissen, um ein Problem zu verstehen. Ein wichtiger Punkt stellt für Norman und Schmidt die Annahme dar, dass ein passender Kontext das Erinnern von Wissen erleichtert. Es gibt zahlreiche Gründe, warum PBL dies bietet. Wenn alle relevanten Prinzipien und Konzepte am Beispiel eines klinischen Falles in Kleingruppendiskussion gelernt werden, ist davon auszugehen, dass dieses Wissen gefestigter und besser abrufbar ist, wenn ein ähnliches Problem später im klinischen Alltag auftritt.

Wissenschaftliche Studien zu diesem Thema haben gezeigt, dass der Transfer von erlerntem Wissen auf analoge Probleme weitaus geringer als erwartet ist (Duncker 1945; Hayes 1977; Gick 1980; Gick 1983; Catrambone 1989). Norman und Schmidt (1992) kommen zu dem Schluss, dass Personen Lerninhalte auf dieselbe Weise bearbeiten sollten, wie sie später an ein neues Problem herangehen werden, damit sie die erlernten Prinzipien und Konzepte darauf übertragen können. Experimente von Needham und Begg (1991) zeigten, dass dieses Ziel mit Hilfe von PBL erreicht werden kann. Allerdings fanden die Wissenschaftler auch heraus, dass der Effekt nicht

stattfindet, wenn kein zeitnahes Feedback zur richtigen Lösung erfolgt. Nach Ansicht von Norman und Schmidt scheint das Erlernen von Konzepten während der Lösung eines Problems eine optimale Strategie für den spontanen Transfer der Konzepte auf ein neues Problem zu sein.

Patel et al (1991) fanden jedoch heraus, dass PBL-Studenten im Vergleich zu Studenten eines herkömmlichen Curriculums zwar mehr mögliche Erklärungen für ein Problem entwickelten, diese allerdings häufig inkorrekt waren. Eine andere Studie von Schmidt et al (1996) zeigte das Gegenteil. Sie untersuchten die Herangehensweise an Probleme von PBL-Studenten und Biochemikern im Vergleich zu Studenten eines konventionellen Curriculums und Internisten. Dabei kam heraus, dass die erste Gruppe sich zuerst mit den pathophysiologischen und biochemischen Aspekten beschäftigte und sie später mit den klinischen Problemen verknüpfte. Die zweite Gruppe verließ sich mehr auf ihr Gedächtnis und versuchte so die Lösung zu finden. Diese Strategie resultierte in wesentlich ungenaueren Antworten und mehr Fehlern bei den Studenten des konventionellen Curriculums. Norman und Schmidt erklären diese gegensätzlichen Ergebnisse mit den Unterschieden in der Strukturierung der PBL-Curricula und der Feedback-Praxis an den beiden Universitäten, an denen die Studien durchgeführt wurden.

Zahlreiche Studien konnten zeigen, dass Entscheidungen tatsächlich auf der Grundlage einer Ähnlichkeit der aktuellen Situation mit einem früheren Ereignis getroffen werden, ohne dass dies der betreffenden Person bewusst ist (Brooks 1987; 1991; Allen 1992). Allerdings schreiben Norman und Schmidt (1992), dass es bislang keine evidenzbasierten Daten zur Wirkung von PBL auf die fallbasierte Argumentation gibt.

In bisherigen Lernmethoden steuert nicht so sehr das intrinsische Interesse die Lernanstrengungen, sondern eher die Belohnung beispielsweise durch das Bestehen einer Prüfung oder einer besseren Verdienstmöglichkeit. Allerdings haben Studien ergeben, dass dies nicht nur das Lernen steuern kann, sondern auch das intrinsische Interesse mindern und die Lerninhalte weniger interessant machen kann (Lepper 1975). De Volder et al (1986) konnten zeigen, dass die Lernenden durch PBL von vornherein mehr in das Thema einbezogen werden. Allerdings konnten die Wissenschaftler keine direkte Auswirkung auf das Abschneiden in einem folgenden

Test zeigen. Andere Experten gehen davon aus, dass durch PBL das intrinsische Interesse der Studenten am Thema gesteigert wird mit der Folge einer erhöhten Lernmotivation. Bruner (1961) glaubt beispielsweise, dass Studenten, die an für sie bedeutsamen Problemen arbeiten, nicht nur für das Bestehen einer Prüfung lernen, sondern sich für weitere verwandte Themengebiete interessieren. Hunt (1971) erklärt diesen Effekt damit, dass Personen versuchen, sich zu informieren und ihre Wissenslücken zu füllen, wenn sie mit einer neuartigen, nicht sofort verständlichen Situation konfrontiert werden.

Sehr häufig wird in der Literatur außerdem das Ziel genannt, selbstbestimmtes Lernen durch PBL zu steigern. Diese Fähigkeit besteht nach Ansicht von Barrows (1980; 1994) aus Komponenten wie der klaren Formulierung von Lernbedürfnissen, der richtigen Nutzung geeigneter Lernressourcen, der Verankerung des neu erworbenen Wissen und Fähigkeiten im Selbststudium, um einerseits auf dem aktuellen medizinischen Wissensstand zu bleiben und andererseits auf geeignete Weise neuartige Probleme in der Praxis zu bewältigen. Norman und Schmidt folgern daraus, dass PBL eine positive Wirkung auf das selbstbestimmte Lernverhalten der Ärzte hat und diese dazu befähigt, mit Hilfe der Forschungsliteratur auf dem aktuellen Wissensstand zu bleiben.

1.3.3. Evidenzbasierte Medizin (evidence-based medicine, EBM)

Evidenzbasierte Medizin ist jede Form von medizinischer Behandlung, bei der patientenorientierte Entscheidungen ausdrücklich auf Basis von bewiesener Wirksamkeit (in klinischen Studien) getroffen werden. Sie steht damit im Gegensatz zu Behandlungsformen, bei denen kein Wirksamkeitsnachweis vorliegt. Der Begriff evidence-based medicine wurde Anfang der 90er Jahre von Gordon Guyatt (1991) aus der Gruppe um David Sackett an der McMaster University, Hamilton, Kanada, geprägt. Im deutschen Sprachraum wurde über das Konzept erstmals 1996 von Klemperer publiziert (1996).

EBM „ist der gewissenhafte, ausdrückliche und vernünftige Gebrauch der gegenwärtig besten externen Evidenz für Entscheidungen in der medizinischen Versorgung

individueller Patienten. Die Praxis der EBM bedeutet die Integration individueller klinischer Expertise mit der besten verfügbaren externen Evidenz aus systematischer Forschung“ (Sackett 1997). EBM beruht demnach auf dem aktuellen Stand der klinischen Medizin auf der Grundlage klinischer Studien, die einen Sachverhalt erhärten oder widerlegen (= externe Evidenz). Die EBM beschäftigt sich nicht mit der Durchführung von klinischen Studien selbst, sondern mit der systematischen Nutzung ihrer Ergebnisse.

Als individuelle klinische Expertise werden die Kenntnisse und die Fähigkeiten bezeichnet, die jeder Arzt im Laufe der klinischen Tätigkeit erlangt. Diese zeigt sich insbesondere in einer effektiven Diagnosefähigkeit und einer einfühlsameren Identifikation und Umgang mit der individuellen Situation eines Patienten, dessen Rechten und Präferenzen bei der klinischen Entscheidungsfindung. Als beste verfügbare externe Evidenz werden die Erkenntnisse klinisch relevanter Forschung insbesondere von patientenzentrierter klinischer Forschung zur Genauigkeit, Aussagekräftigkeit und Stärke diagnostischer Verfahren und der Wirksamkeit und Sicherheit von therapeutischen, präventiven und Rehabilitationsmaßnahmen angesehen. Externe Evidenz kann einerseits bisher akzeptierte diagnostische und therapeutische Verfahren entwerten, andererseits diese durch neuartige, wirksamere und sichere Methoden ersetzen. Gute Ärzte nützen beide Quellen, da keine der beiden Evidenzarten alleine genügt. Die Anwendung von individueller klinischer Expertise alleine birgt die Gefahr, Patienten nicht mehr zeitgemäß zu versorgen. Wenn sie jedoch nicht mit einbezogen wird, ist die individuelle Versorgung des Patienten gefährdet, da externe Evidenz in manchen Fällen nicht anwendbar ist. Dies zu entscheiden liegt in der Hand des klinisch erfahrenen Arztes.

In ihrem Artikel "Evidence based medicine: what it is and what it isn't" schreiben Sackett et al (1996) zur Kritik an EBM: „Die Kritik reicht vom Vorwurf, EBM sei ein alter Hut, bis hin zur Warnung, es handele sich um eine gefährliche Erfindung, vorangetrieben von Hochmütigen, die nur Maßnahmen zur Kostenreduktion unterstützen und die klinische Freiheit unterdrücken.“ Sackett et al entkräften diese Vorwürfe mit den Ergebnissen zahlreicher Studien zu Themen, wie das Verhalten eines Arztes durch die Einstellungen von Patienten gesteuert wird (Weatherall 1994)

oder dass eine evidenz-basierte Krankenversorgung für die große Mehrheit von Patienten praktizierbar ist (Ellis, Mulligan et al. 1995; Geddes, Game et al. 1996; Gill, Dowell et al. 1996). Sie schreiben: „Solche Studien zeigen, dass auch viel beschäftigte Kliniker, die ihre knappe Fortbildungszeit zum selektiven, effizienten und patientenbezogenen Suchen, Bewerten und Anwenden der besten verfügbaren Evidenz nutzen, EBM durchaus praktizieren können." Das Argument, EBM sei eine Kochbuchmedizin, entkräften sie mit der Erklärung, dass das Konzept von EBM nicht mit dem sklavischen Befolgen eines Kochrezeptes zur Patientenbehandlung vereinbar ist, da es eines Ansatzes bedarf, der die beste verfügbare externe Evidenz mit individueller klinischer Expertise und Patientenpräferenzen verbindet. Externe klinische Evidenz könne individuelle klinische Erfahrung zwar ergänzen, aber niemals ersetzen.

Die Anwendung von EBM ist ein Prozess des lebenslangen, selbstbestimmten Lernens, um auf die klinischen Fragestellungen bei der Betreuung von Patienten reagieren zu können (Sackett 1997). Sackett et al definieren dazu fünf Schritte zur praktischen Anwendung von EBM:

1. Formulierung von lösbaren Fragen zu identifizierten klinischen Fragestellungen bzw. Informationsbedarf
2. Finden der Quelle der besten Evidenz, die diese lösen kann – mit maximaler Effizienz
3. Kritische Beurteilung dieser Evidenz in Bezug auf die Validität und den klinischen Nutzen
4. Anwendung der Erkenntnisse in der klinische Praxis
5. Evaluation der eigenen Leistung

Es gibt zahlreiche Methoden, EBM zu lehren oder zu lernen. EBM ist ein häufiges Thema in Präsenzveranstaltungen wie Kursen, Konferenzen, Workshops, Journal-Clubs oder in Leitlinien oder Lehrbüchern. Die Wirksamkeit dieser Methoden wird diskutiert und intensiv beforscht. Die Studien kamen jedoch zu sehr unterschiedlichen Ergebnissen was die Methoden und die Effekte der Fortbildungsmaßnahmen betrifft. So zeigten einige Studien einen Effekt auf den Wissenserwerb der Ärzte (Green and Ellis 1997; Korenstein, Dunn et al. 2002; McCluskey and Lovarini 2005; Schilling, Wiecha et al. 2006; Nicholson, Warde et al. 2007; Shuval, Berkovits et al. 2007),

jedoch keine Änderung des Verhaltens der Ärzte (McCluskey and Lovarini 2005; Shuval, Berkovits et al. 2007) oder einen Effekt auf die Patientenversorgung (Shuval, Berkovits et al. 2007). Andere Studien fanden jedoch eine Wirksamkeit von Audit and Feedback-Maßnahmen bei der Vermittlung von EBM auf das Verhalten und die Patientenversorgung (Sibley, Sackett et al. 1982; Foy, MacLennan et al. 2002; Jamtvedt, Young et al. 2006), (vgl. Abschnitt I. Einleitung 1.2. Fortbildungsmaßnahmen und ihre Effektivität). Der Beweggrund, Fortbildungsveranstaltungen zur EBM zu nutzen, ist allerdings selten ein spezifisches klinisches, auf einen Patienten bezogenes Problem, sondern meist ein genereller Wunsch der Ärzte, sich auf den neuesten Stand zu bringen (Khan and Coomarasamy 2006).

Aufgrund der bisherigen Literatur, wie EBM gelehrt werden kann, entwickelten Khan et al eine Hierarchie der Effektivität der verschiedenen Lehrmethoden. Sie teilten diese dazu in die folgenden drei Levels ein: 1. Interaktive, klinisch integrierte Fortbildungen; 2a. Interaktive Präsenzveranstaltungen (in Hörsälen etc.); 2b. Passive Präsenzveranstaltungen mit klinisch integrierten Aktivitäten; 3. Passive Präsenzveranstaltungen. Das bedeutet, dass evidenzbasierte Medizin ebenso wie andere Fortbildungsthemen effektiver mit interaktiven Fortbildungsmethoden gelehrt und gelernt werden können. Fortbildungsangebote sollten deshalb überwiegend Themen der evidenzbasierten Medizin beinhalten, um das Wissen, die Fähigkeiten und das Verhalten der Ärzte und dadurch die Patientenversorgung wirksam zu verbessern und auf einen evidenzbasierten klinischen Stand zu bringen.

2. Ziele und Fragestellung

Auf den Grundlagen der Erkenntnisse zum Lernverhalten Erwachsener und insbesondere von Ärzten und den in der Literatur beschriebenen Lehrstrategien für effektive CME-Angebote wurde ein neuartiges, interaktives und fallorientiertes Fortbildungskonzept entwickelt, das im Folgenden näher beschrieben wird. Folgende übergeordnete Lernziele für die Fortbildungsveranstaltungen wurden formuliert:

- Vermitteln einer bleibenden Wissensverbesserung
- Vermitteln von Änderungen im diagnostischen und therapeutischen Vorgehen
- Steigerung der Kompetenz für klinische Entscheidungen in der Endokrinologie und Diabetologie
- Verbessern der Fähigkeit zur kritischen Würdigung der verfügbaren Evidenz für diese Entscheidungen
- Vermitteln und Vertiefen relevanter praktischer Fertigkeiten in der Endokrinologie und Diabetologie
- Vermitteln der Grenzen der eigenen Kompetenz (Überweisungsschwelle) an die Teilnehmer

Folgende Daten wurden zur Evaluation des Fortbildungskonzeptes erhoben:

- Motivation, Erwartungen und Akzeptanz der Fortbildungsreihe auf Seiten der Teilnehmer
- Vor- und Nachwissen der Teilnehmer (objektivierbarer Wissenserwerb)
- Daten zum Leistungs-, Diagnose- und Überweisungsverhalten der Ärzte von der Kassenärztlichen Vereinigung Bayern (KVB) in den Quartalen vor und nach der Fortbildungsteilnahme (objektivierbare Verhaltensänderung)

Im Folgenden werden die genauen Lernziele der einzelnen Fortbildungsthemen und die Hypothesen für die Verhaltensänderung der Teilnehmer dargestellt (siehe Tabelle 6).

Tabelle 6: Lernziele und Hypothesen zur Verhaltensänderung

	**Lernziele** Der Teilnehmer sollte gelernt haben/wissen….	**KV-Daten**	**Quar-tale**	**GOP/ PZN**	**Diagnose (ICD-10)**	Erwartungen zum Verhalten der Ärzte nach der Fortbildung	**Arzt-Gruppe**
**Diabetes und assoziierte Erkrankungen**							
**1.**	…wann die Diagnose Diabetes mellitus/ Diab. Fußsyndrom/ Nephropathie/ Neuropathie/ Retinopathie gestellt werden kann.	Diagnose Diabetes mellitus/ Diab. Fußsyndrom/ Diab. Nephropathie/ Neuropathie/ Retinopathie (Augenkomplikationen)	1.2006<br>2.2006<br>1.2007<br>2.2007		E 14.- (NNBez DM)<br>E 11.- (NIDDM)<br>E 10.- (IDDM)<br>E10/11/14.2- (mit Nierenkompl.)<br>E10/11/14.3- (mit Augenkompl.)<br>E10/11/14.4-(mit neurolog. Kompl.)<br>E10/11/14.5- (mit peripheren Kompl.)<br>E10/11/14.7- (mit multiplen Kompl.)	Erhöhte Diagnosen-Angabe	TN-Diabetes (n=76)
**2.**	…wann ein Diabetiker zum Spezialisten (Diabetologen) überwiesen werden sollte.	Überweisungsfrequenz zum Diabetologen*	1.2006<br>2.2006<br>1.2007<br>2.2007		E 14.- (NNBez. DM)<br>E 11.- (NIDDM)<br>E 10.- (IDDM)	Erhöhte Überweisungs-frequenz bei Erstdiagnose und schlechter Einstellung der Patienten	TN Diabetes (n=76)
**3.**	Betreuung von Diabetikern	Abrechnungsfrequenz der Betreuungspauschale	1.2006<br>2.2006<br>1.2007<br>2.2007	32022 (manifester DM)	E 14.- (NNBez. DM)<br>E 11.- (NIDDM)<br>E 10.- (IDDM)	Erhöhte Abrechnungs-frequenz	TN Diabetes (n=76)
**4.**	…dass jeder Diabetiker 1 x im Jahr zur augenärztlichen Kontrolle gehen sollte.	Überweisungsfrequenz zum Augenarzt*	1.2006<br>2.2006<br>1.2007<br>2.2007	06333 (Fundus-kopie)	E 14.- (NNBez. DM)<br>E 11.- (NIDDM)<br>E 10.- (IDDM)<br>**Untergruppe**:<br>E10/11/14.3- (mit Augenkompl.)	erhöhte (1 x jährliche) Überweisungsfrequenz zum Augenarzt	TN Diabetes (n=76)
**5.**	…dass bei Diabetikern regelmäßig ein Test auf Mikroalbuminurie durchgeführt werden sollte.	Frequenz von Mikroalbuminurietests/ Diabetespatienten*	1.2006<br>2.2006<br>1.2007<br>2.2007	32135 (Urin-Mikro-albumin)	E 14.- (NNBez. DM)<br>E 11.- (NIDDM)<br>E 10.- (IDDM)<br>**Untergruppe:**<br>E10/11/14.2- (mit Nierenkompl.)	Erhöhte Frequenz von Mikroalbuminurietests bei Diagnose DM oder diab. Nephropathie	TN Diabetes (n=76)
**6.**	…dass bei Diabetikern eine sorgfältige Fußpflege mit optimaler Schuhversorgung wichtig ist.	Diagnose des Diabetischen Fußsyndroms	1.2006<br>2.2006<br>1.2007<br>2.2007	02311 (Ulkus/ Wunden Th.)	E 14.- (NNBez. DM)<br>E 11.- (NIDDM)<br>E 10.- (IDDM)<br>**Untergruppe:**<br>E10/11/14.5-(mit peripheren Kompl.)	Erhöhte Durchführung	TN Diabetes (n=76)
**Endokrinologie**							
7.	…wann die Diagnose eines sekundären Hypertonus gestellt werden kann (auch V.a.).	Diagnose sekundäre Hypertonie	1.2006<br>2.2006<br>1.2007<br>2.2007		I 15.- (Sekundäre Hypertonie)<br>I 10.- (primäre Hypertonie)	Erhöhte Diagnosen-Angabe	TN art Hypertonie (n=34)
8.	… mit welchen Methoden ein	Anzahl der Bestimmung von	1.2006	32385 (Aldosteron)	I 15.- (Sekundäre Hypertonie)	Erhöhte Anzahl der genannten	TN art

	sekundärer Hypertonus abgeklärt werden soll.	Aldosteron-Renin-Ratio, Katecholamine, Dexamethasonhemmtest, Nierensonographie*	2.2006<br>1.2007<br>2.2007	32386 (Renin)<br>32300 (A/NA)<br>32367 (Cortisol**)<br>33073 (Doppler-Duplex-Sonographie abdomineller Gefäße)	I 10.- (primäre Hypertonie)	Untersuchungen (evtl. mit Diagnose sekundäre Hypertonie)	Hypertonie (n=34)
9.	…wann der Verdacht auf eine bzw. die Diagnose einer Schilddrüsenerkrankung gestellt werden kann.	Diagnose der Schilddrüsenerkrankungen	1.2006<br>2.2006<br>1.2007<br>2.2007		E01.- bis E07.- (Schilddrüsenerkrankungen)	Erhöhte Diagnosen-Angabe	TN Schilddrüse (n=56)
10.	…wann eine Doppler-Duplex (DD) -Sonographie der Schilddrüse durchgeführt werden sollte.	Überweisungsfrequenz oder Durchführung der Schilddrüsen-Doppler-Duplex-Sonographie*	1.2006<br>2.2006<br>1.2007<br>2.2007	33012 (Schilddrüsen-Sonographie)<br>33075 (Farbduplex-sonographie)	E01.- bis E07.- (Schilddrüsenerkrankungen)	Erhöhte Überweisungsrate zur /Anzahl von Sonographien bei V.a. Schilddrüsenerkrankung	TN Schilddrüse (n=56)
11.	…wann eine Schilddrüsen-Szintigraphie indiziert ist.	Überweisungsfrequenz/ Durchführung Schilddrüsen-Szintigraphie*	1.2006<br>2.2006<br>1.2007<br>2.2007	17320 (Schilddrüsen-Szintigraphie)	E01.- bis E07.- (Schilddrüsenerkrankungen)	Erniedrigte Überweisungs-frequenz zur Szintigraphie nach Sonographie	TN Schilddrüse (n=56)
12.	…wann die Indikation zur Operation/ Radiojodtherapie gestellt werden kann.	Überweisungsfrequenz zur Operation/ Radiojodtherapie*	1.2006<br>2.2006<br>1.2007<br>2.2007	17370 (RJT)	E01.- bis E07.- (Schilddrüsenerkrankungen)	Erhöhte Überweisungs-frequenz zur Operation/ Radiojodtherapie	TN Schilddrüse (n=56)

KV= Kassenärztliche Vereinigung Bayern, GOP= Gebührensordnungsposition, PZN= Pharmazentralnummer, TN= Teilnehmer der Fortbildung, DM= Diabetes mellitus; NNBez.= nicht näher bezeichnet, NID= not insulin-dependent, ID= insulin-dependent, A/NA= Adrenalin, Noradrenalin, n= Anzahl

* pro behandelte Diabetiker/Patienten mit entsprechender Erkrankung
** bis 5x bei Suppressions/Stimulationstests, bis 3x bei Tagesprofil

II. Methoden

1. Pilotstudie

Das Fortbildungskonzept wurde erstmals im Rahmen einer Reihe von Intensivseminaren zu Kernthemen der Endokrinologie und Diabetologie eingesetzt. An sechs Veranstaltungen zwischen Februar und Juli 2006 (01.02.2006, 21.02.2006, 05.04.2006, 10.05.2006, 21.06.2006, 19.07.2006) nahmen Allgemeinärzte und Internisten aus dem Münchner Raum teil. Nach dieser Pilotphase wurde das Konzept für eine weitere Fortbildungsreihe zu Themen der Inneren und Allgemeinmedizin übernommen. Die Veranstaltungsreihe „Aus der Praxis – für die Praxis“ des Hausärztlichen Curriculums München (HCM) zeichnet sich durch folgende Charakteristika aus: Es ist interaktiv, fallbasiert, praxisbezogen, didaktisch innovativ und Industrie-unabhängig. Bei den Präsenzveranstaltungen werden praxisorientierte Brennpunktthemen bearbeitet, die im Folgenden mit Online-Lernfällen im Selbststudium vertieft werden können. Das HCM ist als zweijähriges Curriculum mit regelmäßigen Fortbildungsveranstaltungen im Abstand von etwa drei Monaten ausgelegt, in dem auf die wesentlichen klinisch-praktischen Fragen aus der internistischen und allgemeinmedizinischen Praxis eingegangen wird. Im Jahr 2006 (05.07.2006, 20.09.2006, 15.11.2006) fanden drei Fortbildungen mit zahlreichen Teilnehmern aus dem Münchner Einzugsgebiet statt, die in der Hauptstudie evaluiert wurden.

Um eine objektivierbare Verhaltensänderung durch das erlangte Wissen nachzuweisen, sollten die Daten zum Diagnose-, Leistungs-, Verordnungs-, Einweisungs- und Überweisungsverhalten der Ärzte von der Kassenärztlichen Vereinigung (KV) in den Quartalen vor und nach der Fortbildungsteilnahme verglichen werden.

An den Präsenzveranstaltungen der Pilotstudie nahmen 88 Teilnehmer teil, davon besuchten einige mehrere Veranstaltungen. Der Eingangsfragebogen (siehe Anhang 1) wurde von 49 Teilnehmern ausgefüllt. Von den Teilnehmern, die die Fragebögen ausfüllten, waren 30,6% Allgemeinärzte und 46,9% Internisten sowie 18,4% Arzt/Ärztin in Weiterbildung. Außerdem waren 2% Facharzt für Endokrinologie. Das

Alter der Teilnehmer lag zwischen 26-62 Jahren, Durchschnitt: 44,5 Jahre (Standardabweichung (SD): 9,1). 49% der Ärzte waren weiblich, 51% männlich. Die teilnehmenden Ärzte hatten ihren Facharzt seit durchschnittlich 11,7 Jahren (SD: 8,1) oder waren durchschnittlich im 5. (4,7) Weiterbildungsjahr (SD: 1,7). Sie behandelten im IV. Quartal 2005 etwa 103,7 endokrinologische Fälle (SD: 163,7) und 100,6 Patienten mit Diabetes (SD: 123,5). 28% der Teilnehmer meldeten sich für das Gesamtprogramm, 55,1% für ein Einzelseminar, 10,2% für den Block Diabetologie (3 Veranstaltungen) und 6,1% für den Block Endokrinologie (3 Veranstaltungen) an.

34,7% der Ärzte hatten 2005 an mehr als vier Fortbildungen zu den Themen Endokrinologie und Diabetologie teilgenommen, 28,6% an zwei bis vier Fortbildungen, 20,4% an einer Veranstaltung und nur 16,3% an keiner. Sie hatten durchschnittlich 21,6 CME-Punkte (Min.: 2; Max: 65, SD: 17,5) mit diesen Fortbildungen erreicht. Mit Online-Fortbildungen im Durchschnitt 38,7 CME-Punkte (Min: 2, Max: 200, SD: 62,9), mit Fax 21 Punkte (Min: 5, Max: 50, SD: 17,4) und mit Präsenzveranstaltungen 78,0 (Min: 30, Max: 250, SD: 51,6). 83,7% hatten 2006 bereits an Fortbildungsveranstaltungen teilgenommen oder sich für Fortbildungen angemeldet. Die Themen waren sehr vielfältig: am häufigsten wurde die Kardiologie (12 Nennungen), Diabetologie (10), Endokrinologie (8) und Neurologie/Psychiatrie (7) genannt.

Von der Fortbildung hatten die teilnehmenden Ärzte vorwiegend durch schriftliche Einladung (63,3%) und von Kollegen (22,4%) erfahren. Wichtige Gründe für die Teilnahme an der Fortbildung waren die Themen der Fortbildung, die Interaktivität und die Fallorientierung. Die Teilnehmer erwarteten vor allem konkrete Entscheidungshilfen für die Praxis, theoretischen Wissenszuwachs und neueste Erkenntnisse aus der Wissenschaft. Der Kontakt mit Kollegen und das Kennenlernen einer neuen Fortbildungsform wurden nur als mittelmäßig wichtig bewertet. Die Motivation der Teilnehmer war sehr hoch und bezog sich vor allem auf das Interesse an den Themen der Fortbildung und der Relevanz der Themen für ihre praktische Tätigkeit. Ihr eigenes Wissen über die theoretischen Grundlagen und über die Diagnostik und Therapie der in der Fortbildung behandelten Krankheiten schätzten sie als mittelmäßig ein (siehe Tabelle 8).

	N	Min	Max	MW	SD
**Zur Anmeldung zum Intensivseminar haben mich bewogen…**					
Themenschwerpunkte der Fortbildung	49	4	6	5,59	0,6
Interaktivität und Fallorientierung	46	1	6	4,65	1,2
Auswahl der Referenten	47	1	6	3,96	1,5
Zeitlicher Rahmen	40	1	6	3,35	1,6
**Von dieser Fortbildungsveranstaltung erwarte ich…**					
Theoretischen Wissenszuwachs	49	4	6	5,45	0,7
Konkrete Entscheidungshilfen für die Praxis	49	1	6	5,20	1,1
Neueste Erkenntnisse aus der Wissenschaft	48	1	6	4,71	1,2
Kontakt mit Kollegen	48	2	6	3,58	1,3
Kennenlernen einer neuen Fortbildungsform	45	1	6	3,58	1,6
**Ich bin an endokrinologischen und diabetologischen Themen interessiert.**	49	3	6	5,43	,9
**Es macht mir Spaß, mich mit Inhalten der Endokrinologie und Diabetologie zu beschäftigen.**	49	3	6	5,31	,9
**Die Thema Endokrinologie und Diabetologie sind für meine Tätigkeit von hoher Relevanz.**	49	2	6	5,00	1,1
**Ich würde gern mehr über Endokrinologie/ Diabetologie wissen, als ich jetzt weiß.**	49	3	6	5,33	,9
**Für mich ist es eine Herausforderung, mehr über Endokrinologie/ Diabetologie zu lernen.**	49	1	6	5,04	1,1
**Mein jetziges Wissen über die theoretischen Grundlagen der Endokrinologie/ Diabetologie schätze ich als gering ein.**	49	1	6	2,84	1,4
**Mein jetziges Wissen über die Diagnostik und Therapie von Patienten mit endokrinologischen Erkrankungen schätze ich als gering ein.**	49	1	6	2,88	1,4

Tab. 7: Motivation, Erwartungen und Vorwissen der Teilnehmer der Pilotstudie (Likert-Skala: 1= stimmt überhaupt nicht, 6= stimmt genau); N= Anzahl der Teilnehmer, die die Frage beantworteten, Min= minimal vergebener Wert, Max= maximal vergebener Wert, MW= Mittelwert, SD= Standardabweichung

In der Abschlussevaluation (siehe Anhang 2) bewerteten die Teilnehmer die Inhalte als angemessen schwierig, das Seminar als sehr abwechslungs- und lehrreich. Sie gaben an, dass die Fälle sie stark zum Mitdenken angeregt hätten und die erlernten Inhalte ihr Verhalten in der Praxis verändern würden. Die Erwartungen an das Seminar wurden bei der Mehrheit der Befragten erfüllt und sie hatten insgesamt Spaß bei der Fortbildung. Mehr konkrete Entscheidungshilfen oder theoretischen Wissenszuwachs wollten die meisten nicht (siehe Tab. 9).

	N	Min	Max	MW	SD
**Die Inhalte waren für mein Vorwissen angemessen schwierig.**	88	1	6	4,32	1,4
**Das Seminar war abwechslungsreich.**	88	1	6	5,09	1,0
**Die Fälle haben mich zum Mitdenken angeregt.**	88	1	6	5,22	0,9
**Ich habe in diesem Seminar viel gelernt.**	88	1	6	4,70	1,1
**Die erlernten Inhalte werden mein Verhalten in der Praxis verändern.**	88	1	6	4,44	1,3
**Insgesamt hat das Seminar meine Erwartungen erfüllt.**	88	1	6	5,00	1,0
**Ich hätte mir mehr konkrete Entscheidungshilfen für die Praxis gewünscht.**	88	1	6	3,00	1,6
**Ich habe mehr theoretischen Wissenszuwachs vom Seminar erwartet.**	88	1	6	2,49	1,4
**Ingesamt hat mir das Seminar Spaß gemacht.**	88	1	6	5,28	,9

Tab. 8: Akzeptanz der Teilnehmer der Pilotstudie (Likert-Skala: 1= stimmt überhaupt nicht, 6= stimmt genau), N= Anzahl der Teilnehmer, die die Frage beantworteten, Min= minimal vergebener Wert, Max= maximal vergebener Wert, MW= Mittelwert, SD= Standardabweichung

In den freien Kommentaren und Anregungen zur Fortbildung wurden das Fortbildungsformat (Gruppenarbeit, Fallbesprechungen, TED-System) positiv erwähnt (6 Nennungen) und als verbesserungswürdig vor allem der zeitliche Rahmen (3 Nennungen) und der Inhalt der Fortbildungsveranstaltungen (präzisere Angaben zu Fällen, stärker auf Therapiemöglichkeiten eingehen, mehr Infos zu Insulinpräparaten (6 Nennungen) genannt.

Im Bezug auf die Computerkenntnisse befragt (siehe Anhang 2), gaben 98% der Teilnehmer an, selbst mit einem Computer zu arbeiten (77,6% privat, 85,7% beruflich). Sie verbrachten durchschnittlich 13,7 Stunden pro Woche am Computer (Standardabweichung 11.5). Zu den häufigsten Tätigkeiten am Computer zählten das Schreiben von Texten (89,8%), die Nutzung von Nachschlagewerken (73,5%) und Lernprogrammen (69,4%). Die Teilnehmer schätzten ihre Computerkenntnisse als mittelmäßig (Likert-Skala (1-5): 3,16, SD: 1,0) ein. 61,4% der Teilnehmer hatten am Ende der Fortbildung Interesse, Online-Lernfälle als Nachbereitung zu bearbeiten.

Vor- und Nachwissenstest:

Die Teilnehmer der Pilotstudie lösten durchschnittlich 55,5% der Fragen im Eingangswissenstest erfolgreich und 77,1% der Fragen im Abschlusswissenstest. Im Vergleich zum Vorwissenstest beantworteten die Teilnehmer im Abschlusstest durchschnittlich 21,6% mehr Fragen richtig (SD=24,7, $p<0{,}001$).

Aufgrund der hohen Akzeptanz der Teilnehmer und des signifikanten kurzfristigen Wissenszuwachses wurde das Fortbildungskonzept auf eine weitere Fortbildungsreihe, das „Hausärztliche Curriculum München – Aus der Praxis, für die Praxis“ übertragen und in der vorliegenden Studie an einer größeren Teilnehmerzahl erprobt. Dabei wurde darauf geachtet, dass die Themen der evaluierten Veranstaltungen der Fortbildungsreihen die gleichen waren, um eine weitgehende inhaltliche Homogenität zu erreichen. Auch die Dozenten der Themen der Hauptstudie (Diabetes mellitus, arterielle Hypertonie und Schilddrüsenknoten) sowie die Fälle waren dieselben, die Folien der Vorträge unterschieden sich jedoch teilweise. Deshalb wurden auch die für die Pilotstudie formulierten Lernziele und Hypothesen angepasst (vgl. Tabelle 6 und 7: Lernziele Pilotstudie/Hauptstudie).

2. Hauptstudie: Teilnehmercharakteristika

An den drei Terminen der Fortbildungsveranstaltungen „Aus der Praxis – für die Praxis“ des Hausärztlichen Curriculums München (05.07.2006, 20.09.2006, 15.11.2006) nahm eine Gesamtteilnehmerzahl von 230 Personen teil. Da einige Teilnehmer zwei oder alle drei Veranstaltungen besuchten, ergab sich eine Zahl von 169 Ärzten. Der Eingangsfragebogen wurde von 159 Teilnehmern bei ihrem ersten Besuch ausgefüllt (siehe B1). 39 Eingangsfragebögen wurden bei der ersten Veranstaltung ausgefüllt, 69 bei der zweiten und 49 bei der dritten Fortbildung. Das bedeutet eine Rücklaufquote von insgesamt 94,1%.

Von den Teilnehmern, die die Fragebögen ausfüllten, waren 89 (56%) weiblich und 70 (44%) männlich. Das Alter der Teilnehmer lag zwischen 27-68 Jahren, im Durchschnitt 48,4 Jahre (SD: 9,1). Von den Teilnehmern, die die Fragebögen ausfüllten, waren 60,3% Allgemeinärzte und 22,7% Internisten sowie 12,6% Arzt/Ärztin in Weiterbildung, außerdem ein geringer Anteil der Teilnehmer Facharzt auf einem anderen Gebiet.

Die teilnehmenden Ärzte hatten ihren Facharzt seit durchschnittlich 13,0 (0-38) Jahren (SD: 9,0) oder waren durchschnittlich im 4. (1.-7.) Weiterbildungsjahr (SD: 2,0).

Außerdem wurden die Teilnehmer zur Anmeldung bzw. Teilnahme an anderen Veranstaltungen der Reihe befragt. Die eigenen Angaben der teilnehmenden Ärzte stimmen weitgehend mit den Untersuchungsergebnissen der Teilnehmerlisten überein. Von 169 Personen nahmen diesen zufolge 32 (18,9%) an zwei Fortbildungsveranstaltungen teil, 16 Teilnehmer (9,4%) erschienen an allen drei Terminen. Das bedeutet, dass insgesamt 28,4% der Ärzte an zwei und mehr Veranstaltungen teilnahmen.

3. Studiendesign

Die vorliegende Studie ist eine nicht randomisierte Fall-Kontroll-Untersuchung (Pre- and Posttest-only design). Das Studiendesign beruht auf der Evaluation von drei Präsenzveranstaltungen (05.07.2006, 20.09.2006, 15.11.2006) und der Auswertung der Wissenstests und Daten zum Diagnose-, Leistungs- und Überweisungsverhalten der teilnehmenden Ärzte vor und nach der Fortbildungsintervention im Vergleich mit einer Kontrollgruppe. Zudem war eine Evaluation von Daten zum Verschreibungsverhalten geplant, die aus Datenschutzgründen nicht möglich war.

Die Teilnehmer nahmen zuerst an einer oder mehreren Präsenzveranstaltungen in Folge teil. Die Teilnahme an der Fortbildungsveranstaltung war kostenpflichtig und freiwillig, ebenso die Teilnahme an der Befragung mittels Fragebögen und der Wissenstests. Zusätzlich bekamen die Teilnehmer eine schriftliche Zusammenfassung der behandelten Themen der Präsenzveranstaltung in Form eines Syllabus zum Selbststudium. Die Auswertung der anonymisierten Daten zum Diagnose-, Leistungs- und Überweisungsverhalten der teilnehmenden Ärzte fand retrospektiv statt und bezog sich auf zwei Quartale vor der Teilnahme an der Fortbildungsveranstaltung und im Vergleich dazu die zwei entsprechenden Quartale im Jahr darauf. Die jeweils ersten zwei Quartale eines Jahres wurden aus Gründen der Homogenität miteinander verglichen, da erfahrungsgemäß die Diagnosen, Leistungen und Überweisungen jahreszeiten- und deshalb auch quartalsabhängig differieren. In denselben Quartalen wurde das Verhalten der Ärzte der Kontrollgruppe auf die gleiche Weise analysiert.

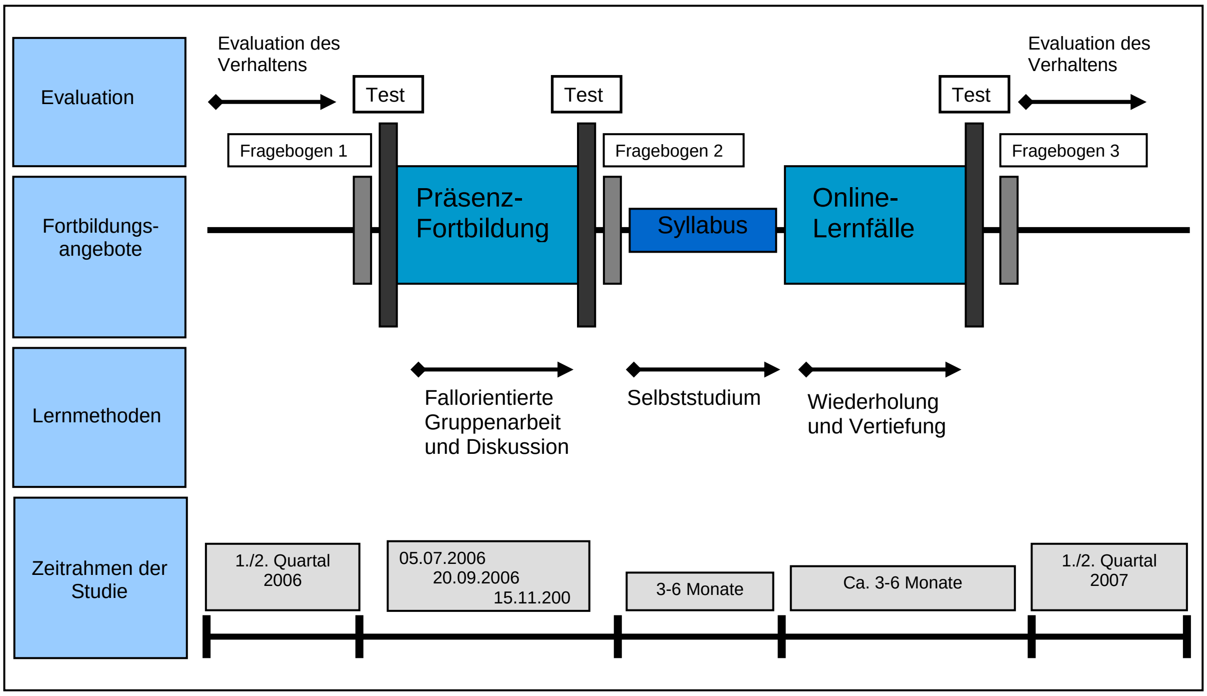

Abb. 2: Studiendesign: Die Datenerhebung der vorliegenden Studie erstreckte sich von Januar 2006 bis Juli 2007. Sie umfasste die Evaluation der Motivation und Erwartungen, des Wissens und des Diagnose-, Leistungs- und Überweisungsverhaltens der Fortbildungsteilnehmer vor der Präsenzveranstaltung mittels eines Fragebogens, eines Vorwissenstests und Daten der Kassenärztlichen Vereinigung (KV). Nach der Präsenzfortbildung wurden dann in gleicher Weise Daten zur Akzeptanz, zum Wissenserwerb und KV-Daten erhoben. Die verwendeten Lernmethoden umfassen fallorientierte Gruppenarbeit (Team-based Learning), Vorträge mit Diskussion und Selbststudium. Die Evaluation des Langzeitbehaltens des erlernten Wissens ist in einer Folgestudie mittels Online-Lernfällen geplant.

4. Beschreibung des Fortbildungskonzeptes

4.1. Präsenzveranstaltung

Format der Fortbildung:

Die Fortbildungsveranstaltungen sind in zwei große Themenblöcke zu drei bis vier klinischen Kernfragen unterteilt. Jeder Themenblock dauert etwa 120 Minuten und ist in einer Art „Sandwich“-Prinzip gegliedert (siehe Abbildung 3)

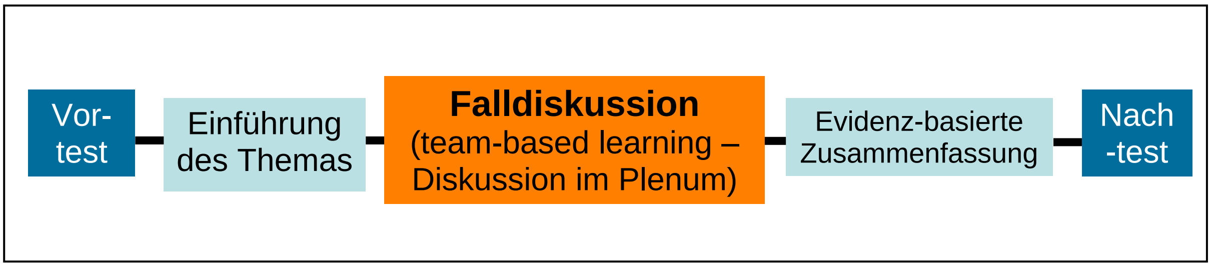

Abb. 3: Konzept der Fortbildung

Nach der Begrüßung der Teilnehmer beginnt die Fortbildung mit einem Vorwissenstest als personalisierte TED-Abfrage (siehe Abbildung 4) im Single-best-answer-Format. In diesem Fragenformat ist immer nur eine Antwortmöglichkeit unter mehreren richtig. Es werden jeweils fünf Fragen zu jedem Themenschwerpunkt gestellt. Dabei wird darauf geachtet, dass in den Expertenvorträgen und den klinischen Fällen die abgefragten Inhalte behandelt werden. Mit dem TED-System ist es möglich, die Abstimmungsergebnisse der Teilnehmer darzustellen, ohne die Lösungen zu verraten. Die Ergebnisse geben für die Teilnehmer selbst und die Dozenten Aufschluss über den Wissensstand vor der Präsenzveranstaltung und zeigen Defizite auf, auf die der Dozent daraufhin ausführlich eingehen kann. Außerdem aktiviert der Vorwissenstest die Aufmerksamkeit der Teilnehmer, da die richtigen Antworten auf die Fragen im Laufe des Vortrages erarbeitet und dargestellt werden.

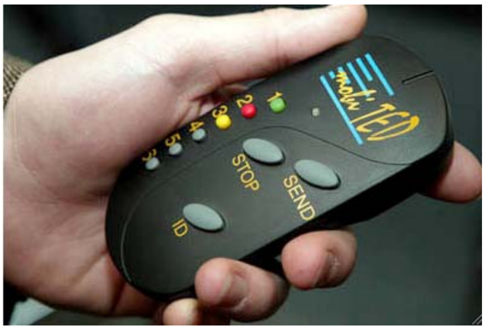

Abb. 4: Mobi-TED

Nach dem Vorwissenstest folgt ein 10-20-minütiger einführender Vortrag des Referenten zum ersten Themenschwerpunkt der Veranstaltung, in dem die Kernfragen kurz skizziert werden. Anschließend beginnt eine etwa halb- bis einstündige Aktivierungsphase in Form von Team-based Learning. In Kleingruppen (4-7 Teilnehmer) werden drei bis vier Kasuistiken zum ersten Themenschwerpunkt gemeinsam von den Teilnehmern ohne Moderation bearbeitet.

Die Fälle sind dabei folgendermaßen aufgebaut: Sie sind meist nur auf wenige Seiten im Syllabus beschränkt und beginnen mit einer Seite über die Anamnese und die ersten Befunde des Fall-Patienten. Darauf folgen Fragen im Multiple-Choice-Format zu Diagnosen, zum weiteren diagnostischen oder therapeutischen Vorgehen. Dabei

sind immer einige Antworten vorgegeben, es gibt aber auch die Antwortmöglichkeit X, bei der die Teilnehmer eigene Lösungsvorschläge formulieren können. Bei diesen Fragen können im Gegensatz zum Vor- und Nachwissenstest mehrere Lösungsmöglichkeiten ausgewählt werden und richtig sein. Sie werden auf Papier beantwortet (siehe Abbildung 5). Auf den nächsten Seiten sind dann weitere Befunde, zum Beispiel Laborergebnisse und Ergebnisse technischer Untersuchungen, und weitere Fragen zu finden. Diese Fragen zum Fall müssen von der Gruppe gemeinsam beantwortet und ein Gruppensprecher gewählt werden, der die Lösungen im Plenum vertritt. Insgesamt bearbeiten die Kleingruppen zwei bis drei Fälle innerhalb von ca. 30-60 Minuten, ohne die Lösungen der Fragen oder die abschließenden Verläufe der Fallpatienten zu erfahren.

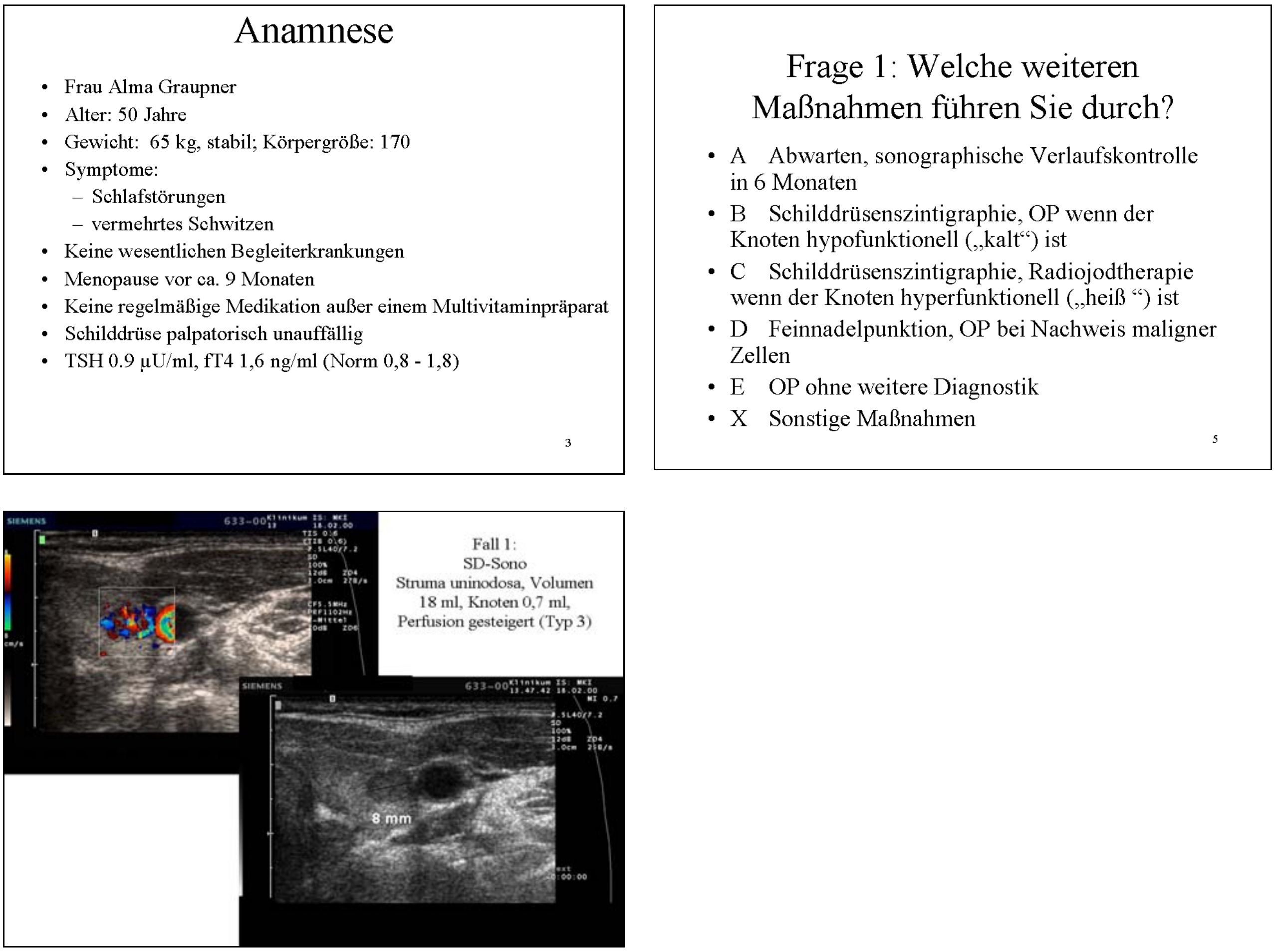

Abb. 5: Beispiel einer Falldarstellung auf Papier

In der folgenden 20-minütigen Reflexionsphase findet eine strukturierte Falldiskussion statt, bei der alle Gruppen ihre Lösungsvorschläge vorstellen und die Ergebnisse im Plenum intensiv mit dem Dozenten besprochen werden. Dazu bekommen die Gruppen Abstimmungskarten mit den Buchstaben der Lösungsmöglichkeiten (A-G, X), die der Gruppensprecher hochhält, damit der Dozent und Moderator die Lösungen sehen kann. Auf diese Weise sind Übereinstimmungen und Abweichungen der Gruppenlösungen gut sichtbar und können diskutiert werden. Eine kontroverse Diskussion ist hierbei erwünscht und wird durch die verschiedenen Antwortmöglichkeiten der Fragen zum Fall – insbesondere der Antwortmöglichkeit X - unterstützt. Abgeschlossen wird jeder Themenschwerpunkt mit einer auf die besprochenen Fälle bezogenen systematischen und evidenzbasierten Zusammenfassung des Dozenten (20-30 Minuten). Nach einer Pause folgt der zweite Themenblock, der wie der erste aufgebaut ist. Am Ende des Seminars wird mit Hilfe eines Abschlusswissenstests im TED-Format evaluiert, ob ein objektiver Wissenszuwachs zu den praxisrelevanten Fragestellungen stattgefunden hat. Dazu werden dieselben Fragen wie im Vorwissenstest gestellt, deren richtige Lösung erst am Ende der Fortbildungsveranstaltung dargestellt und diskutiert wird. Auf diese Weise können die Teilnehmer ihr erworbenes Wissen überprüfen und kann der Dozent sehen, ob die wichtigsten Kernfragen klar geworden sind oder einer weiteren Erklärung bedürfen.

Nach der Pilotphase wurde außerdem bei den HCM-Veranstaltungen zusätzlich eine Doppelmoderation bei der Falldiskussion eingeführt. Dabei moderiert ein niedergelassener Arzt die Diskussion der Kleingruppen im Plenum mit dem Spezialisten, der die Fragen leitliniengerecht beantwortet. Dies entspricht dem Prinzip der örtlichen Multiplikatoren, da niedergelassene Allgemeinmediziner, die unter den Teilnehmern der Fortbildung aufgrund ihres besonderen Engagements bekannt waren, diese Rolle übernahmen.

4.2. Aufbau des Syllabus

Jeder Teilnehmer erhält zu Beginn der Fortbildungsveranstaltung einen Syllabus (Aufbau des Syllabus siehe Anhang 3) mit einer Übersicht über das Veranstaltungsprogramm, den Folien der Vortragenden und den Falldarstellungen auf

Papier. Die Kosten für den Syllabus sind in der Anmeldegebühr inbegriffen. Damit die Fortbildungsteilnehmer genügend Platz für eigene Notizen haben, sind immer nur wenige Folien auf einer Seite abgedruckt. Die Reihenfolge des Syllabus folgt der des Veranstaltungsprogramms. Auf die Folien des Einführungsvortrags des Experten folgen die Falldarstellungen. Diese bestehen jeweils aus wenigen Seiten mit Informationen zur Krankheitsgeschichte und Fragen zu Differentialdiagnose, Diagnostik und Therapie. Die Fallkarten sind nacheinander abgedruckt, so dass die Teilnehmer nach der Bearbeitung einer Frage weitere Informationen bekommen, die natürlich teilweise auch die Lösung der vorangegangenen Frage zulassen. Die Fortbildungsteilnehmer werden jedoch daraufhin gewiesen, die Fragen im eigenen Interesse der Reihe nach zu lösen. Nach den Falldarstellungen (2-4 Fälle) folgen die Folien des zusammenfassenden Vortrags des Experten. Da in der Fortbildungsveranstaltung zwei Themenschwerpunkte behandelt werden, folgt der zweite Schwerpunkt in derselben Form im Syllabus.

Die Referenten und Moderatoren werden vor den Präsenzveranstaltungen genau instruiert (siehe Anhang 4 - Instruktionen für Referenten und Moderatoren). Sie erhalten einen Syllabus der vorangegangenen Veranstaltungen und Beispielfälle in Papier- oder elektronischer Form, um sich den Aufbau besser vorstellen zu können. Außerdem werden die Referenten persönlich oder telefonisch nochmals auf den Ablauf der Veranstaltung und die speziellen Anforderungen des Fortbildungskonzeptes vorbereitet. Dies scheint notwendig zu sein, da sich die Mehrzahl der Referenten beim ersten Mal den genauen Ablauf nicht genau vorstellen konnte. Außerdem wurde den Experten Hilfe bei der Erstellung der Fälle oder der Fragen für die Vor- und Nachwissenstests mit Korrektur und endgültiger Fertigstellung angeboten. Während der Veranstaltung ist immer einer der Organisatoren der Fortbildung anwesend, der das Fortbildungskonzept erklärt, auf dessen Einhaltung und den Zeitvorgaben besteht und durch die Fortbildungsveranstaltung führt.

4.3. Daten zum Diagnose-, Leistungs- und Überweisungsverhalten

Zur Auswertung des Diagnose-, Leistungs- und Überweisungsverhaltens der teilnehmenden Ärzte wurden anonymisierte Daten von der Kassenärztlichen Vereinigung Bayern (KVB) zur Verfügung gestellt. In einem ersten Schritt wurden die genauen Lernziele zu den einzelnen Fortbildungsthemen formuliert und geprüft, ob die Inhalte explizit in den Falldiskussionen und den Vorträgen vermittelt wurden und inwiefern sich ein dementsprechender Wissenserwerb auf das Verhalten der Ärzte auswirken könnte. Dazu wurden Fragestellungen und Hypothesen zur Verhaltensänderung der Ärzte formuliert. In einem zweiten Schritt wurden diese Fragestellungen und Hypothesen an die KVB weitergeleitet, um die Möglichkeiten zu eruieren, diese Fragestellungen zu beantworten. Aufgrund der Rückmeldung der KVB, welche Datenerhebung möglich und sinnvoll wäre, wurden die Fragestellungen verändert und angepasst (siehe Tabelle 6 und 7: Lernziele und Hypothesen zur Verhaltensänderung). Dabei musste beispielsweise leider die Fragestellung nach der Verordnung oder Durchführung von Patientenschulungen zum Thema Adipositas, Diabetes mellitus und Diabetisches Fußsyndrom aufgegeben werden, da diese Daten in den der KVB vorliegenden Daten nicht abgebildet und deshalb nicht auswertbar waren. Leider konnten nicht wie ursprünglich geplant zusätzlich Daten zum Verschreibungsverhalten der Ärzte ausgewertet werden, da diese Arzneimitteldaten aus datenschutzrechtlichen Gründen zum Zeitpunkt der Erhebung nicht zur Verfügung standen. Ausgewertet werden konnten Daten zur Diagnosestellung (nach der Internationalen Klassifikation der Krankheiten ICD-10), zu selbst erbrachten Leistungen der Ärzte (Gebührenordnungspositionen GOP) und zu Überweisungen zu Fachärzten mit deren Leistungen (siehe Tabelle 6 und 7 Lernziele und Hypothesen zur Verhaltensänderung).

Beim Diagnoseverhalten der Ärzte wurden die Häufigkeiten der ICD-10-Nummern ausgewertet und anhand deren Relevanz für die weitere Auswertung teilweise ICD-10-Subkategorien zusammengefasst oder unterteilt. Bei der Analyse der selbst erbrachten Leistungen (GOP) der Teilnehmer der Fortbildungen wurde sowohl deren Häufigkeit untersucht als auch diese Ergebnisse in Bezug zu den relevanten Diagnosen gesetzt. Bei der Auswertung der Überweisungsdaten wurde davon ausgegangen, dass bei

Überweisungen zu Fachärzten und den von diesen erbrachten Leistungen bei demselben Patienten die zuweisenden Ärzte (Teilnehmer der Fortbildung) diese initiierten hatten. Die Zuordnung - aus welchen Gründen die Überweisung zum Facharzt stattfand - konnte leider nicht ausgewertet werden, da nicht auf jeder Überweisung der Grund vermerkt wird. Deshalb konnte nur indirekt auf die Ursache geschlossen werden, beispielsweise über dieselbe Diagnose eines Patienten bei beiden Ärzten oder eine Leistung, die aufgrund dieser Diagnose bei einem Patienten von dem Facharzt erbracht wurde. Beispielsweise wurde untersucht, wie viele Patienten mit der Diagnose Diabetes mellitus zu einem Diabetologen überwiesen wurden oder bei wie vielen Patienten mit der Diagnose einer Osteoporose, die zu einem anderen Arzt überwiesen wurden, eine Knochendichtemessung durchgeführt wurde. Dabei wurde dann davon ausgegangen, dass der zuweisende Arzt (Teilnehmer der Fortbildung) bei diesen Osteoporose-Patienten die Indikation zur Knochendichtemessung gestellt hatte und sie daraufhin an einen geeigneten Facharzt überwiesen hatte. Es konnte auf diese Weise nicht ausgeschlossen werden, dass die Knochendichtemessung gar nicht der Grund für die Überweisung war, sondern der Patient nur zur weiteren Betreuung an den entsprechenden Facharzt überwiesen wurde und dieser dann die Indikation gestellt hatte.

Als Diabetologen wurde Fachärzte für Innere Medizin mit dem Schwerpunkt Endokrinologie und Diabetologie sowie diabetologisch besonders qualifizierte Ärzte (Berechtigung DB101) definiert.

Bei manchen Leistungen wurden sowohl die selbst erbrachten Leistungen der Teilnehmer ausgewertet als auch die Leistungen, die bei an einen Facharzt überwiesenen Patienten durchgeführt wurden (z.B. Anzahl selbst durchgeführter Schilddrüsensonographien der Teilnehmer sowie Anzahl der Schilddrüsen-sonographien, die bei überwiesenen Patienten mit Schilddrüsenerkrankungen von Fachärzten durchgeführt wurden). Die Leistungs- sowie die Überweisungsdaten wurden sowohl insgesamt ausgewertet als auch auf die jeweiligen relevanten Diagnosen bezogen. Dazu wurden die Daten über die Patientenkennung zusammengefügt.

Außerdem wurden nach der ersten Auswertung der Ergebnisse der Hauptstudie die Ärzte nach der Anzahl der von ihnen in den untersuchten Quartalen behandelten Patienten mit der jeweiligen Diagnose (z.B. Diabetiker oder Schilddrüsenpatienten) in Gruppen unterteilt und einer weiteren Analyse unterzogen. Mit Hilfe dieser Unterteilung sollte herausgefunden werden, ob die Teilnehmer der Fortbildung gemessen an ihrer klinischen Erfahrung ihr Verhalten unterschiedlich ändern. Damit sollte zum Beispiel analysiert werden, ob Ärzte, die wenige Patienten mit einer bestimmten Diagnose behandeln, ihr Verhalten stärker oder geringer ändern als Ärzte mit vielen Patienten. Dabei wurden die Ärzte anhand der Anzahl ihrer Patienten in zwei und in vier Gruppen visuell klassiert und die Intervalle aufgrund der gleichen Perzentile auf der Grundlage der durchsuchten Fälle gebildet (2 Gruppen: Perzentile 50%, 4 Gruppen: 25%).

Die Teilnehmer der Fortbildung zum Thema Schilddrüse wurden anhand der Anzahl von ihnen behandelter Patienten mit Schilddrüsenerkrankungen in folgende Gruppen unterteilt: 1. Einteilung in zwei Gruppen: Gruppe 1 mit niedriger Patientenanzahl (min.-151 Patienten) und Gruppe 2 mit hoher Patientenanzahl (152-max.). 2. Einteilung in vier Gruppen: Gruppe 1 mit der niedrigsten Patientenanzahl (min.-101,5), Gruppe 2 (101,6-151) und Gruppe 3 (152-269) mit niedrig und hoher mittlerer Patientenanzahl und Gruppe 4 mit der höchsten Patientenanzahl (270-max.).

Die Ärzte der Diabetes-Fortbildung wurden ebenfalls anhand der Anzahl ihrer Diabetes-Patienten in zwei bzw. vier Gruppen klassifiziert. Bei einer Unterteilung in zwei Gruppen waren alle Ärzte, die 0-109 Patienten mit Diabetes behandelten, in Gruppe 1, mit mehr als 109 Patienten in Gruppe 2. Bei der weiteren Unterteilung in vier Gruppen befanden sich in der Gruppe 1 die Ärzte, die 0-55 Diabetes-Patienten behandelten, in Gruppe 2 die Ärzte mit 56-109 Patienten, in Gruppe 3 Ärzte mit 110-205 Diabetes-Patienten und in Gruppe 4 Ärzte mit mehr als 206 Diabetes-Patienten.

Probanden: Anhand der Teilnehmerlisten der Präsenzveranstaltungen wurden die KV-Nummern der Teilnehmer recherchiert und jeweils den Themenschwerpunkten der Fortbildung, an denen die Ärzte teilgenommen hatten, zugeordnet. Diese Daten konnten nur von einem Teil der Fortbildungsteilnehmer erhoben werden, da nicht alle teilnehmenden Ärzte eine eigene Kassenzulassung (Ärzte in Weiterbildung,

Klinikärzte, Ärzte in Gemeinschaftspraxis ohne eigene Zulassung) und deshalb keine KV-Nummer hatten, die zur Erhebung der Daten zwingend notwendig war.

Bei den 45 Teilnehmern der Pilotstudie aus den offiziellen Teilnehmerlisten konnte bei 27 eine KV-Nummer zugeordnet werden. In der Hauptstudie waren es von 228 verzeichneten Teilnehmern 166 mit KV-Nummer. Dabei ergab sich die folgende Anzahl an Teilnehmern mit KV-Nummern pro Thema: Diabetes (76), Schilddrüsenknoten (56) und Arterielle Hypertonie (34).

Von der KVB wurde dann ausgewertet, ob jeder dieser Ärzte in allen entsprechenden Quartalen abgerechnet hatte. War dieses nicht der Fall (zum Beispiel bei Neuzulassungen in diesem Zeitraum), wurde der entsprechende Arzt aus der weiteren Auswertung ausgeschlossen. Bei der Pilotstudie wurden außerdem aus Gründen der Anonymität Ärzte ausgeschlossen, die aufgrund der weiteren Charakteristika (Praxisgröße, Spezialisierung etc.) aufgefallen wären und deshalb namentlich identifiziert hätten werden können. Da diese Gruppen nach statistischen Kriterien zu klein waren, um einerseits Effekte darstellen zu können und andererseits bei kleinen Gruppen die Anonymität der teilnehmenden Ärzte unter Umständen nicht gewahrt werden kann, wurde auf eine weitere statistische Auswertung der Pilotstudien-Ärzte verzichtet.

Da nicht alle Ärzte die entsprechenden Diagnosen stellten, Leistungen abrechneten oder ihre Patienten überwiesen, wurde die Gesamtzahl der Ärzte, deren Daten erhoben werden konnten, angegeben und die anschließende Auswertung auf diese Gesamtzahl bezogen. Es wurden sowohl absolute Zahlen (z.B. Anzahl der Diabetiker, die von einem Arzt zur Funduskopie zu einem Augenarzt überwiesen wurden) als auch der prozentuale Anteil der Patienten (z.B. Anteil der Diabetiker, die von einem Arzt zur Funduskopie zu einem Augenarzt überwiesen wurden, bezogen auf die Gesamtzahl der Diabetespatienten des Arztes) ausgerechnet und angegeben.

Die Auswertung der KV-Daten wurde im Rahmen eines Praktikums bei der KVB ausschließlich in den Räumen der KVB durchgeführt. Der Verfasserin der vorliegenden Arbeit lagen nur die verschlüsselten Daten der Teilnehmer der Fortbildung vor, die keine namentliche Zuordnung der Ärzte erlaubten. Die Teilnehmer waren mit einem Nummerncode anonymisiert dargestellt und nur den von

ihnen besuchten Fortbildungen zugeordnet. Aus datenschutzrechtlichen und statistischen Gründen wurde auch auf eine Datenauswertung verzichtet, wenn die Gruppengröße der Teilnehmer zu einer Fragestellung bei fünf Ärzten oder weniger lag.

Kontrollgruppe: Als geeignete Kontrollgruppe wurden alle niedergelassenen Allgemeinmediziner und hausärztlich tätigen Internisten in München und Umland definiert. 1023 Ärzte wurden über die Datenbanken der KVB identifiziert. Aus statistischen Gründen wurden etwa 10% dieser Ärzte zufällig ausgewählt und deren Diagnose-, Leistungs- und Überweisungsdaten als Kontrolle ausgewertet. So wurde eine Stichprobe von 87 Ärzten als Kontrollgruppe identifiziert. Auf diese Weise sollte für den statistischen Vergleich gewährleistet werden, dass zwei etwa gleich große Gruppen miteinander verglichen werden können. Die Daten der Ärzte der Kontrollgruppe wurden ebenfalls in anonymisierter Form ausgewertet, so dass keine Zuordnung möglich war. Nach einer ersten Auswertung der Daten der Teilnehmer der Fortbildungsveranstaltungen wurde die Auswertung der Kontrollgruppe auf die Hypothesen und Fragestellungen zum Thema Diabetes mellitus beschränkt (Vergleiche Tabelle 6).

5. Instrumente:

5.1. Fragebögen:

Zwei Fragebögen mit insgesamt 46 Items wurden jeweils direkt vor und am Ende der Veranstaltung an die Fortbildungsteilnehmer verteilt, um deren Charakteristika, Computerkenntnisse, Motivationen, Erwartungen, selbst eingeschätztes Wissen vor und nach der Fortbildungsveranstaltung und deren Akzeptanz zu evaluieren. Dazu wurde eine Likert-Skala von 1 bis 6 benutzt (1= stimmt überhaupt nicht, 6= stimmt genau). Es wurden sowohl Multiple Choice-Fragen als auch freie Fragen gestellt (siehe Anhang 1 und 2).

5.2. Vor- und Nachwissenstests:

Um das Vor- und Nachwissen der Teilnehmer zu prüfen, wurden zehn Multiple Choice-Fragen im Single-best-answer-Format direkt am Anfang und am Ende der Fortbildungsveranstaltung gestellt. In diesem Fragenformat ist immer nur eine Antwortmöglichkeit unter mehreren richtig. Es wurden jeweils fünf Fragen zu jedem Themenschwerpunkt gestellt. Dazu wurde ein elektronisches Abstimmungssystem (MobiTED) benutzt. Die Fragen des Vor- und Nachwissenstest waren identisch, die Lösungen wurden jedoch erst nach dem Nachwissenstest dargestellt. Das bedeutet, dass den Teilnehmern die Fragen zwar vom Vorwissenstest bekannt waren, deren Lösung jedoch nicht. Da die Fragen sich alle auf die spezifischen Lernziele der Fortbildung bezogen, konnten die Teilnehmer die Lösung während der Fallbearbeitung oder der Vorträge identifizieren und im Nachwissenstest richtig beantworten. Die Multiple Choice-Fragen wurden nach evidenzbasierten Richtlinien zum Verfassen von qualitätsvollen MC-Fragen erstellt (IAWF 1999; Case 2001; Haladyna 2002). Allerdings war es mit der vorliegenden Version des elektronischen Abstimmungssystems nicht möglich, die Ergebnisse der jeweils fünf Fragen pro Themenblock getrennt auszuwerten. Deshalb konnte nur eine Aussage über das Vorwissen und Wissen nach der Fortbildung in Bezug auf zwei Themen gemacht werden.

Beispiele für MC-Fragen im Vor- und Nachwissenstest:

Welche Laboruntersuchung ist bei Verdacht auf einen primären Hyperaldosteronismus primär indiziert?

A) Serum-Kalium
B) Aldosteron-/Renin-Quotient
C) NaCl-Belastungstest
D) Orthostasetest
E) Kalium-Ausscheidung im Urin

Zu den Diagnosekriterien des Diabetes mellitus zählt **nicht**: Bitte wählen Sie eine Antwort aus!

(A) Nüchtern-Blutzucker wiederholt >126 mg/dl (= 7,0 mmol/l).
(B) Symptome einer Hyperglykämie und Gelegenheits-Blutzucker > 200 mg/dl (= 11,1 mmol/l).
(C) Blutzucker im oralen Glukosetoleranztest (2h-Wert) > 200 mg/dl (= 11,1 mmol/l).
(D) Wiederholter Nachweis von Glukose im Urin
(E) mehrfach nachweisbare Mikroalbuminurie

Bei der Auswertung der Abstimmungsergebnisse wurden diejenigen Teilnehmer, die keine der zehn MC-Fragen beantworteten, ausgeschlossen. Damit sollte ein verfälschender Einfluss auf die Ergebnisse durch Teilnehmer, die nur an einem der beiden Tests teilnahmen, ausgeschlossen werden. Das TED-System und das Abstimmungsergebnis konnte den Teilnehmern nicht namentlich zugeordnet werden, sondern nur über einen anonymisierten Code, den die Teilnehmer auf den Fragebögen angeben mussten. Dieser bestand aus zwei Buchstaben und einer Ziffer (siehe Abbildung 6 und Anhang 1 und 2).

Code:	__________	__________	________
	1. Buchstabe des Geburts-namens Ihrer Mutter	1. Buchstabe Ihres Geburts-orts	Ihre Haus-nummer

Abb. 6: Anonymisierter Teilnehmer-Code

5.3. Lernfälle:

Vgl. Abschnitt 4.1. Präsenzveranstaltung

5.4. Datenauswertung zum Diagnose-, Leistungs- und Überweisungsverhalten:

Zur Verfügung standen die Datenbanken der Kassenärztlichen Vereinigung Bayern mit den gespeicherten Daten zu Diagnosen, Leistungen und Überweisungen. Diese Daten der teilnehmenden Ärzte mit Kassenzulassung wurden anonymisiert mit der einzigen Zusatzinformation, an welcher Fortbildung derjenige Arzt teilgenommen hatte, ausgewertet. Die Zuordnung der verschlüsselten Daten zu den einzelnen Ärzten war der Autorin der vorliegenden Arbeit nicht möglich, lediglich die Zuordnung zum Thema der Fortbildungsveranstaltung. Dies war nötig, um die Lernziele mit der erwarteten Verhaltensänderung korrelieren zu können.

Die Auswertung und deskriptive Statistik erfolgte mit Microsoft® Office Excel 2003 und SPSS 14.0 und 15.0. Für den statistischen Vergleich der Daten wurde eine Signifikanzanalyse mittels zweiseitigen t-Test für gepaarte Stichproben durchgeführt.

III. Ergebnisse

1. Präsenzveranstaltungen

1.1. Ergebnisse der Eingangsevaluation „Aus der Praxis – für die Praxis“ des Hausärztlichen Curriculums München

1.1.1. Fortbildungsverhalten der Teilnehmer:

Auf die Fragen nach dem Fortbildungsverhalten antwortete die Mehrzahl der Teilnehmer. Nur sechs Ärzte (3,8%) hatten im vorherigen Jahr (2005) nicht an Fortbildungen teilgenommen, 153 (94,1%) an einer oder mehreren Veranstaltungen. Die überwiegende Mehrzahl (100 Teilnehmer, 62,9%) hatte mehr als vier Fortbildungen besucht; ein weiteres knappes Drittel (45 Teilnehmer, 28,3%) an zwei bis vier Veranstaltungen (siehe Tabelle 14).

	Häufigkeit	Prozent
**Keine Angabe**	2	1,3
**nein**	6	3,8
**ja, einmal**	6	3,8
**ja, 2-4mal**	45	28,3
**ja, mehr als 4mal**	100	62,9
**Gesamt**	159	100,0

Tab. 14: Teilnahme an Fortbildungen 2005

Außerdem wurden die Teilnehmer nach der Anzahl der CME-Punkte gefragt, die sie mit verschiedenen Fortbildungsformaten erworben hatten. Allerdings wurde nicht gesondert nach der Nutzung dieser Fortbildungsangebote überhaupt gefragt. Da einige der Angaben unvollständig waren, kann nur eine bedingte Aussage über die Nutzung anderer Fortbildungsformate getroffen werden, insbesondere da jeweils nur 45 bzw. 39 Teilnehmer eine Angabe zu den mit Online- und Fax-Fortbildungen erworbenen CME-Punkten machten. Hieraus kann nicht geschlossen werden, dass die Teilnehmer diese Fortbildungsformate überhaupt nützen oder ob sie die Anzahl der erworbenen Punkte nicht angeben konnten. Die angegebene Anzahl an CME-Punkten unterschied sich deutlich. So erwarben die Teilnehmer zwischen 2 und 300 CME-Punkte,

durchschnittlich 58,8 Punkte (SD: 49,0). Auch die Anzahl der mit Online-CME-Angeboten erworbenen Fortbildungspunkte zeigte deutliche Unterschiede: Durchschnittlich erwarben die Teilnehmer 22,2 CME-Punkte online (SD 27,2). Deutlich weniger Punkte wurden mit Fax-Fortbildungsangeboten erworben: durchschnittlich 6,8 CME-Punkte (SD 6,3). Die meisten CME-Punkte wurden jedoch mit Präsenzveranstaltungen erreicht: durchschnittlich 74,8 Punkte (SD 58,6). Die unterschiedlichen, nicht zusammenpassenden Angaben über die CME-Punkte insgesamt und die in den einzelnen Formaten erworbenen Punkte sind durch fehlende Angaben der Teilnehmer zu erklären (siehe Tabelle 15).

	N	Min	Max	Mittelwert	SD
**CME-Punkte insgesamt**	96	2	300	58,81	49,0
**CME online**	45	0	140	22,22	27,2
**CME Fax**	39	0	20	6,79	6,3
**CME Präsenz-veranstaltung**	105	5	300	74,78	58,6
**Angaben**	28				

Tab. 15: CME-Punkte 2005 insgesamt und nach Veranstaltungsart

Die Mehrheit der Teilnehmer (94,3%, 150 TN) hatte 2006 bereits an Fortbildungsveranstaltungen teilgenommen oder hatte sich schon für Fortbildungen angemeldet. Nur 3,1% (5 TN) der Teilnehmer hatten noch an keiner Fortbildung teilgenommen oder sich noch nicht angemeldet; 2,5% (4 TN) machten keine Angabe. Außerdem wurden die Teilnehmer gebeten, die Themen der bereits besuchten oder der geplanten Fortbildungen zu benennen. Dabei zeigte sich eine sehr breite Themenauswahl der Teilnehmer. Zu den häufigsten Nennungen zählten Themen der Inneren und Allgemeinmedizin (siehe Anhang 4).

Desweiteren wurden die teilnehmenden Ärzte gefragt, wie sie von der Fortbildung erfahren hatten. Als Auswahl wurden schriftliche Einladung, Kollegen, E-mail und Internet genannt, außerdem weitere Nennungen ermöglicht. Rund 60% erfuhren durch schriftliche Einladung von den Fortbildungsveranstaltungen, weitere 10% durch schriftliche Einladung und andere Informationsquellen (Kollegen, E-mail, Zeitschrift)

und 8,2% nur von Kollegen. Weitere Informationsquellen wie E-mail, Internet, Zeitschriften oder über die Universität wurden seltener genannt.

1.1.2. Motivation, Erwartungen und Selbsteinschätzung der Teilnehmer:

Wichtige Gründe für die Teilnahme an der Fortbildung waren die Themen der Fortbildung, die Interaktivität und die Fallorientierung. Als weniger wichtig wurden die Auswahl der Referenten und der zeitliche Rahmen von den Ärzten bewertet. Die Teilnehmer erwarteten vor allem konkrete Entscheidungshilfen für die Praxis, theoretischen Wissenszuwachs und neueste Erkenntnisse aus der Wissenschaft. Der Kontakt mit Kollegen und das Kennenlernen einer neuen Fortbildungsform wurden als weniger wichtig eingestuft. Außerdem gaben die Teilnehmer an, sehr an den Themen der Fortbildung interessiert zu sein, Spaß an diesen Themen zu haben und gerne mehr über sie wissen zu wollen. Außerdem schätzten sie die Relevanz der Themen für ihre praktische Tätigkeit sehr hoch ein. Ihr eigenes Wissen über die theoretischen Grundlagen und über die Diagnostik und Therapie der in der Fortbildung behandelten Krankheiten schätzten sie als mittelmäßig ein (siehe Tabelle 18).

	N	Min	Max	MW	SD
**Zur Anmeldung zum Intensivseminar haben mich bewogen…**					
Themenschwerpunkte der Fortbildung	152	2	6	5,29	,9
Interaktivität und Fallorientierung	148	1	6	4,57	1,4
Auswahl der Referenten	146	1	6	3,65	1,6
Zeitlicher Rahmen	142	1	6	4,04	1,5
**Von dieser Fortbildungsveranstaltung erwarte ich…**					
Theoretischen Wissenszuwachs	154	1	6	5,39	,9
Konkrete Entscheidungshilfen für die Praxis	153	1	6	5,36	,9
Neueste Erkenntnisse aus der Wissenschaft	153	1	6	4,92	1,1
Kontakt mit Kollegen	147	1	6	3,35	1,4
Kennenlernen einer neuen FB-Form	144	1	6	3,28	1,5
**Ich bin an den Themen der heutigen Fortbildung interessiert.**	153	1	6	5,52	,8
**Es macht mir Spaß, mich mit Inhalten der heutigen Fortbildung zu beschäftigen.**	152	1	6	5,36	1,0
**Die Themen der heutigen Fortbildung sind für meine Tätigkeit von hoher Relevanz.**	152	1	6	5,28	1,0
**Ich würde gern mehr über die Themen der heutigen Fortbildung wissen, als ich jetzt weiß.**	150	1	6	5,01	1,2
**Für mich ist es eine Herausforderung, mehr über die Themen der heutigen Fortbildung zu lernen.**	146	1	6	4,75	1,3
**Mein jetziges Wissen über die theoretischen Grundlagen der heutigen Themen schätze ich als gering ein.**	145	1	6	2,59	1,1
**Mein jetziges Wissen über die Diagnostik und Therapie von Patienten mit internistischen Erkrankungen schätze ich als gering ein.**	143	1	6	2,48	1,2
Gültige Werte	121				

Tab. 18: Motivation und Erwartungen der Teilnehmer (Likert-Skala: 1 (stimmt überhaupt nicht) – 6 (stimmt genau), N= Anzahl der Teilnehmer, die die Frage beantworteten, Min= minimal vergebener Wert, Max= maximal vergebener Wert, MW= Mittelwert, SD= Standardabweichung

Überprüfung der Reliabilität: Für die Skala zur Erfassung der „Motivation", die aus 5 Items bestand, ergab sich eine Reliabilität von α = 0,843 (nach Cronbach). Die Skala „selbst geschätztes Vorwissen" wurde mit 2 Items erhoben und hat eine Reliabilität von α = 0,872 nach Cronbach.

Die Teilnehmer hatten zudem die Möglichkeit, weitere Erwartungen, Motivationen und Kommentare anzugeben. Es wurden sowohl die gute Qualität der angebotenen Fortbildungen als auch die Pharmaunabhängigkeit der Veranstaltungen und die Vorbereitung auf die Facharztprüfung oder Wiederauffrischung des Wissens nach Arbeitspause genannt.

**Kommentare zu Motivation etc.**
Allgemeinarztprüfung, Vorbereitung auf Facharztprüfung
Nach 5 Jahren Klinikabsenz ein Crash-Update, Crashkurs nach 5 Jahren Niederlassung
Pharmafreiheit, pharmaunabhängige Information
sehr gute Qualität bisher immer, stets hohe Qualität, FB-Form kenne ich bereits, finde sie sehr gut
Treue zur alten Klinik
Zeitpunkt, Dauer

Tab. 19: Freie Kommentare zu Motivation, Erwartungen etc.

1.1.3. Computerkenntnisse und -nutzung:

98,7% der Teilnehmer arbeiten selbst mit einem Computer (72,3% privat und beruflich, 20,1% nur beruflich, 3,8% nur privat). Sie verbringen durchschnittlich 17,5 Stunden pro Woche am Computer (Standardabweichung 15,9). Bei den Fragen zu den Tätigkeiten am Computer gaben 86,2% an, Texte geschrieben zu haben, 58,5% Computerspiele gespielt und Videos und Bilder angesehen zu haben. Ebenfalls 58,5% gaben an, schon mit Lernprogrammen am Computer gearbeitet zu haben. Die Teilnehmer schätzten ihre Computerkenntnisse als mittelmäßig (Likert-Skala (1-5): 3,1, SD: 0,9) ein.

- Texte geschrieben: 137 (86,2%)
- Computerspiele gespielt: 61 (58,5%)
- Mit Datenbanken gearbeitet: 88 (55,3%)
- Ein Programm installiert: 71 (44,7%)
- Lernprogramme genutzt: 93 (58,5%)
- Videos oder Bilder angesehen: 93 (58,5%)
- Nachschlagwerke genutzt: 97 (61%)
- Statistikprogramme genutzt: 61 (38,4%)
- Programmiert: 11 (6,9%)
- Grafiken oder Diagramme erstellt: 60 (37,7%)
- Mit Tabellenkalkulationen gearbeitet: 40 (25,2%)
- HTML geschrieben: 10 (6,3%)
- Webseiten gestaltet: 12 (7,5%)
- Musik gehört: 44 (27,7%)
- Musik gemacht: 3 (1,9%)
- Sonstiges: Praxisprogramme/-tätigkeit (N: 8), Fachzeitschriften/Literatursuche (N: 3), Emails (N: 3), Banking, Ebay, („bin zu alt, macht Personal, nur Patienten einchecken, Schein anlegen“)

Tab. 20: Was haben Sie selbst an einem Computer bereits gemacht?

Als letzte Frage auf dem Eingangsfragebogen wurden die Teilnehmer zu weiteren Kommentaren oder Anregungen zur Fortbildung aufgefordert. 25 Teilnehmer machten davon Gebrauch, 143 machten keine Angaben. Die Kommentare bezogen sich mehrheitlich auf die Fortbildungsqualität und das Konzept, die Räumlichkeiten und auf den Zeitrahmen der Veranstaltung (siehe Tabelle 21).

Fortbildungsqualität

- Aufbau und Konzept finde ich ansprechend und sinnvoll
- Aufbau: Arbeitsgruppen, Besprechung, Zusammenfassung: sehr gut;
- Take-home-messages, zu spezialistenorientiert
- Basics weglassen; ich will wissen, was ist neu? (Verbot Analog-Insulin, Inhalatives Insulin), was ist praxisrelevant?; die hier vorgestellte universitäre Luxusmedizin ist nicht auf die Kassenpraxis übertragbar
- Die Fortbildung war gut. Das Thema Diabetes ist zu groß, um ausführlich z.B. über neue Insuline oder Therapie zu sprechen, es wäre aber interessant ,das Thema "Diabetes mellitus-Therapie" anzubieten.
- Es war absolut super und lehrreich (beide Teile!);
- Gruppenarbeit weniger effektiv und zu lang, lieber strukturierte Fallbesprechung durch Dozenten, Fälle waren sehr gut; statt Gruppenarbeit lieber TED
- gut, praxisorientiert, dürfte aber nicht länger sein: sinkende Konzentration in letzter Stunde
- nur 1 Thema und kürzer (2 große und wichtige Themen anstrengend und erschöpfend; Vorteile: 1 Thema: weniger Stress, mehr Effekt
- Schulungsvortrag war für mich eher überflüssig, da wir bereits seit Jahren selbst schulen und inzwischen über eine Schulungsgemeinschaft verfügen
- sehr gelungene FB; fantastisch: hervorragende Einhaltung der Zeit, Interaktivität sehr hilfreich, 2. Teil nicht so effektiv (geringer Kenntnisstand der Gruppe); sehr gut: Selbsthilfegruppe-Vertreter
- stets sehr gut! Vielen Dank!
- totale Zeitüberschreitung bei Fall F: Referent konnte sein Thema nicht der Zeit anpassen; Gruppenarbeit eher uneffektiv: viel Tumult, nichts Neues, zweiter Experte sehr gut
- Vielen Dank! Sehr wertvoll! ausgezeichnet, da pharmaunabhängig
- zu komplizierte Fragen; Moderation teilweise chaotisch, Fragen werden nicht richtig verstanden, Zusammenfassung: super Idee endlich weg vom Monolog zum Dialog (praxisorientiert)

Räumlichkeiten:

- der Hörsaal für Gruppenarbeit nicht so geeignet
- Hörsaal für diese Art d. FB nicht geeignet. Uni sollte moderneren Seminarraum anschaffen, nicht nur teure Geräte
- in diesen Räumlichkeiten keine gruppendynamischen Fallbearbeitungen (zu laut), zeitraubend
- Klimaanlage
- Raum muss verdunkelt werden, um Texte besser lesen zu können

Zeitrahmen der Fortbildung:

- Dauer zu lange: eher 14-14.30 Beginn
- etwas konzentriertere FB: kürzer
- etwas zu langwierig: nur bei 1 Thema Gruppenarbeit, Konzentrationsfähigkeit lässt deutlich nach
- evtl. Anfang 15 Uhr,
- Veranstaltung zu lang

Sonstiges**:**

- Der Datendurst sämtlicher Institutionen ist nahezu unerträglich
- ein kleines Brot und Getränk zum Beginn (aus Praxis her hetzend) wäre nicht verkehrt gewesen
- bitte bei Laboruntersuchungen Referenzwerte angeben

Tab. 21: Freie Kommentare des Eingangsfragebogens, nach Themen geordnet

1.2. Ergebnisse der Abschlussevaluation „Aus der Praxis – für die Praxis" Hausärztliches Curriculum München

Am Ende der Fortbildungsveranstaltung beantworteten die Teilnehmer weitere zehn Fragen zur Evaluation. Insgesamt wurden 96 Fragebögen ausgefüllt. Die geringere Anzahl lässt sich mit dem früheren Verlassen der Fortbildung oder der Evaluationsmüdigkeit der Teilnehmer nach der Veranstaltung erklären.

1.2.1. Akzeptanz der Teilnehmer:

In der Abschlussevaluation bewerteten die Teilnehmer die Inhalte als angemessen schwierig, das Seminar als sehr abwechslungsreich und als sehr lehrreich. Sie gaben an, dass die Fälle sie sehr zum Mitdenken angeregt hätten und die erlernten Inhalte ihr Verhalten in der Praxis verändern würden. Die Erwartungen an das Seminar wurden bei der Mehrheit der Befragten erfüllt und sie hatten insgesamt Spaß bei der Fortbildung. Mehr konkrete Entscheidungshilfen oder theoretischen Wissenszuwachs wollten die meisten nicht (siehe Tabelle 23).

	N	Min	Max	MW	SD
**Die Inhalte waren für mein Vorwissen angemessen schwierig.**	95	1	6	4,96	1,2
**Das Seminar war abwechslungsreich.**	95	2	6	5,21	1,0
**Die Fälle haben mich zum Mitdenken angeregt.**	95	3	6	5,46	,7
**Ich habe in diesem Seminar viel gelernt.**	95	3	6	5,16	,8
**Die erlernten Inhalte werden mein Verhalten in der Praxis verändern.**	93	1	6	4,83	1,0
**Insgesamt hat das Seminar meine Erwartungen erfüllt.**	94	2	6	5,21	,9
**Ich hätte mir mehr konkrete Entscheidungshilfen für die Praxis gewünscht.**	94	1	6	2,60	1,4
**Ich habe mehr theoretischen Wissenszuwachs vom Seminar erwartet.**	94	1	6	2,42	1,4
**Ingesamt hat mir das Seminar Spaß gemacht.**	95	2	6	5,37	,9

Tab. 23: Evaluation der Fortbildung (Likert-Skala: 1= stimmt überhaupt nicht, 6= stimmt genau), N= Anzahl der Teilnehmer, die die Frage beantworteten, Min= minimal vergebener Wert, Max= maximal vergebener Wert, MW= Mittelwert, SD= Standardabweichung

Überprüfung der Reliabilität: Für die Skala zur Erfassung der „Akzeptanz der Fortbildung" ergab sich eine Reliabilität von α = 0,872. Diese Skala umfasste 7 Items. Hinsichtlich der Einschätzung der „Fehlenden Inhalte in der Fortbildung" betrug die Reliabilität der 2 Items α = 0,758.

Als letzte Frage auf dem Ausgangsfragebogen wurden die Teilnehmer nochmals zu Kommentaren oder Anregungen zur Fortbildung aufgefordert. 27 Teilnehmer machten davon Gebrauch, 69 machten keine Angaben. Die Kommentare bezogen sich mehrheitlich auf die Fortbildungsqualität und das Konzept mit Gruppenarbeit (siehe Tabelle 24).

Positive Bewertung:
- Einfach Klasse/prima,weiter so/war super, sehr instruktiv
- gute Fortbildung, ohne Pharma-Hintergrund: Bravo!
- recht gute FB, sehr praxisrelevant

Gruppenarbeit:
- Arbeitsgruppen nicht so effektiv wie gedacht - lieber gleich Fallvorstellung + allg. Diskussion, viel Zeitersparnis
- Gruppenarbeit: Idee gut, Ausführung zu verbessern/ Fallbesprechung im Auditorium besser als in kleinen Gruppen/ Für Gruppenarbeit ggf. TED-Nutzung sinnvoller
- Gruppengröße für Diskussionen kleiner, 4 Personen wünschenswert
- Kleingruppen im Hörsaal nicht geeignet

TED:
- TED-Abfrage: lieber keine doppelten Verneinungen in den Fragen
- TED macht Stress
- TED-Fragen gut

Zeitlicher Rahmen:
- Es war ganz schön anstrengend/ zu lange

Sonstiges:
- lieber nur 1 Komplex Inhalt und 2 Fortbildungsdaten

Tab. 24: Ausgewählte freie Kommentare des Ausgangsfragebogens nach Themen geordnet

1.2.2. Interesse an Onlinefällen:

63,1 Prozent der Teilnehmer hatten am Ende der Fortbildung Interesse, Online-Lernfälle als Nachbereitung zu bearbeiten.

1.3. Vor- und Nachwissenstests:

Die Teilnehmer lösten durchschnittlich 47,2% der Fragen im Eingangswissenstest erfolgreich und 70,3% der Fragen im Abschlusswissenstest. Im Vergleich zum Vorwissenstest (VT) beantworteten die Teilnehmer im Abschlusstest (AT) durchschnittlich 23,1 Prozentpunkte mehr Fragen richtig (SD=17,8, p<0,001), dies entspricht einer Steigerung von 48,9%.

Innerhalb der einzelnen Fortbildungsveranstaltungen zeigten sich jedoch auch Unterschiede in Bezug auf das Vorwissen der Teilnehmer und den Wissenserwerb. So war das Vorwissen bei Diabetes mellitus am niedrigsten (43,9% richtige Fragen im Vorwissenstest) und bei arterieller Hypertonie am höchsten (54,3% richtige Fragen im Vorwissenstest). Zum höchsten relativen Wissenszuwachs kam es beim Thema Diabetes mellitus (62,6% richtige Fragen im Nachwissenstest, 18,8 Prozentpunkte Wissenszuwachs, 42,9 % Steigerung) und beim Thema Schilddrüse (79,5% richtige Fragen im Nachwissenstest, 33,5 Prozentpunkte Wissenszuwachs, 42,1% Steigerung), am niedrigsten beim Thema arterielle Hypertonie (76,5% richtige Fragen im Nachwissenstest, 22,1 Prozentpunkte Wissenszuwachs, 40,7% Steigerung). Der Wissenszuwachs in Bezug auf die einzelne Veranstaltung war jedoch bei allen drei Fortbildungen statistisch signifikant. Auf eine große Heterogenität in Bezug auf das Wissen der Teilnehmer lassen auch die hohen Standardabweichungswerte schließen.

	**N**	**Eingangstest MW (SD)**	**Abschlusstest MW (SD)**	**Testvergleich MW (SD)**	**T**	**Signifi-kanz**
**Alle FB**	88	47,2 (17,1)	70,3 (19,0)	23,1 (17,8)	-12,1	< 0,001
**Arterielle Hypertonie**	24	54,3 (17,5)	76,5 (19,5)	22,1 (18,2)	-5,9	< 0,001
**Diabetes mellitus**	44	43,9 (15,6)	62,6 (18,1)	18,8 (16,9)	-7,4	< 0,001
**Schilddrüse**	20	46,0 (18,0)	79,5 (13,7)	33,5 (15,8)	-9,5	< 0,001

Tab. 25: Ergebnisse der Vor- und Nachwissenstests der Fortbildungs-teilnehmer (FB= Fortbildungen, N= Anzahl der Teilnehmer am Wissenstest, MW= Mittelwert, SD= Standardabweichung)

2. Daten zum Diagnose-, Leistungs- und Überweisungsverhalten

2.1. Teilnehmer der Fortbildung Sekundäre Hypertonie

Die Teilnehmer der Fortbildung Sekundärer Hypertonus stellten im 1. und 2. Quartal 2006 bei insgesamt 6969 Patienten die Diagnose primärer Hypertonus (ICD-10 I10) und bei 29 Patienten die Diagnose sekundärer Hypertonus. In den entsprechenden Quartalen 2007 belief sich die Anzahl der Patienten mit primärem Hypertonus auf 6894 und mit sekundärem Hypertonus auf 24.

**1. & 2. Quartal 2006**	**Häufigkeit**	**1. & 2. Quartal 2007**	**Häufigkeit**
Prim. Hypertonus	6969	Prim. Hypertonus	6894
Sekundärer Hypertonus	29	Sekundärer Hypertonus	24
- renovaskulär	2	- renovaskulär	2
- renoparenchymatös	10	- renoparenchymatös	8
- endokrin	4	- endokrin	2
- sonstige Ursachen	2	- sonstige Ursachen	2
- nicht näher bezeichnet	11	- nicht näher bezeichnet	10

Tab. 26: Anzahl der Patienten mit den Diagnosen nach ICD-10 der Teilnehmer der Fortbildung Sekundärer Hypertonus im 1. und 2. Quartal 2006 und 2007

Da die Anzahl der Patienten mit der Diagnose sekundäre Hypertonie so gering war, wurde auf eine weitere statistische Auswertung verzichtet.

Insgesamt werden die Leistungen, die untersucht werden sollten, überwiegend von Laborärzten durchgeführt. In der folgenden Tabelle ist die Anzahl der relevanten Gebührensordnungspositionen, die von allen bayerischen Ärzten im 1. und 2. Quartal 2006 und 2007 abgerechnet wurden, aufgeführt.

		**Anzahl der Untersuchungen in den 1. und 2. Quartalen 2006 und 2007**
**Laborleistung (GOP)**	**Katecholamine (32300)**	Ca. 8000
	**Kortisol (32367)**	Ca. 16500
	**Aldosteron (32385)**	Ca. 3300
	**Renin (32386)**	Ca. 2300

Tab. 27: Anzahl der relevanten Gebührensordnungspositionen zum Thema sekundärer Hypertonus, die von allen bayerischen Ärzten im 1. und 2. Quartal 2006 und 2007 durchgeführt wurden.

2.2. Teilnehmer der Fortbildung Schilddrüsenerkrankungen

2.2.1. Diagnose Schilddrüsenerkrankung und Unterdiagnosen

Die Ärzte, die an der Fortbildung zum Thema Schilddrüse teilnahmen, stellten im 1. und 2. Quartal 2006 bei 12489 Patienten die Diagnose einer Schilddrüsenerkrankung (ICD-10 E01-E07), im 1. und 2. Quartal 2007 bei 13243 Patienten.

Des Weiteren wurden die ICD-10-Nummern eingegrenzt auf die für die Fortbildung relevanten Diagnosen, E01, E04-E07, und diese entweder zusammengefasst unter der Diagnose Schilddrüse allgemein und unter den Diagnosen Hyperthyreose (E05), Struma (E04) und Jodmangelerkrankungen (E01). Nach dieser Einteilung stellten die Teilnehmer der Fortbildung Schilddrüse die Diagnose Schilddrüse allgemein 2006 9660mal, 2007 10086mal; die Diagnose Hyperthyreose 1282mal (2006) und 1240mal (2007); die Diagnose Jodmangelerkrankungen 1633mal (2006) und 1581mal (2007) und die Diagnose Struma 2006 5050mal und 2007 5284mal.

Im Durchschnitt stellten die Teilnehmer der Fortbildung Schilddrüse im 1. und 2. Quartal 2006 bei 201,2 Patienten pro Arzt die Diagnose einer allgemeinen Schilddrüsenerkrankung (E01, E04-07) und 2007 bei 210,1 Patienten. Dies ergibt eine fast signifikante Steigerung der Diagnosehäufigkeit (p=0,04) bei jedoch hoher Standardabweichung. Die Diagnose Hyperthyreose (E05) wurde in den untersuchten Quartalen 2006 durchschnittlich bei 26,7 und 2007 bei 25,8 Patienten pro Arzt gestellt. Das entspricht einer leichten Abnahme der Diagnosehäufigkeit, die nicht statistisch signifikant ist. Die Diagnose Jodmangelerkrankung (E01) wurde 2006 im Durchschnitt bei 45,3 Patienten und 2007 bei 43,9 Patienten von den Teilnehmern der Schilddrüsen-Fortbildung gestellt, was einem nicht signifikanten Rückgang entspricht. Auch die Diagnose „Nicht näher bezeichnete Schilddrüsenerkrankung“ (E07.8/9) wurde untersucht. Dabei zeigte sich ebenfalls ein leichter Rückgang der Diagnosehäufigkeit von durchschnittlich 14,8 Patienten (2006) auf 13,0 Patienten (2007), der jedoch statistisch nicht signifikant war. Insgesamt kann also von einer ziemlich konstanten Anzahl von Patienten mit einer Schilddrüsenerkrankung, die von den Teilnehmern der Schilddrüsen-Fortbildung behandelt werden, ausgegangen werden.

N-Pat-<br>Diagnose	N	Gesamtzahl der Patienten<br>1.&2.Q. 2006	1.&2. Quartal 2006<br>MW (SD)	Gesamtzahl der Patienten<br>1.&2.Q. 2007	1.&2. Quartal 2007<br>MW (SD)	Vergleich<br>MW (SD)	Signifi-kanz
**Schilddrüse allgemein**	48	9660	201,2 (184,6)	10086	210,1 (196,8)	8,9 (29,9)	0,046
**Hyper-thyreose**	48	1282	26,7 (18,9)	1240	25,8 (17,4)	-0,9 (8,1)	n.s.
**Jodmangel-erkrankung**	36	1633	45,3 (52,7)	1584	43,9 (49,2)	-1,4 (9,7)	n.s.
**Nicht näher bezeichnete SD-Erkr.**	30	444	14,8 (19,8)	390	13,0 (19,6)	-1,8 (6,1)	n.s.

Tab. 28: Vergleich der durchschnittlichen Patientenzahl mit der Diagnose einer Schilddrüsenerkrankung der Teilnehmer der Schilddrüsen-Fortbildung im 1. und 2. Quartal 2006 und 2007 (N-Pat-Diagnose= Anzahl der Patienten, bei denen die jeweilige Diagnose gestellt wurde, N= Anzahl der Teilnehmer, deren Diagnosedaten auswertbar waren, MW= Mittelwert, SD= Standardabweichung, Q= Quartal, SD-Erkr.= Schilddrüsenerkrankung)

Gruppenklassierungen

Die Teilnehmer der Fortbildung zum Thema Schilddrüse wurden außerdem anhand der Anzahl von ihnen behandelter Patienten mit Schilddrüsenerkrankungen in Gruppen unterteilt: 1. Einteilung in zwei Gruppen: Gruppe 1 mit niedriger Patientenanzahl (min.-151 Patienten) und Gruppe 2 mit hoher Patientenanzahl (152-max.). 2. Einteilung in vier Gruppen: Gruppe 1 mit der niedrigsten Patientenanzahl (min.-101,5), Gruppe 2 (101,6-151) und Gruppe 3 (152-269) mit niedriger und hoher mittlerer Patientenanzahl und Gruppe 4 mit der höchsten Patientenanzahl (270-max.).

2.2.2. Durchführung einer Schilddrüsensonographie bei Patienten mit Schilddrüsenerkrankungen:

22 Teilnehmer der Schilddrüsen-Fortbildung führten selbst Schilddrüsensonographien durch. Insgesamt führten die Ärzte im 1. und 2. Quartal 2006 2225 Schilddrüsensonographien durch, im 1. und 2. Quartal 2007 2135. Davon waren 2006 1518 Sonographien bei Patienten mit der Diagnose einer Schilddrüsenerkrankung und 2007 1424.

	N	Minimum	Maximum	Mittelwert	Standard-abweichung
**N_PatSD_Sono_2006**	21	0	315	72,29	82,25
**N_PatSD_Sono_2007**	21	0	294	67,81	75,01

Tab. 29: Anzahl der Patienten mit Schilddrüsenerkrankungen, bei denen die Teilnehmer der Fortbildung Schilddrüse eine Schilddrüsensonographie durchführten (N_PatSD_Sono= Anzahl der Patienten mit einer Schilddrüsenerkrankung, bei denen eine Schilddrüsen-sonographie durchgeführt wurde. 20061_2/2006= 1.&2.Quartal 2006, 20071_2/2007= 1.&2.Quartal 2007.)

Bei der statistischen Auswertung zeigte sich im Vergleich der Quartale vor und nach der Fortbildung eine leichte Abnahme der Untersuchungsfrequenz von Patienten insgesamt mittels Schilddrüsensonographie um 4,1 von durchschnittlich 101,1 auf 97,0 Patienten pro Arzt, die nicht statisch signifikant war.

Schilddrüsensonographien bei Patienten mit Schilddrüsenerkrankungen:

Patienten mit Schilddrüsenerkrankungen wurden von den Teilnehmern der Fortbildung Schilddrüse ebenfalls durchschnittlich seltener mittels Schilddrüsensonographie untersucht. Der durchschnittliche Anteil von Schilddrüsen-Patienten, die sonographiert wurden, sank um 6,8% von 10,3% der Schilddrüsen-Patienten pro Arzt auf 9,6% im Vergleich des 1. und 2. Quartals 2006 und 2007 (0,7 Prozentpunkte, p=0,346).

Es zeigte sich, wenn zwei Gruppen gebildet wurden, dass die Ärzte der Fortbildung Schilddrüse mit einer niedrigen Anzahl an Schilddrüsenpatienten 2007 30% weniger Schilddrüsensonographien bei diesen Patienten durchführten: bei 5,0% der Patienten im Jahr 2006 und bei 3,5% Patienten im 1. und 2. Quartal 2007 (1,5 Prozentpunkte, p=0,057). Die Ärzte mit einer höheren Patientenanzahl führten die Untersuchung in beiden untersuchten Zeitspannen bei dem gleichen Anteil an Schilddrüsen-Patienten (15,7%) durch. Wenn die Ärzte anhand der Anzahl von ihnen behandelter Patienten mit Schilddrüsenerkrankungen in vier Gruppen eingeteilt wurden, zeigte sich, dass die Gruppen 1 (niedrige Diagnosefrequenz), 2 (niedrige mittlere Diagnosefrequenz) und 4 (höchste Diagnosefrequenz) 2007 durchschnittlich 15-44% weniger Schilddrüsensonographien durchführten als 2006; die Gruppe 3 jedoch durchschnittlich 18,7% mehr Schilddrüsensonographien durchführte (2,7

Prozentpunkte, p=0,231). Hervorzuheben ist allerdings der große Unterschied der Mittelwerte der einzelnen Gruppen, die hohe Standardabweichung und dass insgesamt nur 21 Ärzte selbst Schilddrüsensonographien durchführten. Je mehr Patienten mit Schilddrüsenerkrankungen die Ärzte hatten, desto höher lag auch der Anteil der bei diesen durchgeführten Schilddrüsensonographien.

**N-Pat-SD**		**N**	**1.&2. Quartal 2006 MW (SD) in %**	**1.&2. Quartal 2007 MW (SD) in %**	**Vergleich MW (SD)**	**Signifikanz**
**SD-Pat**	**Alle TN**	48	10,3 (14,5)	9,6 (13,1)	-0,7 (5,4)	n.s.
	**Gruppe 1**	24	5,0 (11,6)	3,5 (8,3)	-1,5 (3,7)	0,057
	**Gruppe 2**	24	15,7 (15,4)	15,7 (14,2)	0 (6,7)	n.s.
	**Gruppe 1**	12	2,7 (9,4)	1,5 (5,2)	-1,2 (4,1)	n.s.
	**Gruppe 2**	12	7,3 (13,6)	5,5 (10,4)	-1,8 (3,4)	n.s
	**Gruppe 3**	12	14,4 (15,1)	17,1 (15,3)	2,7 (7,3)	n.s.
	**Gruppe 4**	12	17,1 (16,3)	14,5 (13,6)	-2,6 (4,9)	n.s.

Tab. 30: Vergleich des durchschnittlichen Anteils der Patienten mit Schilddrüsenerkrankungen der Teilnehmer der Fortbildung Schilddrüse, bei denen eine Schilddrüsensonographie durchgeführt wurde (GOP 33012) N-Pat= Anzahl der Patienten, bei denen eine Schilddrüsensonographie durchgeführt wurde, SD-Pat= Patienten mit der Diagnose einer Schilddrüsenerkrankung, N= Anzahl der Teilnehmer, deren Diagnosedaten auswertbar waren, MW= Mittelwert, SD= Standardabweichung, n.s.= nicht signifikant; Gruppe 1 (min.-151 Patienten mit Schilddrüsenerkrankung), Gruppe 2 (152-max.). Vier Gruppen: Gruppe 1 (min.-101,5), Gruppe 2 (101,6-151), Gruppe 3 (152-269), Gruppe 4 (270-max.).

2.2.3. Überweisung von Patienten mit Schilddrüsenerkrankungen zur Schilddrüsensonographie:

Neben der Auswertung der selbsterbrachten Leistungen wurde die Anzahl der Patienten mit Schilddrüsenerkrankungen, die zur Schilddrüsensonographie überwiesen wurden, evaluiert. Die Teilnehmer der Schilddrüsenfortbildung überwiesen im 1. und 2. Quartal 2006 insgesamt 1237 Patienten zur Schilddrüsensonographie (GOP 33012), in den entsprechenden Quartalen 2007 1290 Patienten. 2006 wurde die überwiegende Mehrheit der Patienten zu Internisten (190: 55,7%), Radiologen (530: 26,9%) und

Nuklearmedizinern (590: 16,8%) überwiesen; 2007 zu 47,6% an Internisten, 40,4% an Radiologen und 11,4% an Nuklearmediziner. Zur Farbduplexsonographie (GOP 33075) überwiesen die Ärzte der Fortbildung Schilddrüse 2006 (1. und 2. Quartal) insgesamt 1059 Patienten, davon die überwiegende Mehrheit (60,4%) zu Internisten, zu einem sehr geringen Anteil auch an Nuklearmediziner und Allgemeinärzte. 2007 überwiesen die Ärzte dagegen nur 893 Patienten zur Farbduplex-Sonographie, 67,7% davon zu Internisten.

Bei dem Vergleich der durchschnittlichen Anzahl der Patienten mit Schilddrüsenerkrankungen, die von den Teilnehmern der Fortbildung Schilddrüse zur Schilddrüsensonographie überwiesen wurden, zeigte sich eine Abnahme um 16,7% von 10,2% auf 8,5% der Schilddrüsenpatienten pro Arzt (1,7 Prozentpunkte, p=0,039). Auch hier wurden die Ärzte bei der Auswertung anhand der Anzahl ihrer Schilddrüsenpatienten in Gruppen klassifiziert. Es zeigte sich bei einer Unterteilung in zwei Gruppen in Gruppe 1 eine Abnahme der Überweisung zur Schilddrüsensonographie von durchschnittlich 13,9% der Patienten auf 10,4% (p=0,029), was einer 25,2%igen Abnahme entspricht. Bei der Gruppe 2 zeigte sich ein konstanter Anteil von ca. 6,5% der Patienten in beiden untersuchten Zeiträumen. Bei einer Aufteilung in vier Gruppen zeigte sich ein ähnliches Bild: Die Ärzte mit der höchsten Anzahl an Patienten mit Schilddrüsenerkrankungen (Gruppe 4) überwiesen 2006 im Durchschnitt 7,2% der Schilddrüsen-Patienten zur Schilddrüsensonographie und 2007 7,6%. Die durchschnittliche Steigerung von 5,5% war nicht statistisch signifikant (p=0,729). Alle drei Gruppen mit niedrigerer Diagnosefrequenz zeigten dagegen eine nicht signifikante Abnahme des Anteils an Patienten mit Schilddrüsenerkrankung, die zur Schilddrüsensonographie überwiesen wurden: bei Gruppe 1 nahm sie um 31,0% ab, bei Gruppe 2 um 16,7% und Gruppe 3 um 8,5%. Zudem fiel auf, dass die Ärzte mit weniger Schilddrüsenpatienten diese etwa doppelt so häufig zur Schilddrüsensonographie überwiesen wie die Ärzte mit einer hohen Patientenanzahl.

N-Pat-SD		N	1.&2. Quartal 2006 MW (SD) in %	1.&2. Quartal 2007 MW (SD) in %	Vergleich MW (SD)	Signifikanz
SD-Pat	Alle TN	48	10,2 (8,9)	8,5 (8,1)	-1,7 (5,8)	0,039
	Gruppe 1	24	13,9 (8,5)	10,4 (7,5)	-3,5 (4,3)	0,029
	Gruppe 2	24	6,6 (7,9)	6,5 (8,3)	-0,1 (2,9)	n.s.
	Gruppe 1	12	15,8 (9,7)	10,9 (9,3)	-4,9 (9,6)	n.s.
	Gruppe 2	12	12,0 (6,8)	10,0 (5,5)	-2,0 (5,0)	n.s.
	Gruppe 3	12	5,9 (6,3)	5,4 (5,1)	-0,5 (2,1)	n.s.
	Gruppe 4	12	7,2 (9,6)	7,6 (10,7)	0,4 (3,6)	n.s.

Tab. 31: Vergleich des durchschnittlichen Anteils der Patienten mit Schilddrüsen-erkrankungen der Teilnehmer der Fortbildung Schilddrüse, die zur Schilddrüsensonographie überwiesen wurden (GOP 33012) N-Pat-SD= Anzahl der Patienten mit Schilddrüsenerkrankung, die zur Schilddrüsensonographie überwiesen wurden, N= Anzahl der Teilnehmer, deren Diagnosedaten auswertbar waren, MW= Mittelwert, SD= Standardabweichung¸ Gruppe 1 (min.-151 Patienten mit Schilddrüsenerkrankung), Gruppe 2 (152-max.). Vier Gruppen: Gruppe 1 (min.-101,5), Gruppe 2 (101,6-151), Gruppe 3 (152-269), Gruppe 4 (270-max.).

2.2.4. Überweisung von Patienten mit Schilddrüsenerkrankungen zur Schilddrüsenszintigraphie (GOP 17320):

43 Teilnehmer der Fortbildung Schilddrüse überwiesen im 1. und 2. Quartal 2006 insgesamt 258 Patienten mit Schilddrüsenerkrankungen zur Schilddrüsenszintigraphie, in den entsprechenden Quartalen 2007 220 Patienten. Insgesamt zeigte sich eine leichte Abnahme um 6,2% von 3,2% auf 3,0% der Schilddrüsenpatienten pro Arzt, die in den untersuchten Quartalen 2006 und 2007 zur Szintigraphie überwiesen wurden. Diese Veränderung war jedoch statistisch nicht signifikant (0,2 Prozentpunkte, p=0,681).

Bei der Aufteilung in zwei Gruppen zeigte sich bei den Ärzten mit geringer Anzahl an Schilddrüsenpatienten (Gruppe 1) ein leichter Anstieg der Überweisungsrate von 5,7% (0,2 Prozentpunkte), bei den Ärzten mit der höheren Patientenanzahl eine Abnahme der Überweisungsrate um 17,2% (0,5 Prozentpunkte). Beide Vergleiche waren statistisch nicht signifikant. Betont werden sollte, dass die Anzahl der Schilddrüsen-Patienten, die zur Schilddrüsenszintigraphie überwiesen wurden, in beiden Gruppen

sehr niedrig lag. Dies bestätigte sich ebenfalls in der Auswertung in vier Gruppen (auf die weitere Darstellung wurde wegen der geringen Anzahl verzichtet).

N-Pat-SD		N	1.&2. Quartal 2006 MW (SD) in %	1.&2. Quartal 2007 MW (SD) in %	Vergleich MW (SD)	Signifikanz
**SD-Pat**	**Alle TN**	48	3,2 (3,6)	3,0 (4,0)	-0,2 (2,7)	n.s.
	**Gruppe 1**	24	3,5 (4,7)	3,7 (5,4)	0,2 (3,1)	n.s.
	**Gruppe 2**	24	2,9 (2,1)	2,4 (1,7)	-0,5 (2,2)	n.s.

Tab. 32: Vergleich des durchschnittlichen Anteils der Patienten mit Schilddrüsen-erkrankungen der Teilnehmer der Fortbildung Schilddrüse, die zur Schilddrüsenszintigraphie überwiesen wurden (GOP 17320) N-Pat-SD= Anzahl der Patienten mit Schilddrüsenerkrankung, die zur Schilddrüsenszintigraphie überwiesen wurden, N= Anzahl der Teilnehmer, deren Diagnosedaten auswertbar waren, MW= Mittelwert, SD= Standardabweichung, Gruppe 1 (min.-151 Patienten mit Schilddrüsenerkrankung), Gruppe 2 (152-max.).

2.2.5. Überweisung von Patienten mit Schilddrüsenerkrankungen zur Farbduplexsonographie (GOP 33075):

Die Teilnehmer der Fortbildung Schilddrüse überwiesen in den 1. und 2. Quartalen 2006 und 2007 jeweils 232 Patienten mit Schilddrüsenerkrankungen zur Farbduplexsonographie. Der Vergleich des Überweisungsverhaltens zur Farbduplexsonographie der Teilnehmer der Fortbildung Schilddrüse zeigte kaum Veränderung zwischen dem 1. und 2. Quartal 2006 und den entsprechenden Quartalen 2007: die Teilnehmer überwiesen durchschnittlich 2,3% bzw. 2,2% der Schilddrüsenpatienten zur Farbduplexsonographie (Unterschied von 4,3%, 0,1 Prozentpunkte). Bei der Klassierung in zwei Gruppen zeigte sich, dass die Teilnehmer mit einer niedrigeren Anzahl an Schilddrüsenpatienten (Gruppe 1) 2007 durchschnittlich 26,9% weniger Patienten zur Farbduplexsonographie überwiesen als 2006 (0,7 Prozentpunkte, p=0,251) und die Teilnehmer mit der höheren Patientenanzahl durchschnittlich 20,0% mehr Patienten überwiesen (0,4 Prozentpunkte, p=0,201). Ähnliche Ergebnisse ergab auch die weitere Subklassierung in vier Gruppen: bei den beiden Gruppen mit niedrigerer Patientenanzahl (Gruppe 1 und 2) nahm die Überweisungsfrequenz ab, in den beiden Gruppen mit der höheren

Anzahl an Patienten mit Schilddrüsenerkrankungen jedoch zu (Gruppe 3) oder blieb gleich (Gruppe 4). Insgesamt überwiesen 42 Ärzte Schilddrüsenpatienten zur Farbduplexsonographie.

**N-Pat-SD**		**N**	**1.&2. Quartal 2006 MW (SD) in %**	**1.&2. Quartal 2007 MW (SD) in %**	**Vergleich MW (SD)**	**Signifikanz**
**SD-Pat**	**Alle TN**	48	2,3 (2,3)	2,2 (2,2)	0,1 (2,4)	n.s.
	**Gruppe 1**	24	2,6 (3,1)	1,9 (2,5)	-0,7 (2,9)	n.s.
	**Gruppe 2**	24	2,0 (1,0)	2,4 (1,8)	0,4 (41,6)	n.s.
	**Gruppe 1**	12	1,9 (3,3)	1,4 (2,1)	-0,5 (3,5)	n.s.
	**Gruppe 2**	12	3,2 (2,9)	2,3 (2,9)	-0,9 (2,4)	n.s.
	**Gruppe 3**	12	1,8 (0,8)	2,6 (2,2)	0,8 (1,9)	n.s.
	**Gruppe 4**	12	2,2 (1,2)	2,2 (1,4)	0 (1,2)	n.s.

Tab. 33: Vergleich des durchschnittlichen Anteils der Patienten mit Schilddrüsenerkrankungen der Teilnehmer der Fortbildung Schilddrüse, die zur Farbduplexsonographie überwiesen wurden (GOP 33075) N-Pat-SD= Anzahl der Patienten mit Schilddrüsenerkrankung, die zur Farbduplexsonographie überwiesen wurden, N= Anzahl der Teilnehmer, deren Diagnosedaten auswertbar waren, MW= Mittelwert, SD= Standardabweichung¸ Gruppe 1 (min.-151 Patienten mit Schilddrüsenerkrankung), Gruppe 2 (152-max.). Vier Gruppen: Gruppe 1 (min.-101,5), Gruppe 2 (101,6-151), Gruppe 3 (152-269), Gruppe 4 (270-max.).

2.3. Teilnehmer der Fortbildung Diabetes mellitus im Vergleich mit einer Kontrollgruppe

2.3.1. Diagnose Diabetes mellitus und Unterdiagnosen

Die Teilnehmer der Fortbildung Diabetes (N=65) stellten im 1. und 2. Quartal 2006 insgesamt 9478 Mal die Diagnose Diabetes mellitus (ICD-10 E10, E11, E12, E14). Davon war die überwiegende Anzahl Nicht primär insulinabhängiger Diabetes mellitus (Typ 2) und die Subkategorie Nicht näher bezeichneter Diabetes mellitus. Die Teilnehmer der Fortbildung Diabetes stellten im 1. und 2. Quartal 2007 insgesamt 9576 Mal die Diagnose Diabetes mellitus (ICD-10 E10, E11, E12, E14). Davon war die überwiegende Anzahl Nicht primär insulinabhängiger Diabetes mellitus (Typ 2) und die Subkategorie Nicht näher bezeichneter Diabetes mellitus.

Die Ärzte der Kontrollgruppe (N=87) stellten im 1. und 2. Quartal 2006 insgesamt 15923 Mal die Diagnose Diabetes mellitus (ICD-10 E10, E11, E12, E14). Davon war die überwiegende Anzahl Nicht primär insulinabhängiger Diabetes mellitus (Typ 2) und die Subkategorie Nicht näher bezeichneter Diabetes mellitus. Die Ärzte der Kontrollgruppe stellten im 1. und 2. Quartal 2007 insgesamt 16856 Mal die Diagnose Diabetes mellitus (ICD-10 E10, E11, E12, E14). Davon war die überwiegende Anzahl Nicht primär insulinabhängiger Diabetes mellitus (Typ 2) und die Subkategorie Nicht näher bezeichneter Diabetes mellitus.

Bei der statischen Auswertung wurden die Ärzte der Diabetes-Fortbildung und der Kontrollgruppe anhand der Anzahl ihrer Diabetes-Patienten in Gruppen klassifiziert (siehe Abschnitt II. Methoden, 4.5.).

Im Folgenden wurden die ICD-10-Subkategorien E10, E11 und E14, die für die Studie als relevant erachtet wurden, zusammengefasst. Die Teilnehmer der Fortbildung Diabetes stellten im 1. und 2. Quartal 2006 bei insgesamt 9455 Patienten diese Diagnosen; im 1. und 2. Quartal 2007 bei 9554 Patienten. Die Ärzte der Kontrollgruppe (87) stellten im 1. und 2. Quartal 2006 bei insgesamt 7513 Patienten die Diagnosen E10, E11 und E14; im 1. und 2. Quartal 2007 bei 7513 Patienten. Die Diagnose Diabetische Nephropathie (ICD-10 E10/11/14.2) stellten die Teilnehmer der Diabetes-Fortbildung im 1. und 2. Quartal 2006 bei 259 Patienten (Kontrollgruppe: 365 Patienten), im 1. und 2. Quartal 2007 bei 153 Patienten (Kontrollgruppe: 356 Patienten). Die Diagnose Diabetische Retinopathie (ICD-10 E10/11/14.3-) stellten die Fortbildungsteilnehmer im 1. und 2. Quartal 2006 bei 174 Patienten und 2007 bei 226 Patienten (Kontrollgruppe: 1. und 2. Quartal 2006: 329 Patienten und 2007: 359 Patienten). Die Diagnose Diabetische Neuropathie (ICD10 E10/11/14.4-) wurde von den Teilnehmern der Diabetes-Fortbildung im 1. und 2. Quartal 2006 bei 360 und 2007 bei 373 Patienten gestellt. Die Ärzte der Kontrollgruppe stellten die Diagnose dagegen im 1. und 2. Quartal 2006 bei 647 und 2007 bei 686 Patienten. Die Anzahl der Patienten mit der Diagnose Diabetischer Fuß (ICD10 E10/11/14.5-) belief sich bei den Ärzten der Fortbildung im 1. und 2. Quartal 2006 auf 176 (Kontrollgruppe: 216) und im 1. und 2. Quartal 2007 auf 166 (Kontrollgruppe: 225). Die Anzahl der Patienten mit der Diagnose Diabetes mellitus mit multiplen Komplikationen (ICD10

E10/11/14.7-) belief sich bei der Interventionsgruppe im 1. und 2. Quartal 2006 auf 922 und im 1. und 2. Quartal 2007 auf 880, bei der Kontrollgruppe im 1. und 2. Quartal 2006 auf 1261 und im 1. und 2. Quartal 2007 auf 1255.

	**1. & 2. Quartal 2006**				**1. & 2. Quartal 2007**			
	**N** TN/KG	**Anzahl der Patienten** TN/KG	**MW** TN/KG	**SD** TN/KG	**N** TN/KG	**Anzahl der Patienten** TN/KG	**MW** TN/KG	**SD** TN/KG
**Diabetische Nephropathie** (ICD-10 E10/11/14.2)	38/34	259/365	6,82/5,97	19,1/18,5	35/34	153/356	4,37/5,59	6,9/17,5
**Diabetische Retinopathie** (ICD-10 E10/11/14.3-)	37/39	174/329	4,70/4,36	8,5/11,5	34/42	226/359	6,65/4,43	13,6//11,5
**Diabetische Neuropathie** (ICD10 E10/11/14.4-)	42/54	360/647	8,57/6,44	17,0/7,9	43/54	373/686	8,67/6,52	16,8/8,6
**Diabetischer Fuß** (ICD10 E10/11/14.5-)	43/38	176/216	4,09/3,08	8,1/2,7	39/40	166/225	4,26/3,00	8,1/2,7
**Diabetes mellitus mit multiplen Komplikationen** (ICD10 E10/11/14.7-)	39/45	922/1261	23,64/14,69	88,6/42,7	37/49	880/1255	23,78/13,98	86,3/45,1
**Diabetes mellitus allgemein** (ICD-10 E10/11/14)	64/87	9455/7513	147,73/86,36	132,0/68,8	65/86	9554/7547	146,98/87,76	123,0/68,9

Tab. 34: Anzahl der Patienten mit der Diagnose Diabetes mellitus und Subkategorien nach ICD-10, die die Teilnehmer der Fortbildung Diabetes/ der Kontrollgruppe im 1. und 2. Quartal 2006 und 2007 stellten N= Anzahl der Teilnehmer der Fortbildung, die bei Patienten die entsprechende Diagnose stellten, MW= Mittelwert, SD=Standardabweichung, TN= Teilnehmer der Fortbildung, KG= Kontrollgruppe.

Insgesamt zeigte sich eine konstante Diagnosehäufigkeit im Bezug auf die Diagnose Diabetes mellitus. Bei der Klassierung in 2 Gruppen der Teilnehmer der Fortbildung zeigte sich eine statistisch fast signifikante Zunahme der Diagnose Diabetes mellitus in der Gruppe 1, keine Zunahme in der Gruppe 2. Die Ärzte der Fortbildungsgruppe behandelten durchschnittlich 145,5 Patienten (Kontrollgruppe: 87,4 Patienten) mit Diabetes mellitus (2006) und 147,0 Patienten (Kontrollgruppe: 87,8) in den untersuchten Quartalen des Jahres 2007.

		N	1.&2. Quartal 2006 MW (SD)	1.&2. Quartal 2007 MW (SD)	Vergleich MW (SD)	Signifikanz
N-Pat-Diab	KG	86	87,4 (68,6)	87,8 (68,9)	0,4 (11,1)	n.s.
	TN	65	145,5 (132,3)	147,0 (123,0)	1,5 (27,9)	n.s.
	Gruppe 1	33	56,0 (31,2)	59,2 (35,7)	3,2 (10,7)	n.s.
	Gruppe 2	32	237,7 (133,5)	237,5 (115,3)	-0,3 (38,5)	n.s.

Tab. 35: Vergleich der durchschnittlichen Anzahl der Patienten der Teilnehmer der Fortbildung Diabetes/ Ärzte der Kontrollgruppe mit der Diagnose Diabetes mellitus (ICD-10 E10, E11, E14) N-Pat-Diab= Anzahl der Patientin mit der Diagnose Diabetes mellitus, TN= Teilnehmer der Fortbildung Diabetes, KG= Kontrollgruppe, N= Anzahl der Teilnehmer, deren Diagnosedaten auswertbar waren, MW= Mittelwert, SD= Standardabweichung, Gruppe 1: 0-109 Diabetes-Diagnosen; Gruppe 2: 109-max.

Zusammenfassend kann also davon ausgegangen werden, dass die Teilnehmer der Diabetes-Fortbildung und die Ärzte der Kontrollgruppe eine sehr konstante Anzahl an Patienten mit der Diagnose Diabetes mellitus in den jeweils ersten beiden Quartalen 2006 und 2007 behandelten.

2.3.2. Leistungsverhalten der Ärzte:

Auch bei der Abrechnung der Leistungen (Gebührenordnungspositionen=GOP) zeigten sich keine wesentlichen Unterschiede im Vergleich der 1. und 2. Quartale 2006 und 2007. Damit kann auf eine relativ konstante Leistungserbringung sowohl der Ärzte der Diabetes-Fortbildung als auch der Kontrollgruppe geschlossen werden.

	1. & 2. Q. 2006	Häufigkeit TN/KG	Prozent TN/KG	1. & 2. Q. 2007	Häufigkeit TN/KG	Prozent TN/KG
Anzahl der Diabetes-Patienten, bei denen die GOP abgerechnet wurde	02311	44/31	0,5/0,4	02311	21/39	0,2/0,5
	32022	5358/4971	56,7/66,2	32022	5479/5104	57,3/67,6
	32135	1027/468	10,8/6,2	32135	1335/616	13,9/8,1
Gesamtzahl der Diabetes-Patienten		9455/7513	100,0		9554/7547	100,0

Tab. 36: Anzahl der Gebührenordnungspositionen (GOP), die die Teilnehmer der Fortbildung Diabetes/ Ärzte der Kontrollgruppe bei Diabetes-Patienten im 1. und 2. Quartal 2006 und 2007 abrechneten (02311= Ulkus- und Wundentherapie, 32022= Laborpauschale bei Diabetes mellitus, 32135= Test auf Microalbuminurie, Q.= Quartal, TN= Teilnehmer der Fortbildung, KG= Kontrollgruppe.)

2.3.3. Abrechnungsfrequenz der Laborpauschale manifester Diabetes

Die Teilnehmer der Fortbildung Diabetes rechneten im 1. und 2. Quartal 2006 bei 5358 Patienten die Laborpauschale „manifester Diabetes“ (GOP 32022) ab, im 1. und 2. Quartal 2007 bei 5479 Patienten. Die Ärzte der Kontrollgruppe rechneten im 1. und 2. Quartal 2006 8316mal die Laborpauschale „manifester Diabetes“ (GOP 32022) ab, im 1. und 2. Quartal 2007 8551mal. Bei durchschnittlich 90,8 Diabetes-Patienten pro Arzt wurde diese Pauschale von den Fortbildungsteilnehmern im 1. und 2. Quartal 2006 abgerechnet und 2007 bei 92,8 Patienten. Das entspricht einem relativen Anteil von 54,1% der Diabetespatienten 2006 und 54,9% 2007 und somit einer Steigerung von 1,5% (0,8 Prozentpunkte). Bei durchschnittlich 57,6% der Diabetes-Patienten pro Arzt wurde diese Pauschale von den Ärzten der Kontrollgruppe im 1. und 2. Quartal 2006 abgerechnet und 2007 bei 59,1% der Patienten, was einer nicht signifikanten Steigerung von 2,6% (1,5 Prozentpunkte) entspricht.

Nach einer Klassifizierung der Fortbildungsteilnehmer in 2 Gruppen zeigte sich ein ebenfalls nicht statistisch signifikanter, aber etwas größerer Unterschied im Leistungsverhalten der Gruppen der Ärzte, die eine geringere Anzahl an Diabetikern behandelte (2,2% Steigerung versus 0,8%). Nach einer weiteren Unterteilung in 4 Gruppen zeigte sich in den Gruppen 1 und 3 ein erhöhter Anteil an Diabetikern, bei denen diese Pauschale abgerechnet wurde. Die Analyse zeigte eine hohe Standardabweichung und erreichte keine statistische Signifikanz. Bei den Gruppen 2 und 4 konnte im Gegensatz dazu sogar ein leichter Rückgang verzeichnet werden.

N-Pat-Diab		N	1.&2. Quartal 2006 MW (SD) in%	1.&2. Quartal 2007 MW (SD) in %	Vergleich MW (SD)	Signifikanz
**TN**		64	54,1 (27,7)	54,9 (25,9)	0,8 (13,2)	n.s.
**Kontrollgruppe**		87	57,6 (28,2)	59,1 (28,7)	1,5 (15,7)	n.s.
**TN**	**Gruppe 1**	32	49,9 (31,5)	51,0 (29,2)	1,1 (17,2)	n.s.
	**Gruppe 2**	32	58,2 (22,9)	58,7 (22,1)	0,5 (7,8)	n.s.
**TN**	**Gruppe 1**	16	35,4 (29,5)	38,8 (28,4)	3,4 (20,1)	n.s.
	**Gruppe 2**	16	64,5 (27,2)	63,3 (25,1)	-1,2 (13,9)	n.s.
	**Gruppe 3**	16	64,2 (17,3)	67,0(17,8)	2,8 (7,1)	n.s.
	**Gruppe 4**	16	52,2 (26,7)	50,4 (23,3)	-1,8 (7,9)	n.s.

Tab. 37: Vergleich des durchschnittlichen relativen Anteils der Patienten mit Diabetes mellitus der Teilnehmer der Fortbildung Diabetes/ der Ärzte der Kontrollgruppe, bei denen die Laborpauschale „manifester Diabetes mellitus“ (GOP 32022) abgerechnet wurde (N-Pat-Diab= Anzahl der Patientin mit der Diagnose Diabetes mellitus, N= Anzahl der Teilnehmer, deren Diagnosedaten auswertbar waren, MW= Mittelwert, SD= Standardabweichung, TN= Teilnehmer der Fortbildung¸ n.s.= nicht signifikant, Gruppe 1: 0-109 Diabetes-Diagnosen; Gruppe 2: 109-max./ Gruppe 1: 0-55 Diabetes-Diagnosen; Gruppe 2: 56-109; Gruppe 3: 110-205; Gruppe 4: 206-max.)

2.3.4. Frequenz von Mikroalbuminurie-Tests

Die Teilnehmer der Fortbildung Diabetes führten im 1. und 2. Quartal 2006 bei insgesamt 1027 Patienten mit Diabetes mellitus Untersuchungen auf Mikroalbuminurie durch, davon bei insgesamt 78 Patienten mit der Diagnose Diabetische Nephropathie. Im 1. und 2. Quartal 2007 führten sie bei 1335 Patienten mit der Diagnose Diabetes und 51 mit der Diagnose Diabetische Nephropathie Mikroalbuminurie-Tests durch. Insgesamt führten jedoch nur 29 Ärzte diese Untersuchung durch. Die Ärzte der Kontrollgruppe führten im 1. und 2. Quartal 2006 bei insgesamt 468 Patienten mit der Diagnose Diabetes und 35 mit der Diagnose Diabetische Nephropathie einen Test auf Mikroalbuminurie durch. Im 1. und 2. Quartal 2007 führten sie diesen bei insgesamt 616 Patienten mit der Diagnose Diabetes und 54 mit der Diagnose Diabetische Nephropathie. Allerdings führten überhaupt nur

26 Ärzte der Kontrollgruppe 2006 Tests auf Mikroalbuminurie bei Patienten mit Diabetes durch, 2007 waren es 21 Ärzte.

Es zeigte sich zwar eine Zunahme der Anzahl der Diabetes-Patienten, die von den Ärzten der Interventionsgruppe auf Mikroalbuminurie getestet wurden, von durchschnittlich 35,1 Patienten 2006 auf 45,5 Patienten in den Quartalen 2007. Das entspricht einem durchschnittlichen relativen Anteil von 9,8% der Patienten mit Diabetes in den Quartalen 2006 und 10,5% 2007 und somit einer Steigerung um 7,1% (0,7 Prozentpunkte, p=0,563). Auch bei der Kontrollgruppe zeigte sich eine Zunahme des Anteils der Diabetes-Patienten, die auf Mikroalbuminurie getestet wurden, um 10% (0,5 Prozentpunkte) von durchschnittlich 5% der Patienten 2006 auf 5,5% der Patienten in den Quartalen 2007, die jedoch nicht statistisch signifikant war. Außerdem zeigte sich bei beiden Gruppen eine sehr hohe Standardabweichung, die auf das stark unterschiedliche Diagnose- und Leistungsverhalten der Ärzte zurückzuführen ist.

Um diese Unterschiede genauer zu untersuchen wurden die Teilnehmer der Diabetes-Fortbildung anschließend anhand ihres Diagnoseverhaltens in Gruppen unterteilt. Dabei zeigte sich in der Gruppe mit der niedrigeren Diabetikeranzahl eine leichte Abnahme des Anteils an Diabetikern, die auf Mikroalbuminurie getestet wurden, um 2,8%. In der Gruppe mit der höheren Diabetikeranzahl fiel eine Zunahme der Diabetes-Patienten, um durchschnittlich 18,7% auf (1,7 Prozentpunkte, p=0,444). Diese Steigerung erreichte jedoch keine statistische Signifikanz. Nach einer weiteren Klassifizierung in 4 Gruppen zeigte sich in der Gruppe 1 eine geringe Steigerung um 2,8% und eine deutliche Steigerung um 29,6% in Gruppe 4, die jedoch nicht statistisch signifikant waren. In der Gruppe 2 und 3 zeigte sich eine Abnahme von 2,1% in Gruppe 2 und 3,5% in Gruppe 3 im Vergleich vor und nach der Fortbildungsintervention.

Wenn die Diagnose weiter auf Diabetes mellitus mit Nierenkomplikationen eingeschränkt wurde, zeigte sich eine Abnahme des Anteil an Patienten der Interventionsgruppe, bei denen ein Test auf Mikroalbuminurie durchgeführt wurde, von 21,2% auf 13,8% der Patienten, was einer Abnahme von 35,4% entspricht (7,5

Prozentpunkte, p=0,126). In der Kontrollgruppe stellten 41 Ärzte die Diagnose und nur 5 der Ärzte führten bei diesen Patienten auch einen Mikroalbuminurie-Test durch. Das entspricht einem durchschnittlichen Anteil von 8,3% der Patienten mit Diabetischer Nephropathie 2006 und 5,9% 2007, d.h. eine Abnahme um 28,9% (2,4 Prozentpunkte).

Bei einer Unterteilung der Fortbildungsteilnehmer in zwei Gruppen nahm der Anteil in beiden Gruppen auf ähnliche Weise ab, deshalb wurde auf eine weitere Darstellung verzichtet. Allerdings stellten nur 29 Ärzte die Diagnose einer Diabetischen Nephropathie und 14 führten Mikroalbuminurietests durch.

**N-Pat_UEW**		**N**	**1.&2. Quartal 2006 MW (SD) in %**	**1.&2. Quartal 2007 MW (SD) in %**	**Vergleich MW (SD)**	**Signifikanz**
**Diab**	**TN**	65	9,8 (15,3)	10,5 (16,9)	0,7 (9,6)	n.s.
	**KG**	87	5,0 (13,0)	5,5 (14,7)	0,5 (7,1)	n.s.
**Diab-Neph**	**TN**	39	21,2 (35,8)	13,8 (30,1)	-7,5 (29,9)	n.s.
	**KG**	41	8,3 (20,6)	5,9 (15,8)	-2,4 (21,3)	n.s.
**Diab (TN)**	**Gruppe 1**	33	10,5 (16,8)	10,2 (16,6)	-0,3 (4,9)	n.s.
	**Gruppe 2**	32	9,1 (13,8)	10,8 (17,4)	1,7 (12,7)	n.s.
	**Gruppe 1**	17	6,9 (13,9)	6,7 (13,0)	0,2 (4,5)	n.s.
	**Gruppe 2**	16	14,3 (19,1)	13,8 (19,5)	-0,5 (5,6)	n.s.
	**Gruppe 3**	16	9,4 (15,8)	9,2 (15,8)	-0,2 (4,6)	n.s.
	**Gruppe 4**	16	8,8 (12,1)	12,5 (19,3)	3,7 (17,4)	n.s.

Tab. 38: Vergleich des durchschnittlichen Anteils der Patienten mit der Diagnose Diabetes mellitus/ Diabetes mit Nierenkomplikationen der Teilnehmer der Fortbildung Diabetes, bei denen ein Test auf Mikroalbuminurie (GOP 32135) durchgeführt wurde (N-Pat-32135= Anzahl der Patienten, die auf Mikroalbuminurie getestet wurden, Diab= Patienten mit der Diagnose Diabetes mellitus, DiabAug= Patienten mit der Diagnose Diabetes mit Augenkomplikationen, TN= Teilnehmer der Fortbildung, KG= Kontrollgruppe, N= Anzahl der Teilnehmer, deren Diagnosedaten auswertbar waren, MW= Mittelwert, SD= Standardabweichung¸ n.s.= nicht signifikant, Gruppe 1: 0-109 Diabetes-Diagnosen; Gruppe 2: 109-max./ Gruppe 1: 0-55 Diabetes-Diagnosen; Gruppe 2: 56-109; Gruppe 3: 110-205; Gruppe 4: 206-max.)

2.3.5. Therapie des Diabetischen Fußsyndroms

Die Teilnehmer der Fortbildung Diabetes rechneten im 1. und 2. Quartal 2006 insgesamt bei 44 Patienten (Kontrollgruppe: 31) die Gebührenordnungsposition Wunden- bzw. Ulkustherapie (GOP 02311) ab, in den entsprechenden Quartalen 2007 insgesamt bei 21 Patienten (Kontrollgruppe: 39). Von allen Teilnehmer der Fortbildung rechneten nur 15 Ärzte diese Leistung ab, durchschnittlich bei 2,9 (2006) bzw. 1,4 (2007) Patienten. Dies entspricht einem Anteil von 1,6% (2006) und 1,0% (2007) und einer Abnahme von 37,5% (0,6 Prozentpunkte). Insgesamt rechneten nur 17 Ärzte der Kontrollgruppe die Leistung ab (8 davon in beiden Jahren), durchschnittlich bei 3,1 (2006) bzw. 4,5 (2007) Patienten.

Wegen der geringen Anzahl wurde auf eine weitere Analyse mit Gruppenklassierung verzichtet.

**N-Pat-02311**	**N**	**1.&2. Quartal 2006 MW (SD)**	**1.&2. Quartal 2007 MW (SD)**	**Vergleich MW (SD)**	**Signifikanz**
**TN**	15	1,6 (2,0)	1,0 (1,3)	-0,6 (1,9)	n.s.
**KG**	8	3,1 (3,3)	4,5 (4,9)	1,4 (1,9)	n.s.

Tab. 39: Vergleich der durchschnittlichen Anzahl der Patienten der Teilnehmer der Fortbildung Diabetes/ der Ärzte der Kontrollgruppe, bei denen eine Ulkustherapie durchgeführt wurde (GOP 02311) N-Pat-02311= Anzahl der Patienten, bei denen eine Ulkustherapie durchgeführt wurde, TN= Teilnehmer der Fortbildung, KG= Kontrollgruppe, N= Anzahl der Teilnehmer, deren Diagnosedaten auswertbar waren, MW= Mittelwert, SD= Standardabweichung, n.s.= nicht signifikant.

2.3.6. Überweisungsfrequenz zur Funduskopie zum Facharzt für Augenheilkunde

Die teilnehmenden Ärzte der Fortbildung Diabetes überwiesen durchschnittlich 53,4 Patienten im 1. und 2. Quartal 2006 zur Funduskopie zum Facharzt für Augenheilkunde, 56,0 Patienten in den entsprechenden Quartalen 2007. Beim Vergleich des Verhaltens zeigte sich eine gesteigerte Anzahl an Patienten mit allen Diagnosen, die zur Funduskopie überwiesen wurden, die statistisch nicht signifikant war (p=0,06). Die Ärzte der Kontrollgruppe überwiesen durchschnittlich 50,5 Patienten im 1. und 2. Quartal 2006 zur Funduskopie zum Facharzt für Augenheilkunde, 51,5 Patienten in den entsprechenden Quartalen 2007. Beim

Vergleich des Verhaltens zeigte sich eine ziemlich konstante Anzahl an Patienten mit allen Diagnosen, die zur Funduskopie überwiesen wurden (Steigerung von 1, p=0,349).

**N-Pat 06333**	**N**	**1.&2. Quartal 2006 MW (SD)**	**1.&2. Quartal 2007 MW (SD)**	**Vergleich MW (SD)**	**Signifikanz**
**TN**	64	53,4 (34,5)	56,0 (34,3)	2,6 (11,1)	n.s.
**KG**	87	50,5 (35,5)	51,5 (35,2)	1,0 (9,8)	n.s.

Tab. 40: Vergleich der durchschnittlichen Anzahl der Patienten der Teilnehmer der Fortbildung Diabetes/ der Ärzte der Kontrollgruppe, die zur Funduskopie (GOP 06333) zu einem Facharzt für Augenheilkunde überwiesen wurden. N-Pat-06333= Anzahl der Patienten, die zur Funduskopie zu einem Augenarzt überwiesen wurden, TN= Teilnehmer der Fortbildung, KG= Kontrollgruppe, N= Anzahl der Teilnehmer, deren Daten auswertbar waren, MW= Mittelwert, SD= Standardabweichung, n.s.= nicht signifikant.

Bei Patienten mit der Diagnose Diabetes mellitus zeigte sich zwar eine Steigerung der absoluten Anzahl der zur Funduskopie überwiesenen Patienten um 1,0 Patienten pro Arzt (Interventionsgruppe) von 17,9 auf 18,9 Diabetes-Patienten. Das entspricht einem durchschnittlichen relativen Anteil von 11,8% der Patienten mit Diabetes in den Quartalen 2006 und 12,8% 2007 und somit einer Steigerung um 8,5% (1 Prozentpunkt; p=0,172). Bei der Kontrollgruppe zeigte sich ein durchschnittlicher relativer Anteil der zur Funduskopie überwiesenen Patienten von 16,7% der Patienten in den Quartalen 2006 und 16,2% 2007 und somit eine Abnahme um 3% (0,5 Prozentpunkte). Insgesamt überwiesen 80 Ärzte der Kontrollgruppe Diabetiker zu Funduskopie.

Des Weiteren wurde eine Gruppenklassierung in 2 Untergruppen in Hinblick auf die Anzahl der Diabetes-Patienten, die von den Ärzten der Diabetes-Fortbildung insgesamt behandelt wurden, vorgenommen. Dabei zeigte sich in beiden Gruppen eine Zunahme der Überweisungshäufigkeit, jedoch eine etwas höhere in der Gruppe der Ärzte mit der niedrigeren Anzahl an Diabetikern (Gruppe 1, Steigerung um 12,5%, 1,4 Prozentpunkte). In der weiteren Unterklassierung in 4 Gruppen zeigte sich in allen Gruppen eine Steigerung mit der stärksten Veränderung in der Gruppe 1 von 27,7% (2,8 Prozentpunkte, p=0,304). In allen Gruppen wurde keine statistische Signifikanz erreicht.

Bei noch weiterer Einschränkung auf Patienten mit der Diagnose Diabetes mit Augenkomplikationen zeigte sich bei der Interventionsgruppe ebenfalls eine Steigerung von durchschnittlich 3,6 auf 4,9 Patienten pro Arzt. Das bedeutet einen relativen Anteil von 28,4% der Patienten mit diabetischer Retinopathie 2006 und 29,0% 2007 und einer Steigerung von 2,1% (p=0,930). Allerdings stellten nur 38 Fortbildungsteilnehmer die Diagnose eines Diabetes mellitus mit Augenkomplikationen und nur 21 überwiesen diese zur Funduskopie. Bei den Ärzten der Kontrollgruppe zeigte sich eine Zunahme des Anteils um 8,9% (2,8 Prozentpunkte, p=0,657) von durchschnittlich 31,2% (2006) auf 34,0% (2007) Patienten pro Arzt. Nur 19 Ärzte der Kontrollgruppe überwiesen jedoch Patienten mit diabetischer Retinopathie zur Funduskopie in beiden untersuchten Perioden und 43 Ärzte stellten die Diagnose einer diabetischen Retinopathie.

Bei der Gruppenklassierung in 2 Untergruppen nach der Anzahl der Diabetes-Patienten, die von den Ärzten der Fortbildung insgesamt behandelt wurden, zeigte sich eine Zunahme der Überweisungshäufigkeit in Gruppe 1 um 16,5% und eine Abnahme in Gruppe 2 von 3,5%. In der weiteren Unterklassierung in 4 Gruppen zeigte sich in den Gruppen 2 und 3 eine Steigerung mit der stärksten Veränderung in der Gruppe 2 (Steigerung von 150%, p=0,193; Gruppe 3: 11,8%), dagegen eine Abnahme in der Gruppe 1 (50%) und 4 (17,1). In allen Gruppen wurde keine statistische Signifikanz erreicht.

N-Pat_UEW		N	1.&2. Quartal 2006 MW (SD) in %	1.&2. Quartal 2007 MW (SD) in %	Vergleich MW (SD)	Signifikanz
**Diab**	**KG**	87	16,7 (9,9)	16,2 (7,1)	-0,5 (8,8)	n.s.
	**Alle TN**	61	11,8 (5,7)	12,8 (5,2)	1,0 (5,6)	n.s.
	**Gruppe 1**	30	11,2 (6,5)	12,6 (6,3)	1,4 (7,0)	n.s.
	**Gruppe 2**	31	12,3 (4,8)	12,9 (4,0)	0,6 (3,9)	n.s.
	**Gruppe 1**	14	10,1 (7,7)	12,9 (7,8)	2,8 (9,8)	n.s.
	**Gruppe 2**	16	12,2 (5,4)	12,4 (4,9)	0,2 (3,1)	n.s.
	**Gruppe 3**	16	12,9 (5,6)	13,6 (4,7)	0,7 (4,3)	n.s.
	**Gruppe 4**	15	11,7 (3,8)	12,3 (3,1)	0,6 (3,5)	n.s.
**Diab-Aug**	**KG**	43	31,2 (38,6)	34,0 (37,1)	2,8 (41,1)	n.s.
	**TN**	38	28,4 (35,5)	29,0 (32,9)	0,6 (41,1)	n.s.
	**Gruppe 1**	15	20,0 (36,8)	23,3 (32,0)	3,3 (744,2)	n.s.
	**Gruppe 2**	23	33,9 (34,4)	32,7 (33,7)	-1,2 (39,8)	n.s.
	**Gruppe 1**	5	40,0 (54,8)	20,0 (27,4)	-20,0 (57,0)	n.s.
	**Gruppe 2**	10	10,0 (21,1)	25,0 (35,3)	15,0 (33,7)	n.s.
	**Gruppe 3**	12	30,5 (40,7)	34,1 (39,7)	3,6 (53,1)	n.s.
	**Gruppe 4**	11	37,5 (27,3)	31,1 (27,7)	-6,4 (18,0)	n.s.

Tab. 41: Vergleich des durchschnittlichen Anteils der Patienten mit der Diagnose Diabetes mellitus/ Diabetes mit Augenkomplikationen der Teilnehmer der Fortbildung Diabetes/ der Ärzte der Kontrollgruppe, die zur Funduskopie (GOP 06333) zum Facharzt für Augenheilkunde überwiesen wurden. N-Pat-UEW= Anzahl der Patienten, die überwiesen wurden, Diab= Patienten mit der Diagnose Diabetes mellitus, DiabAug= Patienten mit der Diagnose Diabetes mit Augenkomplikationen, TN= Teilnehmer der Fortbildung, KG= Kontrollgruppe, N= Anzahl der Teilnehmer, deren Diagnosedaten auswertbar waren, MW= Mittelwert, SD= Standardabweichung, n.s.= nicht signifikant, Gruppe 1: 0-109 Diabetes-Diagnosen; Gruppe 2: 109-max./ Gruppe 1: 0-55 Diabetes-Diagnosen; Gruppe 2: 56-109; Gruppe 3: 110-205; Gruppe 4: 206-max.

2.3.7. Überweisungsfrequenz zum Diabetologen

Bei dem Vergleich der Anzahl der Diabetes-Patienten, die von den Teilnehmern der Diabetes-Fortbildung an einen Diabetologen überwiesen wurden, zeigte sich eine statistisch signifikante Zunahme von durchschnittlich 7,8% der Diabetes-Patienten in den 1. und 2. Quartalen 2006 auf 10,2% in 2007. Im Vergleich vor und nach der Fortbildung stieg der relative Anteil um 30,8% an (2,4 Prozentpunkte, $p<0,001$). Bei der Kontrollgruppe zeigte sich dagegen eine leichte Abnahme des Anteils der überwiesenen Patienten von 13,4% in den 1. und 2. Quartalen 2006 auf 13,0% im Jahr 2007. Das entspricht einer Abnahme von 3% (0,4 Prozentpunkte, $p=0,546$). Insgesamt fiel auf, dass die Ärzte beider Gruppen einen geringen Anteil der Diabetes-Patienten zum Diabetologen überwiesen.

Bei der Klassierung der Fortbildungsteilnehmer in zwei Gruppen blieb die Zunahme in beiden Gruppen signifikant. Es zeigte sich, dass die Ärzte, die weniger Diabetespatienten behandelten, diese häufiger zum Diabetologen überwiesen als Ärzte mit mehr Patienten. Bei der weiteren Klassierung in vier Gruppen zeigte sich, dass die Ärzte mit einer geringen bis mittleren Anzahl an Diabetes-Patienten diese häufiger zum Diabetologen überwiesen, wobei die Gruppe an Ärzten mit der höchsten Diabetespatientenanzahl diese seltener überwiesen und ihr Verhalten nach der Fortbildung auch nicht signifikant veränderten.

N-Pat-Diab_UEW		N	1.&2. Quartal 2006 MW (SD) in %	1.&2. Quartal 2007 MW (SD) in %	Vergleich MW (SD)	Signifikanz
**TN**		65	7,8 (13,9)	10,2 (14,2)	2,4 (3,8)	<0,001
**KG**		86	13,4 (15,1)	13,0 (12,9)	-0,4 (5,8)	n.s.
**TN**	**Gruppe 1**	33	10,6 (18,5)	13,3 (18,4)	2,7 (4,4)	0,001
	**Gruppe 2**	32	4,8 (5,3)	6,9 (12,7)	2,1 (2,9)	<0,001
**TN**	**Gruppe 1**	17	14,6 (24,7)	18,3 (23,7)	3,7 (5,2)	0,009
	**Gruppe 2**	16	6,3 (6,5)	7,9 (6,3)	1,6 (3,3)	n.s.
	**Gruppe 3**	16	5,6 (5,9)	9,1 (7,9)	3,5 (2,7)	<0,001
	**Gruppe 4**	16	4,1 (4,6)	4,8 (4,3)	0,7 (2,6)	n.s.

Tab. 42: Vergleich der durchschnittlichen Anzahl der Patienten der Teilnehmer der Fortbildung Diabetes/ der Ärzte der Kontrollgruppe mit der Diagnose Diabetes mellitus (ICD-10 E10, E11, E14), die zu einem Diabetologen überwiesen wurden. N-Pat-Diab_UEW= Anzahl der Patientin mit der Diagnose Diabetes mellitus, die zum Diabetologen überwiesen wurden, TN= Teilnehmer der Diabetes Fortbildung, KG= Kontrollgruppe, N= Anzahl der Teilnehmer, deren Daten auswertbar waren, MW= Mittelwert, SD= Standardabweichung, n.s.= nicht signifikant, Gruppe 1: 0-109 Diabetes-Diagnosen; Gruppe 2: 109-max. Vier Gruppen: Gruppe 1: 0-55 Diabetes-Diagnosen; Gruppe 2: 56-109; Gruppe 3: 110-205; Gruppe 4: 206-max.

2.4. Zusammenfassung des Vergleichs des Verhaltens der Teilnehmer der Fortbildung Diabetes mit der Kontrollgruppe

Diagnoseverhalten:

Im Vergleich mit der Interventionsgruppe (Teilnehmer der Fortbildung Diabetes) zeigte sich in der Kontrollgruppe ein sehr ähnliches Diagnoseverhalten bei allerdings höherer Anzahl von Diabetes-Diagnosen. Dabei war die Diagnosehäufigkeit im Bezug auf die Diagnose Diabetes mellitus konstant beim Vergleich der 1. und 2. Quartale 2006 und 2007. Die Interventionsgruppe zeigte im Vergleich eine höhere durchschnittliche Anzahl an behandelten Patienten mit Diabetes mellitus (ca. 147 Patienten mit Diabetes pro Arzt versus 87 Patienten mit Diabetes in der Kontrollgruppe).

Leistungsverhalten:

Abrechnung der Laborpauschale manifester Diabetes: In Bezug auf die Abrechnung der Laborpauschale „manifester Diabetes“ (GOP 32022) zeigte sich ein sehr ähnliches und über die untersuchten Quartale konstantes Leistungsverhalten in beiden Gruppen. Die Ärzte der Interventionsgruppe rechneten diese Pauschale bei 54,1% der Diabetespatienten 2006 und 54,9% 2007 ab. Dies entspricht einer Steigerung von 1,5% (0,8 Prozentpunkte). Nach einer Klassifizierung in 2 Gruppen zeigte sich ein ebenfalls nicht statistisch signifikanter, aber etwas größerer Unterschied im Leistungsverhalten der Gruppen der Ärzte, die eine geringere Anzahl an Diabetikern behandelte (2,2% Steigerung versus 0,8%). Nach einer weiteren Unterteilung in 4 Gruppen zeigte sich in den Gruppen 1 und 3 ein erhöhter Anteil an Diabetikern, bei denen diese Pauschale abgerechnet wurde. Die Analyse zeigte eine hohe Standardabweichung und erreichte keine statistische Signifikanz. Bei den Gruppen 2 und 4 konnte im Gegensatz dazu sogar ein leichter Rückgang verzeichnet werden. Die Ärzte der Kontrollgruppe rechneten bei durchschnittlich 57,6% der Diabetes-Patienten diese Pauschale im 1. und 2. Quartal 2006 ab und 2007 bei 59,1% der Patienten, was einer nicht signifikanten Steigerung von 2,6% (1,5 Prozentpunkte, p=0,384) entspricht. Somit kann auf keine Veränderung des Verhaltens der Ärzte durch die Fortbildungsintervention geschlossen werden.

Frequenz von Mikroalbuminurietests: Bei der Analyse des Leistungsverhaltens zeigte sich bei den Teilnehmern der Fortbildung Diabetes eine Zunahme des relativen Anteils der Mikroalbuminurietests bei Patienten mit Diabetes mellitus von 9,8% der Patienten mit Diabetes in den Quartalen 2006 auf 10,5% 2007 und somit eine Steigerung um 7,1% (0,7 Prozentpunkte, p=0,563). Insgesamt führten jedoch nur 29 Ärzte diese Untersuchung durch. Um diese Unterschiede genauer zu untersuchen wurden die Teilnehmer anschließend anhand ihres Diagnoseverhaltens in Gruppen unterteilt. Dabei zeigte sich in der Gruppe mit der niedrigeren Diabetikeranzahl eine leichte Abnahme des Anteils an Diabetikern, die auf Mikroalbuminurie getestet wurden, um 2,8%. In der Gruppe mit der höheren Diabetikeranzahl fiel eine Zunahme der Diabetes-Patienten, um durchschnittlich 18,7% auf (1,7 Prozentpunkte, p=0,444). Diese Steigerung erreichte jedoch keine statistische Signifikanz. Nach einer weiteren Klassifizierung in 4 Gruppen zeigte sich in der Gruppe 1 eine geringe Steigerung um 2,8% und eine deutliche Steigerung um 29,6% in Gruppe 4, die jedoch nicht statistisch signifikant waren. In den anderen Gruppen zeigte sich eine leichte Abnahme. Bei den Ärzten der Kontrollgruppe fiel ebenfalls eine Zunahme des Anteils der Diabetes-Patienten auf, die auf Mikroalbuminurie getestet wurden, um 10% (0,5 Prozentpunkte) von durchschnittlich 5% der Patienten 2006 auf 5,5% der Patienten in den Quartalen 2007, die jedoch nicht statistisch signifikant war.

Im Vergleich der Quartale 2006 und 2007 zeigte sich zwar sowohl in der Interventions- als auch in der Kontrollgruppe eine Zunahme der Anzahl der Diabetes-Patienten, die auf Mikroalbuminurie getestet wurden, die jedoch nicht statistisch signifikant war. Ebenso zeigte sich eine sehr hohe Standardabweichung, die auf das stark unterschiedliche Diagnose- und Leistungsverhalten der Ärzte beider Gruppen zurückzuführen ist. Beim Vergleich des durchschnittlichen relativen Anteils der Patienten mit Diabetes, die auf Mikroalbuminurie getestet wurden, fiel auf, dass in beiden untersuchten Zeitspannen die Ärzte der Diabetes-Fortbildung durchschnittlich 10% der Diabetiker auf Mikroalbuminurie testeten, doppelt so viele wie die Ärzte der Kontrollgruppe (5%). Es zeigte sich jedoch keine stärkere Veränderung des Verhaltens bei der Interventionsgruppe.

Wenn die Diagnose weiter auf Diabetes mellitus mit Nierenkomplikationen eingeschränkt wurde, zeigte sich in der Interventionsgruppe eine Abnahme des Anteil an Patienten mit dieser Diagnose, bei denen ein Test auf Mikroalbuminurie durchgeführt wurde, von 21,2% auf 13,8% der Patienten, was einer Abnahme von 35,4% entspricht (7,5 Prozentpunkte, p=0,126). Allerdings stellten nur 29 Ärzte die Diagnose einer Diabetischen Nephropathie und 14 führten Mikroalbuminurietests durch. In der Kontrollgruppe stellten nur 41 Ärzte die Diagnose und nur 5 der Ärzte führten bei diesen Patienten auch Mikroalbuminurietests durch. Sie führten sie bei einem durchschnittlichen Anteil von 8,3% der Patienten mit diabetischer Nephropathie 2006 und 5,9% 2007, d.h. eine Abnahme um 28,9% (2,4 Prozentpunkte, p=0,475). Im Vergleich der Gruppen fällt die höhere Anzahl an Ärzten in der Interventionsgruppe auf, die die Untersuchung bei dieser Diagnose durchführte, obwohl in beiden Gruppen etwa die Hälfte der Ärzte die Diagnose eines Diabetes mit Nierenkomplikationen stellte. Allerdings führten die Teilnehmer der Diabetes-Fortbildung in beiden untersuchten Zeiträumen bei einem wesentlich höheren Anteil an Patienten mit diabetischen Nierenkomplikationen einen Mikroalbuminurietest durch als die Ärzte der Kontrollgruppe. Jedoch nahm der Anteil der Patienten in beiden Gruppen 2007 im Vergleich zu 2006 deutlich ab.

Abrechnung einer Wunden- und Ulkustherapie: Die Gebührenordnungsposition Wunden- bzw. Ulkustherapie (GOP 02311) wurde von nur wenigen Ärzte beider Gruppen und insgesamt sehr selten abgerechnet. Die Teilnehmer der Fortbildung Diabetes rechneten im 1. und 2. Quartal 2006 insgesamt bei 44 Patienten die Gebührenordnungsposition Wunden- bzw. Ulkustherapie (GOP 02311) ab, in den entsprechenden Quartalen 2007 insgesamt bei 21 Patienten. Es rechneten nur 15 Ärzte diese Leistung ab. Die Ärzte der Kontrollgruppe rechneten im 1. und 2. Quartal 2006 bei insgesamt 31 Patienten die Gebührenordnungsposition Wunden- bzw. Ulkustherapie (GOP 02311) ab, in den entsprechenden Quartalen 2007 bei 39 Patienten. Von allen Teilnehmern rechneten nur 17 Ärzte diese Leistung ab (8 davon in beiden Jahren), deshalb wurde auf eine weitere Analyse verzichtet.

Überweisungsverhalten:

Überweisung zur Funduskopie zum Facharzt für Augenheilkunde: Die Ärzte der Interventionsgruppe überwiesen durchschnittlich 2007 mehr Diabetes-Patienten zur Funduskopie zum Facharzt für Augenheilkunde als 2006, die Steigerung von 8,5% war jedoch nicht statistisch signifikant (1 Prozentpunkt, p=0,172). Allerdings überwiesen die Teilnehmer der Fortbildung in beiden Quartalen einen geringen relativen Anteil der Patienten mit Diabetes zum Augenarzt (2006: 11,8% und 2007: 12,8%). In der weiteren Gruppenklassierung in 2 bzw. 4 Untergruppen in Hinblick auf die Anzahl der Diabetes-Patienten, die von den Ärzten insgesamt behandelt wurden, zeigte sich in allen Gruppen eine Zunahme der Überweisungshäufigkeit, jedoch die höchste in der Gruppe der Ärzte mit der niedrigeren Anzahl an Diabetikern (2 Gruppen: Gruppe 1: Steigerung um 12,5%, 1,4 Prozentpunkte; 4 Gruppen: Gruppe 1: Steigerung um 27,7%, 2,8 Prozentpunkte). In allen Gruppen wurde keine statistische Signifikanz erreicht. Die Ärzte der Kontrollgruppe überwiesen dagegen weniger Diabetes-Patienten in beiden untersuchten Zeiträumen zur Funduskopie zum Facharzt für Augenheilkunde. Bei Patienten mit der Diagnose Diabetes mellitus zeigte sich ein durchschnittlicher relativer Anteil der zur Funduskopie überwiesenen Patienten von 16,7% der Patienten in den Quartalen 2006 und 16,2% 2007 und somit eine Abnahme um 3% (0,5 Prozentpunkte; p=0,578). Im Vergleich zu den Ärzten der Interventionsgruppe lag jedoch der Anteil der überwiesenen Patienten in beiden Quartalen höher.

Im Bezug auf Patienten mit der Diagnose Diabetes mit Augenkomplikationen zeigte sich in beiden Jahren ein relativ konstante Anzahl zur Funduskopie überwiesener Patienten in der Interventionsgruppe: 28,4% der Patienten mit diabetischer Retinopathie 2006 und 29,0% 2007, was einer Steigerung von 2,1% entspricht (0,6 Prozentpunkte, p=0,930). Allerdings stellten nur 38 Teilnehmer die Diagnose eines Diabetes mellitus mit Augenkomplikationen und nur 21 überwiesen diese zur Funduskopie. Bei der Gruppenklassierung in 2 Untergruppen nach der Anzahl der Diabetes-Patienten, die von den Ärzten insgesamt behandelt wurden, zeigte sich eine Zunahme der Überweisungshäufigkeit in Gruppe 1 um 16,5% und eine Abnahme in Gruppe 2 von 3,5%. In der weiteren Unterklassierung in 4 Gruppen zeigte sich in den

Gruppen 2 und 3 eine Steigerung mit der stärksten Veränderung in der Gruppe 2 (Steigerung von 150%, p=0,193; Gruppe 3: 11,8%), dagegen eine Abnahme in der Gruppe 1 (50%) und 4 (17,1%). In allen Gruppen wurde keine statistische Signifikanz erreicht. Im Vergleich dazu überwiesen die Ärzte der Kontrollgruppe 31,2% der Patienten mit diabetischer Retinopathie in den Quartalen 2006 und 34,0% 2007 zur Funduskopie. Dies entspricht einer Zunahme um 8,9% (2,8 Prozentpunkte, p=0,657). Nur 19 Ärzte der Kontrollgruppe überwiesen jedoch Patienten mit diabetischer Retinopathie zur Funduskopie in beiden untersuchten Perioden und 43 Ärzte stellten die Diagnose einer diabetischen Retinopathie.

Überweisung zum Diabetologen: Bei der Auswertung des Überweisungsverhaltens fiel auf, dass die Ärzte beider Gruppen - der Kontrollgruppe und der Interventionsgruppe - insgesamt einen geringen Anteil der Diabetes-Patienten zum Diabetologen überwiesen: die Ärzte der Kontrollgruppe etwa 13% in beiden Jahren. In der Interventionsgruppe lag der ursprüngliche Anteil zwar mit 7,7% der Diabetes-Patienten, die im 1. und 2. Quartal 2006 zum Diabetologen überwiesen wurden, noch niedriger als in der Kontrollgruppe, allerdings stieg der Anteil statistisch signifikant an auf 10,2% der Patienten (Steigerung von 30,8%, 2,5 Prozentpunkte, p<0,001). Im Vergleich der 1. und 2. Quartale der Jahre 2006 und 2007 nahm der relative Anteil in der Kontrollgruppe dagegen sogar leicht ab (Abnahme um 3%, 0,4 Prozentpunkte, p=0,546). Bei der Klassierung der Ärzte der Fortbildung Diabetes mellitus anhand der Anzahl der von ihnen behandelten Diabetes-Patienten in zwei Gruppen blieb die Zunahme in beiden Gruppen signifikant. Es zeigte sich, dass die Ärzte, die weniger Diabetespatienten behandelten, diese häufiger zum Diabetologen überwiesen als Ärzte mit mehr Patienten. Bei der weiteren Klassierung in vier Gruppen zeigte sich, dass die Ärzte mit einer geringen bis mittleren Anzahl an Diabetes-Patienten diese häufiger zum Diabetologen überwiesen, wobei die Gruppe an Ärzten mit der höchsten Diabetespatientenanzahl diese seltener überwiesen und ihr Verhalten nach der Fortbildung auch nicht signifikant veränderten.

Lernzielparameter	Teilnehmer der Fortbildung Diabetes			Ärzte der Kontrollgruppe			Auffälligkeiten
	1.&2. Q. 2006	1.&2. Q. 2007	Vgl. (%) Sig.	1.&2. Q. 2006	1.&2. Q. 2007	Vgl. (%) Sig.	
**Diagnoseverhalten** (durchschnittl. Anzahl von Pat. mit Diabetes pro Arzt)	145,5	147,0	**+1% p=0,661**	87,4	87,8	**+0,5% p=0,735**	Konstantes Diagnose-verhalten beider Gruppen
**Überweisung zum Diabetologen** (Anteil an allen Pat. mit Diabetes)	7,7%	10,2%	**+30,8% p<0,001**	13,4%	13,0%	**-3% p=0,546**	FB: Ärzte mit weniger Diabetes-Pat. überwiesen häufiger
**Überweisung zur Funduskopie von**							
Pat. mit Diabetes (Anteil an allen Pat. mit Diabetes)	11,8%	12,8%	**+8,5% p=0,172**	16,7%	16,2%	**-3% p=0,578**	FB: Ärzte mit weniger Diabetes-Pat. überwiesen häufiger
Pat. mit diabet. Augenkomplikationen (Anteil an allen Pat. mit diab. Augenkomplikationen)	28,4%	29,0%	**+2,1% p=0,930**	31,2%	34,0%	**+8,9% p=0,657**	
**Abrechnung der Laborpauschale manifester Diabetes** (Anteil an allen Pat.mit Diabetes)	54,1%	54,9%	**+1,5% p=0,625**	57,6%	59,1%	**+2,6% p=0,384**	Relativ konstantes Leistungs-verhalten
**Tests auf Mikroalbuminurie bei**							
Pat. mit Diabetes (Anteil an allen Pat. mit Diabetes)	9,8%	10,5%	**+7,1% p=0,563**	5%	5,5%	**+10% p=0,5**	FB: Ärzte mit mehr Diabetes-Pat. veränderten ihr Verhalten deutlicher
Pat. mit diabet. Nierenkomplikationen (Anteil an allen Pat. mit diab. Nierenkomplikationen)	21,2%	13,8%	**-35,4% p=0,126**	8,3%	5,9%	**-28,9% p=0,475**	beide Gruppen veränderten ihr Verhalten negativ, in KG führten nur 5 Ärzte US durch
**Ulkus- und Wunden-Therapie** (Anzahl von Patienten)	44 Pat.	21 Pat.		31 Pat.	39 Pat.		Beide Gruppen führten sie sehr selten durch

Tab. 43: Vergleich der Verhaltensänderungen der Ärzte der Interventions- und Kontrollgruppe in Bezug auf die Lernzielparameter in den 1. und 2. Quartalen 2006 und 2007. Vgl.= Vergleich, PP= Prozentpunkte, Pat.= Patient/en, FB= Fortbildungsgruppe, KG= Kontrollgruppe, US= Untersuchung

IV. Diskussion und Ausblick

1. Diskussion der Ergebnisse

Level 1 – Zufriedenheit der Lernenden:

In der vorliegenden Studie konnte gezeigt werden, dass ein innovatives, interaktives Fortbildungskonzept mit Falldiskussionen von den Teilnehmern sehr gut bewertet wurde (Evaluationslevel 1: Zufriedenheit der Lernenden nach Kirkpatrick und Curran et al (Kirkpatrick 1994; Curran and Fleet 2005). Besonders die Interaktivität und die Fallorientierung wurden in der Evaluation als ausschlaggebend für die Teilnahme an der Fortbildungsveranstaltung angegeben. Auch die Erwartung an die Fortbildung, gleichzeitig konkrete Entscheidungshilfen für die Praxis und neueste Erkenntnisse aus der Wissenschaft zu erfahren, scheint sich in diesem Fortbildungskonzept aus Expertenvorträgen zu Kernthemen kombiniert mit fallbasierter Kleingruppenarbeit zu erfüllen. Dies zeigte sich besonders in der Abschlussevaluation, bei der die Teilnehmer angaben, dass sie die Fortbildung sehr abwechslungsreich und lehrreich fanden und äußerten, dass die Fälle sie zum Mitdenken aktiviert hätten. Die in der Eingangsevaluation angegebenen hohen Erwartungen an die Fortbildung wurden bei der Mehrheit der Befragten erfüllt. Zudem nahmen viele Ärzte an mehreren Veranstaltungen der Fortbildungsreihe teil.

Die Erkenntnisse der vorliegenden Arbeit decken sich mit denen einer Studie von Gerlach et al, die gezeigt hat, dass „aus Sicht der Befragten didaktisch gelungene Veranstaltungen, kognitive Wissensvermittlung mit unmittelbarem Sachbezug, die Gelegenheit zur intensiven Diskussion, die Vermittlung aktueller Informationen und ein problemorientiertes Vorgehen Attribute guter Fortbildung sind" (Gerlach and Beyer 1999). Die Autoren folgerten, dass es einen ausdrücklichen Bedarf an Angeboten gibt, die „gleichzeitig den interkollegialen Austausch durch die Aktivierung der Teilnehmer fördern, praxisorientierte Themen behandeln, und die außer einem Erkenntnisgewinn auch einen Kompetenzzuwachs ermöglichen". Weitere Studien, die die Zufriedenheit der Teilnehmer mit interaktiven Fortbildungsmethoden evaluierten, kamen ebenfalls zu positiven Ergebnissen (Heale, Davis et al. 1988;

Premi, Shannon et al. 1994; Doucet, Purdy et al. 1998; Garrard, Choudary et al. 2006; Armson, Kinzie et al. 2007). Andere Studien hingegen zeigten, dass Frontalvorträge immer noch das beliebteste Fortbildungsformat für Ärzte darstellen (Kelly and Murray 1994; Stancic, Mullen et al. 2003), obwohl inzwischen zahlreiche Studien deren Ineffektivität bewiesen haben (Grimshaw and Russell 1993; Davis, Thomson et al. 1995; Davis and Taylor-Vaisey 1997; Davis, O'Brien et al. 1999). Thomson O'Brien et al (2001) schreiben, dass es angesichts des lang bekannten Wissens über das selbst bestimmte Lernen von Erwachsenen und der allgemeinen Skepsis, ob formale CME-Angebote wirksam sind, sowie aufgrund der Erkenntnisse der Literaturreviews die Frage gestellt werden muss, warum diese in der Medizin weiter angeboten und zertifiziert werden. Gründe dafür sind sicherlich, dass sie einfach zu organisieren sind und dass die pharmazeutische Industrie bislang viele dieser Fortbildungen veranstaltet oder sponsert. Die Forscher fordern deshalb die Verantwortlichen dazu auf, das System der Zertifizierung und Vergabe von CME-Punkten kritisch zu prüfen, und die Teilnehmer dazu, über ihre Motivation und Ziele nachzudenken. Thomson O'Brien et al betonen aus den Erkenntnissen der Reviews, dass die zukünftige Forschung sich auf interaktive Fortbildungsmaßnahmen konzentrieren und nicht weiter passive Fortbildungsmaßnahmen evaluieren sollte, da deren Ineffektivität bereits gezeigt wurde. Es sei wichtig herauszufinden, welche Qualitätsmerkmale interaktiver Maßnahmen die Effektivität beeinflussen und steigern.

Andere Studien ergaben, dass auch Ärzte selbst passive Fortbildungsformen wie Vorträge für wenig lehrreich halten (Hayes, Allery et al. 1990). Zudem zeichnet sich die Tendenz ab, dass Ärzte, deren Studium und Weiterbildung näher zurückliegt, aktiveren Fortbildungsangeboten (Gruppenarbeit, praktisches Training etc.) als reinen Vorträgen den Vorzug geben (Kelly and Murray 1994; Armson, Kinzie et al. 2007). In der vorliegenden Arbeit schätzten die befragten Ärzte die Interaktivität und Fallorientierung der Fortbildung und das neue Fortbildungsformat als sehr positiv und anregend ein.

Im Gegensatz dazu werteten die Teilnehmer der vorliegenden Studie den Kontakt zu Kollegen als nicht sehr wichtig. Dies stellten auch Kelly und Murray (1994) fest: Nur 9% der von ihnen befragten Ärzten gaben an, dass der soziale Kontakt ein wichtiges

Teilnahmekriterium für Präsenzveranstaltungen darstellt. In anderen Studien zeigte sich jedoch, dass Ärzte den Kontakt zu Kollegen als einen wichtigen Grund für den Besuch einer Fortbildung erachteten (Mamary and Charles 2000; Reddy, Harris et al. 2001).

Level 2 - Wissen:

In der vorliegenden Studie konnte zudem gezeigt werden, dass ein interaktives, fallorientiertes Fortbildungskonzept zu einem signifikanten Wissensanstieg bei Teilnehmern mit einem bereits gut ausgeprägten Vorwissen führt (Evaluationslevel 2: Lernergebnisse nach Kirkpatrick und Curran et al (Kirkpatrick 1994; Curran and Fleet 2005). Dabei lagen die durchschnittlichen Werte im Vorwissenstest sehr hoch. Am niedrigsten war das Vorwissen beim Thema Diabetes mellitus (43,8% richtige Fragen im Vorwissenstest), am höchsten bei arterieller Hypertonie (54,3% richtige Fragen im Vorwissenstest). Zum höchsten relativen Wissenszuwachs kam es beim Thema Diabetes mellitus (62,6% richtige Fragen im Nachwissenstest, 42,9% Steigerung) und Schilddrüse (79,5% richtige Fragen im Nachwissenstest, 42,1% Steigerung), am niedrigsten lag er beim Thema arterielle Hypertonie (76,5% richtige Fragen im Nachwissenstest, 40,7% Steigerung). Der Nachwissenstest wurde wie in der vorliegenden Studie direkt nach der Intervention durchgeführt.

Die Ärzte gaben auch selbst an, viel gelernt zu haben. Außerdem schätzten die Befragten, dass sie ihr Verhalten in der Praxis aufgrund des neu erlangten Wissens ändern würden (subjektive Verhaltensänderung/ Motivation zur Verhaltensänderung).

Im Vergleich zur vorliegenden Arbeit zeigte sich in einer Studie von Qureshi et al (2007), die den Effekt einer interaktiven, fallbasierten Fortbildung auf die Compliance bei antihypertensiver Therapie evaluierte, ein durchschnittlicher Wissenszuwachs von 30,9%. Die Werte im Vorwissenstest lagen jedoch deutlich niedriger als in der vorliegenden Studie: Die Ärzte erreichten 32,3% im Vorwissenstest und 63,2% im Nachwissenstest. In einer weiteren Studie von Doucet et al (1998), die eine Fortbildung im PBL-Format mit einem formalen Fortbildungsformat (Vortrag) verglichen, zeigte sich kein signifikanter Wissenszuwachs. Die Teilnehmer beider Gruppen lösten nur etwa 6% mehr Fragen im Nachwissenstest als im Vorwissenstest.

Doucet et al stellten 40 Multiple-Choice Fragen, von denen die Teilnehmer durchschnittlich 24 im Vorwissenstest und 31 bzw. 33 (Vortrags- bzw. PBL-Gruppe) im Nachwissenstest lösten. Zusätzlich testeten die Wissenschaftler jedoch auch das Wissen und die Fähigkeit zur klinischen Entscheidungsfindung der Ärzte nach drei Monaten mittels Key-Feature-Fällen (Bordage 1995).[2] Dabei stellten sich signifikante Unterschiede heraus: Die PBL-Gruppe löste 25% mehr Key-Feature-Fälle als die Vortrags-Gruppe. Eine Evaluation des Langzeitbehaltens konnte in der vorliegenden Arbeit aufgrund der mangelnden Teilnahme der Ärzte an dem Onlineangebot nicht wie ursprünglich geplant durchgeführt werden. Eine positive Auswirkung auf das Wissen der teilnehmenden Allgemeinärzte durch eine problembasierte Fortbildung mit Kleingruppenarbeit konnten auch Premi et al (1994) zeigen. Sie wiesen einen Wissenszuwachs von 12,1% im Vergleich von Vor- und Nachwissenstest in der Interventionsgruppe nach. Eine Kontrollgruppe aus Ärzten, die an der Fortbildung teilnehmen wollten, jedoch keinen Platz bekamen, zeigte nur einen Wissenszuwachs von 2,8%. Der Unterschied dieser Gruppen war statistisch hochsignifikant. Im Vergleich dazu konnten Chan et al (1999) und Heale et al (1988) keinen effektiven Wissenszuwachs bei den teilnehmenden Ärzten zeigen. Beide Forschergruppen evaluierten in randomisierten Studien PBL-basierte Fortbildungsmaßnahmen bei Allgemeinärzten. Chan et al verglichen eine Internet-Intervention mit einer PBL-basierten E-mail-Fortbildung. Heale et al führten einen Vergleich von PBL in Kleingruppen mit einer formalen Fortbildungsmaßnahme (Diskussion in großer Gruppe und Vortrag) durch.

Patwardhan et al (2006) fanden in ihrer Studie zum Management von Migränepatienten von Allgemeinärzten heraus, dass eine interaktive

---

[2] Key Feature-Fälle sind Fälle, die die Fähigkeit der klinischen Entscheidungsfindung prüfen. Als Key Features werden kritische Entscheidungen definiert, die getroffen werden müssen, um ein klinisches Problem zu lösen. Ein Fall, der solche Key Features enthält, wird Key Feature-Fall genannt. Dieser besteht aus der knappen Darstellung einer klinischen Situation, dem so genannten Stamm, gefolgt von drei bis fünf Fragen, den Key Feature-Fragen. Die Fragen zur klinischen Situation beziehen sich klassischerweise auf die Differentialdiagnosen, auf die diagnostischen Untersuchungen, die zur weiteren Abklärung der Diagnose nötig sind und auf das Management und die therapeutischen Entscheidungen (nach Kopp, V., Möltner, A., Fischer, M.R. (2006). "Key-Feature-Probleme zum Prüfen von prozeduralem Wissen: Ein Praxisleitfaden." GMS Zeitschrift für Medizinische Ausbildung **23**(3).).

Fortbildungsmaßnahme mit Diskussionen und Rollenspiel zu einem statistisch signifikanten kurzfristigen Wissenserwerb führt. Sie stellten dazu den Teilnehmern 20 Fragen zur Prävalenz, Pathophysiologie, Diagnostik und Therapie von Migräne vor und nach der Intervention. Verglichen mit einer Kontrollgruppe von nicht teilnehmenden Ärzten zeigte sich kein Unterschied im Vorwissen. Im Nachwissenstest zeigte sich dann in 16 der 20 Fragen ein signifikanter Wissenszuwachs. Das Abschneiden der Teilnehmer in den einzelnen Items des Fragebogens divergierte allerdings stark. Auch Garrard et al (2006) konnten einen positiven Effekt auf das Wissen der Ärzte im Rahmen einer interaktiven Fortbildungsmaßnahme mit Teamarbeit zum Management von Hepatitis C nachweisen.

Fordis et al (2005), die ein internetbasiertes CME-Angebot mit einem interaktiven Workshop mit Kleingruppenarbeit zum Thema Fettstoffwechselstörungen verglichen, fanden in beiden Gruppen einen signifikanten Wissenszuwachs von durchschnittlich 31% im Vergleich von Vor- und Nachwissenstest. Sie evaluierten zusätzlich nach zwölf Wochen nochmals das Wissen der Teilnehmer mit demselben Test aus Multiple-Choice-Fragen und Fallvignetten, der aus 39 Items bestand. Dabei zeigte sich ein weiterer Wissenszuwachs von 5,4%, der ebenfalls statistisch signifikant war.

Zusammenfassend kann gesagt werden, dass das Ergebnis der vorliegenden Studie bezüglich des Wissens der Teilnehmer mit den Erkenntnissen früherer Studien zu interaktiven Fortbildungsmaßnahmen, insbesondere solchen mit Team-basierten und Problem-basierten Lernstrategien, weitgehend übereinstimmt. Im Vergleich zu formalen Fortbildungsmaßnahmen wie reinen Vorträgen, die einen geringen oder fehlenden Wissenszuwachs gezeigt haben, zeigt sich hier ein eindeutiger Vorteil interaktiver Interventionen.

Level 3 - Verhaltensänderung:

Im dritten Schritt wurde evaluiert, ob dieser Wissenserwerb zu einer objektivierbaren Verhaltensänderung geführt hat (Evaluationslevel 3: Verhaltensverbesserung nach Kirkpatrick und Curran et al (Kirkpatrick 1994; Curran and Fleet 2005). Es gibt zahlreiche Studien zur Effektivität verschiedener Fortbildungsmaßnahmen zur Verhaltensänderung bei Ärzten. Diese untersuchten verschiedene Dimensionen des

Verhaltens von Ärzten, unter anderem zur Verschreibung von Medikamenten (Gullion, Tschann et al. 1987; Rossi and Every 1997; Montgomery, Fahey et al. 2000), Betreuung bei chronischen Krankheiten (Dickinson, Warshaw et al. 1981; Rogers, Haring et al. 1982; Barnett, Winickoff et al. 1983; White, Albanese et al. 1985; Heale, Davis et al. 1988; Jennett, Wilson et al. 1989; Clark, Gong et al. 1998), Prävention und Screening (Jennett, Laxdal et al. 1988; Dietrich, O'Connor et al. 1992; Browner, Baron et al. 1994; Boissel, Collet et al. 1995; Fordis, King et al. 2005), leitliniengerechter Behandlung (Fordis, King et al. 2005), Beratung und Kommunikationsfähigkeiten (Maiman, Becker et al. 1988; Wilson 1992; Levinson and Roter 1993; Roter, Hall et al. 1995; Ockene, Hebert et al. 1996). Die Methoden, die Verhaltensänderungen zu erfassen, waren ebenso vielfältig: Auswertung von Patientenakten (White, Albanese et al. 1985; Jennett, Laxdal et al. 1988; Sulmasy, Geller et al. 1992; Browner, Baron et al. 1994; Parker, Leggett-Frazier et al. 1995; Sulmasy, Song et al. 1996; Ward and Sanson-Fisher 1996; Fordis, King et al. 2005) oder Rezepten bzw. Verschreibungsdaten von Medikamenten (Angunawela, Diwan et al. 1991; Bexell, Lwando et al. 1996; Hadiyono, Suryawati et al. 1996), bzw. Kontrollen von organisatorischen Abläufen (Garrard, Choudary et al. 2006), Auswertung von verschriebenen Untersuchungen (Perera, LoGerfo et al. 1983; Dietrich, O'Connor et al. 1992; Boissel, Collet et al. 1995). In anderen Untersuchungen wurden Videoaufnahmen ausgewertet (Roter, Hall et al. 1995; Smith, Shaw et al. 1995), Patientenbefragungen durchgeführt (Kottke, Brekke et al. 1989; Strecher, O'Malley et al. 1991; Pekarik 1994; Roter, Hall et al. 1995; Ockene, Hebert et al. 1996; Sulmasy, Song et al. 1996; Ward and Sanson-Fisher 1996; Dolan, Ng et al. 1997) oder mit Hilfe von standardisierten Patienten das Verhalten der Ärzte evaluiert (Wood, Littlefield et al. 1989; Carney, Dietrich et al. 1995; Roter, Hall et al. 1995).

Allerdings berufen sich einige Studien auch nur auf die Selbstaussagen der Ärzte zur eigenen Verhaltensänderung nach einer Fortbildung (Saturno 1995; Zwar, Gordon et al. 1995; Francke, Luiken et al. 1997) oder evaluierten die Ergebnisse nicht in klinischen Situationen (Terry, Wang et al. 1981; Stross 1983; Roter, Cole et al. 1990; Campbell, Fletcher et al. 1991; Quirk, Ockene et al. 1991; Dunn, Niday et al. 1992; Ockene, Hebert et al. 1996; Carlsson, Gravgaard et al. 1998; Langewitz, Eich et al.

1998)). Als Ärzte nach dem Besuch einer Fortbildungsveranstaltung gefragt wurden, ob sie vorhatten, ihr Verhalten zu ändern und welche speziellen Dinge sie verändern wollten, zeigte sich, dass Ärzte, die dies bejahten, eher ihr Verhalten änderten als die Ärzte, die dies verneinten. Allerdings zeigte sich kein Unterschied zwischen einer Gruppe an Ärzten, die zusätzlich in der Fortbildung über die Barrieren, die einer Verhaltensänderung entgegenstehen, aufgeklärt wurden, und Ärzten, die nur die Fortbildung besuchten (Mazmanian, Daffron et al. 1998). Diese so genannte „Commitment-to-change“-Strategie scheint ebenfalls einen Effekt zu haben.

Aufgrund der weiten Verbreitung formaler Fortbildungsmaßnahmen untersuchten mehrere Forscher in Metaanalysen die Effektivität dieser Fortbildungsformate in Bezug auf eine objektivierbare Verhaltensänderung der Ärzte oder eine verbesserte Patientenversorgung (Davis, O'Brien et al. 1999; Thomson O'Brien, Freemantle et al. 2001). Im aktuellsten Review von Thomson O’Brien et al wurden 32 Studien mit 36 Vergleichen von Interventionen identifiziert. In sieben Studien zu passiven Fortbildungsveranstaltungen zeigte sich kein wesentlicher Effekt auf das Verhalten und die Patientenversorgung der Ärzte (Wirtschafter, Sumners et al. 1986; Angunawela, Diwan et al. 1991; Sulmasy, Geller et al. 1992; Browner, Baron et al. 1994; Boissel, Collet et al. 1995; Parker, Leggett-Frazier et al. 1995; Dolan, Ng et al. 1997). Im Gegensatz dazu fanden die Wissenschaftler bei sechs von zehn Untersuchungen von interaktiven Fortbildungsmaßnahmen einen mittelmäßigen bis mittelhohen, statistisch signifikanten Effekt (Jennett, Laxdal et al. 1988; Wood, Littlefield et al. 1989; Kimberlin, Berardo et al. 1993; Smith, Shaw et al. 1995; Hadiyono, Suryawati et al. 1996) und in vier einen geringen Effekt (Heale, Davis et al. 1988; Dietrich, O'Connor et al. 1992). Interventionen, die passive und aktive Fortbildungsformen kombinierten, zeigten in 12 Studien einen mittelmäßigen bis mittelhohen Effekt, der bei elf signifikant war (Perera, LoGerfo et al. 1983; White, Albanese et al. 1985; Mazzuca, Barger et al. 1987; Maiman, Becker et al. 1988; Kottke, Brekke et al. 1989; Strecher, O'Malley et al. 1991; Sulmasy, Geller et al. 1992; Wilson 1992; Roter, Hall et al. 1995; Ward and Sanson-Fisher 1996; Messmer, Jones et al. 1998), und einen geringen in sechs Vergleichen, von denen einer signifikant war (Levinson and Roter 1993; Pekarik 1994; Westphal, Taddei et al. 1995; Bexell,

Lwando et al. 1996; Ockene, Hebert et al. 1996; Sulmasy, Song et al. 1996; Jones, Carr et al. 1998). Thomson O'Brien et al (2001) schließen daraus, dass ein Effekt von passiven Fortbildungsveranstaltungen auf das Verhalten der Ärzte und die Patientenversorgung unwahrscheinlich ist, interaktive Workshop diesen jedoch bewirken können. Zum gleichen Ergebnis kommen auch Davis et al (1998) in ihrer Metaanalyse zur Wirkung von Fortbildungsmaßnahmen auf das ärztliche Verhalten und die Patientenversorgung. Mehr als zwei Drittel der Studien (70%) zeigten eine Veränderung im Verhalten der Ärzte, während fast die Hälfte (48%) der Interventionen einen Effekt auf die Patientenversorgung hatte. Zu diesem Schluss kommen auch Bloom (2005) und Oxman et al (1995) in ihren Metaanalysen zur Effektivität verschiedener Fortbildungsformate.

Viele der Studien zur Verhaltensänderung zeigten außerdem, dass die Qualität des Verhaltens vor einer Fortbildungsmaßnahme gering war (Jennett, Laxdal et al. 1988; Angunawela, Diwan et al. 1991; Sulmasy, Geller et al. 1992; Wilson 1992; Browner, Baron et al. 1994; Parker, Leggett-Frazier et al. 1995; Smith, Shaw et al. 1995; Westphal, Taddei et al. 1995; Hadiyono, Suryawati et al. 1996; Sulmasy, Song et al. 1996; Ward and Sanson-Fisher 1996). Mittelmäßige Qualität des Verhaltens zeigten einige Studien (White, Albanese et al. 1985; Dietrich, O'Connor et al. 1992; Dolan, Ng et al. 1997; Clark, Gong et al. 1998; Jones, Carr et al. 1998) und nur vereinzelt fand sich eine hohe Qualität (Wood, Littlefield et al. 1989; Sulmasy, Geller et al. 1992). In der vorliegenden Studie zeigten sowohl die Teilnehmer der Fortbildungsveranstaltungen als auch die Kontrollgruppe im Bereich Diabetes mellitus im Durchschnitt ein wenig leitliniengerechtes Verhalten in Bezug auf die Diagnostik und Betreuung von Patienten mit den Erkrankungen Diabetes mellitus, Schilddrüsenerkrankungen und sekundärer Hypertonie. So fielen große Unterschiede im Verhalten der Ärzte auf, was auf ein sehr unterschiedliches Vorwissen und einen inhomogenen Erfahrungsschatz schließen lässt. Dies kann zumindest teilweise durch die unterschiedlich lange Berufserfahrung der Ärzte oder ihre unterschiedliche Spezialisierung erklärt werden. Das Alter der teilnehmenden Ärzte lag in der vorliegenden Studie zwischen 27 - 68 Jahren, im Durchschnitt bei 48,4 Jahren. 60,3% der Teilnehmer war Allgemeinärzte und 22,7% Internisten sowie 12,6% Arzt/Ärztin in

Weiterbildung. Die teilnehmenden Ärzte hatten ihren Facharzt seit durchschnittlich 13,0 (0-38) Jahren. Allerdings haben auch Studien gezeigt, dass gerade Ärzte mit langer Berufserfahrung in Gefahr sind, eine schlechtere medizinische Versorgung zu leisten (Choudhry, Fletcher et al. 2005). Eine genauere Auswertung in Bezug auf die Berufserfahrung und das Alter der teilnehmenden Ärzte war jedoch aus Datenschutzgründen nicht möglich, da nur anonymisierte Daten der Kassenärztlichen Vereinigung zur Verfügung standen.

Fortbildungsthema Diabetes mellitus

In der vorliegenden Studie konnte gezeigt werden, dass die teilnehmenden Ärzte der Fortbildung zum Thema Diabetes ihr Verhalten in den Bereichen der Grundversorgung von Diabetikern (Mikroalbuminurietests, Überweisung zur Funduskopie und zum Diabetologen) bis auf die Wundversorgung und die Abrechnung der Laborpauschale für manifesten Diabetes mellitus veränderten. Allerdings zeigte sich insgesamt ein heterogenes Verhalten der Ärzte sowohl vor als auch nach der Fortbildungsintervention, was sich in der Analyse in einer sehr hohen Standardabweichung niederschlug. Dies zeigte sich auch bei den Ärzten der Kontrollgruppe. Insgesamt konnte jedoch ein konstantes Diagnoseverhalten sowohl der Ärzte der Interventionsgruppe (Teilnehmer der Diabetes-Fortbildung) als auch der Kontrollgruppe in den untersuchten Zeiträumen (jeweils 1. und 2. Quartal 2006 und 2007) beobachtet werden. Interessanterweise behandelten die Ärzte der Interventionsgruppe durchschnittlich 40% mehr Patienten mit Diabetes mellitus als die Ärzte der Kontrollgruppe, allerdings zeigten sich auch große Unterschiede in der Anzahl der behandelten Patienten innerhalb der Gruppen (Interventionsgruppe: min. 1, max. ca. 400 Diabetes-Patienten; Kontrollgruppe: min. 1, max. ca. 800 Patienten).

Eine statistisch signifikante Verhaltensänderung der Fortbildungsteilnehmer zeigte sich in Bezug auf die Überweisungsfrequenz von Patienten mit Diabetes mellitus zum Spezialisten (Diabetologen). Die Teilnehmer überwiesen nach der Fortbildung 30,8% mehr Diabetes-Patienten zum Spezialisten. Eine deutliche Steigerung der Überweisung zum Facharzt für Augenheilkunde zur Funduskopie von 8,5% konnte ebenfalls gezeigt werden; diese Zunahme erreichte jedoch keine statistische Signifikanz. Im Vergleich

dazu nahm in diesen beiden untersuchten Bereichen die Überweisungsfrequenz der Ärzte der Kontrollgruppe ab, so dass diese Verhaltensänderung durch die Fortbildungsintervention hervorgerufen sein könnte. In der Subuntersuchung in Bezug auf Patienten mit bekannten Augenkomplikationen zeigte sich allerdings auch in der Kontrollgruppe eine Steigerung des Überweisungsverhaltens zur Funduskopie.

Bei der Analyse des Leistungsverhaltens der Ärzte der Fortbildung Diabetes zeigte sich eine Zunahme der durchgeführten Mikroalbuminurietests pro Diabetes-Patienten um 7,1% nach der Fortbildungsintervention. Eine ähnliche Steigerung konnte jedoch auch bei der Kontrollgruppe nachgewiesen werden. Allerdings fiel auf, dass die Fortbildungsteilnehmer schon vor der Fortbildung bei etwa doppelt soviel Diabetes-Patienten eine Untersuchung auf Mikroalbuminurie durchführten. In der Subuntersuchung des Anteils der Patienten mit diabetischen Nierenkomplikationen, bei denen Mikroalbuminurietests abgerechnet wurden, zeigte sich im Gegensatz zur Untersuchung bei allen Diabetes-Patienten sowohl in der Interventions- als auch Kontrollgruppe eine starke Abnahme.

Ingesamt konnte jedoch nachgewiesen werden, dass die Grundversorgung der Diabetespatienten bisher nicht vollständig den Leitlinien entspricht. So wird nur ein sehr geringer Teil der Patienten mit Diabetes mellitus zum Diabetologen oder zur Funduskopie zum Facharzt für Augenheilkunde überwiesen. Auch die selbst erbrachten Leistungen wie beispielsweise Mikroalbuminurietests bei Diabetikern wurden durchschnittlich selten und nur von einem Teil der teilnehmenden Ärzte durchgeführt. Allerdings ist es möglich, dass die Patienten teilweise auch ohne Überweisung selbstständig für diese Untersuchungen zu den entsprechenden Fachärzten gehen. Davon kann jedoch bei einer meist geringen Compliance bei Diabetikern (Möbes 2003; Cramer 2004) nicht unbedingt ausgegangen werden. Außerdem kann es sein, dass die Ärzte die Patienten zwar zum Facharzt überwiesen, diese sich jedoch dort nicht vorstellten. In manchen Bereichen zeigte sich außerdem, dass Ärzte, die im Durchschnitt eine geringere Anzahl an Diabetikern behandeln, anscheinend stärker ihr Verhalten ändern als Ärzte, die viele Patienten mit Diabetes mellitus behandeln. Dies fiel sowohl bei der Überweisungsfrequenz zum Diabetologen als auch zum Augenarzt zur Funduskopie auf. Somit kann darauf geschlossen werden,

dass Ärzte mit mehr Erfahrung weniger von einer Fortbildungsintervention profitieren als Ärzte mit weniger Erfahrung. Ein bedeutsamer Grund dafür könnte ein Decken-Effekt sein. Dies bedeutet, dass die Veränderungen bei erfahrenen, kompetenten Ärzten weniger ausgeprägt sind als bei weniger erfahrenen Kollegen, da zweitere relativ gesehen mehr dazulernen und daraufhin auch ihr Verhalten ändern können. Bei der Überweisung von Diabetikern zum Spezialisten ist es außerdem möglich, dass die Ärzte mit vielen Diabetes-Patienten auf ihre eigene Kompetenz vertrauen und die Patienten deshalb seltener zum Spezialisten überweisen. Bei der Analyse der Mikroalbuminurietest-Frequenz bei Diabetes-Patienten zeigte sich allerdings der umgekehrte Fall. Dort konnte nachgewiesen werden, dass die Ärzte der Interventionsgruppe mit der höchsten Anzahl an Diabetes-Patienten nach der Fortbildung bei etwa 30% mehr Patienten Mikroalbuminurietests durchführten. Eine Begründung dafür könnte wiederum in der wahrscheinlich höheren Kompetenz dieser Ärzte liegen, die diese Basisuntersuchungen selbst durchführen, anstatt die Patienten dafür gleich zum Spezialisten zu überweisen.

Fortbildungsthema Schilddrüse

Beim Fortbildungsthema Schilddrüse zeigten sich bei der Auswertung der Daten keine wesentlichen Verhaltensänderungen der Teilnehmer der Fortbildung. Die Ärzte mit weniger Patienten mit Schilddrüsenerkrankungen führten sogar bei diesen Patienten nach der Fortbildung seltener eine Schilddrüsensonographie als vorher durch. Die Gruppe der Ärzte mit einer höheren Patientenanzahl zeigte dagegen keine Verhaltensänderung. Je mehr Patienten mit Schilddrüsenerkrankungen die Ärzte hatten, desto höher lag jedoch der Anteil der bei diesen durchgeführten Schilddrüsensonographien. Bei der Analyse des Überweisungsverhaltens fiel auf, dass die teilnehmenden Ärzte Patienten mit Schilddrüsenerkrankungen im Vergleich der Quartale vor und nach der Fortbildung seltener zum Schilddrüsensonographie zu anderen Fachärzten überwiesen. Bei der Analyse in Gruppen nach der Anzahl der behandelten Schilddrüsenpatienten zeigte sich, dass die Ärzte mit weniger Patienten diese deutlich seltener zur Sonographie überwiesen, die Ärzte mit vielen Patienten ihr Überweisungsverhalten aber nicht änderten. Die Ärzte mit weniger

Schilddrüsenpatienten überwiesen diese etwa doppelt so häufig zur Schilddrüsensonographie wie die Ärzte mit einer hohen Patientenanzahl. Beide Vergleiche waren statistisch nicht signifikant. Die Abnahme der Untersuchungsfrequenz kann eventuell teilweise durch eine Qualitätsoffensive der Kassenärztlichen Vereinigung Bayern im Bereich Sonographie erklärt werden, die seit dem 1. August 2006 läuft (www.kvb.de). Die Qualitätsoffensive beschränkte sich zwar zunächst nur auf die Sonographie des Abdomens, könnte jedoch trotzdem Auswirkungen auf die Sonographie anderer Organsysteme wie die Schilddrüse haben. Bei dem Qualitätsprogramm werden sowohl die fachlichen Fähigkeiten sonographisch tätiger Ärzte als auch die Qualität ihrer Geräte geprüft. Zur Selbstüberprüfung der fachlichen Kenntnisse hat die KVB gemeinsam mit Experten und Vertretern von Haus- und Fachärzten ein webbasiertes Tutorial entwickelt. Rund 8000 Haus- und Fachärzte können sich auf freiwilliger Basis zertifizieren lassen und Fortbildungspunkte erwerben. Die Mindestanforderungen an die Gerätequalität entsprechen im Wesentlichen denen der Stufe 1 der DEGUM (Deutschen Gesellschaft für Ultraschall in der Medizin). Voraussetzung sind mindestens 256 Graustufen, womit die Anforderungen an die weiteren Qualitätsparameter wie beispielsweise Dynamik, Prozesskanäle, Bildrate oder Sendeleistung abgedeckt sind. Ab dem 1. Januar 2008 erhalten alle sonographisch tätigen Ärzte, die über ein entsprechendes Zertifikat der KVB verfügen, für jede abdominelle Sonographie eine Zusatzvergütung in Höhe von 4,25 Euro. Alle Ärzte ohne Zertifikat erhalten für diese Leistung künftig nur noch einen Punktwert in Höhe von fünfzig Prozent des Auszahlungspunktwertes der Fachgruppe. Eine Ausweitung der Qualitätssicherung im Bereich der Sonographie auf andere Organsysteme ist geplant. Es wäre also gut möglich, dass Ärzte, die bisher nur ein Sonographiegerät besaßen, das den oben genannten Qualitätsansprüchen nicht genügte, Sonographieuntersuchungen seit Einführung des Sonographieprogramms insgesamt seltener durchführen und dies auch die Schilddrüsensonographien einschließt. Eine andere Änderung des Abrechnungs- oder Vergütungssystems in den Jahren 2006 und 2007 wurde jedoch nicht durchgeführt. Im Prinzip könnte die Verhaltensänderung aber auch mit einer besseren Einschätzung der eigenen Kompetenz in der Schilddrüsensonographie zusammenhängen. Allerdings ist diese Erklärung nicht wahrscheinlich, da die Ärzte die Patienten auch seltener zur

Sonographie an andere Fachärzte überwiesen. Es könnte jedoch sein, dass die Ärzte seltener Schilddrüsensonographien durchführten, da sie die Indikation nach der Fortbildung seltener stellten. Dies könnte in späteren Studien mittels einer Befragung der Ärzte herausgefunden werden.

Bei der Überweisung von Schilddrüsenpatienten zur Schilddrüsenszintigraphie zeigte sich ein relativ konstantes Verhalten der Ärzte vor und nach der Fortbildungsintervention. Bei der Gruppenklassierung fiel jedoch auf, dass die Ärzte mit weniger Patienten diese etwas häufiger zur Szintigraphie überwiesen, die Ärzte mit einer höheren Patientenanzahl deutlich seltener. Betont werden sollte, dass die Anzahl der Schilddrüsen-Patienten, die zur Schilddrüsenszintigraphie überwiesen wurden, insgesamt sehr niedrig lag. Das Verhalten der Ärzte mit der höheren Anzahl an Schilddrüsenpatienten könnte darin begründet sein, dass diese die Indikation nach der Fortbildung enger stellten und die Patienten deshalb seltener zur Schilddrüsenszintigraphie überwiesen.

Der Vergleich des Überweisungsverhaltens zur Farbduplexsonographie der Teilnehmer der Fortbildung Schilddrüse zeigte insgesamt ebenfalls kaum Veränderung zwischen dem 1. und 2. Quartal 2006 und den entsprechenden Quartalen 2007. Bei dem Vergleich der zwei Gruppen von Ärzten anhand ihrer Patientenanzahl konnte jedoch nachwiesen werden, dass die Ärzte mit weniger Patienten diese nach der Intervention seltener überwiesen, die Teilnehmer mit einer höheren Patientenzahl aber häufiger. Eine schlüssige Erklärung dieses Verhaltens konnte nicht gefunden werden.

Mögliche Barrieren, das Verhalten zu ändern, wurden unter anderem von Wensing (1998) beschrieben. Sie können beispielsweise in der Organisation der Praxisstruktur, Zeitmangel, negativen finanziellen Auswirkungen, negativen Einstellungen unter Kollegen oder der abwehrenden Haltung von Patienten liegen. So wäre es möglich, dass manche Lernziele nicht mit der Ansicht der Ärzte zusammenpassen, nur ungenügend diskutiert und deshalb auch nicht angenommen wurden. Donner-Banzhoff (2001) schreibt dazu: „Von großer Bedeutung ist, ob mit einer Innovation konfrontierte Ärzte diese als relevant, hilfreich und praktikabel für ihren Arbeitsbereich ansehen; hier bestehen besonders gegen von außen eingebrachte Anforderungen vielfach Vorbehalte, die zum Teil nur verdeckt geäußert werden

können.“ Er führt außerdem an, dass der permanente Zwang zur Umsetzung administrativer Erfordernisse (Gebührenordnungen, Wirtschaftlichkeitsgebot, Diagnosen-Kodierung) wenig Spielraum für Lernen und Neuerungen lasse und eine gewisse Hierarchie aus Anforderungen und Bedürfnissen berücksichtigt werden müsse. Allerdings muss einschränkend gesagt werden, dass in der vorliegenden Arbeit keine komplexen Tätigkeiten (z.B. Kommunikationsfähigkeit) oder sehr zeitaufwendige Untersuchungsmethoden evaluiert wurden. Eine wichtige Rolle spielen sicher auch die finanziellen Umstände. So werden immer mehr Untersuchungen in Pauschalen abgerechnet und deren Qualität nicht bewertet.

Fortbildungsthema Sekundäre Hypertonie

Beim Thema Sekundäre Hypertonie zeigte sich, dass die Teilnehmer insgesamt sehr wenige Patienten mit dieser Erkrankung behandeln bzw. die Diagnose außerordentlich selten stellen. Wenn davon ausgegangen wird, dass beispielsweise bis zu 10% der Patienten mit einer arteriellen Hypertonie einen primären Hyperaldosteronismus haben (Young 2003), müsste die Diagnoserate weit höher liegen. Auch der subklinische Hyperkortisolismus tritt weitaus häufiger auf, als die Diagnoserate der sekundären Hypertonie aufgrund endokriner Erkrankungen der Teilnehmer schließen lassen würde. Diese Fakten wurden in der Fortbildung gezeigt und diskutiert. Auch die entsprechenden Untersuchungen (Bestimmung des Aldosteron-Renin-Quotienten, der Plasma-Metanephrine oder der Test auf Katecholamine im Urin) wurden von den Teilnehmern so selten durchgeführt, dass eine weitere statistische Auswertung nicht sinnvoll erschien. Entweder werden also diese Patienten sofort zum Facharzt oder in die Klinik überwiesen oder diagnostisch nicht weiter abgeklärt. Aus diesen Erkenntnissen könnte der Schluss gezogen werden, dass die Fortbildungslernziele nicht praxisnah genug formuliert waren. Deshalb sollten die Lernziele zur arteriellen Hypertonie entsprechend angepasst werden und beispielsweise der Fokus eher auf die Erkennung der Patienten, bei denen eine sekundäre Hypertonie ausgeschlossen werden sollte, als auf die weitere Diagnostik der sekundären Hypertonie gelegt werden. Um eine den realistischen Lernzielen angepasste Fortbildung entwickeln zu können, müssten also der Wissensstand und die gegenwärtige Arbeitsweise der Ärzte bekannt

sein, um auf derselben Stufe eine Wissens- und Verhaltensverbesserung zu bewirken. Es ergibt beispielsweise keinen Sinn, die technische Diagnostik und die weitere Therapie zu vermitteln, wenn die grundlegende klinische Diagnostik unklar ist. Allerdings ist das Diagnoseverhalten der Ärzte anhand der Daten der Kassenärztlichen Vereinigung auch nur eingeschränkt analysierbar, da nur die Diagnose bei einem bestimmten Patienten bekannt ist und nicht, warum der Arzt diese gestellt hat oder ob er sie von einem anderen Arzt übernommen hat.

2. Stärken und Limitationen der vorliegenden Studie

Das in dieser Arbeit vorgestellte Fortbildungskonzept wurde aus den Erkenntnissen bisheriger Studien zum Lernverhalten Erwachsener (Boud 1987; Collins 2004) und (Medizin-) Studenten (Bruner 1961; Hunt 1971; Barrows 1980; Norman and Schmidt 1992; Barrows 1994; Birmingham 2002; Michaelsen 2002), sowie insbesondere von Ärzten (Slotnick 1999; Collins 2004) entwickelt und evaluiert. Im folgenden Abschnitt soll kurz auf die Stärken und Limitationen der vorliegenden Arbeit eingegangen werden.

Studienqualität:

Zwar ist die vorliegende Studie keine randomisierte Studie, sondern eine Fall-Kontroll-Studie mit nicht-randomisierten Vergleichsgruppen mit Vor- und Nachtests. Cantillon et al (Cantillon and Jones 1999) schreiben, dass kontrollierte Studien von Ausbildungsaktivitäten besonders schwierig sind, da häufig Probleme bei der Identifizierung von geeigneten Kontrollgruppen auftreten. Außerdem könnten sie aufgrund der einzigartigen Natur jeder Lernumgebung nur in geringem Maße auf andere Gebiete übertragen werden. Die Schwäche von randomisierten Studien in diesem Themengebiet ist jedoch, dass die Ärzte wissen, dass ihr Verhalten evaluiert wird, und deshalb die Motivation zur Verhaltensänderung gesteigert ist. In der vorliegenden Arbeit wurde die Verhaltensänderung der Ärzte anhand der anonymisierten Daten der Kassenärztlichen Vereinigung Bayern (KVB) ohne Wissen der Ärzte erhoben. Dies erlaubt zwar nur einen Rückschluss auf die Teilnehmergruppe insgesamt und keine Auswertung einer individuellen Verhaltensänderung in Bezug auf weitere Eigenschaften eines Arztes, die sicher entscheidend zur Wirksamkeit einer Fortbildungsintervention beitragen, sehr wohl aber eine allgemeine Aussage über die Wirksamkeit der Fortbildungsmaßnahme auf das Verhalten eines Querschnitts der Teilnehmer.

Teilnehmer:

Ein wichtiger Grundsatz für eine effektive Fortbildung ist nach Collins (Collins 2004), dass die Lernenden als Individuen respektiert werden und die Teilnahme an der Fortbildung freiwillig erfolgt. Zu den Stärken dieser Untersuchung zählt deshalb die Freiwilligkeit der Teilnahme an der Fortbildungsveranstaltung und deren Evaluation. Das vorliegende Fortbildungskonzept wurde im Rahmen einer neuen Fortbildungsreihe am Klinikum der Universität München erstmals eingesetzt und evaluiert. Dazu wurden die Teilnehmer wie üblich schriftlich eingeladen. In der Einladung erfuhren die Ärzte, dass ein neues interaktives Konzept angewendet werden sollte. Zu Beginn der Veranstaltungen wurden die Teilnehmer dann darum gebeten, freiwillig Fragebögen auszufüllen, um eine Evaluation im Rahmen einer medizinischen Doktorarbeit zu ermöglichen. Zusätzlich wurde bei der Einführung auf diese Evaluation hingewiesen.

Die Teilnehmer erhielten außerdem nicht wie in anderen Studien (Fordis, King et al. 2005) finanzielle oder andere Anreize, um an der vorliegenden Studie teilzunehmen. Sie mussten Fortbildungsgebühren bezahlen. Daher kann davon ausgegangen werden, dass die Motivation der Ärzte zum Besuch von Fortbildungen nicht wesentlich beeinflusst und deshalb die Ergebnisse nicht verfälscht wurden. Allerdings ist eine Störung der internen und externen Validität nicht ausgeschlossen, da einerseits Messeffekte (das Wissen, dass das Wissen getestet wird) und andererseits eine Selektion das Verhalten und die Motivation der Ärzte verändern können. Die Selektion kann dabei auf der einen Seite darin bestehen, dass vorwiegend Ärzte an einer Fortbildung teilnehmen, die große Wissenslücken bei sich festgestellt haben, auf der anderen Seite aber auch Ärzte anzieht, die sich besonders für ein bestimmtes Thema interessieren und bereits ein gutes Vorwissen haben. Letzteres konnten Sibley et al (1982) zeigen.

In der Evaluation zeigte sich, dass die Mehrheit (zwei Drittel) der teilnehmenden Ärzte sehr häufig Fortbildungen besucht und dass die Themen der evaluierten Fortbildungen eine hohe Relevanz für sie hatten. Circa 20% der Ärzte gaben außerdem an, bereits Fortbildungen zu den Themen Endokrinologie und Diabetologie besucht zu haben. In der Eingangsevaluation gaben sie an, dass die Interaktivität und die Fallorientierung

wichtige Gründe für die Teilnahme waren. So kann einerseits darauf geschlossen werden, dass die Studienpopulation aus sehr engagierten und interessierten Ärzten bestand, die sich gezielt diese Fortbildungsform ausgesucht hatten. Im Vergleich mit der Kontrollgruppe zeigte sich so zum Beispiel, dass die Ärzte der Interventionsgruppe im Durchschnitt etwa 40% mehr Diabetiker behandelten. Auch beim Vergleich des durchschnittlichen relativen Anteils der Patienten mit Diabetes, die auf Mikroalbuminurie getestet wurden, fiel auf, dass in beiden untersuchten Zeitspannen die Ärzte der Diabetes-Fortbildung durchschnittlich 10% der Diabetiker auf Mikroalbuminurie testeten, doppelt so viele wie die Ärzte der Kontrollgruppe (5%). Andererseits kann nicht darauf geschlossen werden, dass die Effekte auf das Verhalten der Ärzte nur auf dieser Intervention beruhen. Dies ist ein Grund, weshalb eine Verallgemeinerung oder eine generalisierte Aussage der Ergebnisse der vorliegenden Studie nicht möglich ist. Außerdem war es nicht möglich, das Verhalten aller Teilnehmer anhand der KV-Daten zu analysieren, da nicht alle Ärzte eine eigene KV-Nummer hatten oder an einem Krankenhaus angestellt waren und so keine KV-Daten verfügbar waren.

Lernziele:

In dem vorliegenden Fortbildungskonzept wurden konkrete Lernziele formuliert und die Präsenzveranstaltung darauf abgestimmt. Dies fordern auch andere Experten für qualitätvolle Fortbildungsveranstaltungen (Soumerai and Avorn 1990; Collins 2004; BAEK 2007). Allerdings zeigte sich in der Auswertung des Diagnose- und Leistungsverhaltens der Ärzte, dass manche Lernziele – insbesondere die der Fortbildung zum Thema Sekundäre Hypertonie – anscheinend an der Praxis vorbeigehen und deshalb überdacht werden müssen. Außerdem könnte es sein, dass die Anzahl der definierten Lernziele zu hoch liegt und eingeschränkt werden sollte, um wirklich eine belegbare Verhaltensänderung der Ärzte bewirken zu können. Wahrscheinlich ist es sinnvoller, nur einige wenige relevante Lernziele zu definieren und diese zu bearbeiten, statt einen Überblick über ein großes Themengebiet zu geben. Dies wurde in dem vorliegenden Fortbildungsformat zwar bereits versucht, indem Kernfragen zu den einzelnen Themenkomplexen gestellt wurden. Jedoch fokussierten

sich die Experten und die Patientenfälle trotz dieses Konzepts vielleicht nicht ausreichend auf diese Kernfragen. Ob es sinnvoll ist, sich beispielsweise auf sehr wenige Kernfragen zu beschränken, und ob dies von den Fortbildungsteilnehmern angenommen wird, muss in weiteren Studien untersucht werden.

Fortbildungsformat:

Außerdem muss beachtet werden, dass sich die Lernstile Erwachsener voneinander unterscheiden. Als Lösung rät Collins (2004), verschiedene Methoden der Wissensvermittlung wie Diskussionen in Kleingruppen oder im Plenum, Rollenspiele, Vorträge, Fallstudien, Spiele, Wissenstests und Lerntechnologien einzusetzen und abzuwechseln. Collins schreibt: „Studien dazu haben gezeigt, dass über einen Zeitraum von drei Tagen nur 10% dessen, was wir lesen, 20% von Gehörtem, 30% von Gesehenem, 50% was gehört und gesehen wurde, 70 % von selbst Gesagtem und 90% von dem, was wir sagen und tun, behalten wird. Diese Ergebnisse zeigen, dass eine optimale Fortbildung demonstrative, praktische und lehrende Elemente vereint." Diesen Empfehlungen schließt sich auch die Bundesärztekammer (BAEK 2007) an und definiert entsprechende Kriterien für Fortbildungsveranstaltungen, die wirksam Wissen vermitteln. Dabei werden die Praxisrelevanz, die Interaktivität, die Vorerfahrungen und persönlichen Bedürfnisse der Lernenden und die Notwendigkeit von Feedback und weiteren aktivierenden Fortbildungsmethoden betont. Dafür bietet sich nach der Bundesärztekammer problemorientiertes, fallbezogenes Lernen, vorzugsweise in Kleingruppen, mit verfügbaren Experten zur individuellen Beratung an, um gleichzeitig die persönlichen Lernbedürfnisse berücksichtigen zu können, eine kritische Anwendung von Expertenwissen auf eigene Probleme zu aktivieren und das Problembewusstsein und den Umgang mit Informationsquellen zu stärken. Die Bundesärztekammer geht davon aus, dass diesen Ansprüchen eher Veranstaltungen in kleinen Gruppen als solche vor großem Auditorium gerecht werden. Im Gegensatz dazu geht Michaelsen (2002) davon aus, dass Lernen in Gruppen (Team-based Learning) auch in großen Gruppen funktioniert (vgl. Abschnitt I.1.3.1. Team-based Learning).

Die oben genannten Grundsätze für effektive Fortbildungen wurden im vorliegenden Fortbildungskonzept berücksichtigt und integriert. Dabei wurde neben dem Konzept des Problem-based Learning insbesondere die Theorie des Team-based Learning verwendet und angepasst. Die Problemorientiertheit der Fortbildung und die Gruppenarbeit an realistischen Patientenfällen wurden von den Teilnehmern als sehr anregend und lehrreich bewertet. Die Relevanz und Praxisnähe der Themen der Fortbildungen und die Interaktivität wurden von den Teilnehmern ebenfalls sehr hoch bewertet. In der vorliegenden Arbeit konnte gezeigt werden, dass ein problembasiertes Fortbildungskonzept auf eine hohe Akzeptanz der teilnehmenden Ärzte stieß, die Teilnehmer zu interaktiven Diskussionen in Kleingruppen und im Plenum aktivierte und die Fälle die Teilnehmer zum Nachdenken brachten. Dabei gab es jedoch keinen Tutor, sondern nur den Dozenten als Berater für alle Gruppen. Allerdings wird in der Literatur die Notwendigkeit eines Tutors bei PBL in Frage gestellt (Norman and Schmidt 1992).

In der Literatur wird außerdem empfohlen, auf das selbstbestimmte Lernverhalten Erwachsener Rücksicht zu nehmen. Als Unterstützung eignen sich beispielsweise Handouts und Literaturempfehlungen (Collins 2004). Dies erfolgte in der vorliegenden Studie mittels des zu Beginn der Fortbildungsveranstaltungen ausgeteilten Syllabus mit allen Vortragsfolien der Experten und den Fällen, die den Teilnehmern ein umfassendes Selbststudium und eine Wiederholung oder Klärung der behandelten Inhalte ermöglichten. Bisher gibt es jedoch kaum wissenschaftliche Erkenntnisse dazu, inwieweit gedruckte Fortbildungsmaterialen die klinische Tätigkeit beeinflussen können (Farmer 2003). Wie diese Möglichkeit zum Selbststudium von den Teilnehmern genutzt wurde, wurde in der vorliegenden Arbeit nicht evaluiert.

Fortbildungsstruktur:

Die Struktur der Präsenzveranstaltung war genau vorgegeben und verlief immer nach demselben Muster. Dieses wurden den Teilnehmern der Fortbildung bei der Begrüßung und Einführung kurz erklärt. Diese Voraussetzung nennt auch Collins (2004) in ihren zehn Grundlagen für effektive Fortbildungsveranstaltungen. Sie schreibt, dass Leiter von Fortbildungsveranstaltungen zu Beginn die Inhalte und

Lernziele klar definieren und die Veranstaltung gut strukturieren sollten, da Erwachsene sehr zielorientiert lernen. Auch die von den Teilnehmern aufgebrachte Zeit sollte wertgeschätzt werden, indem die Fortbildungsveranstaltungen zeitlich effektiv und strukturiert aufgebaut werden. Diese Forderung wurde auch aus den Freitextkommentaren deutlich, die die Teilnehmer der beschriebenen Fortbildungsveranstaltungen formulierten.

Nach Ansicht von Collins sind Erwachsene außerdem eher problem- als subjektorientierte Lerner und lernen am besten anhand praktischer Anwendungsmöglichkeiten des neuen Wissens. Der Fortbildungsinhalt sollte deshalb eine hohe Relevanz und Praxisnähe aufweisen. Zusätzlich sollten Fortbildungen lernerzentriert aufgebaut sein, das bedeutet, den Lerner aktiv einzubeziehen und dadurch zu aktivieren. Das gelingt beispielsweise, wenn die Teilnehmer dazu stimuliert werden, Erfahrungen auszutauschen, in Frage zu stellen und zu diskutieren. Die Diskussionsteile sollten sich mit praktischen Übungseinheiten abwechseln, um die Teilnehmer zu aktivieren. Als Strategien, um dieses Ziel zu erreichen, nennt Collins gemeinschaftliches Lösen authentischer Fälle, realer Bezug der neu erlernten Ideen auf bekannte praktische Probleme und das Anbieten von Lösungen.

Rolle des Dozenten:

Im vorliegenden Fortbildungskonzept wandelte sich die Rolle des Experten von einer rein vortragenden Tätigkeit zu einer eher beratenden und steuernden Funktion. Nach der kurzen Einführung des Themas standen die Dozenten den Kleingruppen beratend zur Verfügung und bei der anschließenden interaktiven Gruppendiskussion im Plenum als Diskussionsleiter und Experten. Diese Empfehlung spricht auch Collins (2004) aus. Sie ist der Ansicht, dass in einer optimalen Lernsituation alle Teilnehmer, auch die Dozenten, voneinander lernen sollten und der Dozent eher als Führer statt als der einzige Wissende gesehen werden sollte. Premi et al (1994) schreiben, dass eine praxisnahe, fallbasierte Fortbildung mit Teamarbeit die traditionelle Beziehung des Vortragenden zu den Teilnehmern verändert und die Vortragenden und Leiter eine mehr beratende Funktion bekommen. Nach seiner Ansicht entsteht durch die eher beratende Funktion der Experten eine konstruktives, verständnisvolles und

gemeinschaftliches Klima, das es den teilnehmenden Ärzten ermöglicht, die Schwierigkeiten zu diskutieren, die bei der Integration von neuen wissenschaftlichen Erkenntnissen in die spätere praktische Arbeit auftreten.

Der Doppelrolle des Dozenten als Moderator der Gruppendiskussion im Plenum sowie als Experte wurde in der Hauptstudie durch eine Doppelmoderation Rechnung getragen, indem dem Dozenten ein hausärztlich tätiger Kollege zur Seite gestellt wurde, der die Diskussion effektiver und aus hausärztlicher Sicht gestalten sollte. Allerdings zeigte sich, dass eine stringente Moderation nur bei bereits erfahrenen Moderatoren wirklich funktionierte, trotz intensiver mündlicher Erklärung der Rolle. Deshalb stellt sich die Frage, ob diese Rolle nicht von einem ausgebildeten Moderator, der nicht unbedingt hausärztlich tätig sein muss, besser ausgefüllt werden kann. Eine stringente Moderation der Gruppendiskussion ist unbedingt notwendig, um die Struktur und den Zeitplan der Veranstaltung einzuhalten und die Diskussion effektiv zu gestalten. Eine wenig strukturierte Diskussion und mangelnde Moderation führte zu einer hohen Unzufriedenheit der Teilnehmer. Eine effektive Lernumgebung empfehlen auch andere Experten (Donner-Banzhoff 2001; Collins 2004; BAEK 2007).

Testinstrumente:

Als Stärke der Studie kann die hohe Rücklaufquote der Evaluationsfragebögen genannt werden. Der Eingangsfragebogen (FB1) wurde von 94,1% der teilnehmenden Ärzte ausgefüllt. Die Gründe hierfür können wiederum in der Freiwilligkeit und Anonymität sowie den zeitlichen Rahmenbedingungen liegen. Die Teilnehmer erhielten den Fragebogen zusammen mit den Fortbildungsunterlagen bei der Anmeldung unmittelbar vor der Veranstaltung. Wahrscheinlich nutzten viele die Zeit bis zu Beginn der Fortbildung zum Ausfüllen des Fragebogens. Dies scheint also ein guter Zeitpunkt für die Evaluation zu sein.

Kritisch diskutiert werden kann die Verwendung derselben Fragen für die Tests im Vor- und Nachwissenstest. Zur Verteidigung des Testformates kann angeführt werden, dass die Lösungen der Fragen im Vorwissenstest nicht dargestellt wurden und so wirklich nur als formative Evaluation des Vorwissens der Teilnehmer verwendet wurden. Durch die Auflösung und Diskussion der richtigen Antworten direkt nach

dem Nachwissenstest bekamen die Teilnehmer ein zeitnahes Feedback, und offene Fragen konnten geklärt werden. Außerdem ermöglichte diese Evaluation dem Dozenten und den Leitern der Fortbildung, zu überprüfen, ob die Lernziele ausreichend behandelt wurden und erreicht worden sind. Zudem sind die Wissenstests auf diese Weise gut vergleichbar, da sie die gleiche Schwierigkeit, Validität und Reliabilität haben. Denselben Wissenstest vor und nach einer Fortbildungsintervention führten auch Patwardhan et al (2006) durch und konnten dabei einen signifikanten Wissenserwerb in einem Großteil der Fragen feststellen. Fordis et al (2005) benützten ebenfalls dieselben Fragen, um das Vor- und Nachwissen zu testen, stellten jedoch die Reihenfolge der Fragen um.

Durch die Verwendung von Multiple-Choice Fragen kann jedoch nur eine bestimmte Art von Wissen, nämlich deskriptives Wissen, geprüft werden (Miller 1990; Schmidt 2000).

Eine verbesserte klinische Entscheidungsfähigkeit der Ärzte konnte jedoch nicht nachgewiesen werden. Eine Aussage über den Wissenserwerb durch das beschriebene Fortbildungskonzept beschränkt sich deshalb auf das Faktenwissen. Die Qualität der summativen Evaluation des Wissens könnte durch die Verwendung eines anderen Fragenformates, beispielsweise eines Key-Feature-Formats (siehe Abschnitt IV.1.; (Bordage 1987; Bordage 1995; Kopp 2006), verbessert werden. Mit diesem Testformat kann prozedurales Wissen (klinische Entscheidungsfindung) geprüft werden.

Wissensevaluation:

Collins (2004) betont in ihren zehn Grundsätzen des Lernverhaltens Erwachsener (siehe Abbildung 1: Principles of Adult Learning), dass es wichtig sei, dass Fortbildungsleiter erwachsenen Lernenden dabei helfen, die Verbindungen zwischen früheren Erfahrungen und neuen Informationen zu erkennen. Deshalb sollte bei jeder Fortbildungsveranstaltung zu Beginn festgestellt werden, welches Wissen bereits vorhanden ist. Eine Aktivierung der Teilnehmer durch die Erhebung des Vorwissens betonen auch Soumerai et al (1990). Nach Bransford et al (1972) erleichtert eine Aktivierung des Vorwissens den darauf folgenden Lernprozess und den Erwerb neuen Wissens. Diese Maxime der kognitiven Psychologie wird durch mehrere Studien in

verschiedenen Fachgebieten (De Groot 1946; Spilich 1972) gestützt; andere haben allerdings gezeigt, dass dieser Lernprozess nicht stattfindet, wenn kein relevantes Vorwissen aktiviert wird (Franks 1982; Schmidt, Norman et al. 1990). Norman und Schmidt (1992) ziehen deshalb den Schluss, dass der Lernkontext eine solche Aktivierung ermöglichen muss. Die Diskussion eines Problems in Kleingruppen ist nach Ansicht von Norman und Schmidt eine der Methoden, die relevantes Vorwissen zu Tage fördern kann. Die Evaluation des Lernerfolges wird in zahlreichen Studien und auch von der Bundesärztekammer als wichtig eingeschätzt. Konstruktives Feedback ist nach Ansicht von Collins (2004) einer der wichtigsten Grundsätze in der Erwachsenenbildung, um erfolgreiches Lernen und die Meisterung neuen Wissens zu erreichen. Die Bundesärztekammer regt außerdem an, den Lernenden die Möglichkeit zu geben, den Fortbildungserfolg, das bedeutet den Zuwachs an Wissen und Fähigkeiten, in Form einer Selbstkontrolle überprüfen und dokumentieren zu können (BAEK 2007). Um die Evaluation und Qualitätskontrolle von Fortbildungsmaßnahmen mit der Zeit selbstverständlich werden zu lassen, vergibt die Bundesärztekammer deshalb für Veranstaltungen, die Lernkontrollen beinhalten, zusätzliche Fortbildungspunkte.

Als sehr effektive Vorwissenstestmöglichkeit erwähnt Collins (2004) elektronische Abstimmsysteme, da mit diesen sowohl der Lernende als auch der Lehrer herausfinden kann, welcher Wissenstand vorhanden ist. Das in der vorliegenden Studie verwendete elektronische Abstimmungssystem wurde von den Teilnehmern sehr gut angenommen. Die formative und summative Wissensevaluation mit einem solchen System hat außerdem den Vorteil, dass die Teilnehmer sich selbst mit den anderen Teilnehmern vergleichen können, ohne das Gefühl zu haben, geprüft zu werden. Diese formative Evaluation bringt Teilnehmer dazu, ihr Vorwissen zu aktivieren, ohne eine individuelle Bewertung und daraus folgende Konsequenzen fürchten zu müssen (Donner-Banzhoff 2001). Teilweise wünschten sich die Ärzte in den Freitextkommentaren der Fragebögen auch eine intensivere Nutzung der Abstimmungstechnologie mit mobiTED. Allerdings könnte es sein, dass vorwiegend Teilnehmer diese Abstimmungsmöglichkeit positiv bewerten, die sich bezüglich ihres Vorwissens sicher sind oder sehr motiviert sind, dazuzulernen.

Zusammenfassung

Insgesamt zeigte sich die hohe Relevanz der Themen Diabetes mellitus und Schilddrüsenerkrankungen, da die Teilnehmer der Fortbildungsveranstaltungen im Durchschnitt sehr viele Patienten mit diesen Diagnosen behandelten. Allerdings stellten sich hier sehr große Unterschiede heraus sowohl in der Anzahl der behandelten Patienten, der erbrachten Leistungen und der Überweisungsfrequenz der Patienten an Fachärzte. Damit kann auf ein sehr inhomogenes Vorwissen und Verhalten in der Praxis der Ärzte, die an den Fortbildungen teilnahmen, geschlossen werden. Beim Thema Diabetes mellitus zeigte sich auch im Vergleich zur Kontrollgruppe, dass die Teilnehmer der Diabetes-Fortbildung im Durchschnitt eine deutlich höhere Patientenanzahl mit Diabetes mellitus behandelten.

Die vorliegende Arbeit erbrachte in Bezug auf eine objektivierbare Verhaltensänderung der Ärzte gemischte Ergebnisse. So konnte gezeigt werden, dass eine interaktive Fortbildungsmaßnahme in gewissen Bereichen zu einer Verhaltensveränderung der Ärzte führen kann. Interessanterweise war dies vor allem beim Thema Diabetes mellitus zu beobachten, wo die Teilnehmer im Wissenstest durchschnittlich ein geringeres Vorwissen aufwiesen als bei den anderen beiden Themen Schilddrüsenerkrankungen und sekundäre Hypertonie. Die Teilnehmer beim Thema Diabetes erreichten zudem den höchsten durchschnittlichen Wissenszuwachs im Nachwissenstest. Dies zeigt, dass die Teilnehmer der Diabetes-Fortbildung die Lernziele der Fortbildung erreicht haben und sich diese in den Daten der Kassenärztlichen Vereinigung auch gut widerspiegeln. Allerdings muss einschränkend gesagt werden, dass die Veränderungen nur bei der Überweisungsfrequenz zum Diabetologen statistisch signifikant waren. In anderen Lernzielparametern zeigte sich zwar ebenfalls eine deutliche Verbesserung des Verhaltens (Überweisung zur Funduskopie, Durchführung von Mikroalbuminurietests), die jedoch statistisch nicht signifikant war und auch im Vergleich mit der Kontrollgruppe nicht eindeutig war. Allerdings können auch statistisch nicht signifikante Verhaltensänderungen eine sehr große Wirkung auf die Patientenversorgung haben.

Gerade beim Thema Schilddrüsenerkrankungen, wo es zum zweithöchsten Wissenszuwachs im Wissenstest bei den Teilnehmer kam, zeigte sich jedoch keine objektivierbare Verhaltensänderung der Teilnehmer, in manchen Untergruppen sogar eine deutliche Verhaltensänderung entgegen der ursprünglichen Erwartungen. Dies könnte einerseits vielleicht an einer besseren Einschätzung der eigenen Kompetenz bezüglich der Untersuchungsmethode (Schilddrüsensonographie) liegen, was aber aufgrund der ebenfalls nicht veränderten Überweisungsrate zu Fachärzten zur Schilddrüsensonographie eher ausgeschlossen werden kann. Andererseits könnten abrechnungstechnische Gründe vorliegen, zum Beispiel dass diese Untersuchung in einer Patientenpauschale abgerechnet wird oder insgesamt seltener durchgeführt wird, da sie nicht oder schlecht bezahlt wird. Die Tatsache, dass die Ärzte, die viele Schilddrüsenpatienten behandeln, diese nach der Fortbildung seltener zur Schilddrüsenszintigraphie überwiesen, könnte allerdings auch darauf hindeuten, dass diese die Indikation zur Schilddrüsenszintigraphie nach den Kriterien, die in der Fortbildung erklärt wurden, enger als vorher stellten und deshalb weniger Patienten überwiesen. Eine geringere Anzahl an Patienten mit der Diagnose einer Schilddrüsenerkrankung kann jedenfalls nicht die Ursache sein, da die Anzahl der behandelten Patienten mit Schilddrüsenerkrankungen in den untersuchten Quartalen weitgehend konstant blieb.

3. Ausblick

Die Kombination von Kurzvorträgen mit fallbasierter Gruppenarbeit und interaktiver Diskussion im Plenum stieß auf eine hohe Akzeptanz der teilnehmenden Ärzte und führte zu einem signifikanten kurzfristigen Wissenzuwachs und einer Verhaltensänderung in manchen Bereichen.

Um herauszufinden, ob das interaktive und fallorientierte Fortbildungskonzept zu einem guten Langzeitbehalten und zu einer Verbesserung der strukturierten Problemanalyse beim Selbstlernen führt, sollen die Ergebnisse der Nachbearbeitung von Online-Lernfällen zu den Fortbildungsthemen in einer weiteren Studie evaluiert werden. Zu diesem Zweck wurden insgesamt 11 CME-Module zu Themen der Endokrinologie (z.B. Schilddrüsenknoten, Osteoporose) und Diabetologie (z.B.: Diagnose und Einstellung des Diabetes mellitus) erstellt, die jeweils zwei bis sechs Kurzfälle enthalten. Der Zeitaufwand zur Erstellung der Online-Lernfälle betrug pro CME-Modul etwa 10 bis 15 Stunden. Die Module wurden von Inhaltsexperten, einer Medizinpädagogin und einer Sachverständigen für Prüfungsfragen hinsichtlich inhaltlicher, formaler und didaktischer Gesichtspunkte geprüft (siehe Anhang 6 - Erstellung der Online-Lernfälle). Das verwendete Online-Lernprogramm CASUS ist sehr benutzerfreundlich, einfach anzuwenden, hat sich bereits in der Studentenausbildung bewährt und kann von jedem Computer mit Internetzugang aus genutzt werden (Fischer, Schauer et al. 1996; Simonsohn and Fischer 2004).

Die Kerninhalte der Online-Lernfälle beruhen auf den bereits in den Präsenzveranstaltungen bearbeiteten Fällen und wurden durch Bild- und Informationsmaterial ergänzt. Jedes Modul besteht aus einer Lernphase mit einzelnen Kurzfällen. Zu jedem Fall müssen die Teilnehmer mehrere Fragen (Multiple-Choice-Fragen, Freitext-Fragen, Long menue-Fragen) beantworten. Diese werden sofort bewertet, die richtigen und falschen Antwortmöglichkeiten intensiv kommentiert und zusätzlich mit Expertenkommentaren angereichert. Nach dieser Lernphase folgt eine kurze Zusammenfassung der Inhalte und Lernziele. Um CME-Punkte zu erwerben, können die Teilnehmer weitere zehn MC-Fragen im Single-Best-Answer-Format als Erfolgskontrolle zum Thema des gesamten CME-Moduls beantworten. Dieses

Fragenformat fordert die Bayerische Landesärztekammer bislang als Erfolgskontrolle bei ärztlichen Fortbildungen der Kategorie D (Strukturierte interaktive Fortbildung über Printmedien, Online-Medien und audiovisuelle Medien). Wenn sich die Teilnehmer mit ihren persönlichen Daten einloggen, können sie von der Bayerischen Landesärztekammer zertifizierte Fortbildungspunkte erhalten. Die Teilnehmer können auf diese Weise ihr Wissen vertiefen und zusätzliche Fortbildungspunkte sammeln. Die Motivation und Akzeptanz der Online-Fortbildung allgemein und als Ergänzung und im Vergleich zur Präsenzveranstaltung soll mittels eines Fragebogens evaluiert werden.

Die Erwartungen an und Erfahrungen von Ärzten mit interaktiven Online-CME-Aktivitäten und die Faktoren, die diese beeinflussen, analysierten Sargeant et al (2004) in einer Studie. Sie fanden heraus, dass die Ärzte ihre Erwartungen und Haltungen gegenüber Online-Fortbildungen aus einem Vergleich mit dem Format, mit dem sie die meisten Erfahrungen hatten, den Präsenzveranstaltungen, ableiteten. Ihre Ansichten wurden einerseits von der Qualität des Programms, andererseits von der Fähigkeit des Formats beeinflusst, individuelle Vorlieben zu befriedigen sei es in Bezug auf die Selbstbestimmung, die Dauer und Fortschritt in der Bearbeitung, der Lernmethode und der Möglichkeit zur Reflexion. Außerdem richteten sich die Ansichten und Erwartungen der Ärzte nach der Qualität und Quantität der interpersonellen Interaktion. Die wichtigsten Faktoren waren dabei die Atmosphäre, der Lerneffekt der Interaktionen und die Rolle des Tutors oder Leiters der Online-Fortbildung. Vorherige Erfahrungen mit Online-CME-Aktivitäten beeinflussten die Haltung der Ärzte ebenfalls. Die Autoren der Studie folgerten daraus, dass Online-Fortbildungen auf der Basis der bisherigen wissenschaftlichen Evidenz für effektive CME-Aktivitäten entwickelt und eingesetzt werden sollten. Dazu sollten Angebote zur Verfügung stehen, die unterschiedlichen Lernvorlieben Rechnung tragen. Die Anbieter sollten außerdem geeignete Tutoren für die Onlineformate ausbilden und zur Verfügung stellen. Die Entwicklung und der Einsatz von Online-Fortbildungsangeboten sollten auf Prinzipien der Lerntheorie aufbauen.

Frühere Studien hatten bereits gezeigt, dass es in Bezug auf Lernqualität und –erfolge keine Unterschiede zwischen Online- und Präsenzfortbildungen gibt. Die bevorzugten

Lernstile beeinflussten die Erfolgsrate in beiden Fortbildungsformaten nicht. Johnson (2000) zeigte jedoch, dass die Teilnehmer etwas zufriedener mit dem Präsenzformat waren als mit den Online-Angeboten. In einer Metaanalyse zur Wirksamkeit von webbasierten CME-Aktivitäten fanden Curran et al (2005) aber heraus, dass die teilnehmenden Ärzte generell zufrieden mit webbasierten Fortbildungen waren. Das Internet wurde als ideales Medium für CME angesehen, da Informationen leicht verfügbar, auf dem neuesten Stand und schnell erhältlich sind. Der Hauptnutzen scheint neben dem leichten Zugang, geringem Aufwand und dem Multimedia-Format die Möglichkeit zu sein, interaktive klinische Fälle anzubieten. Außerdem ermöglichen webbasierte Fortbildungsangebote den Ärzten, ihre Lernziele eigenständig und unabhängig von Zeit und Ort zu verfolgen. Diese Tatsache könnte nach Peterson et al (1999) auch eine Ursache dafür sein, dass traditionelle CME-Angebote nicht effektiv zu Wissenstransfer und Verhaltensänderungen führen können, da sie aufgrund ihrer Struktur sehr unflexibel sind. In traditionellen CME-Aktivitäten wählt der Teilnehmer weder die Themen noch das Tempo oder den Ort der Fortbildung selbst aus. Der Wissenserwerb kann auf diese Weise nicht in der Situation oder in dem Maße stattfinden, in der er für eine konkrete Problemstellung im Arbeitsalltag des Arztes benötigt wird.

Allerdings existieren immer noch Barrieren, die Online-CME-Aktivitäten zu nutzen: Zehn dieser Barrieren definierte Aragon et al (2002) in einer großen Studie. Zu den wichtigsten zählen Zugangs- und Anmeldevorgänge, technische Anforderungen und fehlende soziale Interaktion. Curran et al (2005) nennen außerdem mangelnde Computerkenntnisse und das Gefühl der Informationsüberflutung. Deshalb sollten die Online-CME-Angebote sehr benutzerfreundlich aufgebaut sein, sich gut in den Arbeitsalltag der Ärzte integrieren lassen und sich mit konkreten, abgegrenzten Themen beschäftigen. Zusätzlich könnte ein Benutzertraining angeboten werden. Die Aufgabe von Entwicklern von Online-Angeboten ist es also, diese Erfordernisse im Auge zu behalten und Pädagogik, Technik, Organisation und Strategien mit geeigneten Lernprinzipien für Online-Fortbildungen zu kombinieren.

Curran et al beklagen den Mangel an klarer Evidenz in der bisherigen Literatur, welche Methoden und Formate webbasierter Fortbildungsmaßnahmen effektiv zu

einem veränderten Verhalten und einer verbesserten Patientenversorgung führen können. Sie kommen jedoch zu dem Schluss, dass interaktive CME-Maßnahmen, die die Teilnehmer aktivieren und die Möglichkeit zur Erlangung von Fertigkeiten bieten, eine Verhaltensänderung auslösen können und in manchen Fällen auch einen Effekt auf die Gesundheitsversorgung haben könnten. Nach Curran et al stehen die Entwickler und Anbieter von Online-Fortbildungen vor der Herausforderung, interaktive Lernumgebungen zu gestalten, die die Möglichkeiten des Internets für eine erleichterte Kommunikation zwischen Lehrenden und Lernenden ausnützen und Informationen in interessanten Formaten mit Multimedia-Unterstützung anbieten. Weitere Studien seien nötig, die verschiedene Formate und Methoden miteinander vergleichen, um herauszufinden, welche Lehrstrategien am effektivsten sind. Curran et al stellen die Frage: „Wie können wir webbasierte CME-Fortbildung auf eine Weise entwickeln und fördern, die Kernprinzipien der Erwachsenenbildung berücksichtigt und zu Wissenszuwachs und –transfer im klinischen Alltag von Ärzten führt?"

Wutoh et al (2004) schlagen zur Untersuchung von internetbasierten CME-Aktivitäten Folgendes vor:

1. Zu untersuchen, welche Strategien und Methoden in traditionellen Formaten erfolgreich sind, und diese auf internetbasierte Fortbildungsformate zu übertragen.
2. Mehr Studien durchzuführen, die wie randomisierte, kontrollierte Studien auf soliden wissenschaftlichen Grundlagen beruhen, um anhand von objektiven Bewertungsmaßstäben herauszufinden, ob Onlinefortbildungen zu einer Verhaltensänderung führen.
3. Erstellung von zusätzlichen Online-Lernprogrammen, die individuellen Ansprüchen und Vorlieben genügen (basierend auf Marktanalysen).

Außerdem fordern sie aggressive Marketingstrategien, um Ärzte dazu anzuregen und dabei zu unterstützen, die Hindernisse im Bereich der Technik zu überwinden.

Nach Ronald Hardens (2005) Ansicht können die Kriterien für eine effektive Fortbildung von E-Learning-Angeboten erfüllt werden, da sie sowohl Möglichkeiten zum selbstbestimmten Lernen als auch zum zeitnahen Lernen bieten. Zu den Kernelementen bei der Einwicklung eines E-Learning-Programmes zählen eine Datenbank mit wiederverwertbaren Lerninhalten, eine virtuelle Praxis und eine Vielfalt an Lernzielen und Methoden zur Selbsteinschätzung und –testung. Auch

internetbasierte Fortbildungsformate sollten dabei im Sinne eines Curriculums strukturiert sein. Ronald Harden schreibt in seinem Artikel „A new vision for distance learning and Continuing Medical Education“ (Harden 2005), dass internetbasierte CME-Angebote eine zunehmende Rolle in der ärztlichen Fortbildung spielen werden. Allerdings geht er davon aus, dass Onlineformate die bisherigen Fortbildungsangebote nicht ersetzen, sondern Teil eines gemischten Ansatzes („Blended-learning“) in der Aus-, Weiter- und Fortbildung von Ärzten sein werden.

Die oben genannten Kriterien wurden in dem oben beschriebenen Onlineangebot umgesetzt. So wurden ansprechende Fälle mit Zusatzmaterial in Form von Sonographiebildern, Laborergebnissen und anderem Bildmaterial sowie Internetlinks erstellt und angeboten, deren Bearbeitung jederzeit unterbrochen und wieder aufgenommen werden konnte. Die Fälle beziehen sich auf die Themen der Präsenzveranstaltungen und beschäftigen sich mit konkreten, klar umschriebenen klinischen Kernfragen und -problemen. Denkbare Strategien, um Ärzte zur Teilnahme zu motivieren, sind unter anderem eine ausführliche Darstellung in den Präsenzveranstaltungen mit der Möglichkeit, die Programme unter Anleitung zu testen, sowie ein gezieltes Benutzertraining. Sinnvoll wäre auch eine Befragung der Teilnehmer der Präsenzveranstaltungen zu ihren konkreten Vorerfahrungen mit medizinischen Lernprogrammen sowie ihren Erwartungen und Wünschen. So könnte das Onlineangebot gezielt auf die Bedürfnisse der potentiellen Nutzer zugeschnitten werden. Außerdem sollte die richtige Zeitspanne bei einem solchen Zusatzangebot, die zwischen der Präsenzfortbildung und den Online-Lernfällen liegen sollte, herausgefunden werden. Die Online-Fortbildungen sollen nicht nur als Zusatz zur Präsenzveranstaltung sondern auch als Alternative angeboten werden. Gerade für Ärzte in ländlichen Regionen könnte dieses Angebot attraktiv sein. Deshalb soll die Werbestrategie umfassend ausgebaut werden. Zusätzlich ist geplant, die Online-Lernfälle auch im Zusammenhang mit Podcasts[3] von Fortbildungen und mit interaktiven Diskussionsforen mit den Experten und anderen Teilnehmern einzusetzen und die Nutzung dieser Fortbildungsangebote wissenschaftlich zu erforschen.

[3] Podcasts= Mediendateien, die über das Internet produziert und angeboten werden

4. Zusammenfassung:

In der vorliegenden Studie wurde ein innovatives, interaktives Fortbildungskonzept für Allgemeinmediziner und Internisten, basierend auf dem theoretisch fundierten Konzept des Team-based Learning, entwickelt und bezüglich Akzeptanz, Wissenserwerb und objektivierbarer Verhaltensänderung der teilnehmenden Ärzte evaluiert. Das Fortbildungskonzept kombiniert Expertenvorträge zu relevanten Kernthemen mit fallbasierter Kleingruppenarbeit und moderierter Diskussion. Es zeigte sich eine hohe Akzeptanz der Teilnehmer. Besonders die Interaktivität und die Fallorientierung wurden in der Evaluation als ausschlaggebend für die Teilnahme an der Fortbildungsveranstaltung angegeben. Auch die Erwartung an die Fortbildung, sowohl konkrete Entscheidungshilfen für die Praxis als auch neueste Erkenntnisse aus der Wissenschaft zu erfahren, scheint sich in diesem Fortbildungskonzept aus Expertenvorträgen zu Kernthemen kombiniert mit fallbasierter Kleingruppenarbeit zu erfüllen. Dies zeigte sich besonders in der Abschlussevaluation, bei der die Teilnehmer angaben, dass sie die Fortbildung sehr abwechslungsreich und lehrreich fanden. Sie äußerten, dass die Fälle sie zum Mitdenken aktiviert hätten. Die in der Eingangsevaluation angegebenen hohen Erwartungen an die Fortbildung wurden bei der Mehrheit der Befragten erfüllt. Zudem nahmen viele Ärzte an mehreren Veranstaltungen der Fortbildungsreihe teil.

Desweiteren führte das interaktive, fallorientierte Fortbildungskonzept zu einem signifikanten Wissenszuwachs bei Teilnehmern mit einem bereits gut ausgeprägten Vorwissen. Dabei lagen die durchschnittlichen Werte im Vorwissenstest sehr hoch. Zum höchsten relativen Wissenszuwachs kam es beim Thema Diabetes mellitus (62,6% richtige Fragen im Nachwissenstest, 42,9% Steigerung) und Schilddrüsenknoten (79,5% richtige Fragen im Nachwissenstest, 42,1% Steigerung). Am niedrigsten lag er beim Thema sekundäre Hypertonie (76,5% richtige Fragen im Nachwissenstest, 40,7% Steigerung). Die Ärzte gaben selbst an, viel gelernt zu haben und ihr Verhalten in der Praxis aufgrund des neu erlangten Wissens ändern zu wollen.

Bei der Analyse der Daten zum Diagnose-, Leistungs- und Überweisungsverhalten der Kassenärztlichen Vereinigung Bayern zeigte sich, dass die teilnehmenden Ärzte der

Fortbildung zum Thema Diabetes ihr Verhalten in der Versorgung von Diabetikern (Mikroalbuminurietests, Überweisung zur Funduskopie und zum Diabetologen) bis auf die Wundversorgung im Sinne der Lernziele der Fortbildung tatsächlich in der Praxis veränderten. Eine statistisch signifikante Verhaltensänderung der Fortbildungsteilnehmer zeigte sich dabei in Bezug auf die Überweisungsfrequenz von Patienten mit Diabetes mellitus zum Diabetologen. Die Teilnehmer überwiesen nach der Fortbildung 30,8% mehr Diabetes-Patienten zum Diabetologen. Eine deutliche Steigerung der Überweisung zum Facharzt für Augenheilkunde zur Funduskopie von 8,5% konnte ebenfalls gezeigt werden. Diese Zunahme erreichte jedoch keine statistische Signifikanz. Im Vergleich dazu nahm in diesen beiden untersuchten Bereichen die Überweisungsfrequenz der Ärzte der Kontrollgruppe ab (3%, n.s.). Bei der Analyse des Leistungsverhaltens der Ärzte der Fortbildung Diabetes zeigte sich eine Zunahme der durchgeführten Mikroalbuminurietests pro Diabetes-Patienten um 7,1%. Allerdings konnte eine ähnliche Steigerung auch bei der Kontrollgruppe nachgewiesen werden.

Beim Fortbildungsthema Schilddrüsenknoten zeigten sich bei der Auswertung der Daten keine wesentlichen Verhaltensänderungen bzgl. der eigenen Durchführung der oder der Überweisung zur weiteren Diagnostik (Schilddrüsensonographie, -farbduplexsonographie, -szintigraphie) der Teilnehmer der Fortbildung. Beim Thema sekundäre Hypertonie zeigte sich, dass die Teilnehmer insgesamt sehr wenige Patienten mit dieser Erkrankung behandeln bzw. die Diagnose außerordentlich selten stellen und auch die entsprechenden Untersuchungen (Bestimmung des Aldosteron-Renin-Quotienten, der Plasma-Metanephrine oder der Test auf Katecholamine im Urin) fast nie durchführen. Deshalb konnte keine statistische Auswertung erfolgen.

Bei hoher Akzeptanz der Teilnehmer stellt das vorliegende Fortbildungskonzept eine praktikable Möglichkeit dar, einen signifikanten kurzfristigen Wissenszuwachs und eine objektivierbare Verhaltensänderung bei Allgemeinmedizinern und Internisten zu bewirken. Ob das Fortbildungskonzept zu einem guten Langzeitbehalten und zu einer Verbesserung der strukturierten Problemanalyse führt und in anderen Bereichen der ärztlichen Fort- und Weiterbildung (z.B. Online) eingesetzt werden kann, soll in weiteren Studien evaluiert werden.

V. Literatur:

(2003). GKV-Modernisierungsgesetz. B. f. Gesundheit.
(2005). Großer Ansturm auf zertifizierte Fortbildungsveranstaltungen. Deutsche Ärztezeitung.
ACCME (2006). Annual report. 2008.
Allen, M., S. Ferrier, et al. (2007). "Family physicians' perceptions of academic detailing: a quantitative and qualitative study." BMC Med Educ **7**: 36.
Allen, S. W., Norman, G.R. and Brooks, L.R. (1992). "Experimental studies of learning dermatologic diagnosis: The impact of examples." Teaching and Learning in Medicine **4**: 35-44.
Anderson, J. G., L. L. Casebeer, et al. (1999). "Medcast: evaluation of an intelligent pull technology to support the information needs of physicians." Proc AMIA Symp: 466-70.
Angunawela, II, V. K. Diwan, et al. (1991). "Experimental evaluation of the effects of drug information on antibiotic prescribing: a study in outpatient care in an area of Sri lanka." Int J Epidemiol **20**(2): 558-64.
Aragon, S. R., Johnson, S.D. (2002). "Emerging Roles and Competencies for Training in E-Learning Environments." Advances in Developing Human Resources **4**(4): 424-439.
Armson, H., S. Kinzie, et al. (2007). "Translating learning into practice: lessons from the practice-based small group learning program." Can Fam Physician **53**(9): 1477-85.
Avorn, J. and S. B. Soumerai (1983). "Improving drug-therapy decisions through educational outreach. A randomized controlled trial of academically based "detailing"." N Engl J Med **308**(24): 1457-63.
BAEK (2006). Berufsordnung für die deutschen Ärztinnen und Ärzte.
BAEK (2007). Empfehlungen zur ärztlichen Fortbildung.
Barnett, G. O., R. N. Winickoff, et al. (1983). "A computer-based monitoring system for follow-up of elevated blood pressure." Med Care **21**(4): 400-9.
Barrows, H. S. (1994). Practice-Based Learning: Problem-Based Learning Applied to Medical Education. Springfield, Southern Illinois University School of Medicine.
Barrows, H. S., Tamblyn, R.M. (1980). Problem-Based Learning: An Approach to Medical Education. New York, Springer Publishing Company.
Bennett, N. L., L. L. Casebeer, et al. (2004). "Physicians' Internet information-seeking behaviors." J Contin Educ Health Prof **24**(1): 31-8.
Bennett, N. L., L. L. Casebeer, et al. (2006). "Information-seeking behaviors and reflective practice." J Contin Educ Health Prof **26**(2): 120-7.
Bernhardt, J. M., C. W. Runyan, et al. (2003). "Implementation and evaluation of a Web-based continuing education course in injury prevention and control." Health Promot Pract **4**(2): 120-8.
Bero, L. A., R. Grilli, et al. (1998). "Closing the gap between research and practice: an overview of systematic reviews of interventions to promote the implementation of research findings. The Cochrane Effective Practice and Organization of Care Review Group." Bmj **317**(7156): 465-8.
Bexell, A., E. Lwando, et al. (1996). "Improving drug use through continuing education: a randomized controlled trial in Zambia." J Clin Epidemiol **49**(3): 355-7.
Birmingham, C., McCord, M (2002). Group Process Research: Implications for Using Learning Groups. Team-based Learning - A Transformative Use of Small Groups. L. K. Michaelsen, Knight, A.B., Fink, L.D., Praeger Publishers.
Black, N. and A. Hutchings (2002). "Reduction in the use of surgery for glue ear: did national guidelines have an impact?" Qual Saf Health Care **11**(2): 121-4.

Bloom, B. S. (2005). "Effects of continuing medical education on improving physician clinical care and patient health: a review of systematic reviews." Int J Technol Assess Health Care **21**(3): 380-5.
Boissel, J. P., J. P. Collet, et al. (1995). "Education program for general practitioners on breast and cervical cancer screening: a randomized trial. PRE.SA.GF Collaborative Group." Rev Epidemiol Sante Publique **43**(6): 541-7.
Bordage, G., Brailovsky, C., Carretier, H., Page, G. (1995). "Content validation of key features on a national examination of clinical decision-making skills." Acad Med **70**(4): 276-281.
Bordage, G., Page, G. (1987). An alternate approach to PMPs, the key feature concept. Further Developments in Assessing Clinical Competence. I. Hart, Harden, R. Montreal, An-Heal Publications**:** 57-75.
Boud, D., Griffin, V (1987). Appreciating adults learning: grom the learner's perspective. London, Kogan Page.
Bransford, J. D. a. J., M.K. (1972). "Contextual Prerequisites fo Understanding: Some Investigations of Comprehension and Recall." Journal of Verbal Learning and Verbal Behavior **11**(6): 717-726.
Brennan, C. P. (1995). "Managed care and health information networks." J Health Care Finance **21**(4): 1-5.
Brooks, L. R. (1987). Journal of experimental psychology. Learning, memory, and cognition. Concepts and conceptual development. U. Nasser. Cambridge, Cambridge University Press**:** 141-174.
Brooks, L. R., Allen, S.W. and Norman G.R. (1991). "Rule and Instance-based Inference in Medical Diagnosis." Journal of experimental psychology. Learning, memory, and cognition **120**: 278-287.
Browner, W. S., R. B. Baron, et al. (1994). "Physician management of hypercholesterolemia. A randomized trial of continuing medical education." West J Med **161**(6): 572-8.
Bruner, J. S. (1961). "The act of discovery." Harvard Educational Revue **31**: 21-32.
Bull, F. C., C. L. Holt, et al. (2001). "Understanding the effects of printed health education materials: which features lead to which outcomes?" J Health Commun **6**(3): 265-79.
Burack, R. C., P. A. Gimotty, et al. (1998). "How reminders given to patients and physicians affected pap smear use in a health maintenance organization: results of a randomized controlled trial." Cancer **82**(12): 2391-400.
Burgers, J. S., R. P. Grol, et al. (2003). "Characteristics of effective clinical guidelines for general practice." Br J Gen Pract **53**(486): 15-9.
Campbell, H. S., S. W. Fletcher, et al. (1991). "Improving physicians' and nurses' clinical breast examination: a randomized controlled trial." Am J Prev Med **7**(1): 1-8.
Cantillon, P. and R. Jones (1999). "Does continuing medical education in general practice make a difference?" Bmj **318**(7193): 1276-9.
Carlsson, B., A. M. Gravgaard, et al. (1998). "Teaching oncology and cancer care to general practice trainees in Sweden: a two-year prospective, randomized study." J Cancer Educ **13**(1): 14-9.
Carney, P. A., A. J. Dietrich, et al. (1995). "A standardized-patient assessment of a continuing medical education program to improve physicians' cancer-control clinical skills." Acad Med **70**(1): 52-8.
Case, S. M., Swanson, D.B. (2001). Constructing Written Test Questions for the Basic and Clinical Sciences. N. B. o. M. Examiners. Philadelphia, National Board of Medical Examiners.
Casebeer, L., N. Bennett, et al. (2002). "Physician Internet medical information seeking and on-line continuing education use patterns." J Contin Educ Health Prof **22**(1): 33-42.

Catrambone, R., Holyoak, K.J. (1989). "Overcoming contextual limintations on problem-solving transfer." Journal of experimental psychology. Learning, memory, and cognition **15**(6): 1147-1156.
Chan, D. H., K. Leclair, et al. (1999). "Problem-based small-group learning via the Internet among community family physicians: a randomized controlled trial." MD Comput **16**(3): 54-8.
Choudhry, N. K., R. H. Fletcher, et al. (2005). "Systematic review: the relationship between clinical experience and quality of health care." Ann Intern Med **142**(4): 260-73.
Clark, N. M., M. Gong, et al. (1998). "Impact of education for physicians on patient outcomes." Pediatrics **101**(5): 831-6.
Cobb, S. C. (2004). "Internet continuing education for health care professionals: an integrative review." J Contin Educ Health Prof **24**(3): 171-80.
Cole, T. B. (1998). "Journal-based continuing medical education." J Med Pract Manage **14**(3): 123-6.
Cole, T. B. and R. M. Glass (2004). "Learning associated with participation in journal-based continuing medical education." J Contin Educ Health Prof **24**(4): 205-12.
Collins, J. (2004). "Education techniques for lifelong learning: principles of adult learning." Radiographics **24**(5): 1483-9.
Cramer, J. A. (2004). "A systematic review of adherence with medications for diabetes." Diabetes Care **27**(5): 1218-24.
Curran, V. R. and L. Fleet (2005). "A review of evaluation outcomes of web-based continuing medical education." Med Educ **39**(6): 561-7.
Davis, D. (1998). "Does CME work? An analysis of the effect of educational activities on physician performance or health care outcomes." Int J Psychiatry Med **28**(1): 21-39.
Davis, D., M. A. O'Brien, et al. (1999). "Impact of formal continuing medical education: do conferences, workshops, rounds, and other traditional continuing education activities change physician behavior or health care outcomes?" Jama **282**(9): 867-74.
Davis, D. A. and A. Taylor-Vaisey (1997). "Translating guidelines into practice. A systematic review of theoretic concepts, practical experience and research evidence in the adoption of clinical practice guidelines." Cmaj **157**(4): 408-16.
Davis, D. A., M. A. Thomson, et al. (1992). "Evidence for the effectiveness of CME. A review of 50 randomized controlled trials." Jama **268**(9): 1111-7.
Davis, D. A., M. A. Thomson, et al. (1995). "Changing physician performance. A systematic review of the effect of continuing medical education strategies." Jama **274**(9): 700-5.
De Groot, A. D. (1946). Het Denken van den Schaker (Thinking Processes in Chess Players). Den Haag, The Netherlands: Noord Holland.
De Volder, M. L., Schmidt, H.G., Moust, J.H.C., De Grave, W.S. (1986). Problem-based-learning and intrinsic motivation. Achievement and task motivation. J. H. C. van der Berchen, Bergen, Th.C.M., de Bruyn, E.E.I. Lisse, Swets & Zeitlinger and Swets North America.
Dee, C. and R. Blazek (1993). "Information needs of the rural physician: a descriptive study." Bull Med Libr Assoc **81**(3): 259-64.
Demakis, J. G., C. Beauchamp, et al. (2000). "Improving residents' compliance with standards of ambulatory care: results from the VA Cooperative Study on Computerized Reminders." Jama **284**(11): 1411-6.
Dickinson, J. C., G. A. Warshaw, et al. (1981). "Improving hypertension control: impact of computer feedback and physician education." Med Care **19**(8): 843-54.
Dietrich, A. J., G. T. O'Connor, et al. (1992). "Cancer: improving early detection and prevention. A community practice randomised trial." Bmj **304**(6828): 687-91.

Dolan, N. C., J. S. Ng, et al. (1997). "Effectiveness of a skin cancer control educational intervention for internal medicine housestaff and attending physicians." J Gen Intern Med **12**(9): 531-6.
Donner-Banzhoff, N. (2001). Professionelles Lernen - ein Leben lang
Stellungnahme zur ärztlichen Fortbildung. Deutsche Gesellschaft für Allgemeinmedizin und Familienmedizin (DEGAM).
Doucet, M. D., R. A. Purdy, et al. (1998). "Comparison of problem-based learning and lecture format in continuing medical education on headache diagnosis and management." Med Educ **32**(6): 590-6.
Duncker, K. (1945). "On Problem Solving." Psychological Monographs **58**: 270.
Dunn, S., P. Niday, et al. (1992). "The provision and evaluation of a neonatal resuscitation program." J Contin Educ Nurs **23**(3): 118-26.
Echlin, P. S., R. E. Upshur, et al. (2004). "Lack of chart reminder effectiveness on family medicine resident JNC-VI and NCEP III guideline knowledge and attitudes." BMC Fam Pract **5**: 14.
Eisenstaedt, R. S., W. E. Barry, et al. (1990). "Problem-based learning: cognitive retention and cohort traits of randomly selected participants and decliners." Acad Med **65**(9 Suppl): S11-2.
Ellis, J., I. Mulligan, et al. (1995). "Inpatient general medicine is evidence based. A-Team, Nuffield Department of Clinical Medicine." Lancet **346**(8972): 407-10.
Fahey, T., K. Schroeder, et al. (2006). "Interventions used to improve control of blood pressure in patients with hypertension." Cochrane Database Syst Rev(4): CD005182.
Farmer, A. (2003). "Printed educational materials: effects on professional practice and health care outcomes (Protocol)." The Cochrane Collaboration(3).
Fischer, M. R., S. Schauer, et al. (1996). "[CASUS model trial. A computer-assisted author system for problem-oriented learning in medicine]." Z Arztl Fortbild (Jena) **90**(5): 385-9.
Fordis, M., J. E. King, et al. (2005). "Comparison of the instructional efficacy of Internet-based CME with live interactive CME workshops: a randomized controlled trial." Jama **294**(9): 1043-51.
Foy, R., G. MacLennan, et al. (2002). "Attributes of clinical recommendations that influence change in practice following audit and feedback." J Clin Epidemiol **55**(7): 717-22.
Francke, A. L., J. B. Luiken, et al. (1997). "Effects of a continuing education program on nurses' pain assessment practices." J Pain Symptom Manage **13**(2): 90-7.
Frank, E., G. Baldwin, et al. (2000). "Continuing medical education habits of US women physicians." J Am Med Womens Assoc **55**(1): 27-8.
Franks, J. J., Bransford, J.D., and Auble, P.M. (1982). The Activation and Utilization of Knowledge. Handbook of Research Methods in Human Memory and Cognition. C. R. Puff. New York, Academic Press: 395-425.
Freemantle, N., E. L. Harvey, et al. (1997). "WITHDRAWN: Printed educational materials: effects on professional practice and health care outcomes." Cochrane Database Syst Rev(2): CD000172.
Garrard, J., V. Choudary, et al. (2006). "Organizational change in management of hepatitis C: evaluation of a CME program." J Contin Educ Health Prof **26**(2): 145-60.
Geddes, J. R., D. Game, et al. (1996). "What proportion of primary psychiatric interventions are based on evidence from randomised controlled trials?" Qual Health Care **5**(4): 215-7.
Gerlach, F. M. and M. Beyer (1999). "[Continuing medical education from the view of ambulatory care physicians--representative outcomes and needs in Bremen and Saxony-Anhalt]." Z Arztl Fortbild Qualitatssich **93**(8): 581-9.
Gesundheit, B. f. (2003). GKV-Modernisierungsgesetz.

Gick, M. L., and Holyoak, K.J. (1980). "Analogical Problem Solving." Cognitive Psychology **12**: 306-355.
Gick, M. L., and Holyoak, K.J. (1983). "Schema Induction and ANalogical Transfer." Cognitive Psychology **15**: 1-38.
Gill, P., A. C. Dowell, et al. (1996). "Evidence based general practice: a retrospective study of interventions in one training practice." Bmj **312**(7034): 819-21.
Glowniak, J. V. (1995). "Medical resources on the Internet." Ann Intern Med **123**(2): 123-31.
Goldberg, H. R. and G. M. McKhann (2000). "Student test scores are improved in a virtual learning environment." Adv Physiol Educ **23**(1): 59-66.
Gorman, P. N. and M. Helfand (1995). "Information seeking in primary care: how physicians choose which clinical questions to pursue and which to leave unanswered." Med Decis Making **15**(2): 113-9.
Goulet, F., R. J. Gagnon, et al. (1998). "Participation in CME activities." Can Fam Physician **44**: 541-8.
Green, M. L. and P. J. Ellis (1997). "Impact of an evidence-based medicine curriculum based on adult learning theory." J Gen Intern Med **12**(12): 742-50.
Greer, A. L. (1988). "The state of the art versus the state of the science. The diffusion of new medical technologies into practice." Int J Technol Assess Health Care **4**(1): 5-26.
Grilli, R., N. Freemantle, et al. (2000). "Mass media interventions: effects on health services utilisation." Cochrane Database Syst Rev(2): CD000389.
Grilli, R. and J. Lomas (1994). "Evaluating the message: the relationship between compliance rate and the subject of a practice guideline." Med Care **32**(3): 202-13.
Grimshaw, J. M. and I. T. Russell (1993). "Effect of clinical guidelines on medical practice: a systematic review of rigorous evaluations." Lancet **342**(8883): 1317-22.
Grol, R., J. Dalhuijsen, et al. (1998). "Attributes of clinical guidelines that influence use of guidelines in general practice: observational study." Bmj **317**(7162): 858-61.
Gullion, D. S., J. M. Tschann, et al. (1987). "Physicians' management of hypertension: a randomized controlled CME trial." Res Med Educ **26**: 115-20.
Guyatt, G., D. Feeny, et al. (1991). "Issues in quality-of-life measurement in clinical trials." Control Clin Trials **12**(4 Suppl): 81S-90S.
Hadiyono, J. E., S. Suryawati, et al. (1996). "Interactional group discussion: results of a controlled trial using a behavioral intervention to reduce the use of injections in public health facilities." Soc Sci Med **42**(8): 1177-83.
Hadjianastassiou, V. G., D. Karadaglis, et al. (2001). "A comparison between different formats of educational feedback to junior doctors: a prospective pilot intervention study." J R Coll Surg Edinb **46**(6): 354-7.
Haladyna, T. (2002). "A Review of Multiple-Choice Item-Writing Guidelines for Classroom Assessment." Appl Measurement in Edu **15**(3): 309-334.
Hall, J. C. and C. Platell (1997). "Half-life of truth in surgical literature." Lancet **350**(9093): 1752.
Harden, R. M. (2005). "A new vision for distance learning and continuing medical education." J Contin Educ Health Prof **25**(1): 43-51.
Harris, J. M., Jr., C. Novalis-Marine, et al. (2003). "Women physicians are early adopters of on-line continuing medical education." J Contin Educ Health Prof **23**(4): 221-8.
Hayes, J. R. a. S., H.A. (1977). Psychological Differences among Problem Isomorphs. Cognitive Theory. N. J. Castellan, Pison, D.B. and Potts, G.P. Hillsdale, Erlbaum.
Hayes, T. M., L. A. Allery, et al. (1990). "Continuing education for general practice and the role of the pharmaceutical industry." Br J Gen Pract **40**(341): 510-2.
Heale, J., D. Davis, et al. (1988). "A randomized controlled trial assessing the impact of problem-based versus didactic teaching methods in CME." Res Med Educ **27**: 72-7.

Herzig, S. (2003). "Freiwilliges Fortbildungszertifikat oder Pflichtfortbildung." Ärzteblatt Sachsen(05/2003): 165.
Hiss, R. G., MacDonald R, David WR (1978). Identification of physician educational influentials in small communitiy hospitals. . Seventeenth Annual Conference on Research in Medical Education, Washington DC.
Holmboe, E., R. Scranton, et al. (1998). "Effect of medical record audit and feedback on residents' compliance with preventive health care guidelines." Acad Med **73**(8): 901-3.
Hoskins, G., R. G. Neville, et al. (1997). "Does participation in distance learning and audit improve the care of patients with acute asthma attacks? The General Practitioners in Asthma Group." Health Bull (Edinb) **55**(3): 150-5.
Hunt, J. M. V. (1971). Intrinsic motivation: information and circumstances. Personality theory and information processing. H. M. Schroderen, Suedfeld, P. New York, Ronald.
Hysong, S. J., R. G. Best, et al. (2006). "Audit and feedback and clinical practice guideline adherence: Making feedback actionable." Implement Sci **1**: 9.
IAWF (1999). Kompetent prüfen. Bern/Wien, Institut für Aus-, Weiter- und Fortbildung, Medizinische Fakultät Universität Bern.
Jamtvedt, G., J. M. Young, et al. (2006). "Audit and feedback: effects on professional practice and health care outcomes." Cochrane Database Syst Rev(2): CD000259.
Jennett, P. A., O. E. Laxdal, et al. (1988). "The effects of continuing medical education on family doctor performance in office practice: a randomized control study." Med Educ **22**(2): 139-45.
Jennett, P. A., T. W. Wilson, et al. (1989). "Desirable behaviours in the office management of hypertension addressed through continuing medical education." Can J Public Health **80**(5): 359-62.
Johnson, T. (2000). "Functional health pattern assessment on-line. Lessons learned." Comput Nurs **18**(5): 248-54.
Jones, A., E. K. Carr, et al. (1998). "Positioning of stroke patients: evaluation of a teaching intervention with nurses." Stroke **29**(8): 1612-7.
Judge, J., T. S. Field, et al. (2006). "Prescribers' responses to alerts during medication ordering in the long term care setting." J Am Med Inform Assoc **13**(4): 385-90.
Kelly, M. H. and T. S. Murray (1994). "General practitioners' views on continuing medical education." Br J Gen Pract **44**(387): 469-71.
Khan, K. S. and A. Coomarasamy (2006). "A hierarchy of effective teaching and learning to acquire competence in evidenced-based medicine." BMC Med Educ **6**: 59.
Kimberlin, C. L., D. H. Berardo, et al. (1993). "Effects of an education program for community pharmacists on detecting drug-related problems in elderly patients." Med Care **31**(5): 451-68.
Kirkpatrick, D. (1994). Evaluating Training Programs: The Four Levels. . San Francisco, Berrett-Koehler.
Klemperer, D. (1996). "Qualität in der Medizin. Der patientenzentrierte Qualitätsbegriff und seine Implikationen." Dr. med. Mabuse: 22-27.
Knowles, M. S. (1970). The Modern Practice of Adult Education; Andragogy versus Pedagogy. New York, The Association Press.
Knowles, M. S. (1985). "Application in continuing education for the health professions: chapter five of "Andragogy in Action"." Mobius **5**(2): 80-100.
Kopp, V., Möltner, A., Fischer, M.R. (2006). "Key-Feature-Probleme zum Prüfen von prozeduralem Wissen: Ein Praxisleitfaden." GMS Zeitschrift für Medizinische Ausbildung **23**(3).
Korenstein, D., A. Dunn, et al. (2002). "Mixing it up: integrating evidence-based medicine and patient care." Acad Med **77**(7): 741-2.

Kottke, T. E., M. L. Brekke, et al. (1989). "A randomized trial to increase smoking intervention by physicians. Doctors Helping Smokers, Round I." Jama **261**(14): 2101-6.
Kreuter, M. W. (2001). "How do people process health information? Applications in an age of individualized communication." Current Directions in Psychological Science **10**(6): 206-9.
Kreuter, M. W. and V. J. Strecher (1996). "Do tailored behavior change messages enhance the effectiveness of health risk appraisal? Results from a randomized trial." Health Educ Res **11**(1): 97-105.
Langewitz, W. A., P. Eich, et al. (1998). "Improving communication skills--a randomized controlled behaviorally oriented intervention study for residents in internal medicine." Psychosom Med **60**(3): 268-76.
Lau, J., E. M. Antman, et al. (1992). "Cumulative meta-analysis of therapeutic trials for myocardial infarction." N Engl J Med **327**(4): 248-54.
Lecoq-D'Andre, F. and J. C. Balblanc (1999). "Continuing medical education for rheumatologists in France. Results of a national survey." Rev Rhum Engl Ed **66**(12): 717-23.
Lepper, M. D., Greene, D. (1975). "Turning play into work; effects of adult surveillance and extrinsic rewards on children's intrinsic motivation." Journal of Personality and Social Psychology **31**: 479-486.
Levinson, W. and D. Roter (1993). "The effects of two continuing medical education programs on communication skills of practicing primary care physicians." J Gen Intern Med **8**(6): 318-24.
Litzelman, D. K., R. S. Dittus, et al. (1993). "Requiring physicians to respond to computerized reminders improves their compliance with preventive care protocols." J Gen Intern Med **8**(6): 311-7.
Lomas, J. (1991). "Words without action? The production, dissemination, and impact of consensus recommendations." Annu Rev Public Health **12**: 41-65.
Maiman, L. A., M. H. Becker, et al. (1988). "Improving pediatricians' compliance-enhancing practices. A randomized trial." Am J Dis Child **142**(7): 773-9.
Mamary, E. M. and P. Charles (2000). "On-site to on-line: barriers to the use of computers for continuing education." J Contin Educ Health Prof **20**(3): 171-5.
Mann, C., C. Beziat, et al. (2001). "[Quality assurance program for postoperative pain management: impact of the Consensus Conference of the French Society of Anesthesiology and Intensive Care]." Ann Fr Anesth Reanim **20**(3): 246-54.
Manning, P. R. and L. DeBakey (2001). "Continuing medical education: the paradigm is changing." J Contin Educ Health Prof **21**(1): 46-54.
Mantovani, F., G. Castelnuovo, et al. (2003). "Virtual reality training for health-care professionals." Cyberpsychol Behav **6**(4): 389-95.
Marshall, J. N., M. Stewart, et al. (2001). "Small-group CME using e-mail discussions. Can it work?" Can Fam Physician **47**: 557-63.
Martenson, D., H. Eriksson, et al. (1985). "Medical chemistry: evaluation of active and problem-oriented teaching methods." Med Educ **19**(1): 34-42.
Martin, S. (2004). "Younger physicians, specialists use Internet more." Cmaj **170**(12): 1780.
Masic, I., A. Novo, et al. (2007). "How to assess and improve quality of medical education: lessons learned from Faculty of Medicine in Sarajevo." Bosn J Basic Med Sci **7**(1): 74-8.
Mazmanian, P. E., S. R. Daffron, et al. (1998). "Information about barriers to planned change: a randomized controlled trial involving continuing medical education lectures and commitment to change." Acad Med **73**(8): 882-6.

Mazzuca, S. A., G. J. Barger, et al. (1987). "Arthritis care in older-adult centers. A controlled study of an education program for public health nurses." Arthritis Rheum **30**(3): 275-80.
McCluskey, A. and M. Lovarini (2005). "Providing education on evidence-based practice improved knowledge but did not change behaviour: a before and after study." BMC Med Educ **5**: 40.
Messmer, P. R., S. Jones, et al. (1998). "Knowledge, perceptions, and practice of nurses toward HIV+/AIDS patients diagnosed with tuberculosis." J Contin Educ Nurs **29**(3): 117-25.
Michaelsen, L. K., Black, R.H., Watson, W.E. (1989). "A Realistic Test of Individual Versus Group Consensus Decision Making." Journal of Applied Psychology **74**(5): 834-839.
Michaelsen, L. K., Knight, A.B., Fink, L.D. (2002). Team-based Learning - A Transformative Use of Small Groups, Praeger Publishers.
Miller, G. E. (1990). "The assessment of clinical skills/competence/performance." Acad Med **65**(9 Suppl): S63-7.
Millis, B. J., Cotell, P.G. (1998). Cooperative Learning in Higher Education Today. Phoenix, American Council on Education/Oryx Press.
Mittman, B. S., X. Tonesk, et al. (1992). "Implementing clinical practice guidelines: social influence strategies and practitioner behavior change." QRB Qual Rev Bull **18**(12): 413-22.
Möbes, J. (2003). "Compliance: Neue Positionen am Beispiel des Diabetes mellitus." Zeitschrift für Allgemeinmedizin **05**.
Montgomery, A. A., T. Fahey, et al. (2000). "Evaluation of computer based clinical decision support system and risk chart for management of hypertension in primary care: randomised controlled trial." Bmj **320**(7236): 686-90.
Moore, D. A. and D. J. Klingborg (2003). "Using clinical audits to identify practitioner learning needs." J Vet Med Educ **30**(1): 57-61.
Needham, D. R. and I. M. Begg (1991). "Problem-oriented training promotes spontaneous analogical transfer: memory-oriented training promotes memory for training." Mem Cognit **19**(6): 543-57.
Nicholson, L. J., C. M. Warde, et al. (2007). "Faculty training in evidence-based medicine: improving evidence acquisition and critical appraisal." J Contin Educ Health Prof **27**(1): 28-33.
Nilasena, D. S. and M. J. Lincoln (1995). "A computer-generated reminder system improves physician compliance with diabetes preventive care guidelines." Proc Annu Symp Comput Appl Med Care: 640-5.
Norman, G. R. and H. G. Schmidt (1992). "The psychological basis of problem-based learning: a review of the evidence." Acad Med **67**(9): 557-65.
Nylenna, M. and O. G. Aasland (2000). "Primary care physicians and their information-seeking behaviour." Scand J Prim Health Care **18**(1): 9-13.
Ockene, I. S., J. R. Hebert, et al. (1996). "Effect of training and a structured office practice on physician-delivered nutrition counseling: the Worcester-Area Trial for Counseling in Hyperlipidemia (WATCH)." Am J Prev Med **12**(4): 252-8.
Ornstein, S. M., D. R. Garr, et al. (1991). "Computer-generated physician and patient reminders. Tools to improve population adherence to selected preventive services." J Fam Pract **32**(1): 82-90.
Osheroff, J. A., D. E. Forsythe, et al. (1991). "Physicians' information needs: analysis of questions posed during clinical teaching." Ann Intern Med **114**(7): 576-81.
Oxman, A. D., M. A. Thomson, et al. (1995). "No magic bullets: a systematic review of 102 trials of interventions to improve professional practice." Cmaj **153**(10): 1423-31.

Palmer, R. H., T. A. Louis, et al. (1985). "A randomized controlled trial of quality assurance in sixteen ambulatory care practices." Med Care **23**(6): 751-70.
Parker, M. T., N. Leggett-Frazier, et al. (1995). "The impact of an educational program on improving diabetes knowledge and changing behaviors of nurses in long-term care facilities." Diabetes Educ **21**(6): 541-5.
Patel, V. L., G. J. Groen, et al. (1991). "Effects of conventional and problem-based medical curricula on problem solving." Acad Med **66**(7): 380-9.
Patwardhan, M. B., G. P. Samsa, et al. (2006). "Changing physician knowledge, attitudes, and beliefs about migraine: evaluation of a new educational intervention." Headache **46**(5): 732-41.
Pekarik, G. (1994). "Effects of brief therapy training on practicing psychotherapists and their clients." Community Ment Health J **30**(2): 135-44.
Perera, D. R., J. P. LoGerfo, et al. (1983). "Teaching sigmoidoscopy to primary care physicians: a controlled study of continuing medical education." J Fam Pract **16**(4): 785-8.
Peterson, M. W., J. R. Galvin, et al. (1999). "Realizing the promise: delivering pulmonary continuing medical education over the Internet." Chest **115**(5): 1429-36.
Premi, J., S. Shannon, et al. (1994). "Practice-based small-group CME." Acad Med **69**(10): 800-2.
Quirk, M., J. Ockene, et al. (1991). "Training family practice and internal medicine residents to counsel patients who smoke: improvement and retention of counseling skills." Fam Med **23**(2): 108-11.
Qureshi, N. N., J. Hatcher, et al. (2007). "Effect of general practitioner education on adherence to antihypertensive drugs: cluster randomised controlled trial." Bmj **335**(7628): 1030.
Reddy, H., I. Harris, et al. (2001). "Continuing medical education. What do Minnesota physicians want?" Minn Med **84**(3): 58-61.
Rogers, J. L., O. M. Haring, et al. (1982). "Medical information systems: assessing impact in the areas of hypertension, obesity and renal disease." Med Care **20**(1): 63-74.
Rossi, R. A. and N. R. Every (1997). "A computerized intervention to decrease the use of calcium channel blockers in hypertension." J Gen Intern Med **12**(11): 672-8.
Roter, D. L., K. A. Cole, et al. (1990). "An evaluation of residency training in interviewing skills and the psychosocial domain of medical practice." J Gen Intern Med **5**(4): 347-54.
Roter, D. L., J. A. Hall, et al. (1995). "Improving physicians' interviewing skills and reducing patients' emotional distress. A randomized clinical trial." Arch Intern Med **155**(17): 1877-84.
Rothenberg, E., M. Wolk, et al. (1982). "Continuing medical education in New York County: physician attitudes and practices." J Med Educ **57**(7): 541-9.
Rowan, M. S., W. Hogg, et al. (2006). "Family physicians' reactions to performance assessment feedback." Can Fam Physician **52**(12): 1570-1.
Sackett, D. L. (1997). "Evidence-based medicine." Semin Perinatol **21**(1): 3-5.
Sackett, D. L., W. M. Rosenberg, et al. (1996). "Evidence based medicine: what it is and what it isn't." Bmj **312**(7023): 71-2.
Sargeant, J., V. Curran, et al. (2004). "Interactive on-line continuing medical education: physicians' perceptions and experiences." J Contin Educ Health Prof **24**(4): 227-36.
Saturno, P. J. (1995). "Training health professionals to implement quality improvement activities. Results of a randomized controlled trial after one year of follow-up." Int J Qual Health Care **7**(2): 119-26.
Schilling, K., J. Wiecha, et al. (2006). "An interactive web-based curriculum on evidence-based medicine: design and effectiveness." Fam Med **38**(2): 126-32.

Schmidt, H. G. (1993). "Foundations of problem-based learning: some explanatory notes." Med Educ **27**(5): 422-32.
Schmidt, H. G., M. Machiels-Bongaerts, et al. (1996). "The development of diagnostic competence: comparison of a problem-based, an integrated, and a conventional medical curriculum." Acad Med **71**(6): 658-64.
Schmidt, H. G., Moust, J.H.C. (2000). "Towards a Taxonomy of Problems. Used in Problem-Based Learning Curricula." J Excell Coll Tech **11**(2): 57-72.
Schmidt, H. G., G. R. Norman, et al. (1990). "A cognitive perspective on medical expertise: theory and implication." Acad Med **65**(10): 611-21.
Scolapio, J. S., W. Deluise, et al. (2007). "Continued medical education provided by medical journals: the "red journal's" experience." Am J Gastroenterol **102**(8): 1590-3.
Shakespeare, T. P., R. K. Mukherjee, et al. (2005). "Evaluation of an audit with feedback continuing medical education program for radiation oncologists." J Cancer Educ **20**(4): 216-21.
Shea, S., W. DuMouchel, et al. (1996). "A meta-analysis of 16 randomized controlled trials to evaluate computer-based clinical reminder systems for preventive care in the ambulatory setting." J Am Med Inform Assoc **3**(6): 399-409.
Shuval, K., E. Berkovits, et al. (2007). "Evaluating the impact of an evidence-based medicine educational intervention on primary care doctors' attitudes, knowledge and clinical behaviour: a controlled trial and before and after study." J Eval Clin Pract **13**(4): 581-98.
Sibley, J. C., D. L. Sackett, et al. (1982). "A randomized trial of continuing medical education." N Engl J Med **306**(9): 511-5.
Simonsohn, A. B. and M. R. Fischer (2004). "[Evaluation of a case-based computerized learning program (CASUS) for medical students during their clinical years]." Dtsch Med Wochenschr **129**(11): 552-6.
Sklar, B. (2001). "The current status of online CME."
Slotnick, H. B. (1996). "How doctors learn: the role of clinical problems across the medical school-to-practice continuum." Acad Med **71**(1): 28-34.
Slotnick, H. B. (1999). "How doctors learn: physicians' self-directed learning episodes." Acad Med **74**(10): 1106-17.
Smith, D. K., R. W. Shaw, et al. (1995). "Training obstetricians and midwives to present screening tests: evaluation of two brief interventions." Prenat Diagn **15**(4): 317-24.
Sohn, W., A. I. Ismail, et al. (2004). "Efficacy of educational interventions targeting primary care providers' practice behaviors: an overview of published systematic reviews." J Public Health Dent **64**(3): 164-72.
Soumerai, S. B. and J. Avorn (1990). "Principles of educational outreach ('academic detailing') to improve clinical decision making." Jama **263**(4): 549-56.
Soumerai, S. B., T. J. McLaughlin, et al. (1989). "Improving drug prescribing in primary care: a critical analysis of the experimental literature." Milbank Q **67**(2): 268-317.
Spilich, G. J., Vesonder, G.T., Chiese, H.L. and Voss, J.F. (1972). "Text Processing of Domain-related Information for Individuals with High and Low Domain Knowledge." Journal of Verbal Learning and Verbal Behavior **18**: 352-373.
Stancic, N., P. D. Mullen, et al. (2003). "Continuing medical education: what delivery format do physicians prefer?" J Contin Educ Health Prof **23**(3): 162-7.
Strecher, V. J., M. S. O'Malley, et al. (1991). "Can residents be trained to counsel patients about quitting smoking? Results from a randomized trial." J Gen Intern Med **6**(1): 9-17.
Stross, J. K. (1983). "Maintaining competency in advanced cardiac life support skills." Jama **249**(24): 3339-41.

Sulmasy, D. P., G. Geller, et al. (1992). "The quality of mercy. Caring for patients with 'do not resuscitate' orders." Jama **267**(5): 682-6.

Sulmasy, D. P., K. Y. Song, et al. (1996). "Strategies to promote the use of advance directives in a residency outpatient practice." J Gen Intern Med **11**(11): 657-63.

Tans, R. W., Schmidt, H.G., Schade-Hoogeveen, B.E.J., and Gijselaers, W.H. (1986). "Directing the Learning Process by Means of Problems: A Field Experiment." Tijdschrift voor Onderwijsresearch **11**: 35-46.

Terry, P. B., V. L. Wang, et al. (1981). "A continuing medical education program in chronic obstructive pulmonary diseases: design and outcome." Am Rev Respir Dis **123**(1): 42-6.

Thomson O'Brien, M. A., N. Freemantle, et al. (2001). "Continuing education meetings and workshops: effects on professional practice and health care outcomes." Cochrane Database Syst Rev(2): CD003030.

Thomson O'Brien, M. A., A. D. Oxman, et al. (2000). "Audit and feedback: effects on professional practice and health care outcomes." Cochrane Database Syst Rev(2): CD000259.

Thomson O'Brien, M. A., A. D. Oxman, et al. (2000). "Local opinion leaders: effects on professional practice and health care outcomes." Cochrane Database Syst Rev(2): CD000125.

Tredwin, C. J., A. Eder, et al. (2005). "British Dental Journal based Continuing Professional Development: a survey of participating dentists and their views." Br Dent J **199**(10): 665-9, discussion 654.

Walton, H. J. and M. B. Matthews (1989). "Essentials of problem-based learning." Med Educ **23**(6): 542-58.

Ward, J. and R. Sanson-Fisher (1996). "Does a 3-day workshop for family medicine trainees improve preventive care? A randomized control trial." Prev Med **25**(6): 741-7.

Watson, W. E., Kumar, K., Michaelsen, L.K. (1993). "Cultural diversity's impact on interaction process and performance: Comparing homogeneous and diverse task groups." Academy of Management Journal **36**(3): 590-602.

Weatherall, D. J. (1994). "The inhumanity of medicine." Bmj **309**(6970): 1671-2.

Wensing, M., T. van der Weijden, et al. (1998). "Implementing guidelines and innovations in general practice: which interventions are effective?" Br J Gen Pract **48**(427): 991-7.

Westphal, M. F., J. A. Taddei, et al. (1995). "Breast-feeding training for health professionals and resultant institutional changes." Bull World Health Organ **73**(4): 461-8.

White, C. W., M. A. Albanese, et al. (1985). "The effectiveness of continuing medical education in changing the behavior of physicians caring for patients with acute myocardial infarction. A controlled randomized trial." Ann Intern Med **102**(5): 686-92.

Wilson, D. M. C., Ciliska, D., Singer, J., Williams, K., Alleyne J, Lindsay E. (1992). "Family Physicians and Exercise Counseling Can they be influenced to provide more?" Can Fam Physician **38**: 2003-2010

Wirtschafter, D. D., J. Sumners, et al. (1986). "Continuing medical education using clinical algorithms. A controlled-trial assessment of effect on neonatal care." Am J Dis Child **140**(8): 791-7.

Wood, P. R., J. H. Littlefield, et al. (1989). "Telephone management curriculum for pediatric interns: a controlled trial." Pediatrics **83**(6): 925-30.

Wutoh, R., S. A. Boren, et al. (2004). "eLearning: a review of Internet-based continuing medical education." J Contin Educ Health Prof **24**(1): 20-30.

Youl, P. H., B. A. Raasch, et al. (2007). "The effect of an educational programme to improve the skills of general practitioners in diagnosing melanocytic/pigmented lesions." Clin Exp Dermatol **32**(4): 365-70.
Young, W. F., Jr. (2003). "Minireview: primary aldosteronism--changing concepts in diagnosis and treatment." Endocrinology **144**(6): 2208-13.
Zwar, N. A., J. J. Gordon, et al. (1995). "Evaluation of an educational program in rational prescribing for GP trainees." Aust Fam Physician **24**(5): 833-8.

VI. Anhang

Anhang 1 – Eingangsfragebogen

Eingangs-Evaluation zum Intensivseminar Diabetologie am 19.07.2006

Bitte füllen Sie den Fragebogen sorgfältig aus. Sie helfen uns damit, wichtige Anregungen zur Verbesserung des Seminars zu erhalten. Wir danken Ihnen für Ihre Mitarbeit!

Code:	______________	______________	______________
	1. Buchstabe des Geburtsnamens Ihrer Mutter	1. Buchstabe Ihres Geburtsorts	Ihre Hausnummer

Geschlecht:

- ❑ weiblich
- ❑ männlich

Alter: ______Jahre

Ich bin

- ❑ Facharzt/ärztin für Allgemeinmedizin
- ❑ Facharzt/ärztin für Innere Medizin
- ❑ Facharzt für __________________
- ❑ Arzt/Ärztin in Weiterbildung

Ich bin Facharzt seit _____ Jahren

Ich bin im _____. Jahr meiner Weiterbildung

Ich habe mich

- ❑ nur für das heutige Einzelseminar angemeldet
- ❑ für den Block Endokrinologie angemeldet (3 Veranstaltungen)
- ❑ für den Block Diabetologie angemeldet (3 Veranstaltungen)
- ❑ für das Gesamtprogramm angemeldet (6 Veranstaltungen)

Im IV. Quartal 2005 habe ich etwa _____ endokrinologische Fälle und etwa _____ Diabetes-Patienten behandelt.

Ich habe 2005 an Fortbildungen zum Thema Endokrinologie/Diabetologie teilgenommen:

❍ Nein ❍ Ja, einmal ❍ Ja, 2-4mal ❍ Ja, mehr als 4mal

Wenn ja, wie viele CME Punkte haben Sie damit erworben? ________ Punkte

Wie viele CME-Punkte haben Sie 2005 insgesamt mit welcher Veranstaltungsart erworben?
_____ CME-Punkte online
_____ CME-Punkte per Fax
_____ CME-Punkte über Präsenzveranstaltungen

Ich habe 2006 bereits an Fortbildungsveranstaltungen teilgenommen oder habe mich für Fortbildungen angemeldet.

❍ Nein ❍ Ja ❍ Wenn ja, zu welchen Themen? ________________________________

Vom Intensivseminar Praktische Endokrinologie und Diabetologie habe ich erfahren ...

- ❑ durch eine schriftliche Einladung zum Seminar (Broschüre)
- ❑ von Kollegen
- ❑ über E-mail
- ❑ über Internet
- ❑ _______________

**Zur Anmeldung zum Intensivseminar Praktische Endokrinologie und Diabetologie haben mich bewogen**	**stimmt über-haupt nicht**					**stimmt genau**
Themenschwerpunkte der Fortbildung	❍	❍	❍	❍	❍	❍
Interaktivität und Fallorientierung	❍	❍	❍	❍	❍	❍
Auswahl der Referenten	❍	❍	❍	❍	❍	❍
Zeitlicher Rahmen	❍	❍	❍	❍	❍	❍
Sonstige Gründe, und zwar						
**Von dieser Fortbildungsveranstaltung erwarte ich….**	**stimmt über-haupt nicht**					**stimmt genau**
Theoretischen Wissenszuwachs	❍	❍	❍	❍	❍	❍
Konkrete Entscheidungshilfen für die Praxis	❍	❍	❍	❍	❍	❍
Neueste Erkenntnisse aus der Wissenschaft	❍	❍	❍	❍	❍	❍
Kontakt mit Kollegen	❍	❍	❍	❍	❍	❍
Kennenlernen einer neuen Fortbildungsform	❍	❍	❍	❍	❍	❍
Sonstige Gründe, und zwar						
	**stimmt über-haupt nicht**					**stimmt genau**
Ich bin an endokrinologischen und diabetologischen Themen interessiert.	❍	❍	❍	❍	❍	❍
Es macht mir Spaß, mich mit Inhalten der Endokrinologie und Diabetologie zu beschäftigen.	❍	❍	❍	❍	❍	❍
Das Thema Endokrinologie und Diabetologie ist für meine Tätigkeit von hoher Relevanz.	❍	❍	❍	❍	❍	❍
Ich würde gern mehr über Endokrinologie/Diabetologie wissen, als ich jetzt weiß.	❍	❍	❍	❍	❍	❍
Für mich ist es eine Herausforderung, mehr über Endokrinologie/Diabetologie zu lernen.	❍	❍	❍	❍	❍	❍
Mein jetziges Wissen über die theoretischen Grundlagen der Endokrinologie/Diabetologie schätze ich als gering ein.	❍	❍	❍	❍	❍	❍
Mein jetziges Wissen über die Diagnostik und Therapie von Patienten mit diabetologischen Erkrankungen schätze ich als gering ein.	❍	❍	❍	❍	❍	❍

Arbeiten Sie selbst mit dem Computer? ❍ nein ❍ ja, privat ❍ ja, beruflich

Wie viel Zeit in Stunden verbringen Sie durchschnittlich pro Woche am Computer?_______ Stunden

Was haben Sie selbst an einem Computer bereits gemacht?
(Hier können mehrere Felder angekreuzt werden)

❑	Texte geschrieben	❑	programmiert
❑	Computerspiele gespielt	❑	Grafiken oder Diagramme erstellt
❑	mit Datenbanken gearbeitet	❑	mit Tabellenkalkulationen gearbeitet
❑	ein Programm installiert	❑	HTML geschrieben
❑	Lernprogramme genutzt	❑	(eigene) Webseiten gestaltet
❑	Videos oder Bilder angesehen	❑	Musik gehört
❑	Nachschlagwerke genutzt (z.B. Lexika, Wörterbücher)	❑	Musik gemacht
❑	Statistikprogramme genutzt		sonstiges:_______________

Wie schätzen Sie Ihre Computerkenntnisse ein?

❍sehr gut ❍ eher gut ❍ mittelmäßig ❍ eher schlecht ❍ sehr schlecht

Kommentare und Anregungen zu dieser Fortbildung:

____

____

____

____

____

____

____

____

Anhang 2 – Abschlussfragebogen

Abschluss-Evaluation zum Intensivseminar Diabetologie am 19.07.2006

Bitte füllen Sie den Fragebogen sorgfältig aus. Sie helfen uns damit, wichtige Anregungen zur Verbesserung des Seminars zu erhalten. Wir danken Ihnen für Ihre Mitarbeit!

Code: _____________ _____________ _____________

1. Buchstabe des Geburts-namens Ihrer Mutter	1. Buchstabe Ihres Geburts-orts	Ihre Haus-nummer

	**stimmt über-haupt nicht**				**stimmt genau**
Insgesamt hat mir das Seminar Spaß gemacht.	❍	❍	❍	❍	❍	❍
Die Inhalte waren für mein Vorwissen angemessen schwierig.	❍	❍	❍	❍	❍	❍
Das Seminar war abwechslungsreich.	❍	❍	❍	❍	❍	❍
Die Fälle haben mich zum Mitdenken angeregt.	❍	❍	❍	❍	❍	❍
Ich habe in diesem Seminar viel gelernt.	❍	❍	❍	❍	❍	❍
Die erlernten Inhalte werden mein Verhalten in der Praxis verändern.	❍	❍	❍	❍	❍	❍
Insgesamt hat das Seminar meine Erwartungen erfüllt.	❍	❍	❍	❍	❍	❍
Ich hätte mir mehr konkrete Entscheidungshilfen für die Praxis gewünscht.	❍	❍	❍	❍	❍	❍
Ich habe mehr theoretischen Wissenszuwachs vom Seminar erwartet.	❍	❍	❍	❍	❍	❍

Ich habe Interesse, Online-Lernfälle als Nachbearbeitung zu bearbeiten.

❍nein ❍ja

Kommentare und Anregungen zum heutigen Seminar:

____
____
____
____
____
____
____
____

Anhang 3 - Aufbau des Syllabus

1. Übersicht über das Veranstaltungsprogramm

2. Erster Themenschwerpunkt:

2.1. 2-5 Seiten mit den abgedruckten Originalfolien des Einführungsvortrags mit genügend Platz für Notizen

2.2. Falldarstellungen:

3-5 Seiten zu einem Fall mit Anamnese, Befunden, 2-3 Fragen und zum Teil weiteren Befunden und Verläufen

2-4 Fälle hintereinander mit gleichem Aufbau

2.3. 10-20 Seiten mit den abgedruckten Originalfolien des zusammenfassenden Vortrags

3. Zweiter Themenschwerpunkt:

3.1. 2-5 Seiten mit den abgedruckten Originalfolien des Einführungsvortrags mit genügend Platz für Notizen

3.2. Falldarstellungen:

3-5 Seiten zu einem Fall mit Anamnese, Befunden, 2-3 Fragen und zum Teil weiteren Befunden und Verläufen

2-4 Fälle hintereinander mit gleichem Aufbau

3.3. 10-20 Seiten mit den abgedruckten Originalfolien des zusammenfassenden Vortrags

4. Verläufe der Fälle (für die Besprechung der Lösungsvorschläge im Plenum mit Dozenten)

Anhang 4 - Instruktionen für Referenten und Moderatoren

Ziel dieser Veranstaltung ist es, die aktuellen therapeutischen und diagnostischen Leitlinien zu präsentieren und den Teilnehmern Gelegenheit zu geben, dazu praktische Fragen zu stellen. Die Fortbildung ist folgendermaßen aufgebaut: Nach der Begrüßung und einem Vorwissenstest folgt der Einführungsvortrag (15 Minuten). Dann bearbeiten die Teilnehmer etwa 20-25 Minuten lang in Kleingruppen 2-3 Fälle zum Thema, die in der anschließenden Diskussion (ca. 30 Minuten) im Plenum intensiv diskutiert werden. Dazu würden wir Sie bitten, 2-3 klinische Kurzfälle zum Thema Ihres Vortrags mit integrierten Fragen an die Teilnehmer (z.B. zum Vorgehen, zur Therapieempfehlung, etc.) zu erstellen.

Für den Vor- und Nachwissenstest im Multiple-Choice-Fragenformat teilen wir außerdem zu Beginn der Veranstaltung TED-Geräte aus, so dass eine Lernerfolgskontrolle – wie von den Teilnehmern erwünscht – möglich ist. Dazu bräuchten wir etwa 5 MC-Fragen zu Ihrem Thema. Beachten Sie bitte dabei, dass die Fragen klar gestellt werden und die 6 Antwortmöglichkeiten kurz ausfallen (es darf nur EINE Antwort richtig sein - diese bitte unbedingt markieren!).

Jeder Teilnehmer erhält einen Syllabus mit dem Ausdruck der PowerPoint-Dateien der einzelnen Vorträge. Wir bitten Sie daher um zwei Punkte:
1.) Könnten Sie uns bitte Ihre PowerPoint-Datei mit den Fällen am besten elektronisch zukommen lassen (wir müssen diese dann kopieren und binden).

2.) Könnten Sie uns bitte fünf Fragen mit Kennzeichnung der korrekten Antwort zu Ihrem Thema vorbereiten. Die Fragen sollen mit dem TED-System beantwortet werden, das heißt, es gibt 6 Antwortmöglichkeiten ohne Kombinationen (d.h. auch hier darf nur eine Antwort richtig sein).“

Anhang 5 - Themen bereits besuchter Fortbildungs-veranstaltungen bzw. geplanter Fortbildungen

Nennungen insgesamt 265	Häufigkeit	Prozent
Diabetes mellitus	38	14, 34
Innere Medizin allgemein	25	9,43
Kardiologie	24	9,06
Infektiologie, Reisemedizin, Impfungen	15	5,66
Arterielle Hypertonie	14	5,28
Schmerz	13	4,90
Endokrinologie (Schilddrüse)	11	4,15
diverse	10	3,77
Orthopädie, Sportmedizin	9	3,40
Geriatrie, Demenz	9	3,40
Pneumologie (Asthma, COPD)	8	3,02
Rheumatologie	8	3,02
Allgemeinmedizin	8	3,02
Akupunktur	7	2,64
Psychiatrie, Psychosomatik, Psychotherapie	7	2,64
Gastroenterologie	6	2,26
Neurologie	6	2,26
Notfallmedizin	5	1,89
Qualitätsmanagement, Qualitätszirkel	5	1,89
Nephrologie, Osteoporose, diese Reihe, 1. HCM-FB, Curriculum, Sonographie, Palliativmedizin, Chirurgie	4	1,50
Homöopathie, Ernährungsmedizin	2	0,75
Adipositas, Hygiene, Augen, Krebs, Pädiatrie, BWL für Praxis, Gynäkologie, Kommunikation, Gerinnung	1	0,37

Anhang 6 - Erstellung der Onlinefälle:

Diabetologie 1 – Diabetes und Adipositas: 4 Fälle (2 zu Diabetes und 2 zur Adipositas), Zeitaufwand: ca. 12 Stunden

Diabetologie 2 – Insulintherapie und Diabetisches Fußsyndrom: 5 Fälle (2 zu Insulintherapie und 3 zum Diabetischen Fußsyndrom), Zeitaufwand: ca. 14 Stunden

Diabetologie 3 – Mikro- und Makrovaskuläre Komplikationen: 4 Fälle (2 zu mikrovaskulären Komplikationen und 2 zu makrovaskulären Komplikationen), Zeitaufwand: ca. 13 Stunden

Endokrinologie 1 – Therapierefraktäre arterielle Hypertonie und komplizierter Schilddrüsenknoten: 8 Fälle (4 Kurzfälle zur arteriellen Hypertonie und 4 Kurzfälle zum komplizierten Schilddrüsenknoten), Zeitaufwand: ca. 18 Stunden

Endokrinologie 2 – Osteoporose und Nebennierenzufallstumor: 4 Fälle (2 Fälle zur Osteoporose und 2 Fälle zum Nebennierenzufallstumor), Zeitaufwand: 12 Stunden

Endokrinologie 3 – Hypophyse, erektile Dysfunktion, Anti-Aging: 5 Fälle (2 Fälle zu Hypophysenerkrankungen, 2 Fälle zur erektilen Dysfunktion und 1 zu Anti-Aging), Zeitaufwand: 20 Stunden

Zeitaufwand insgesamt: ca. 90 Stunden, durchschnittlich 15 h

2. Review der Onlinefälle (von Angelika Simonsohn und Thomas Eversmann)

Angelika Simonsohn (pädagogisches Review): Zeitaufwand: ca.15 Stunden

Thomas Eversmann (inhaltliches Review): Zeitaufwand: ca. 5 Stunden

3. Organisation und technische Umsetzung (von Inga Hege und Martin Adler)

Zeitaufwand: ca. 10 Stunden

VII. Lebenslauf

Lisa Kühne-Eversmann, geb. Eversmann
Geboren am 26.11.1979 in München
Eltern: Dr. med. Thomas und Dr. phil. Susanne Eversmann
Ehegatte: Florian Kühne

Ausbildung:

1986-1990 Grundschule an der Haimhauserstrasse in München
1990-1999 St.-Anna-Gymnasium, München
1999 Abitur

ab 1999 **Studium der Humanmedizin**
erstes Semester in Halle/Saale
ab 2. Semester Ludwig-Maximilians-Universität, München
2001 Physikum
2002 Erstes Staatsexamen
2005 Zweites Staatsexamen
2006 Drittes Staatsexamen (Gesamtnote 1,6)
2006 Approbation als Ärztin (16. Juni)

Medizinische Praktika:

2002 Famulatur in einer Schwerpunktpraxis für Innere Medizin, Endokrinologie und Diabetologie in München
2003 Famulatur in Allgemeinchirurgie und Notfallmedizin im Krankenhaus Elliot Lake, Kanada
2004 Famulatur in Anästhesie und Intensivmedizin im Krankenhaus Brixen, Italien
2004 Famulatur am Deutschen Herzzentrum Berlin
2005 Praktisches Jahr:
Tertial Innere Medizin in der Medizinischen Klinik Innenstadt in München
Tertial Anästhesie in der Klinik für Anästhesiologie der LMU München
Tertial Chirurgie im Städtischen Krankenhaus Neuperlach und im Krankenhaus der Medunsa-Universität in Pretoria, Südafrika

Berufliche Laufbahn:

Seit 1. Oktober 2006 Assistenzärztin (Facharztweiterbildung für Innere Medizin) und wissenschaftliche Mitarbeiterin am Schwerpunkt Medizindidaktik der Medizinischen Klinik Innenstadt, Klinikum der Universität München, Ziemssenstr. 1.
